# Supplementary figures and images for: LncRNA Snhg3 aggravates hepatic steatosis via PPARγ signaling (part 1 of 2)
Source: eLife. 2024 Oct 22;13:RP96988. doi: 10.7554/eLife.96988 (PMC11495842; doi:10.7554/eLife.96988)

Figure 5E  
repeat 1.

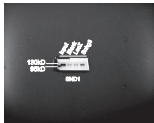

2.

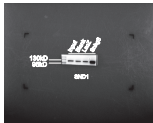

3.

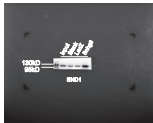

Supplement: Figure 5—source data 3. [file elife-96988-fig5-data3.pdf]

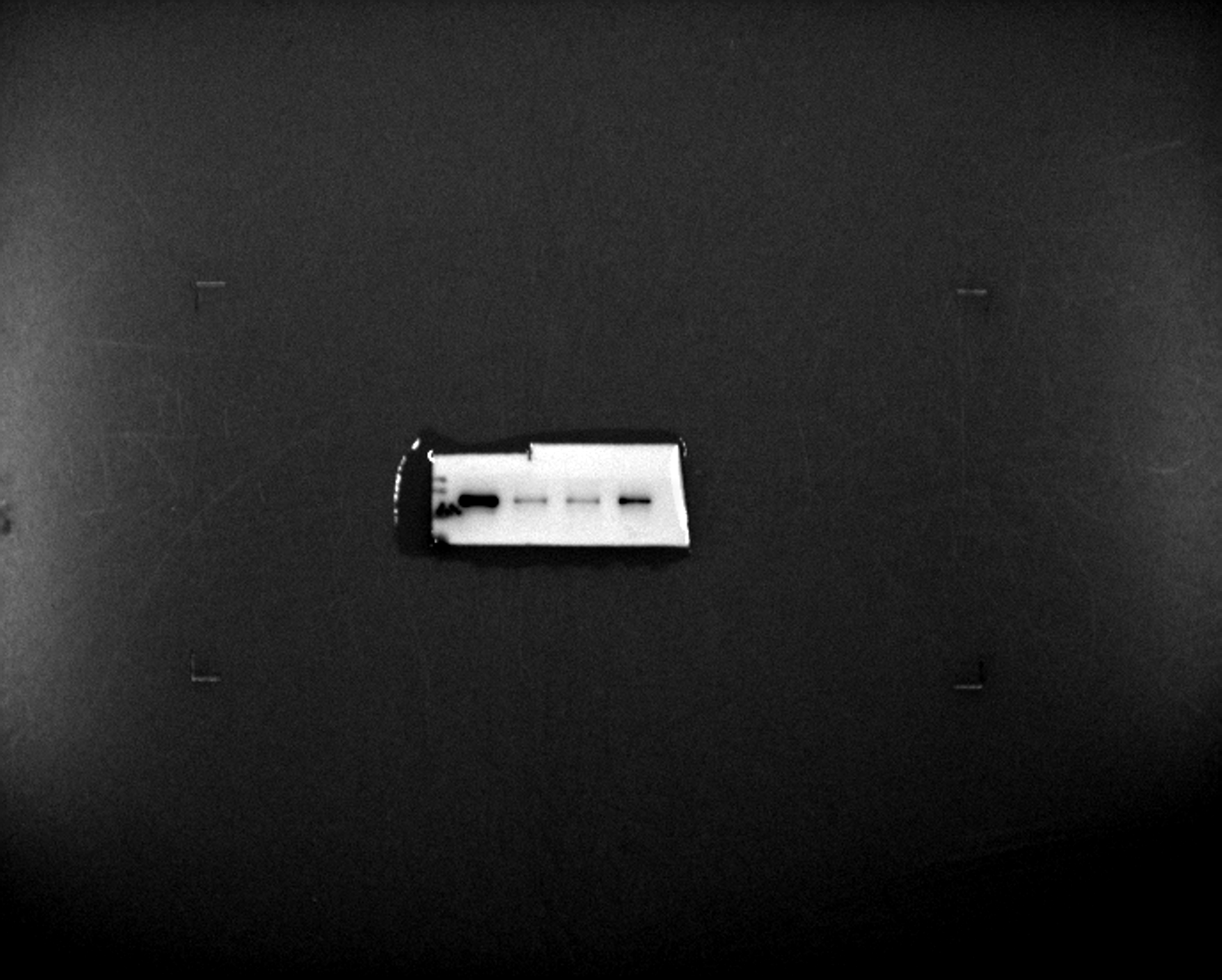

Supplement: Figure 5—source data 4. [file elife-96988-fig5-data4.zip › Figure 5-source data 4/1/SND1.Tif]

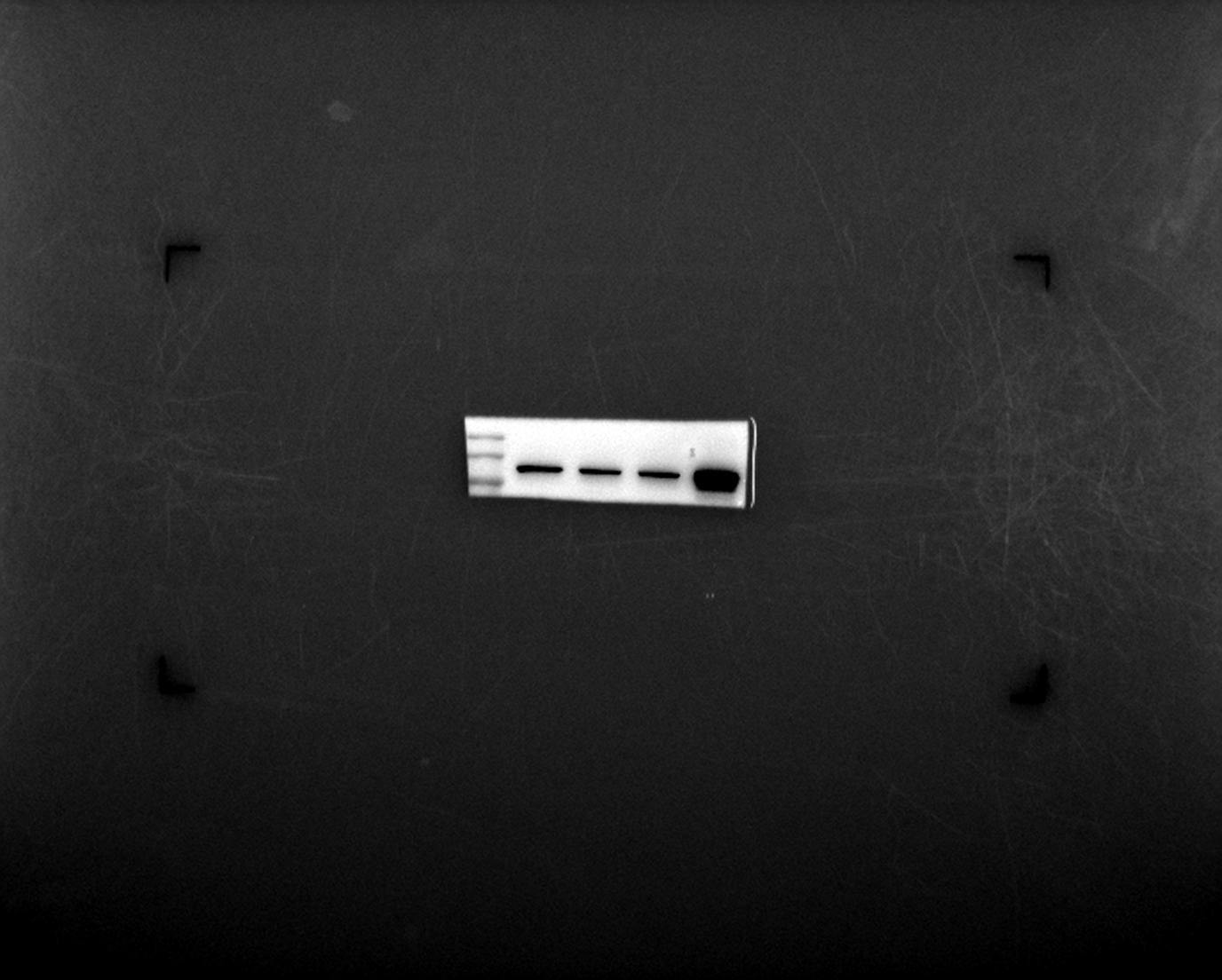

Supplement: Figure 5—source data 4. [file elife-96988-fig5-data4.zip › Figure 5-source data 4/2/SND1.tif]

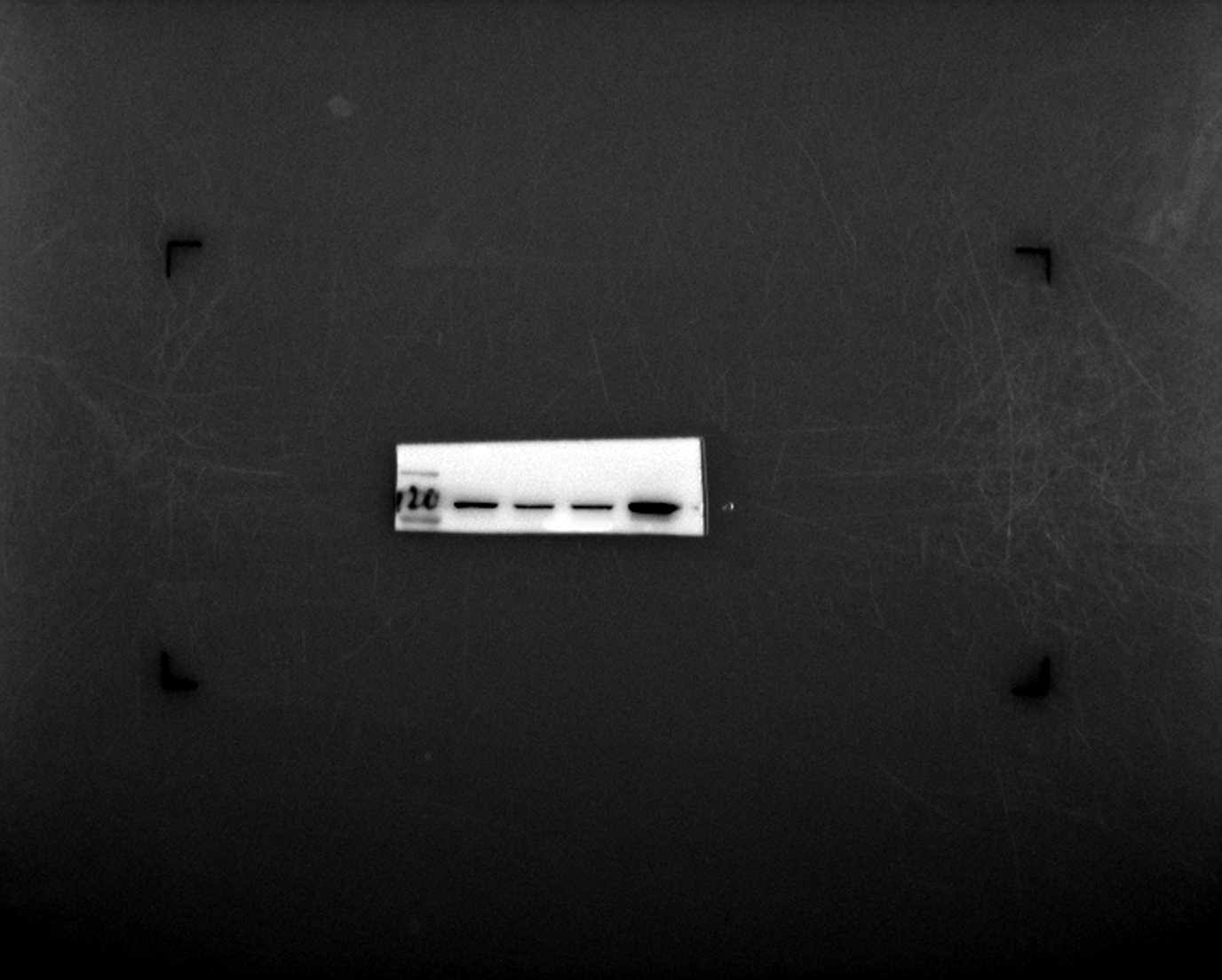

Supplement: Figure 5—source data 4. [file elife-96988-fig5-data4.zip › Figure 5-source data 4/3/SND1.tif]

Figure 5G

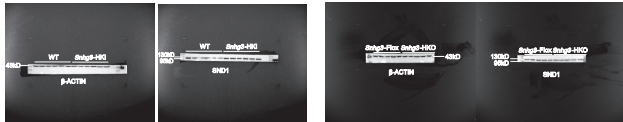

Supplement: Figure 5—source data 5. [file elife-96988-fig5-data5.pdf]

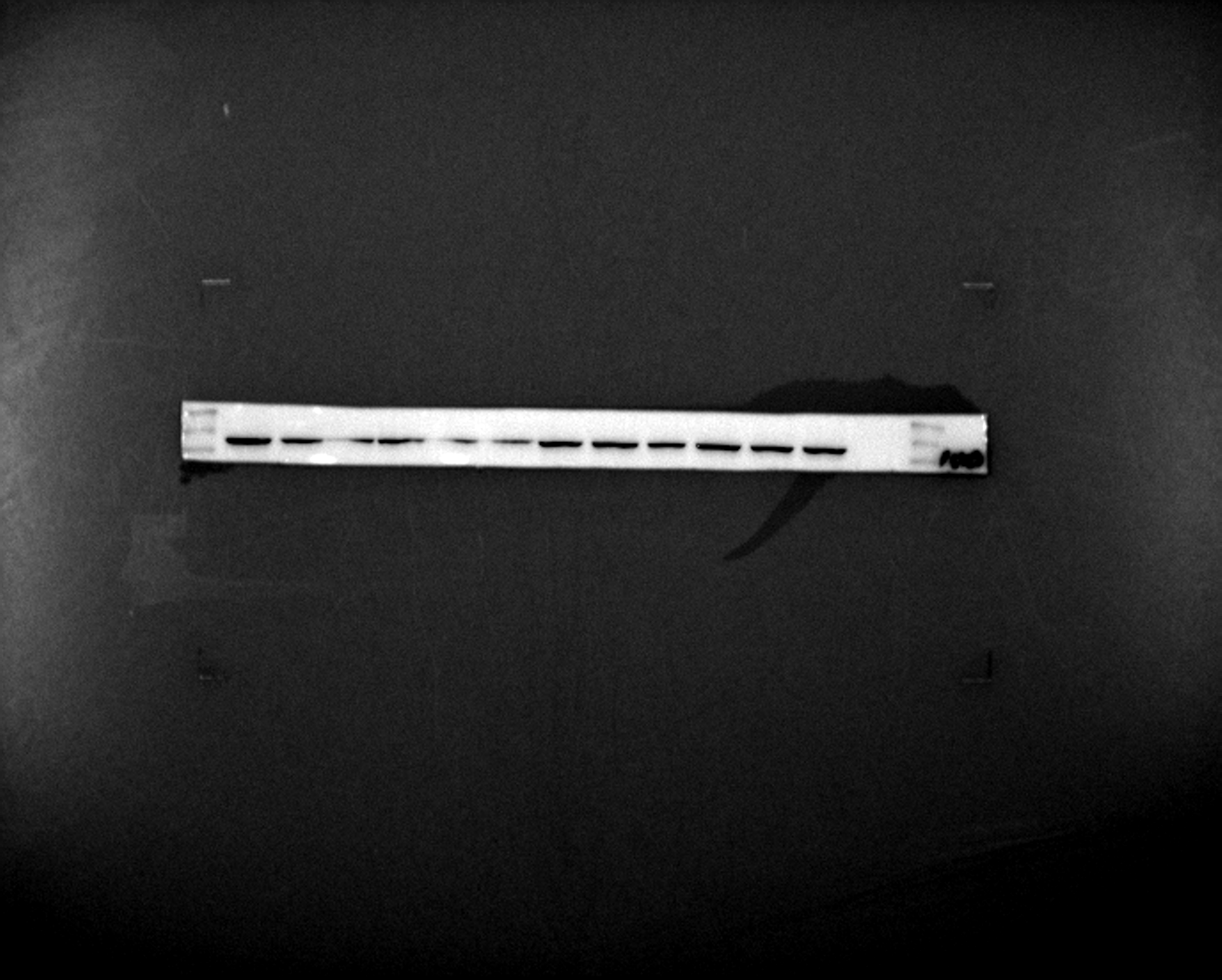

Supplement: Figure 5—source data 6. [file elife-96988-fig5-data6.zip › Figure 5-source data 6/SND1 expression in Snhg3-HKI mice/SND1.Tif]

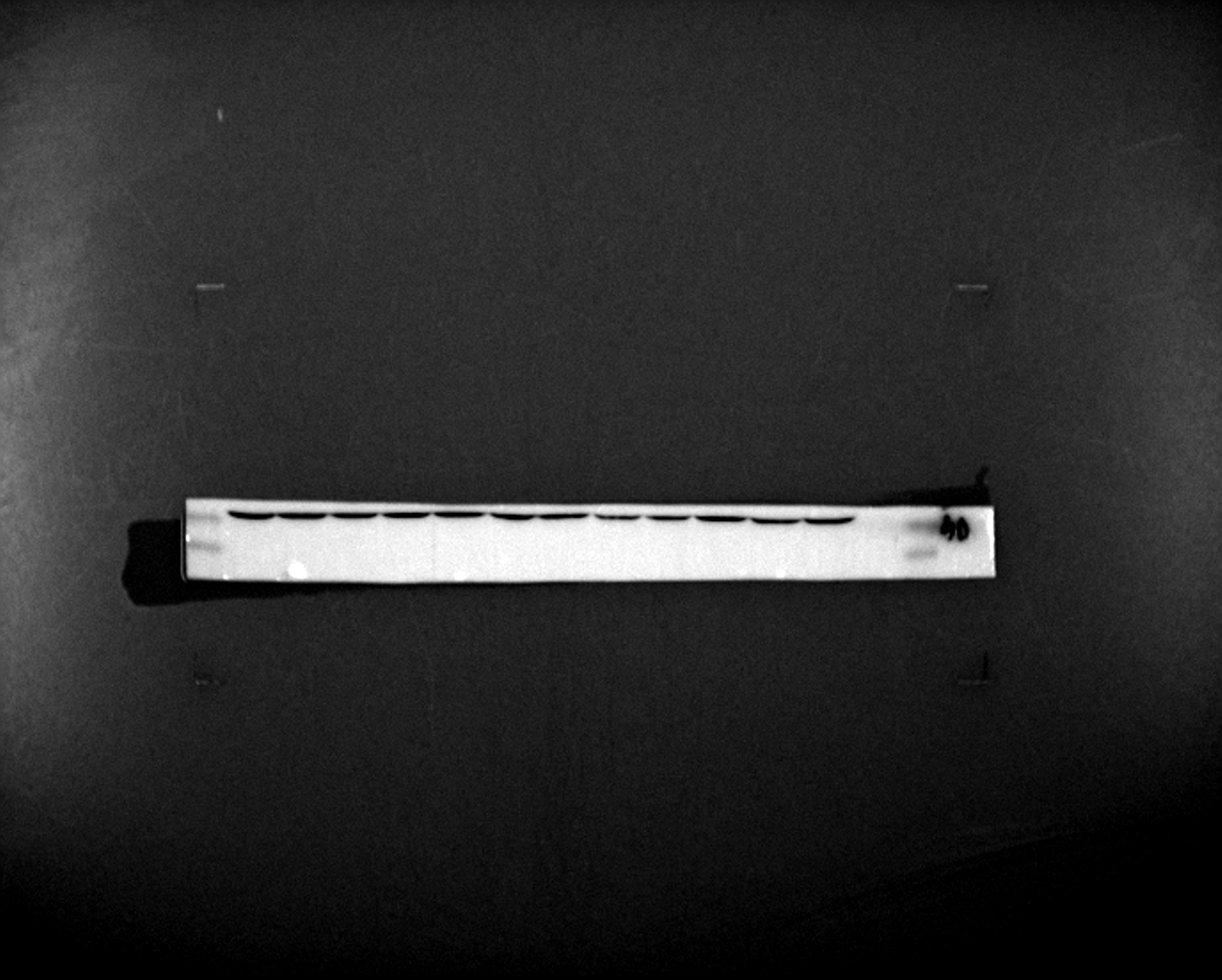

Supplement: Figure 5—source data 6. [file elife-96988-fig5-data6.zip › Figure 5-source data 6/SND1 expression in Snhg3-HKI mice/β-ACTIN.Tif]

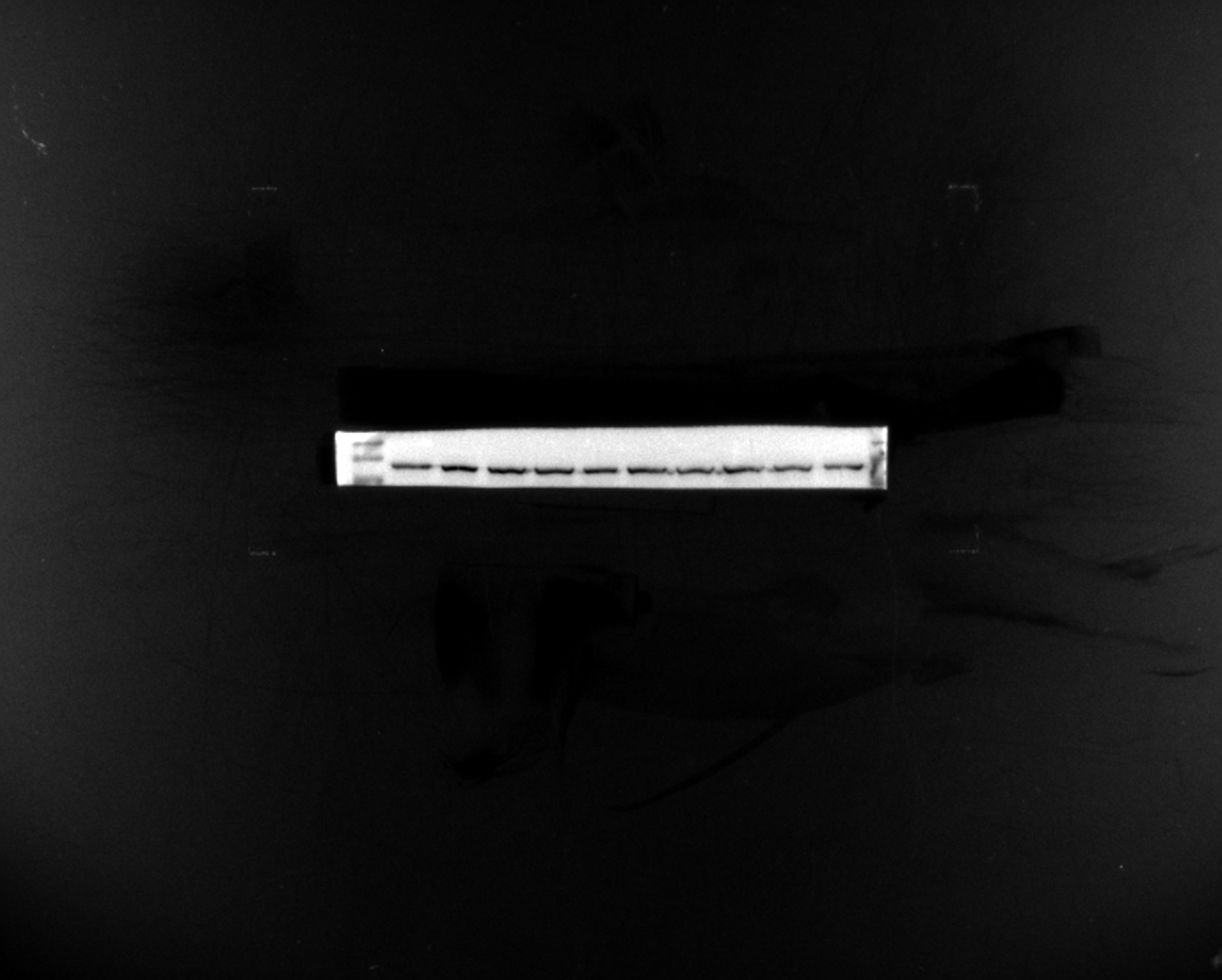

Supplement: Figure 5—source data 6. [file elife-96988-fig5-data6.zip › Figure 5-source data 6/SND1 expression Snhg3-HKO mice/SND1.tif]

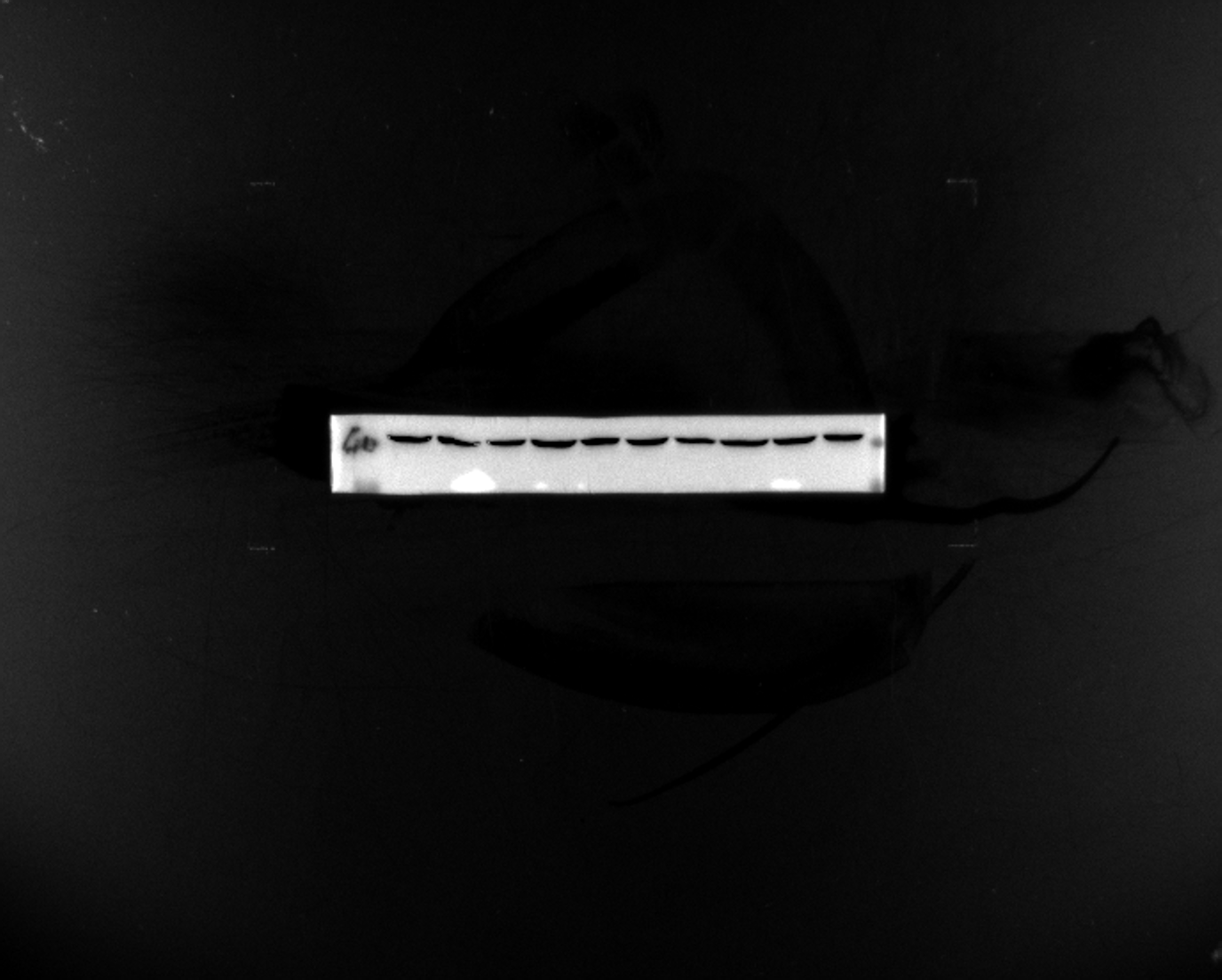

Supplement: Figure 5—source data 6. [file elife-96988-fig5-data6.zip › Figure 5-source data 6/SND1 expression Snhg3-HKO mice/β-ACTIN.tif]

# Figure 5I

BSA

1

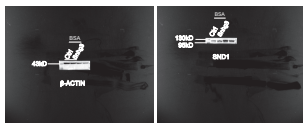

2

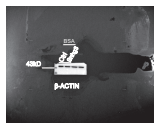

3

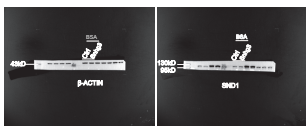

4

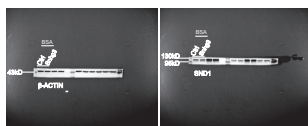

PA

1

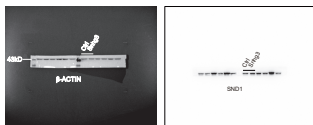

2

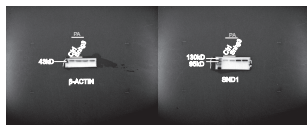

3

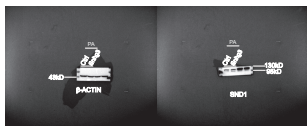

4

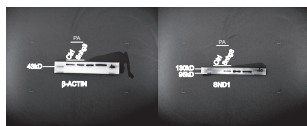

Supplement: Figure 5—source data 7. [file elife-96988-fig5-data7.pdf]

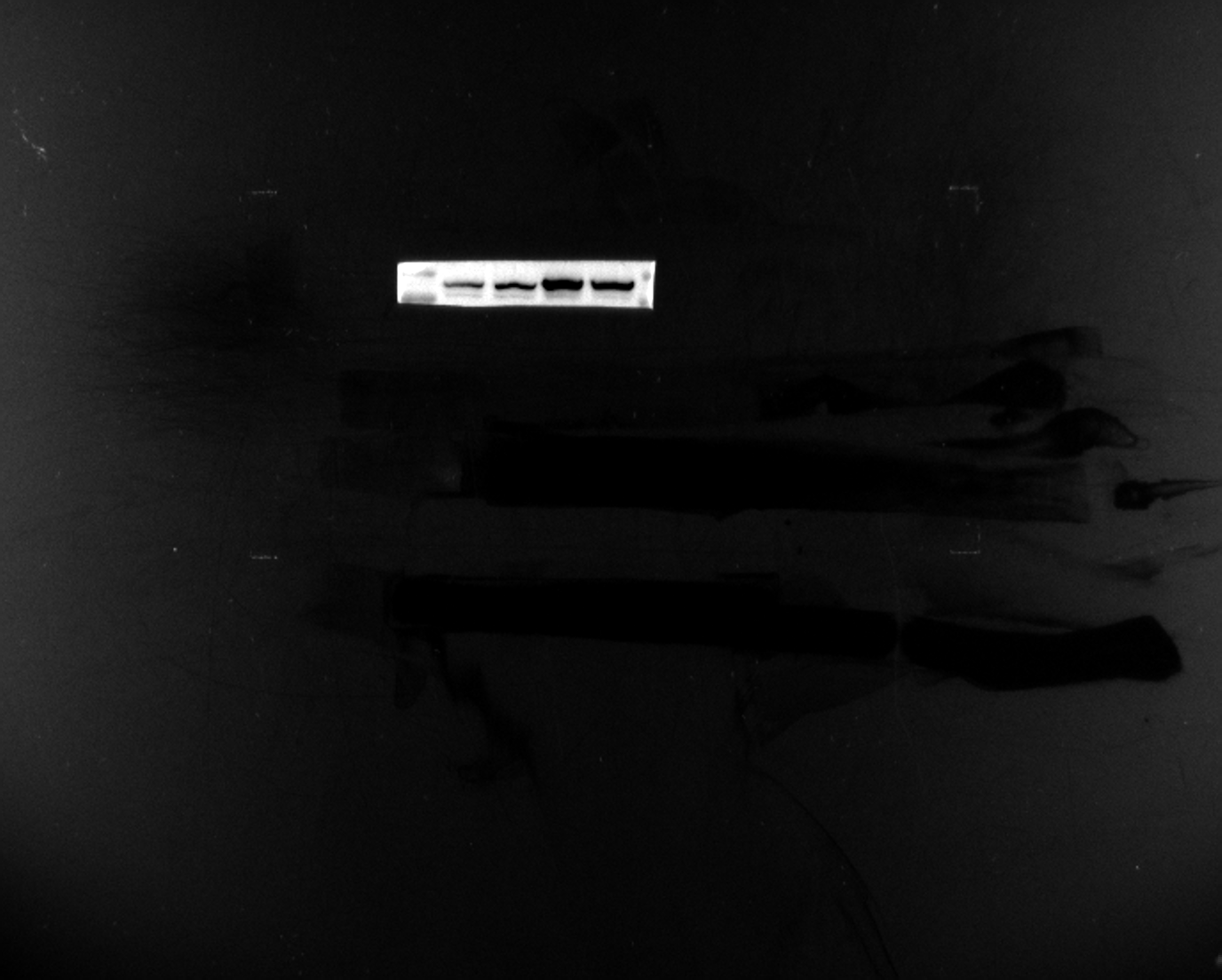

Supplement: Figure 5—source data 8. [file elife-96988-fig5-data8.zip › Figure 5-source data 8/BSA/1/SND1.tif]

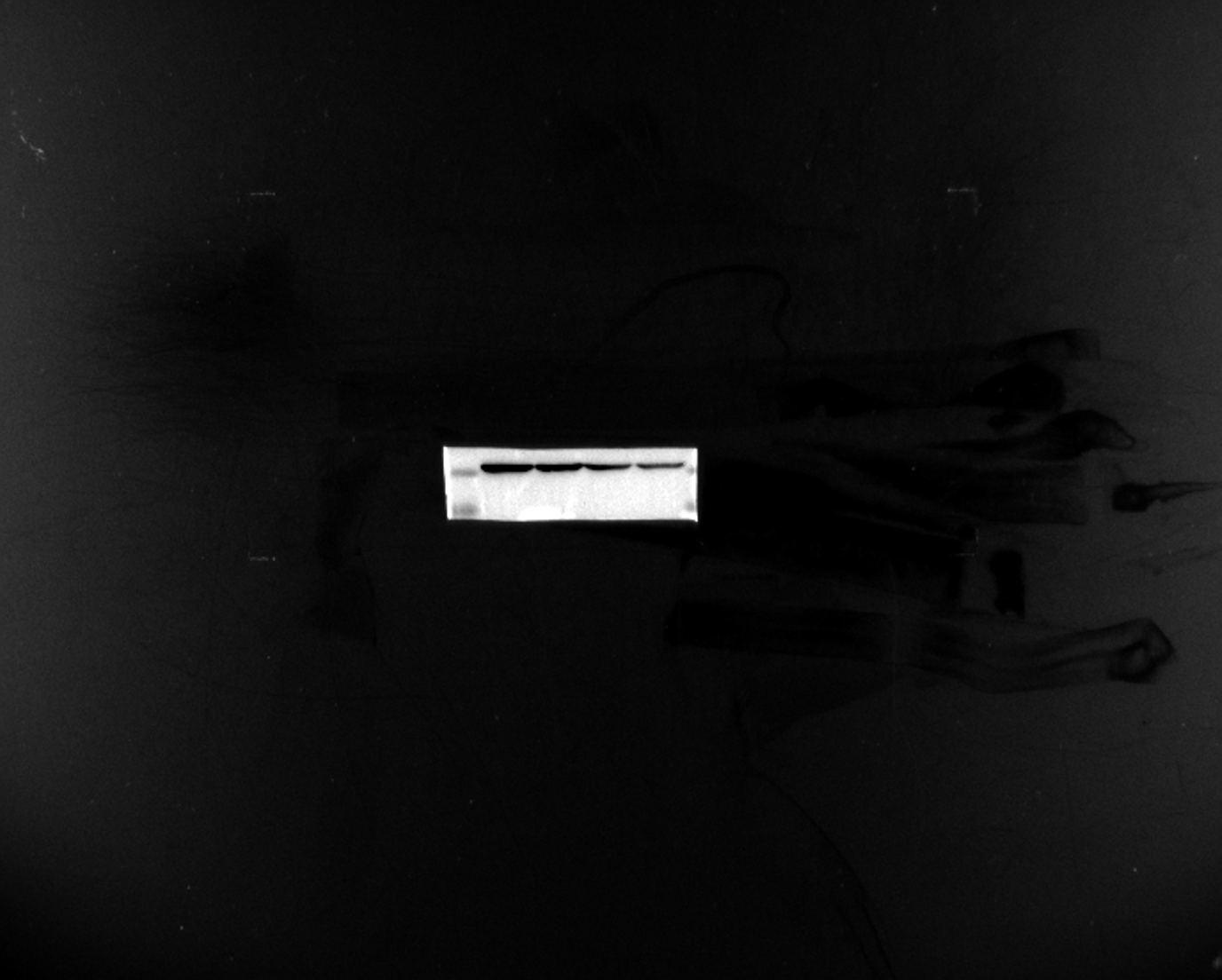

Supplement: Figure 5—source data 8. [file elife-96988-fig5-data8.zip › Figure 5-source data 8/BSA/1/β-ACTIN.tif]

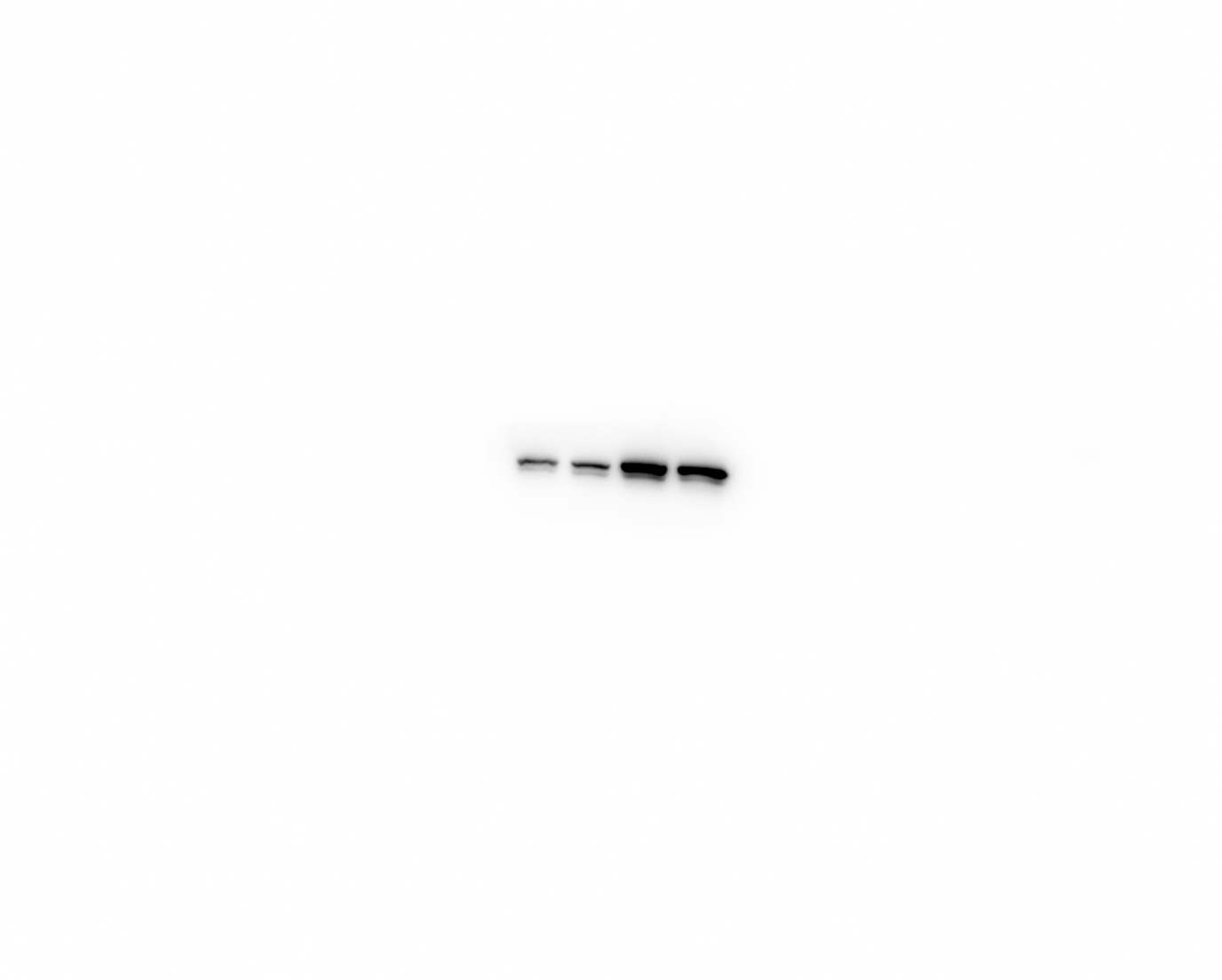

Supplement: Figure 5—source data 8. [file elife-96988-fig5-data8.zip › Figure 5-source data 8/BSA/2/SND1.Tif]

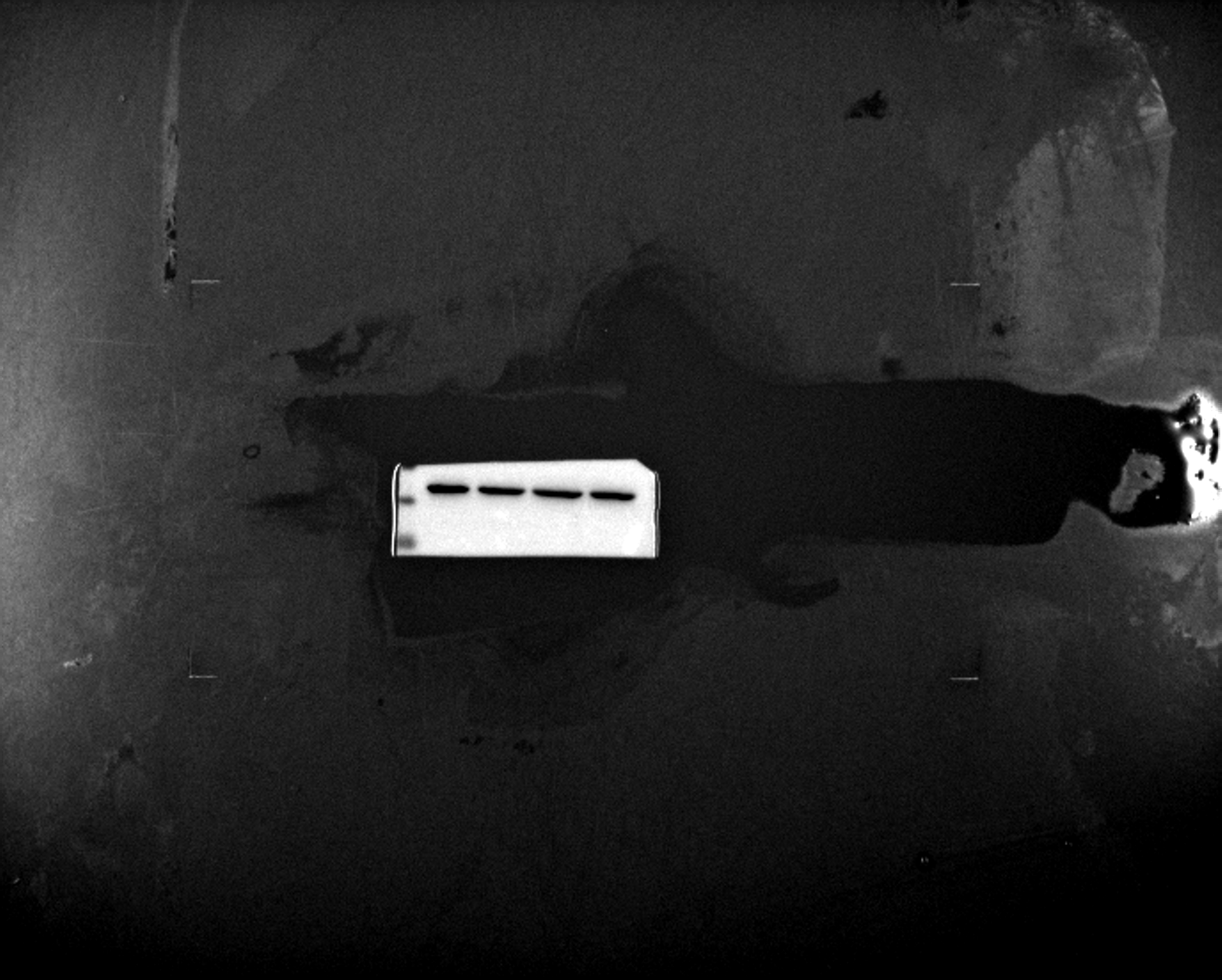

Supplement: Figure 5—source data 8. [file elife-96988-fig5-data8.zip › Figure 5-source data 8/BSA/2/β-ACTIN.Tif]

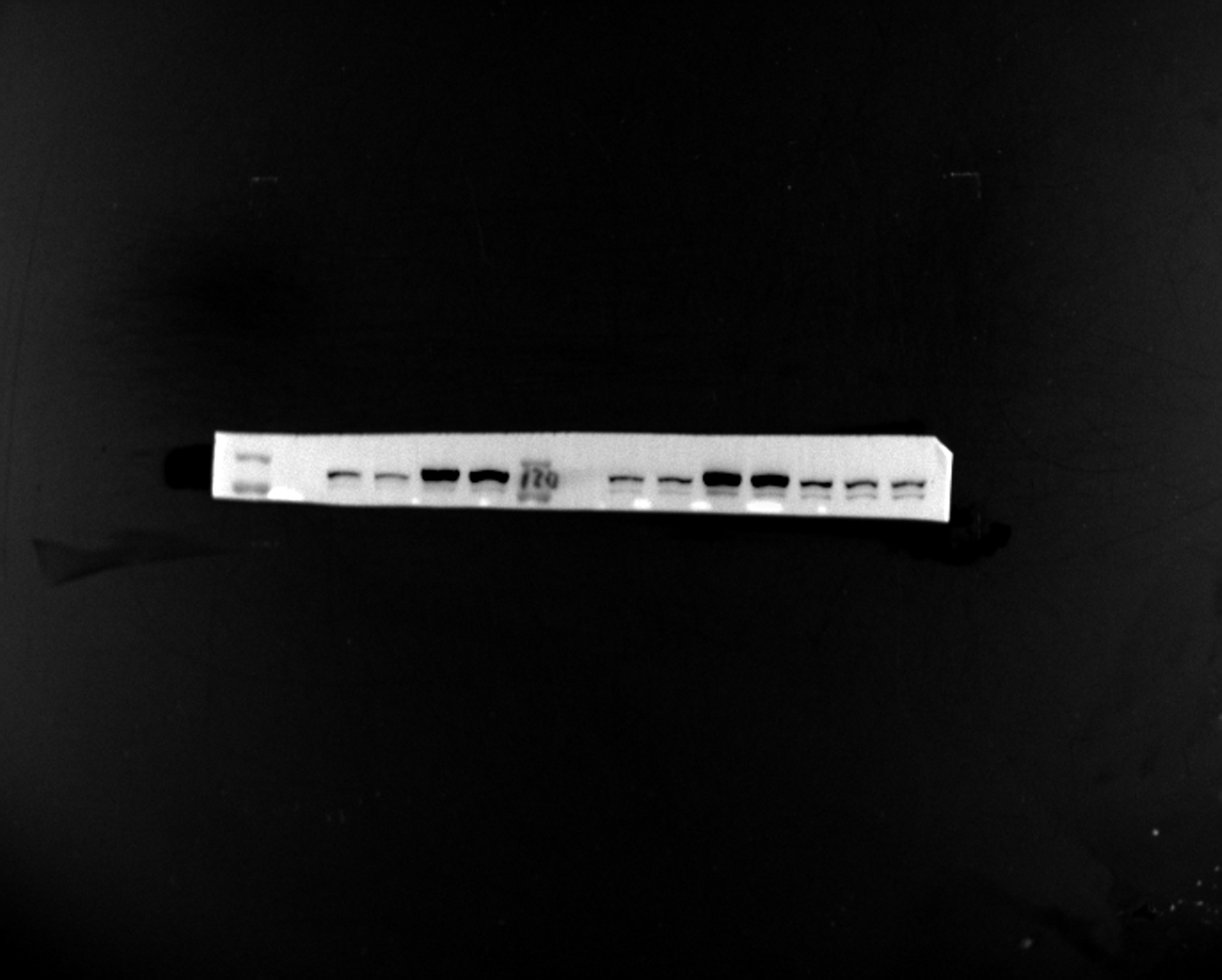

Supplement: Figure 5—source data 8. [file elife-96988-fig5-data8.zip › Figure 5-source data 8/BSA/3/SND1.tif]

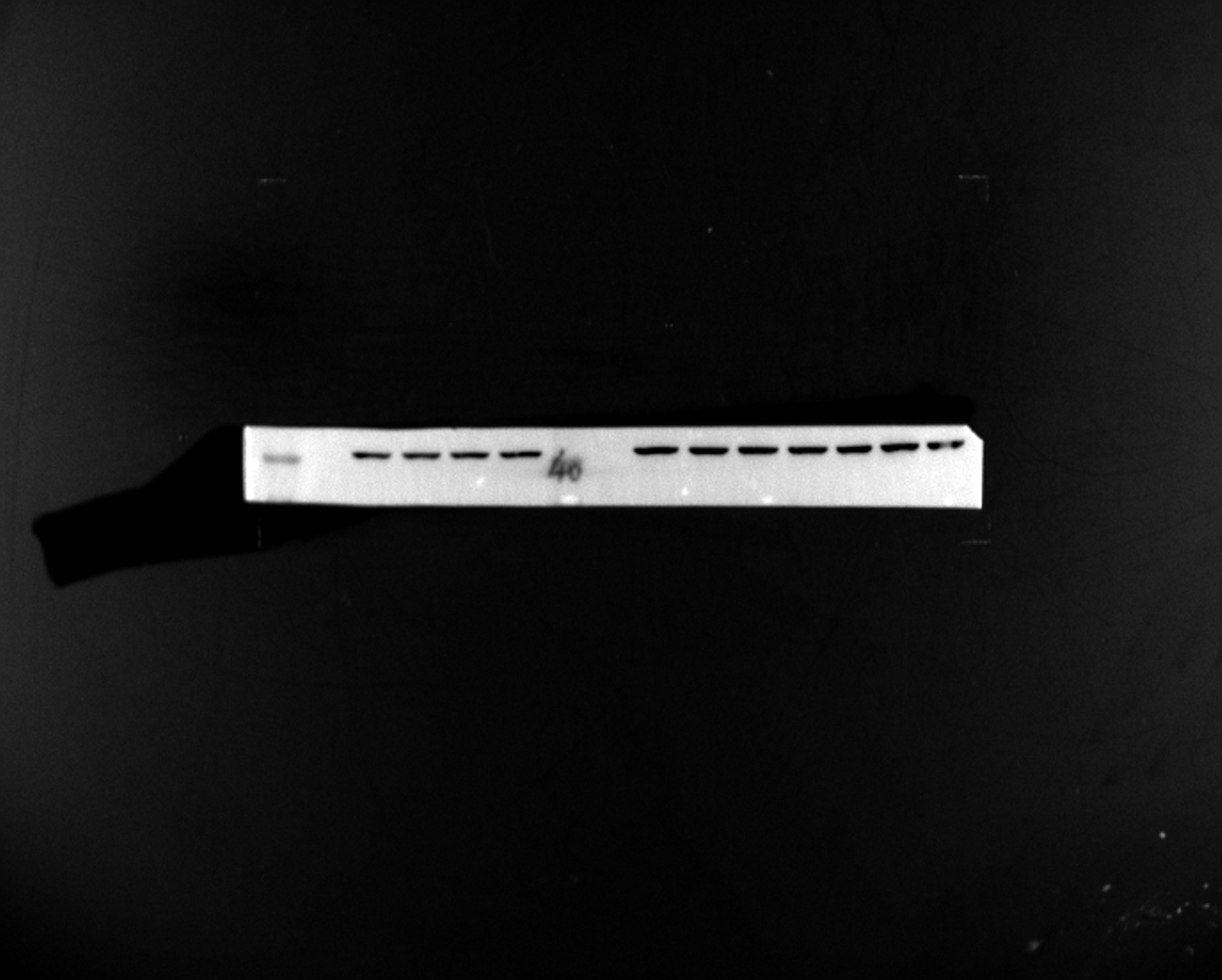

Supplement: Figure 5—source data 8. [file elife-96988-fig5-data8.zip › Figure 5-source data 8/BSA/3/β-ACTIN.tif]

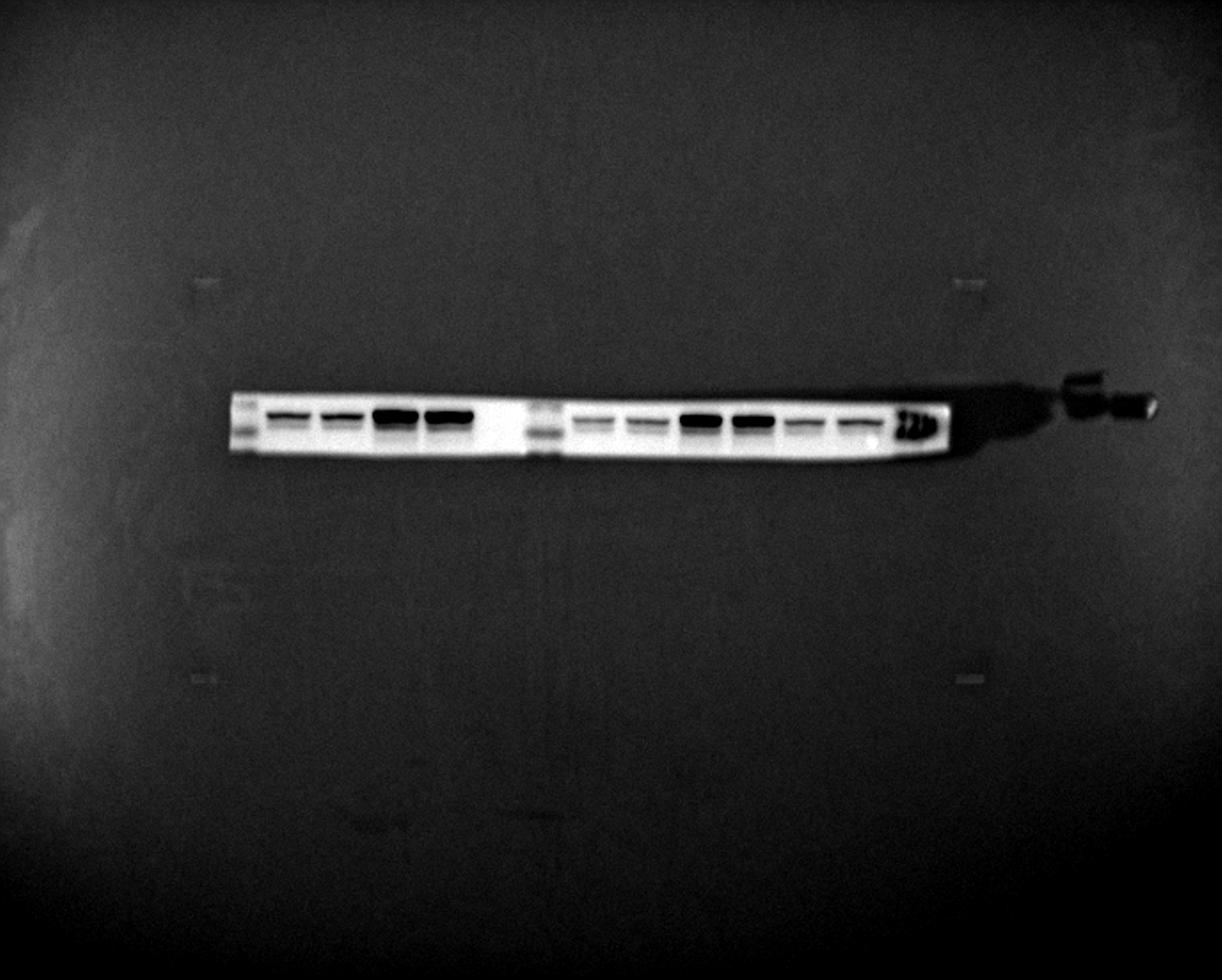

Supplement: Figure 5—source data 8. [file elife-96988-fig5-data8.zip › Figure 5-source data 8/BSA/4/SND1.Tif]

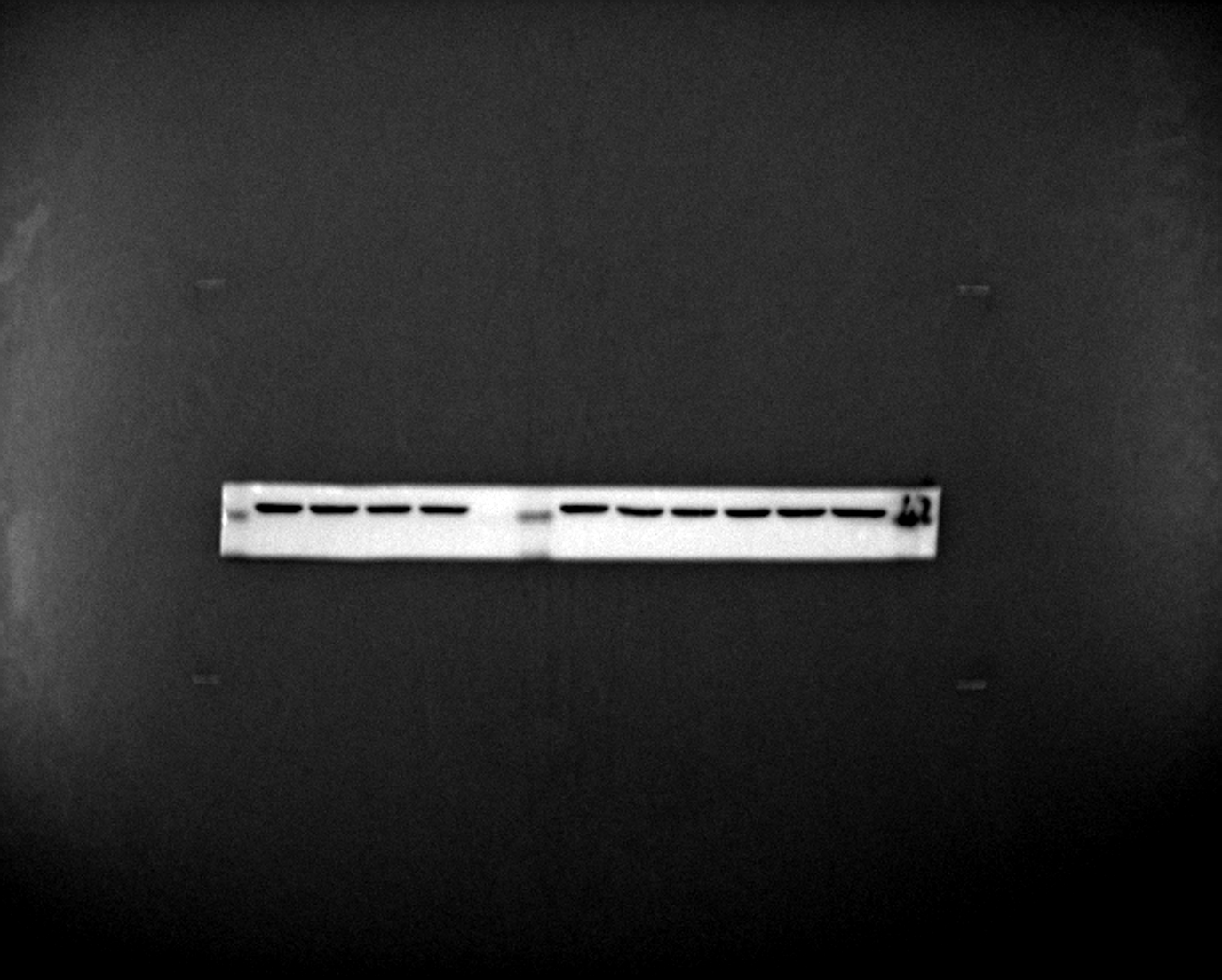

Supplement: Figure 5—source data 8. [file elife-96988-fig5-data8.zip › Figure 5-source data 8/BSA/4/β-ACTIN.Tif]

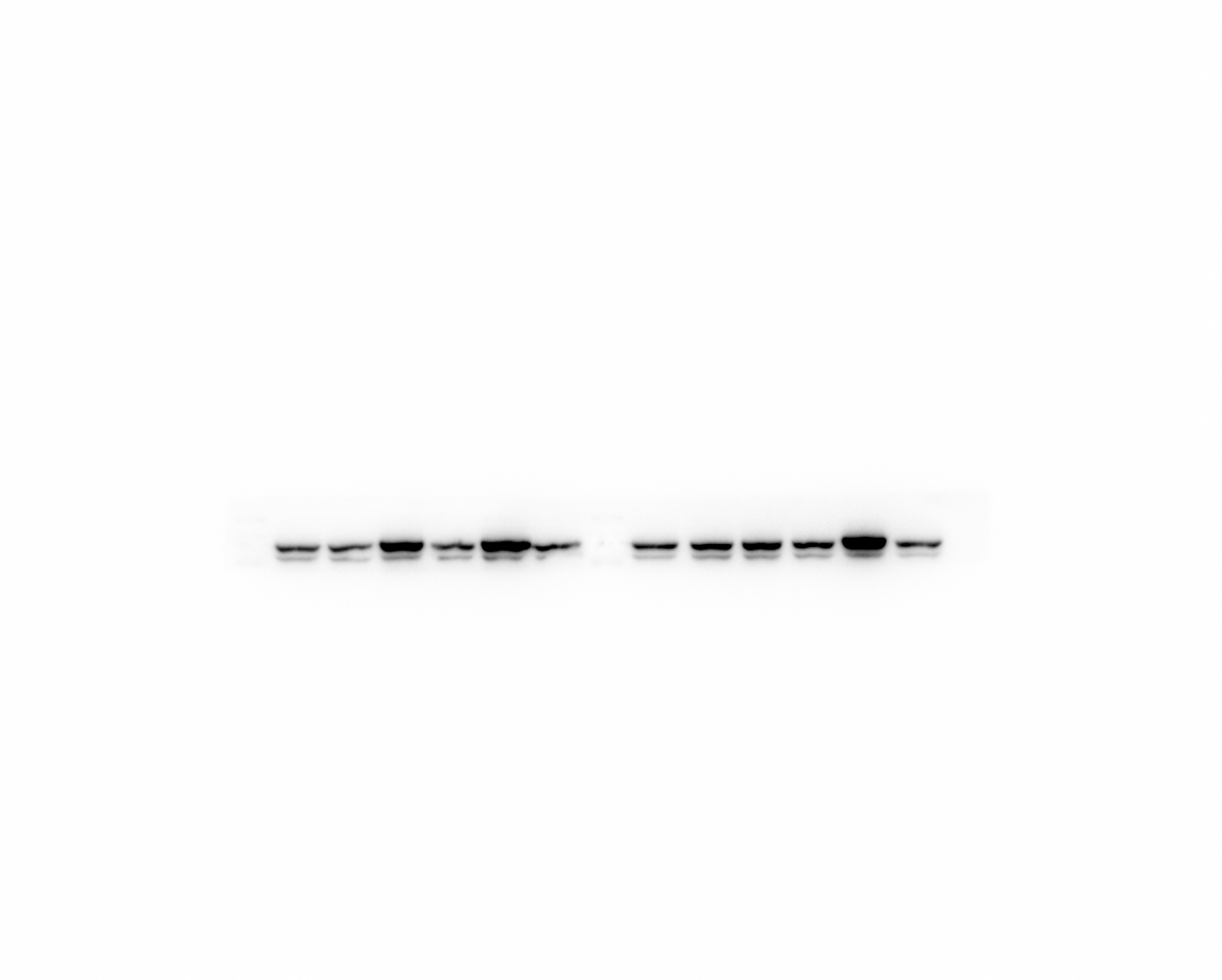

Supplement: Figure 5—source data 8. [file elife-96988-fig5-data8.zip › Figure 5-source data 8/PA/1/SND1.Tif]

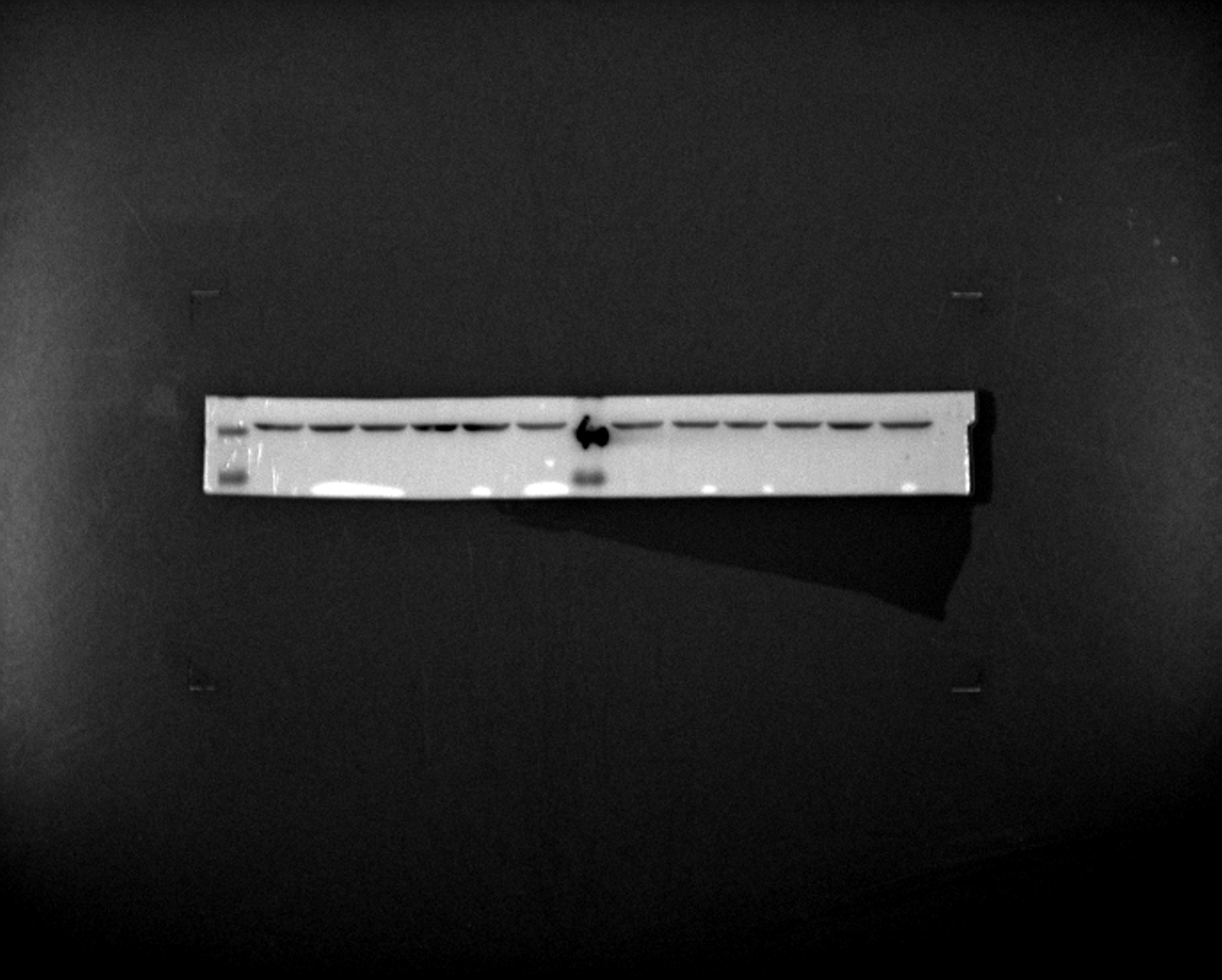

Supplement: Figure 5—source data 8. [file elife-96988-fig5-data8.zip › Figure 5-source data 8/PA/1/β-ACTIN.Tif]

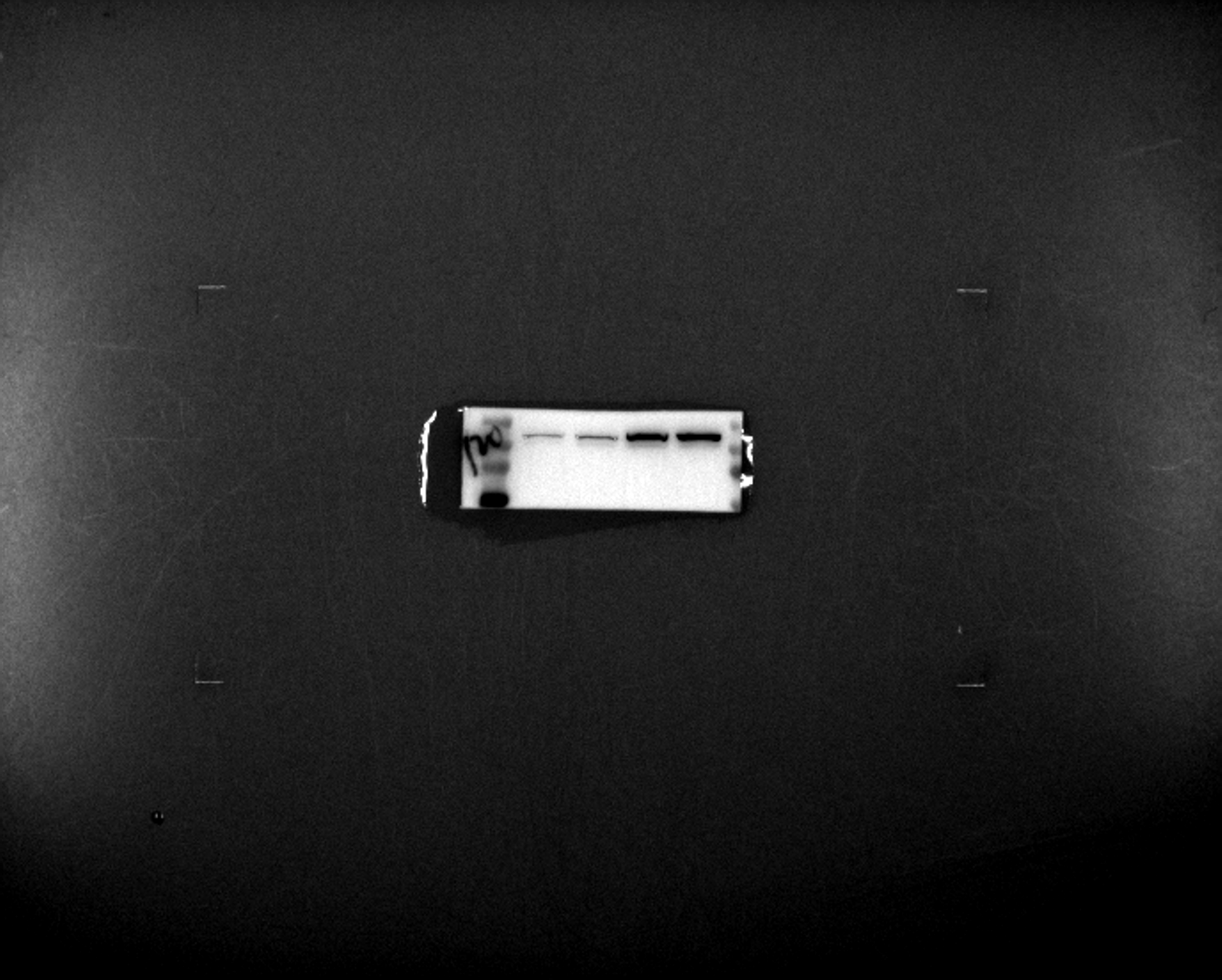

Supplement: Figure 5—source data 8. [file elife-96988-fig5-data8.zip › Figure 5-source data 8/PA/2/SND1.Tif]

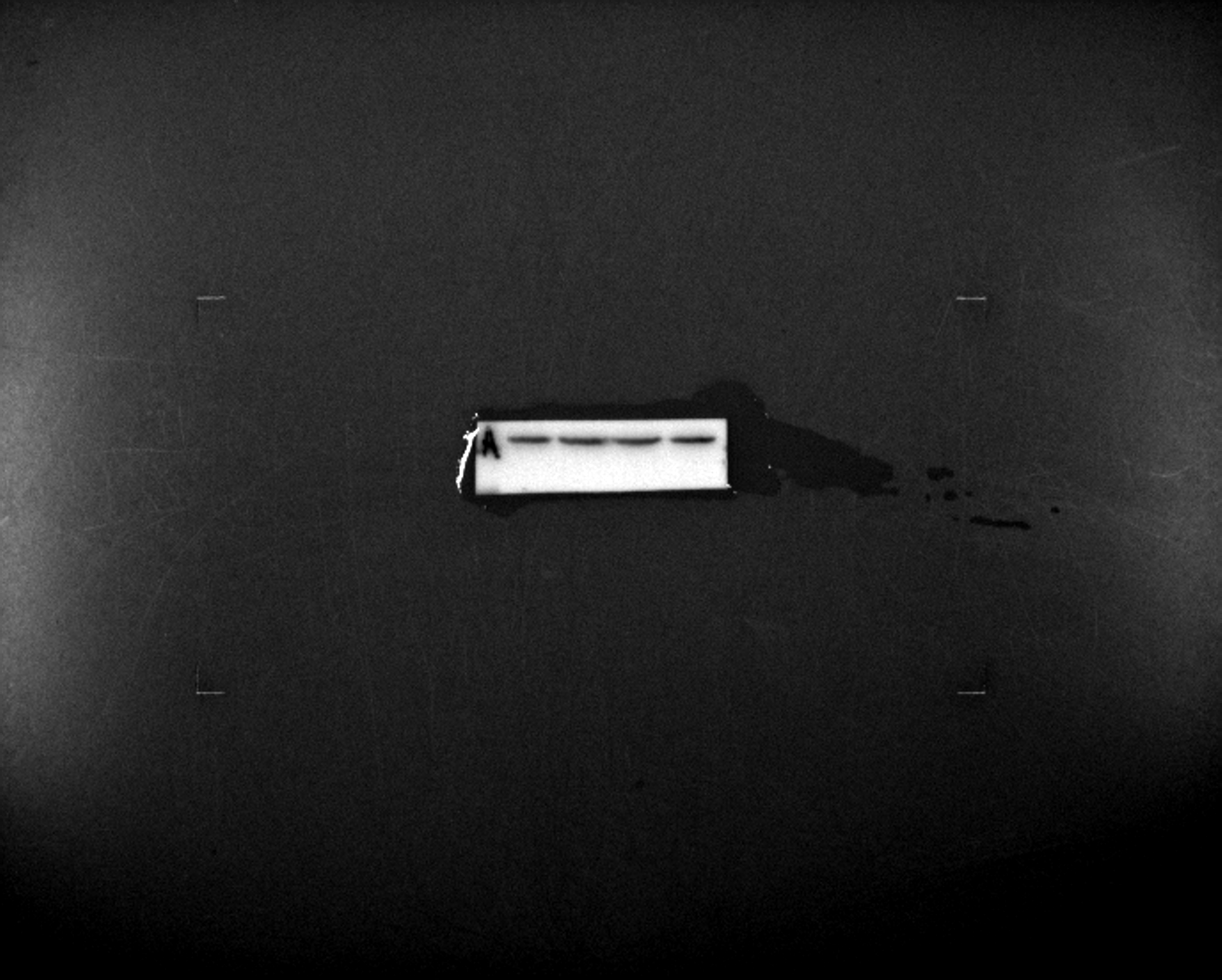

Supplement: Figure 5—source data 8. [file elife-96988-fig5-data8.zip › Figure 5-source data 8/PA/2/β-ACTIN.Tif]

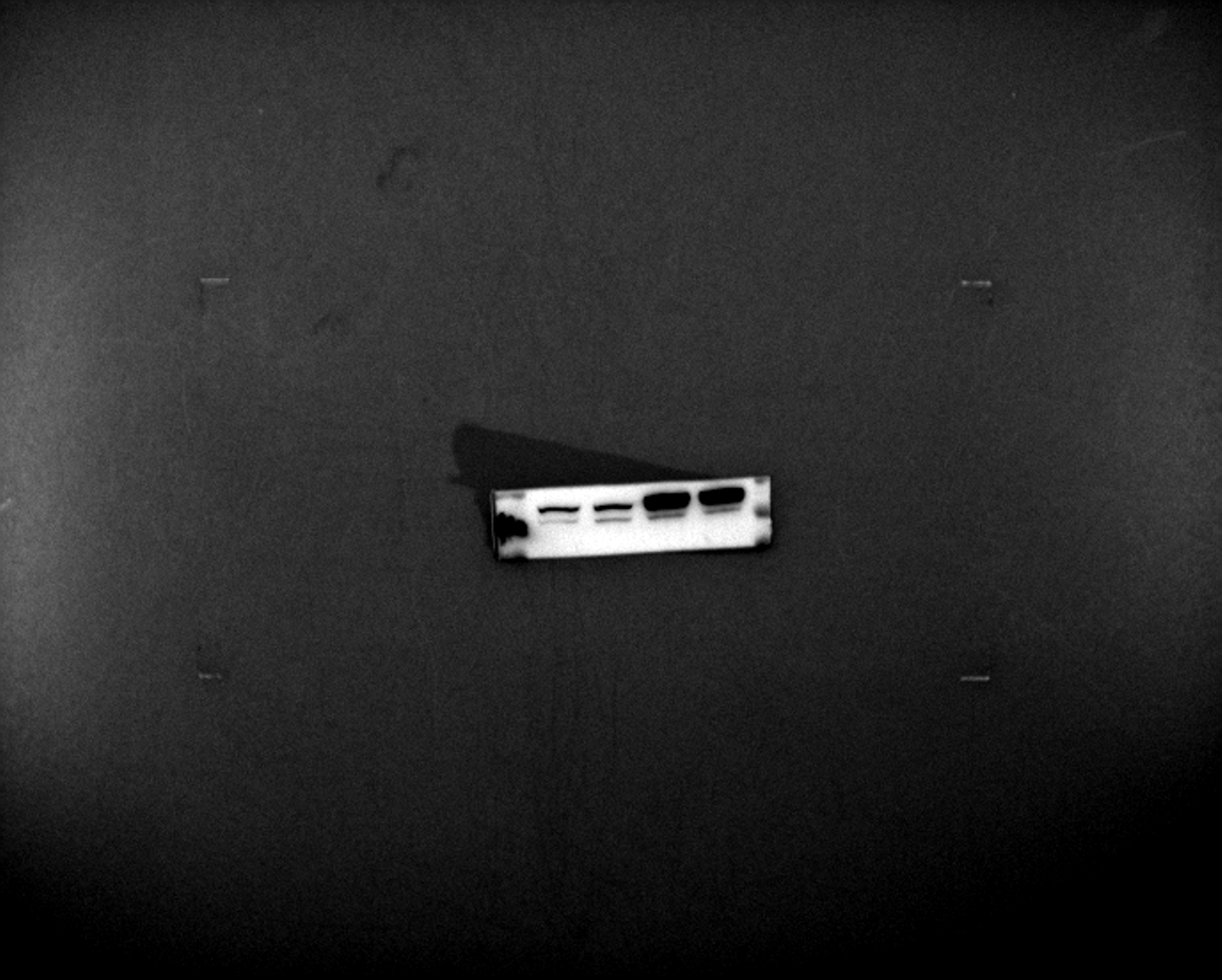

Supplement: Figure 5—source data 8. [file elife-96988-fig5-data8.zip › Figure 5-source data 8/PA/3/SND1.Tif]

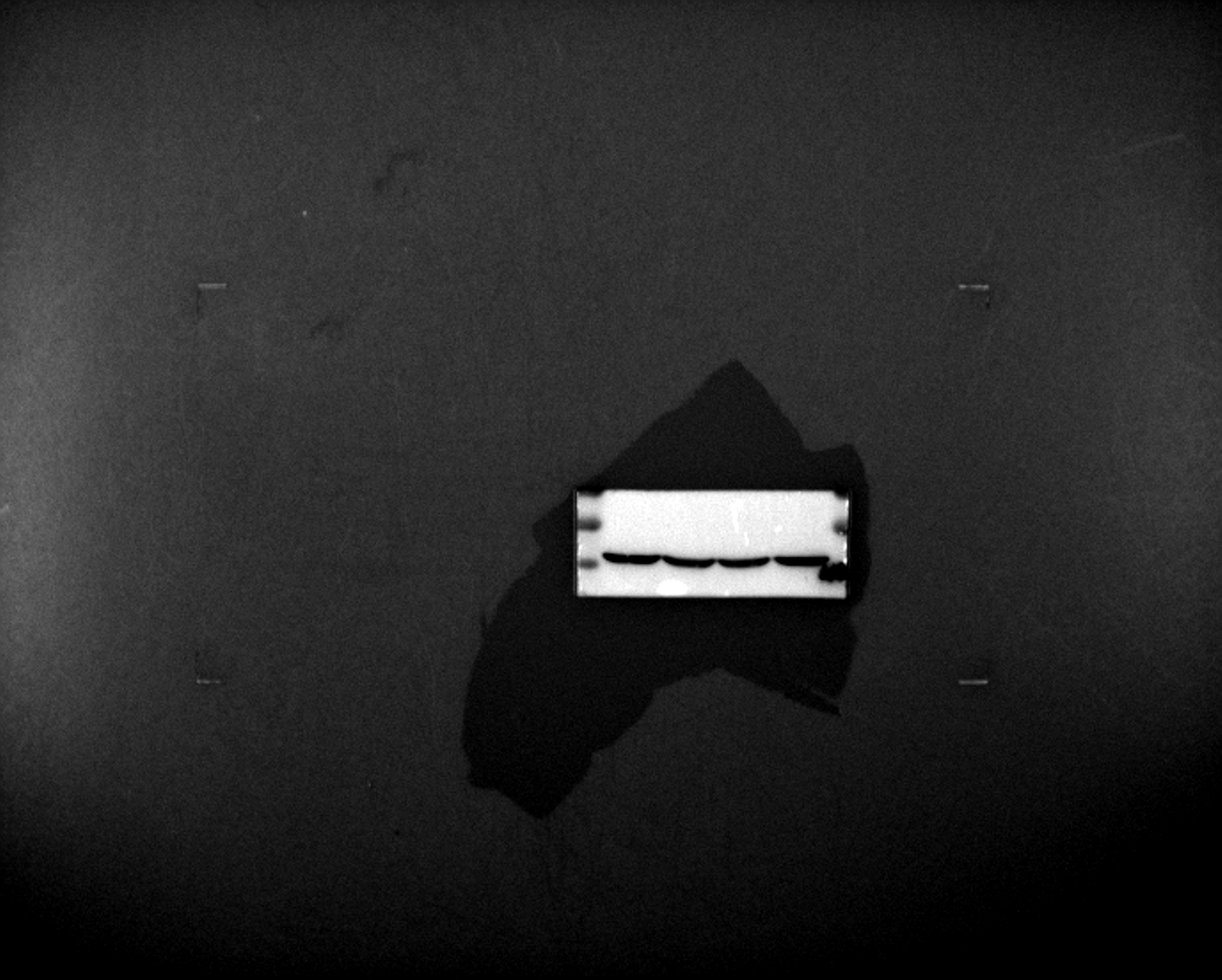

Supplement: Figure 5—source data 8. [file elife-96988-fig5-data8.zip › Figure 5-source data 8/PA/3/β-ACTIN.Tif]

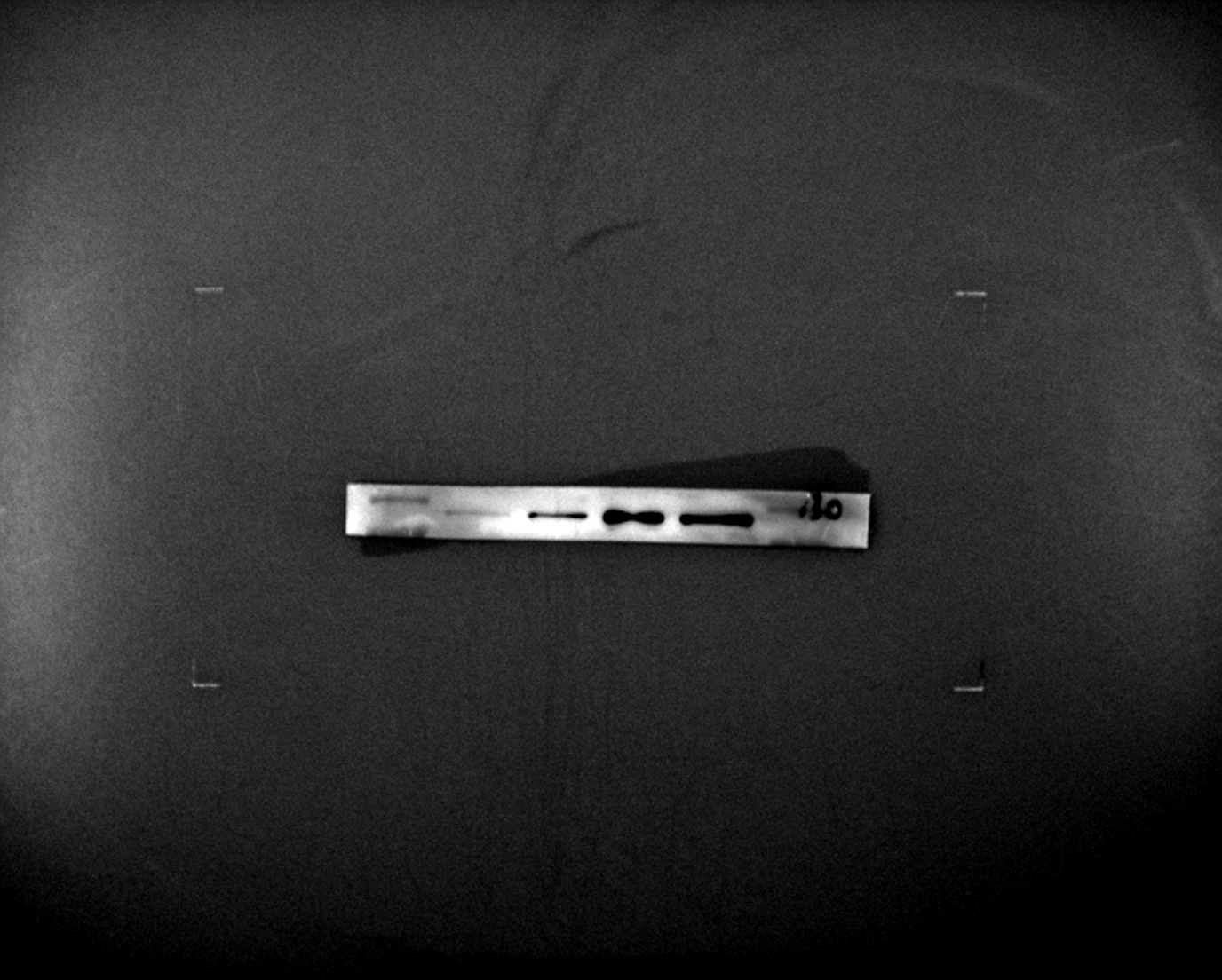

Supplement: Figure 5—source data 8. [file elife-96988-fig5-data8.zip › Figure 5-source data 8/PA/4/SND1.Tif]

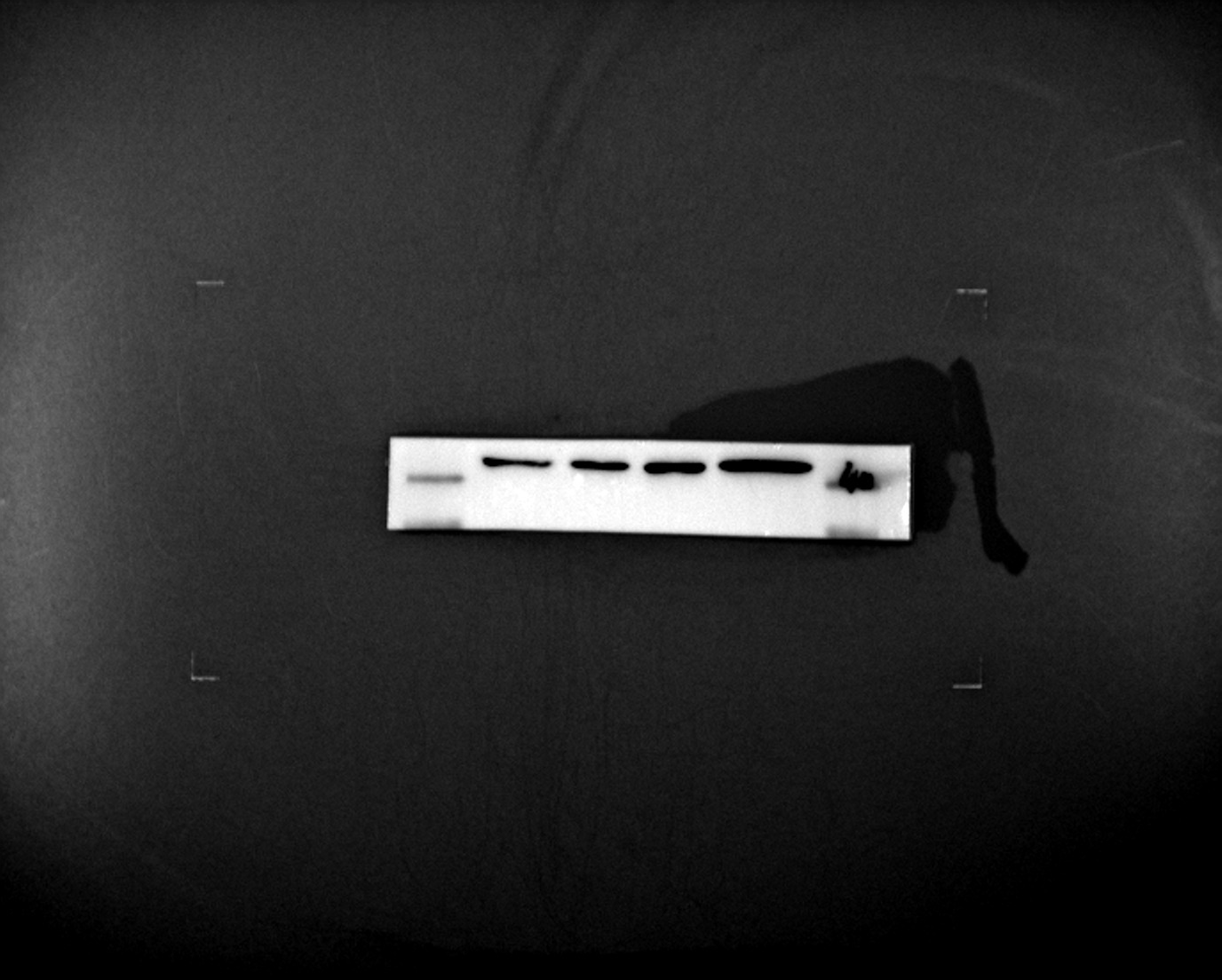

Supplement: Figure 5—source data 8. [file elife-96988-fig5-data8.zip › Figure 5-source data 8/PA/4/β-ACTIN.Tif]

Figure 5J

1.

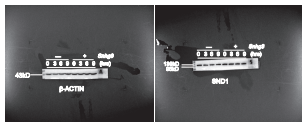

2.

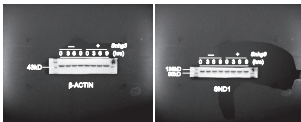

3.

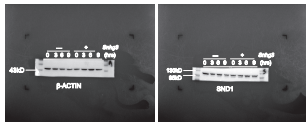

4.

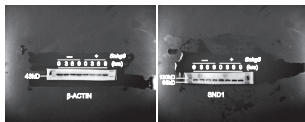

Supplement: Figure 5—source data 9. [file elife-96988-fig5-data9.pdf]

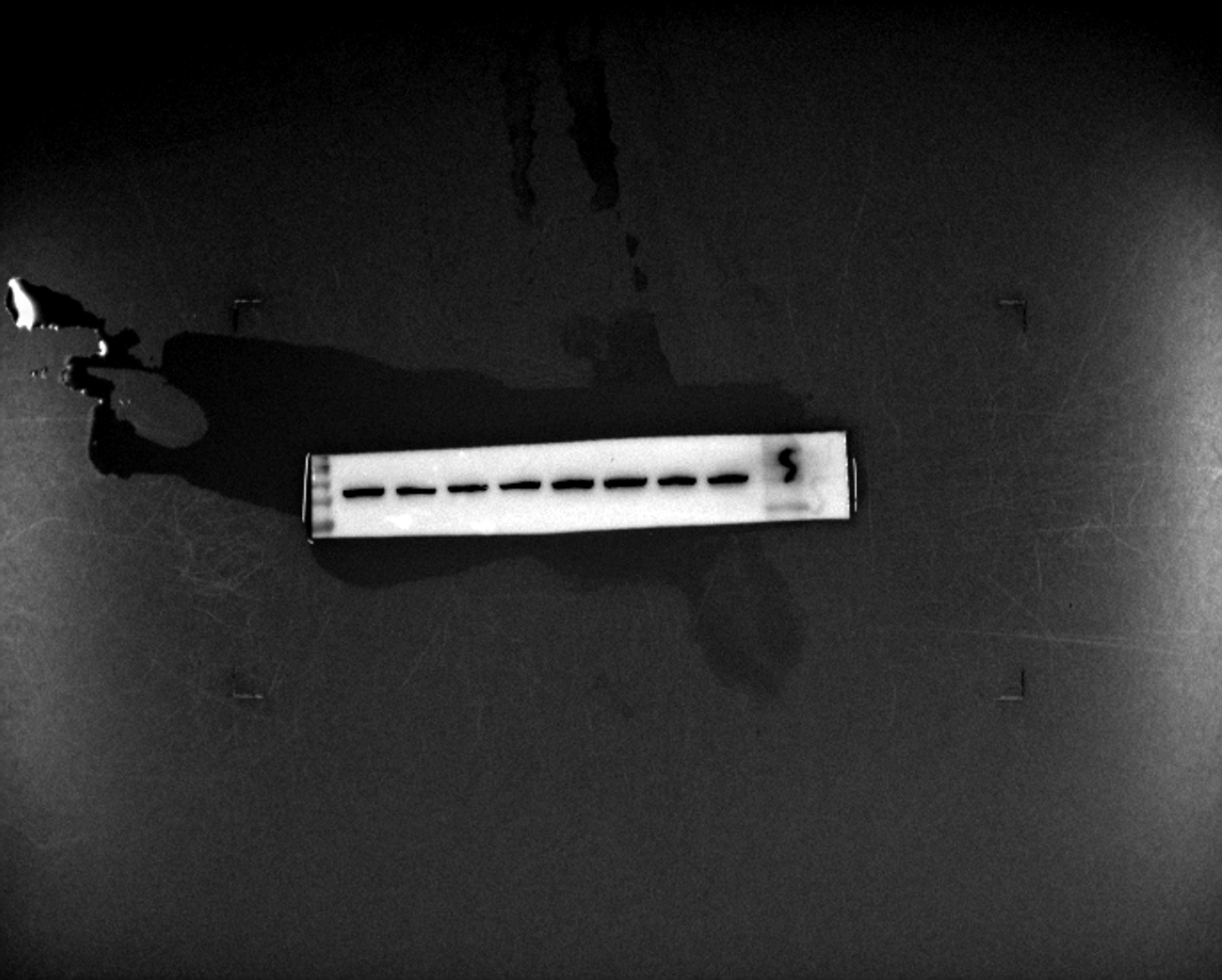

Supplement: Figure 5—source data 10. [file elife-96988-fig5-data10.zip › Figure 5-source data 10/1/SND1.Tif]

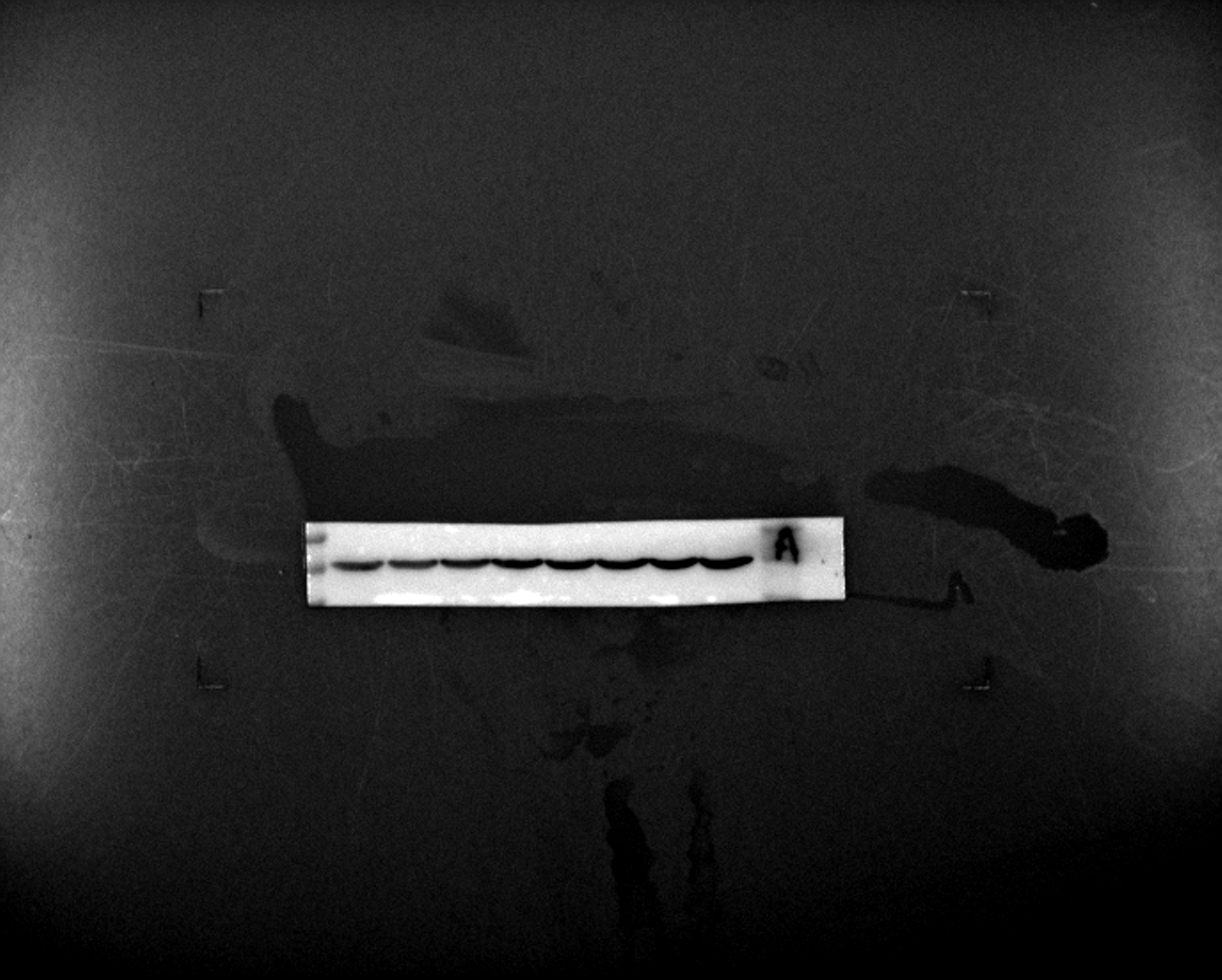

Supplement: Figure 5—source data 10. [file elife-96988-fig5-data10.zip › Figure 5-source data 10/1/β-ACTIN.Tif]

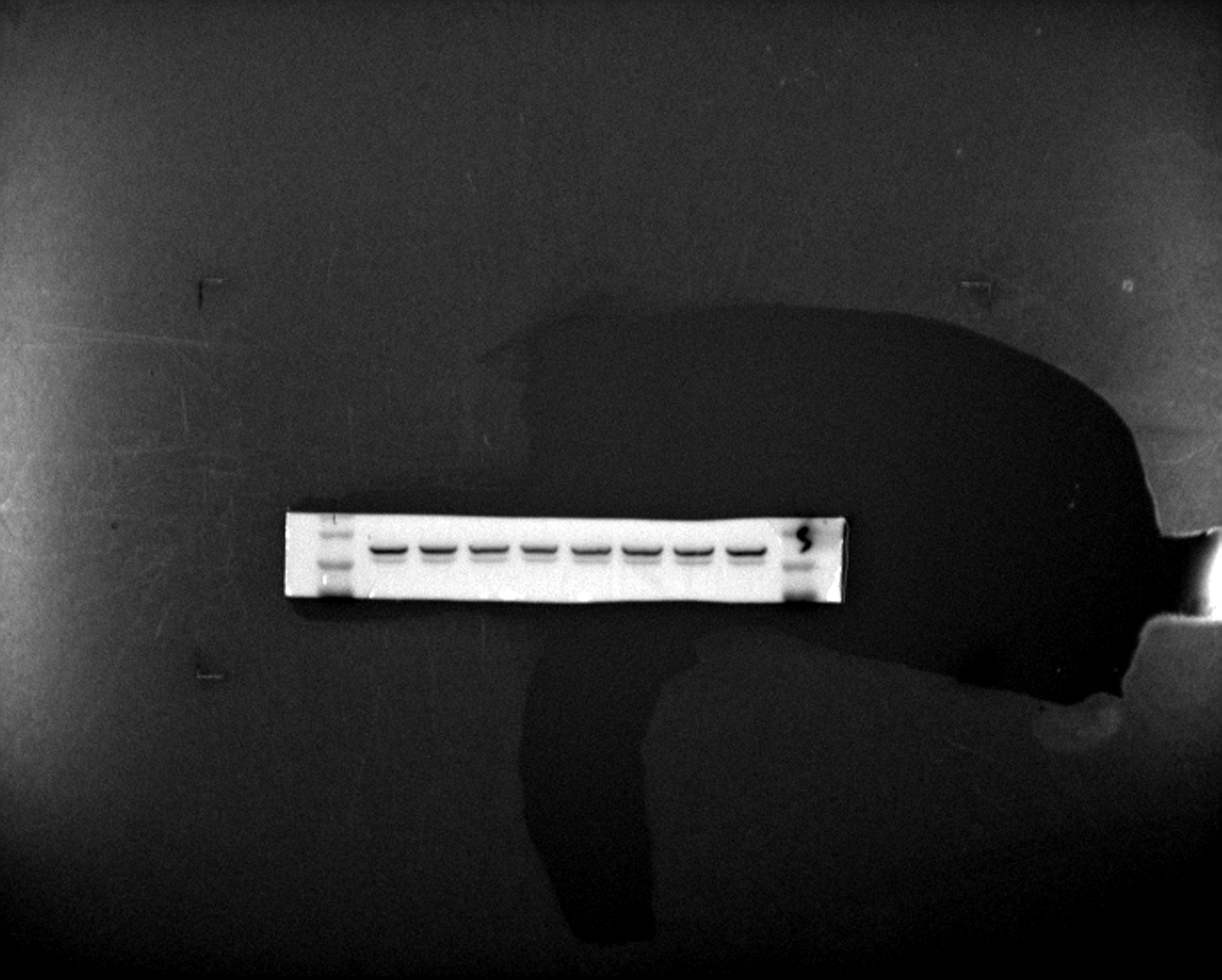

Supplement: Figure 5—source data 10. [file elife-96988-fig5-data10.zip › Figure 5-source data 10/2/SND1.Tif]

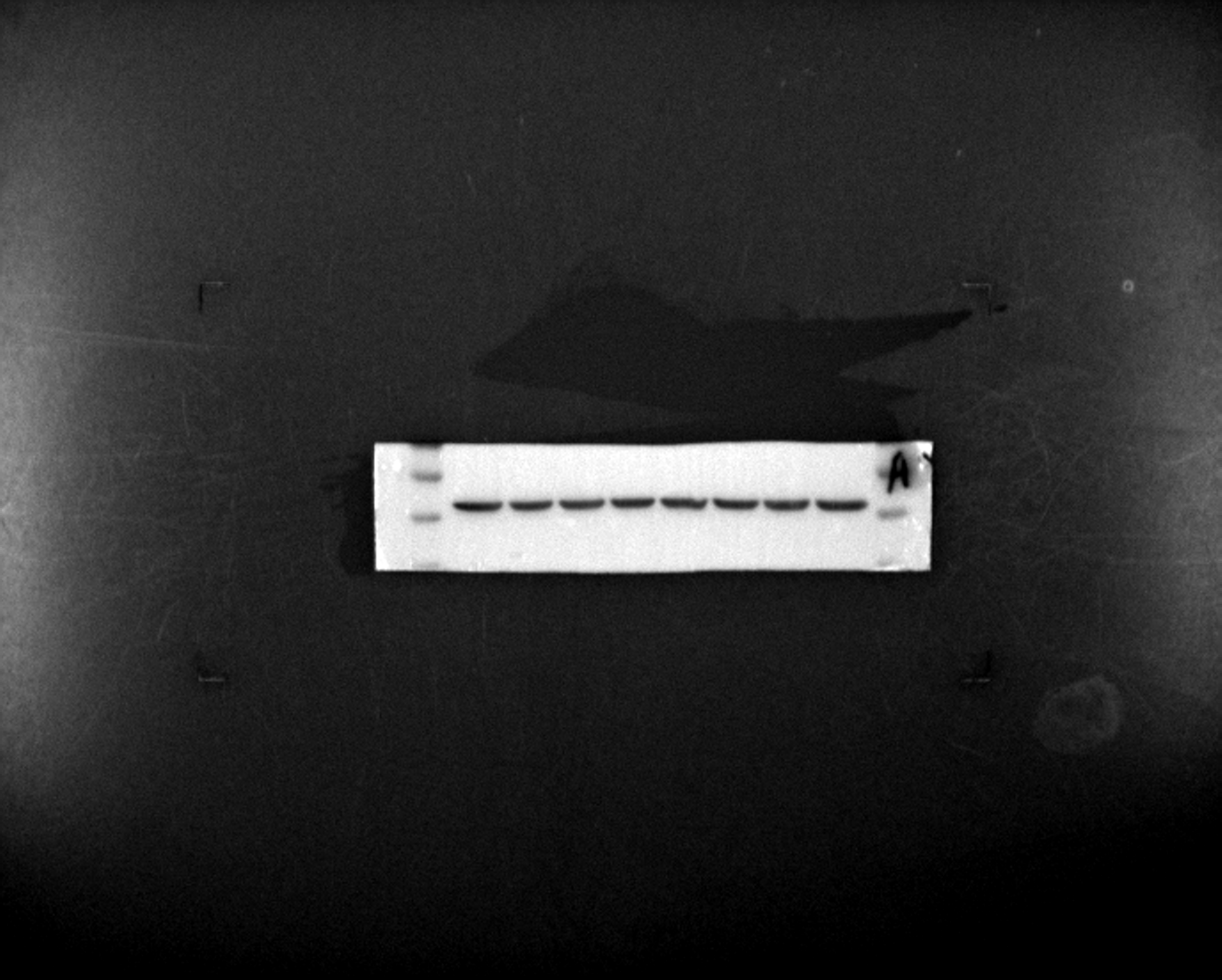

Supplement: Figure 5—source data 10. [file elife-96988-fig5-data10.zip › Figure 5-source data 10/2/β-ACTIN.Tif]

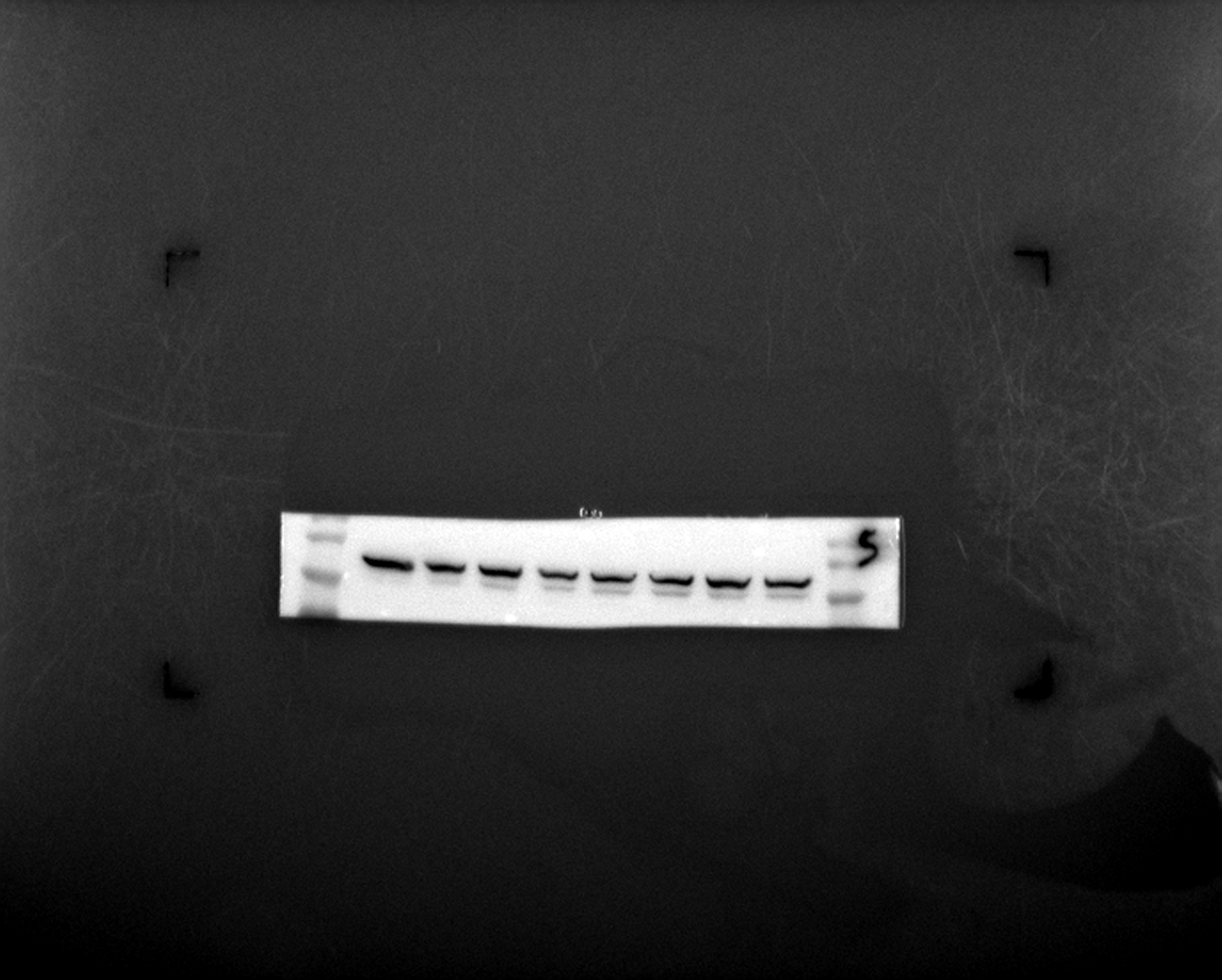

Supplement: Figure 5—source data 10. [file elife-96988-fig5-data10.zip › Figure 5-source data 10/3/SND1.tif]

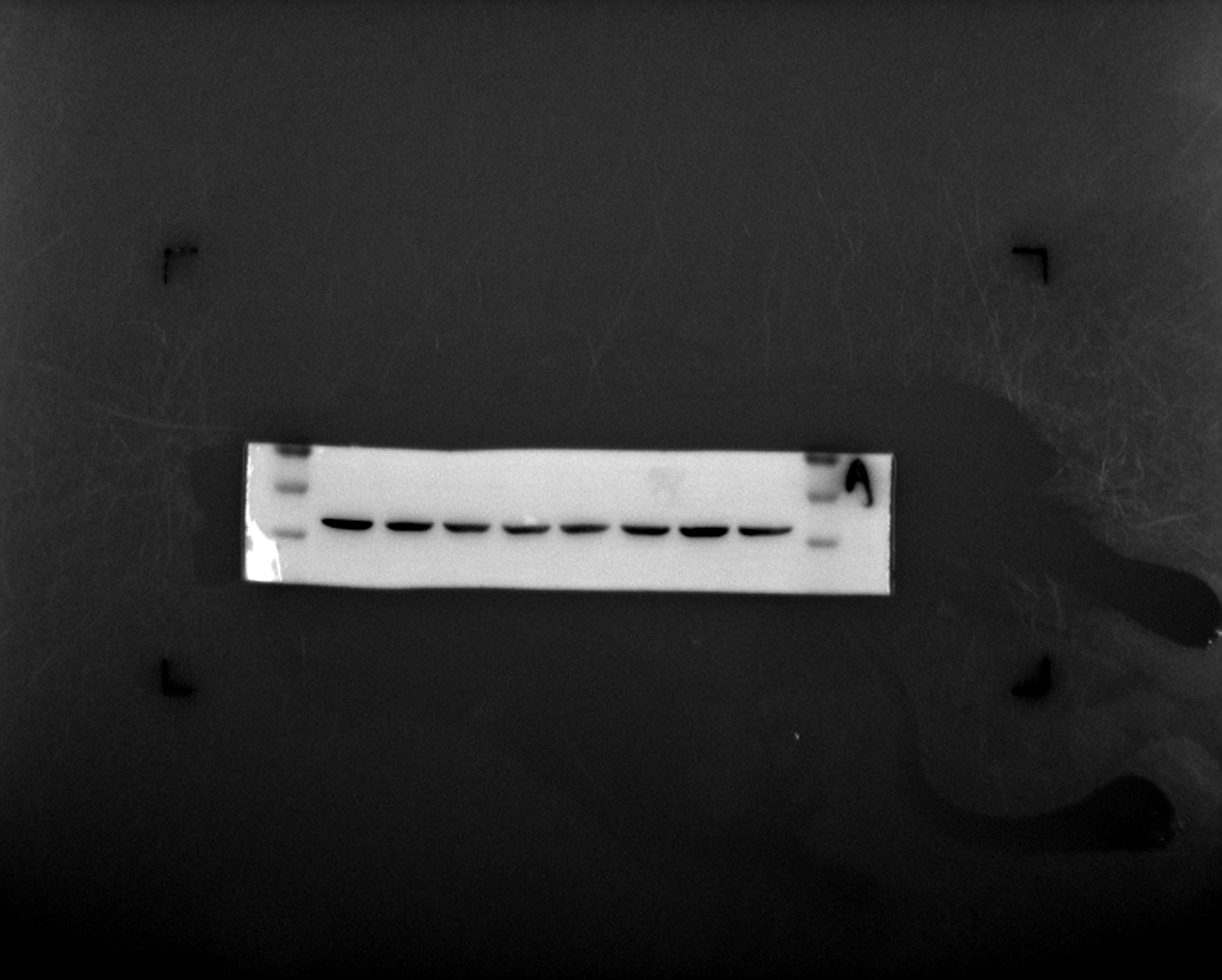

Supplement: Figure 5—source data 10. [file elife-96988-fig5-data10.zip › Figure 5-source data 10/3/β-ACTIN.tif]

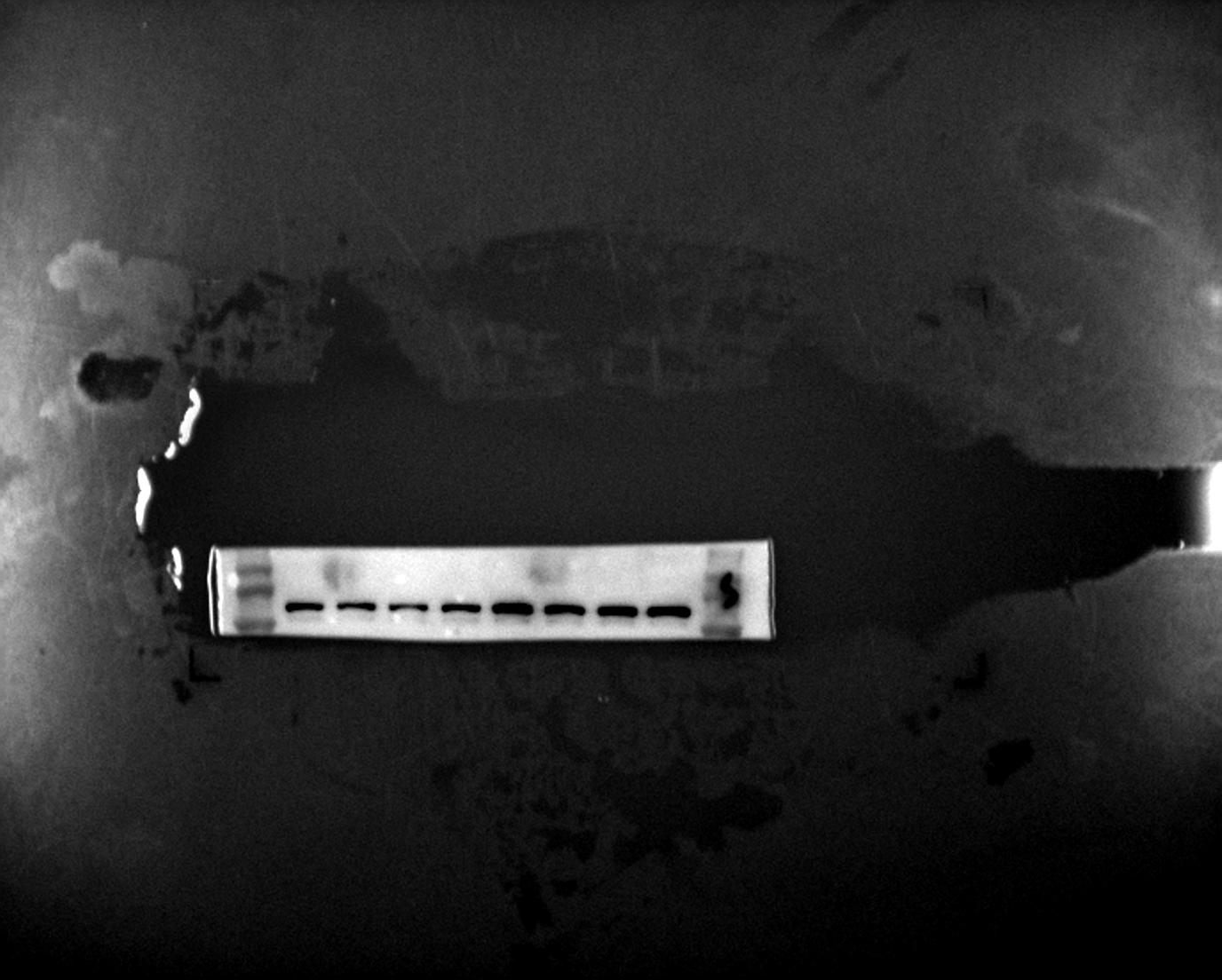

Supplement: Figure 5—source data 10. [file elife-96988-fig5-data10.zip › Figure 5-source data 10/4/SND1.Tif]

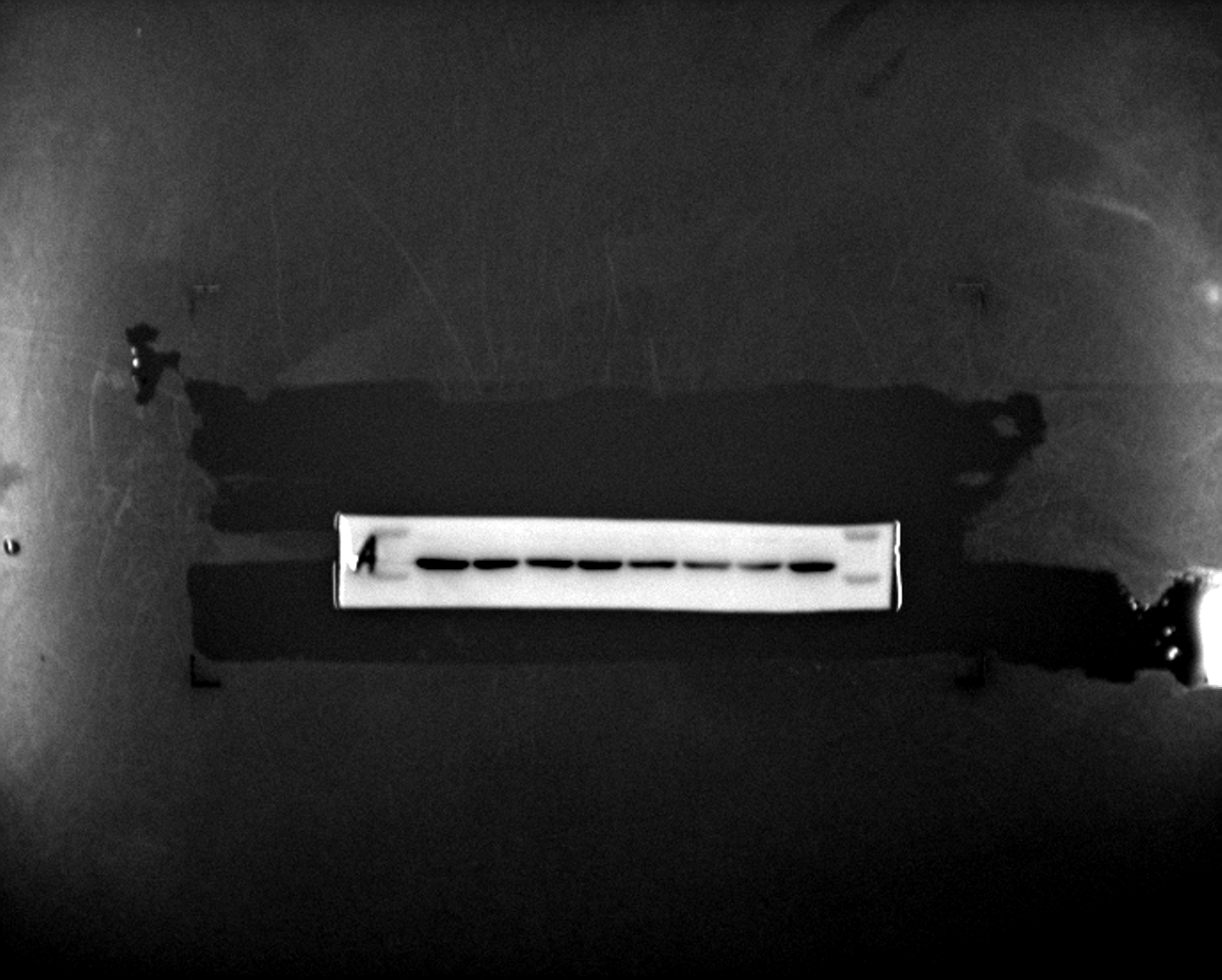

Supplement: Figure 5—source data 10. [file elife-96988-fig5-data10.zip › Figure 5-source data 10/4/β-ACTIN.Tif]

# Figure 5K

1.

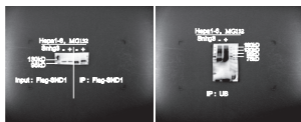

2.

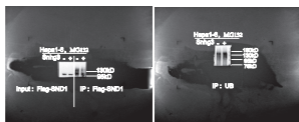

3.

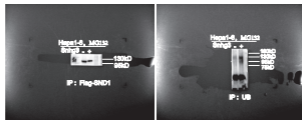

Supplement: Figure 5—source data 11. [file elife-96988-fig5-data11.pdf]

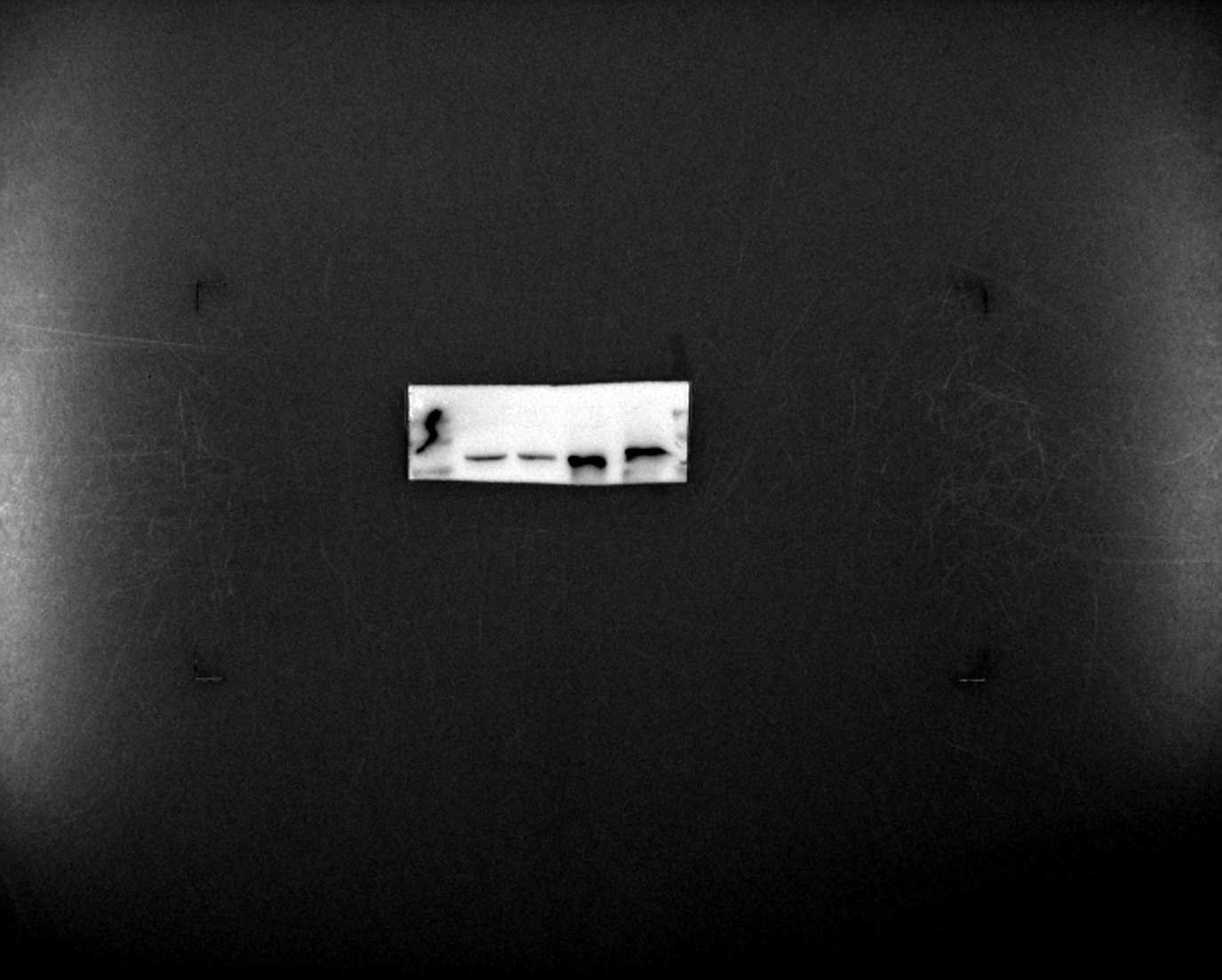

Supplement: Figure 5—source data 12. [file elife-96988-fig5-data12.zip › Figure 5-source data 12/1/SND1.Tif]

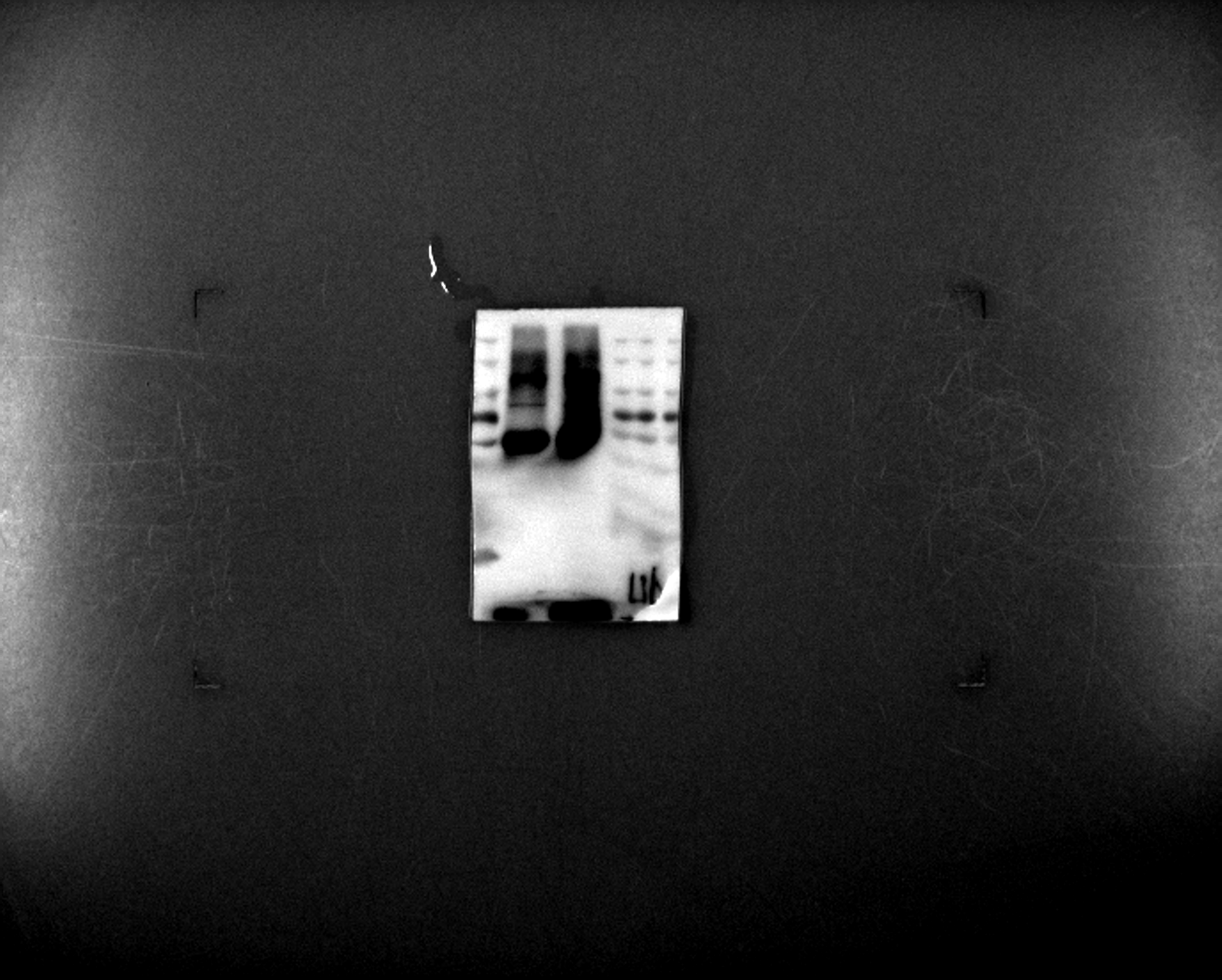

Supplement: Figure 5—source data 12. [file elife-96988-fig5-data12.zip › Figure 5-source data 12/1/UB.Tif]

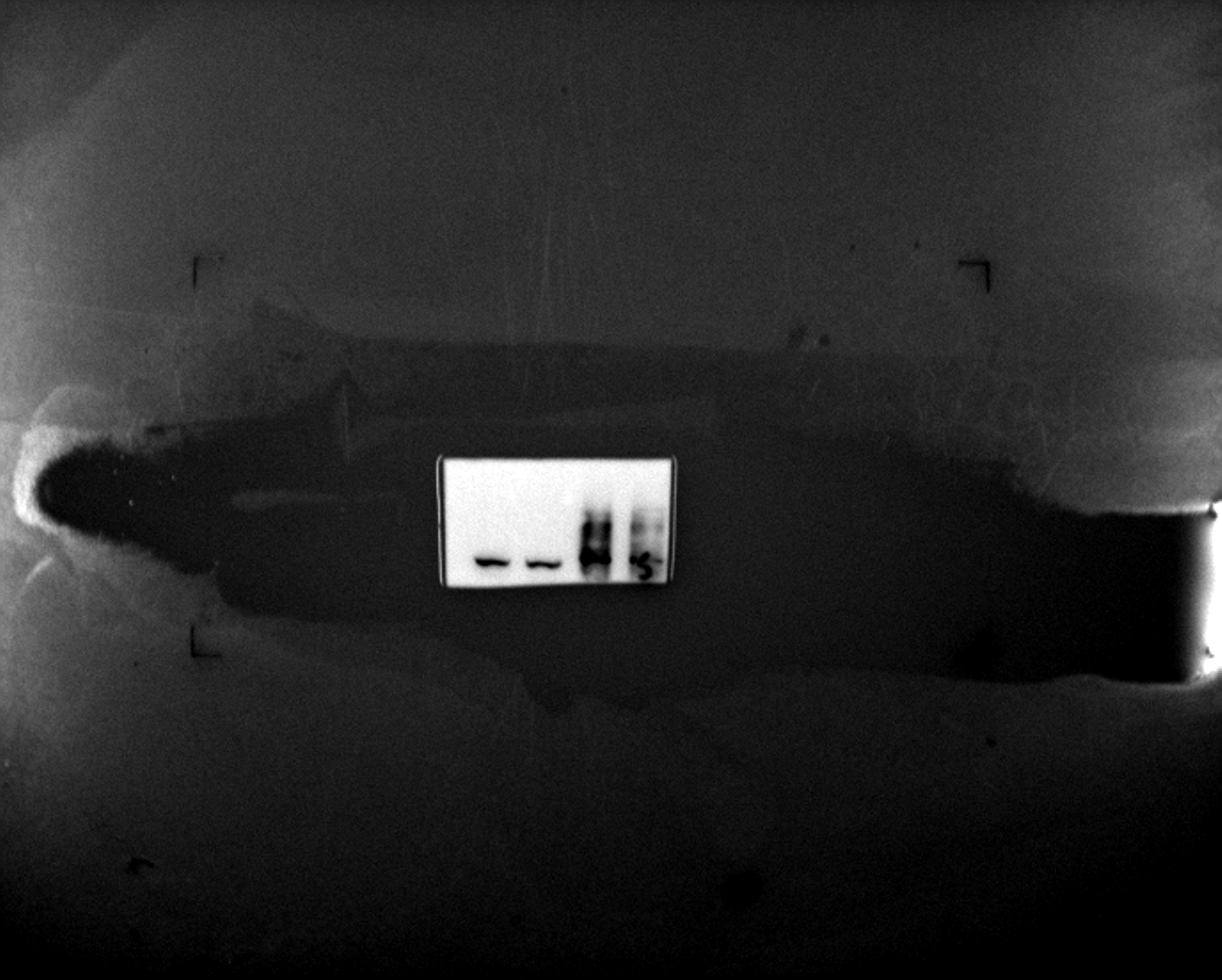

Supplement: Figure 5—source data 12. [file elife-96988-fig5-data12.zip › Figure 5-source data 12/2/SND1.Tif]

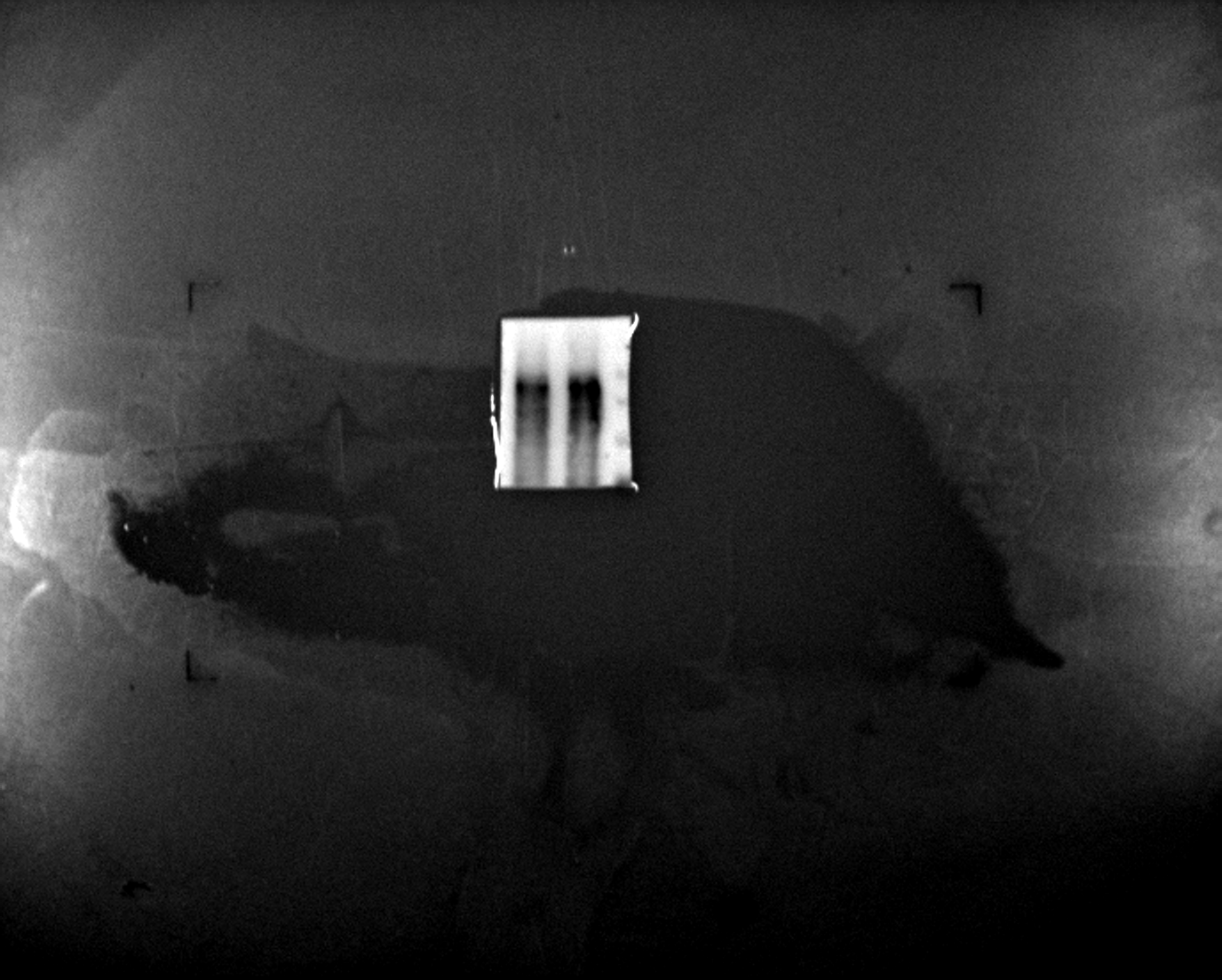

Supplement: Figure 5—source data 12. [file elife-96988-fig5-data12.zip › Figure 5-source data 12/2/Ub.Tif]

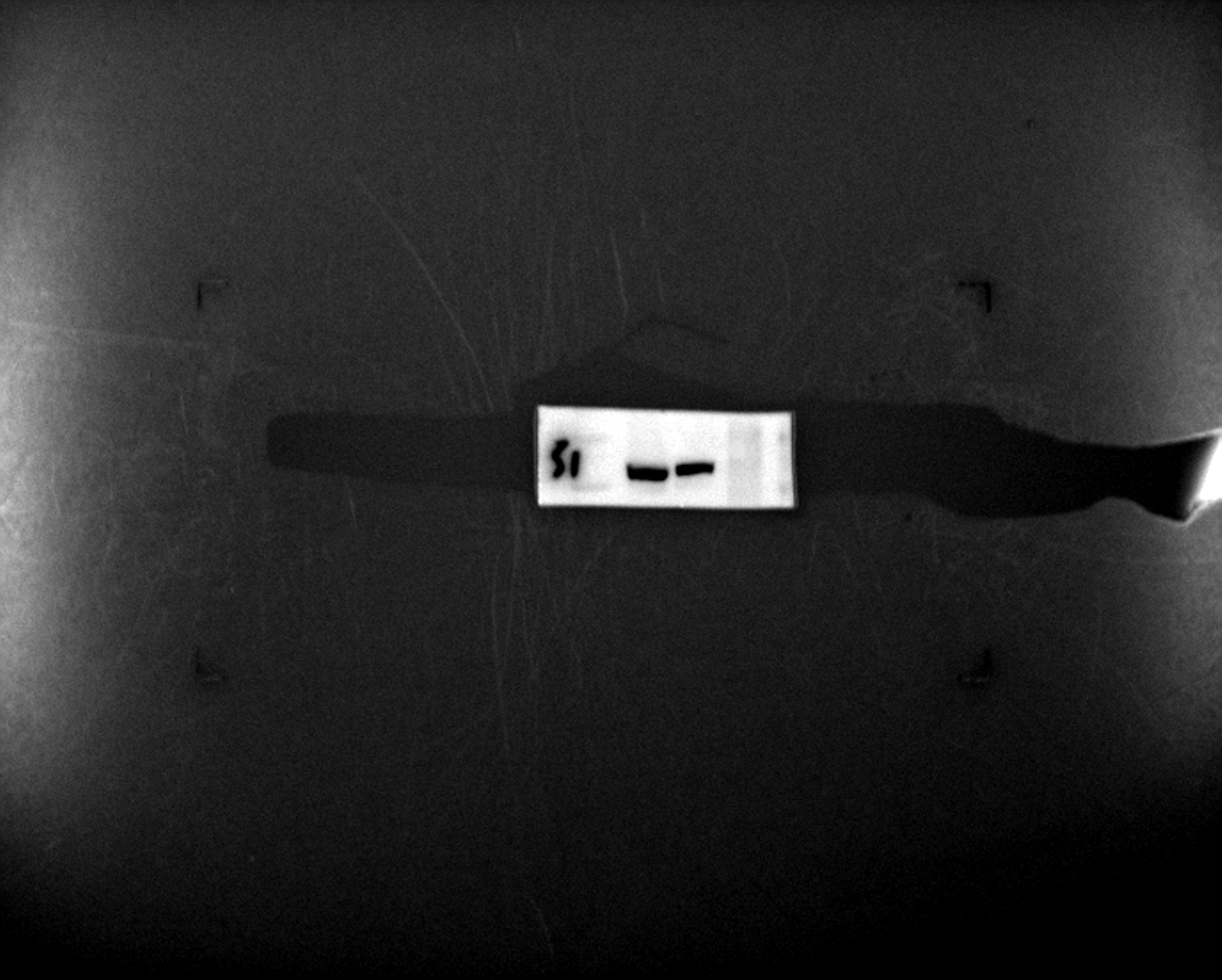

Supplement: Figure 5—source data 12. [file elife-96988-fig5-data12.zip › Figure 5-source data 12/3/IP-SND1.Tif]

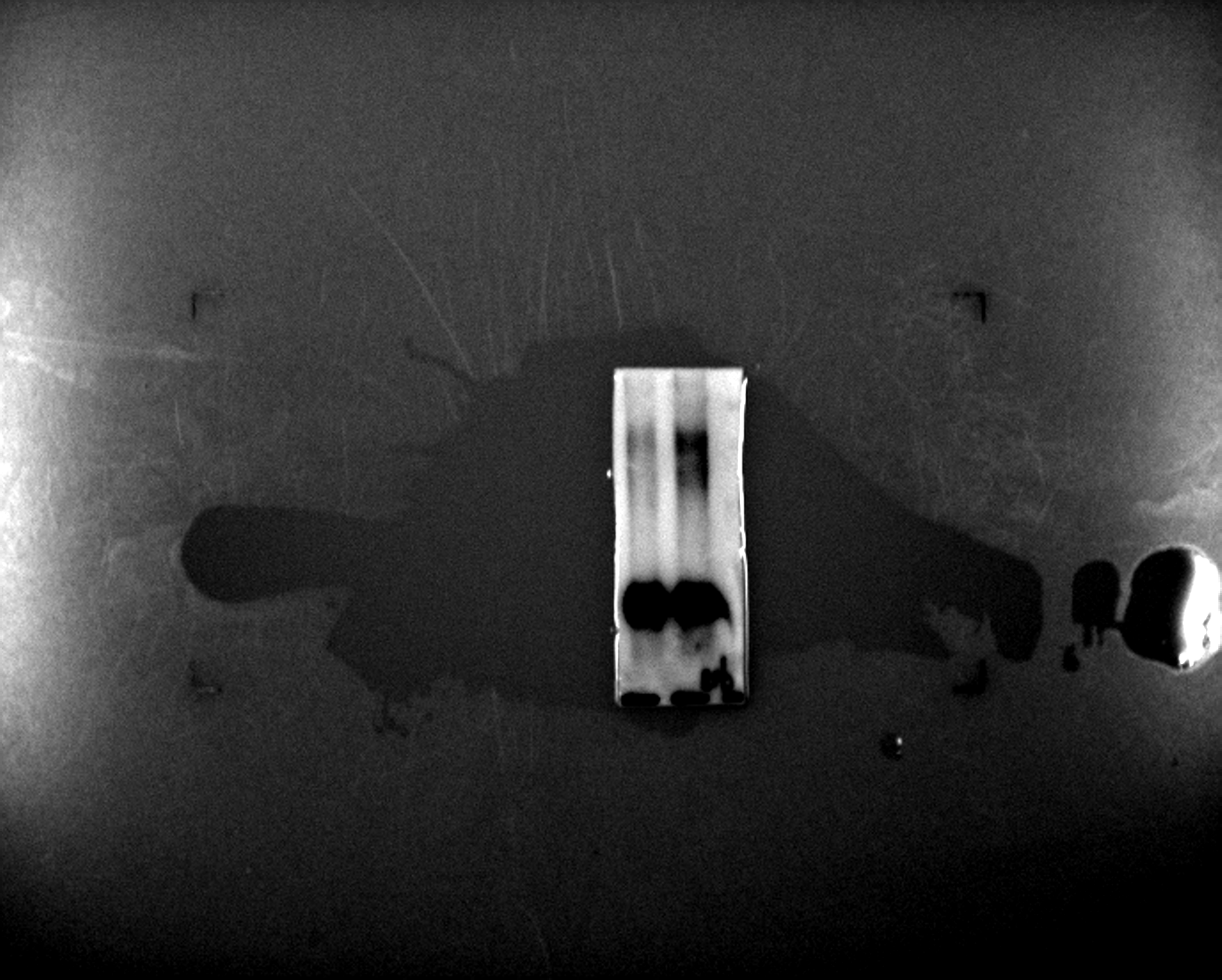

Supplement: Figure 5—source data 12. [file elife-96988-fig5-data12.zip › Figure 5-source data 12/3/UB.Tif]

# Figure 5L

1.

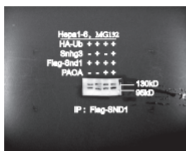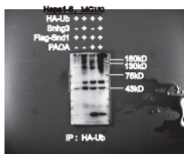

2.

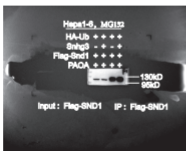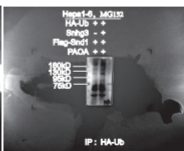

Supplement: Figure 5—source data 13. [file elife-96988-fig5-data13.pdf]

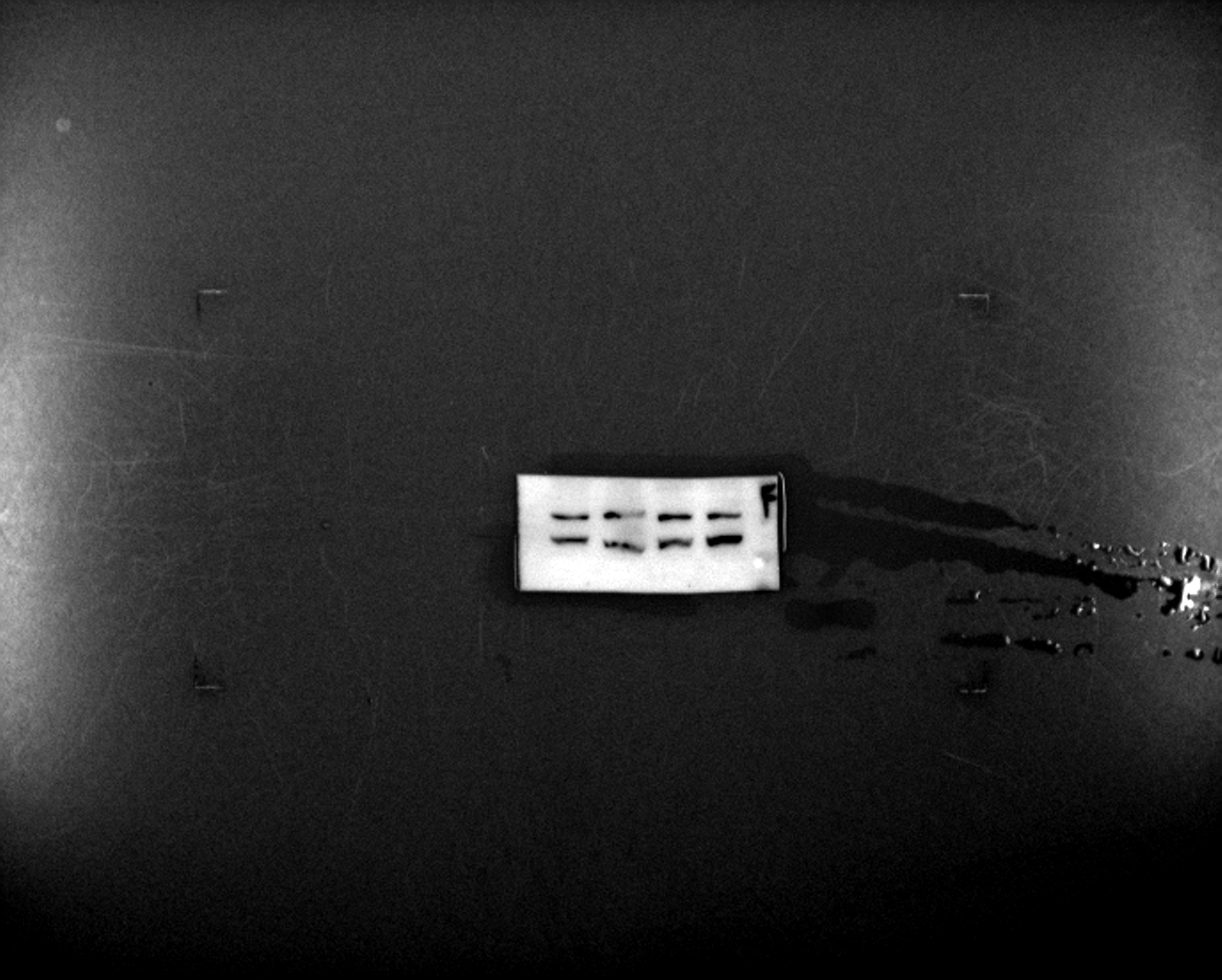

Supplement: Figure 5—source data 14. [file elife-96988-fig5-data14.zip › Figure 5-source data 14/1/IP-Flag-SND1.Tif]

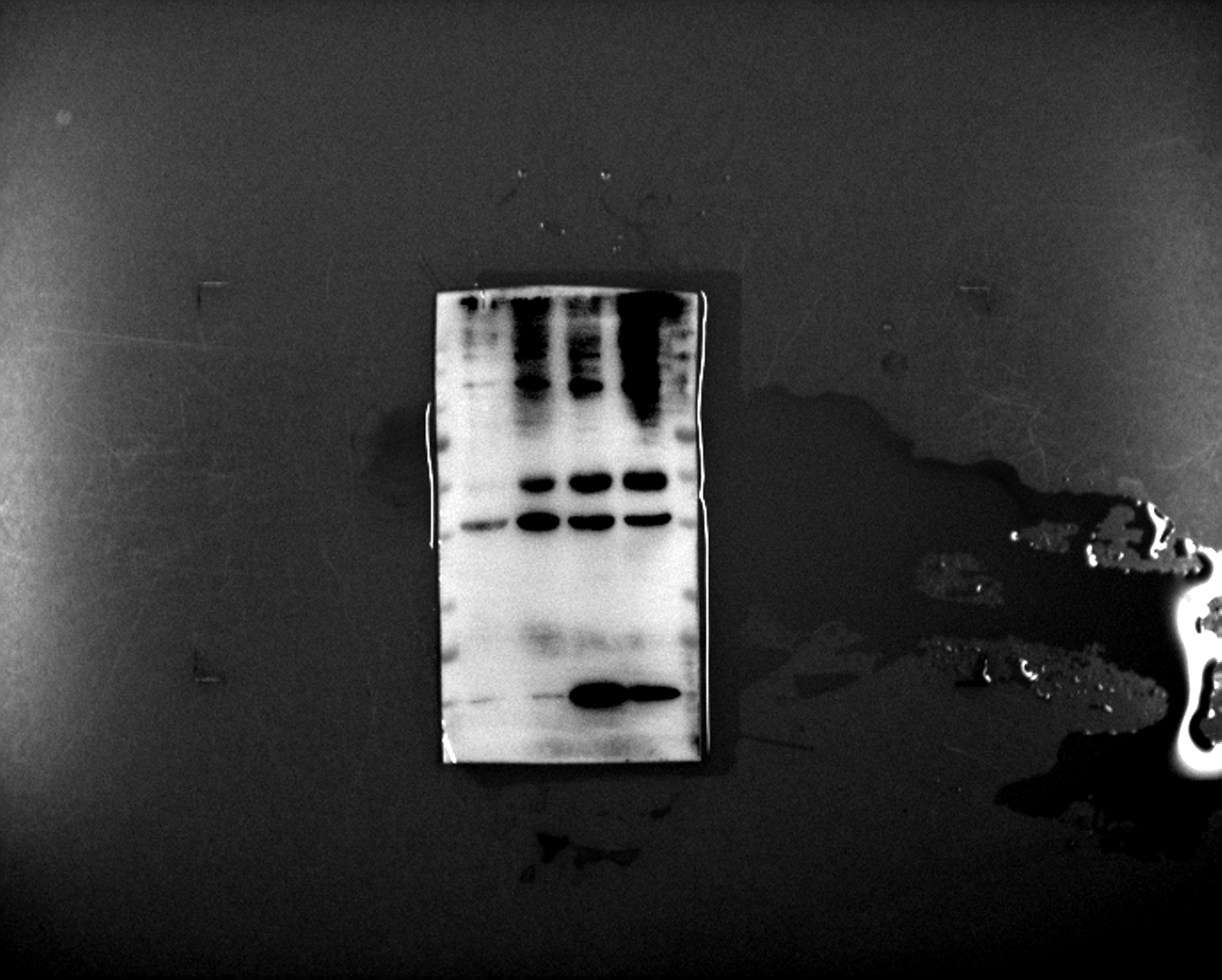

Supplement: Figure 5—source data 14. [file elife-96988-fig5-data14.zip › Figure 5-source data 14/1/IP-HA-Ub.Tif]

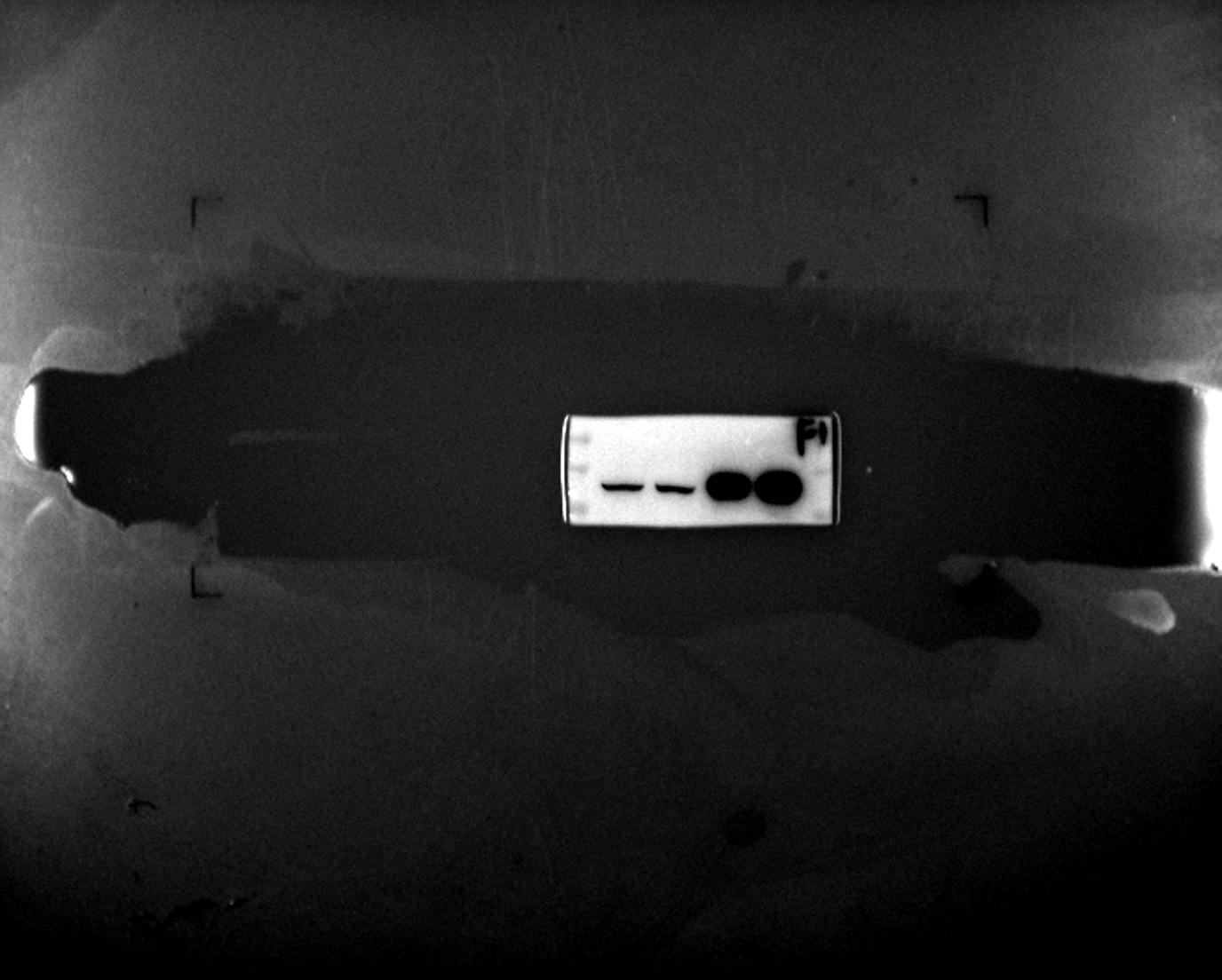

Supplement: Figure 5—source data 14. [file elife-96988-fig5-data14.zip › Figure 5-source data 14/2/Flag-SND1.Tif]

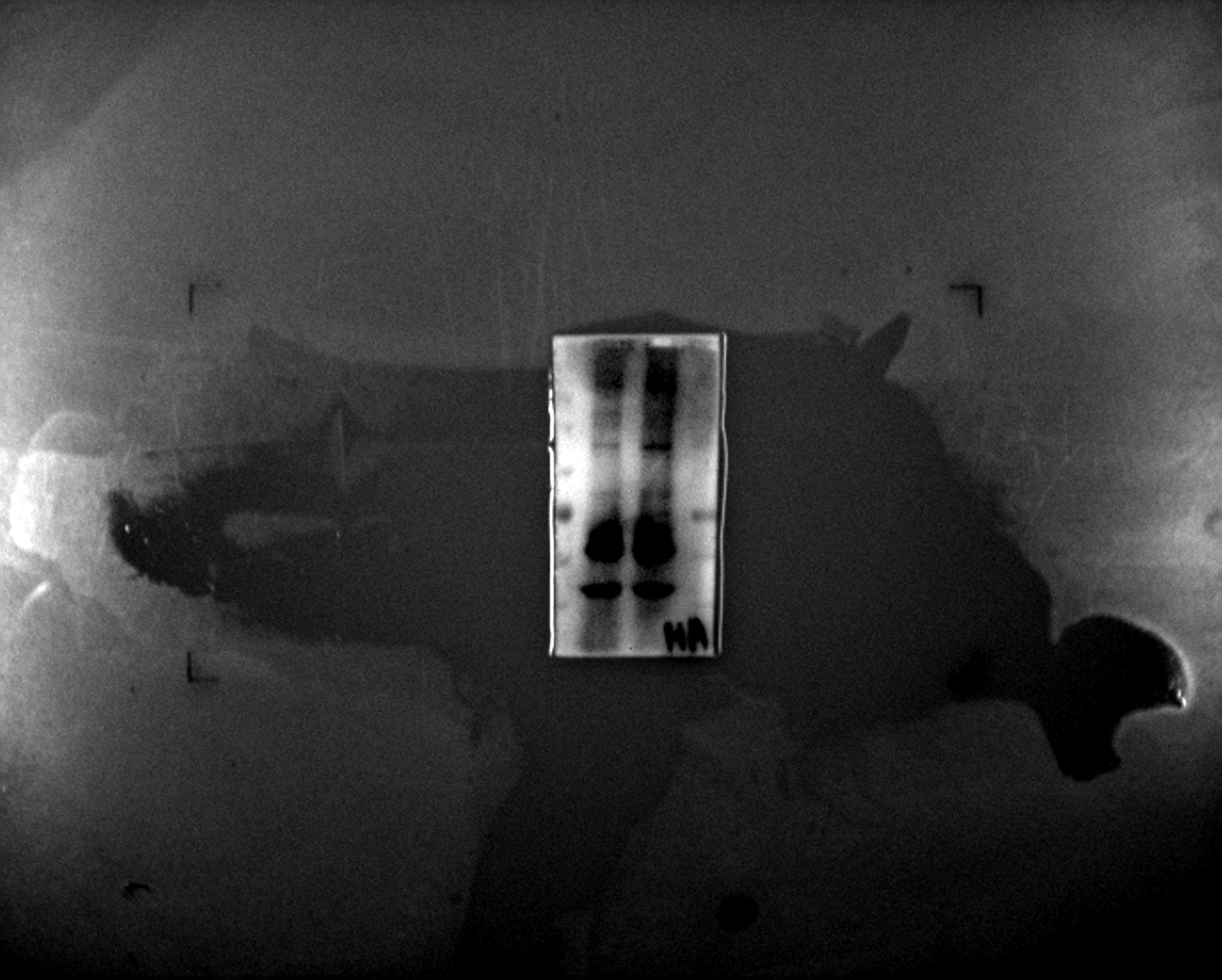

Supplement: Figure 5—source data 14. [file elife-96988-fig5-data14.zip › Figure 5-source data 14/2/HA-Ub.Tif]

# Figure 5M

1

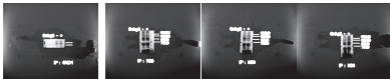

2

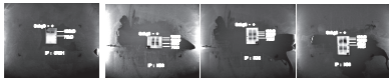

Supplement: Figure 5—source data 15. [file elife-96988-fig5-data15.pdf]

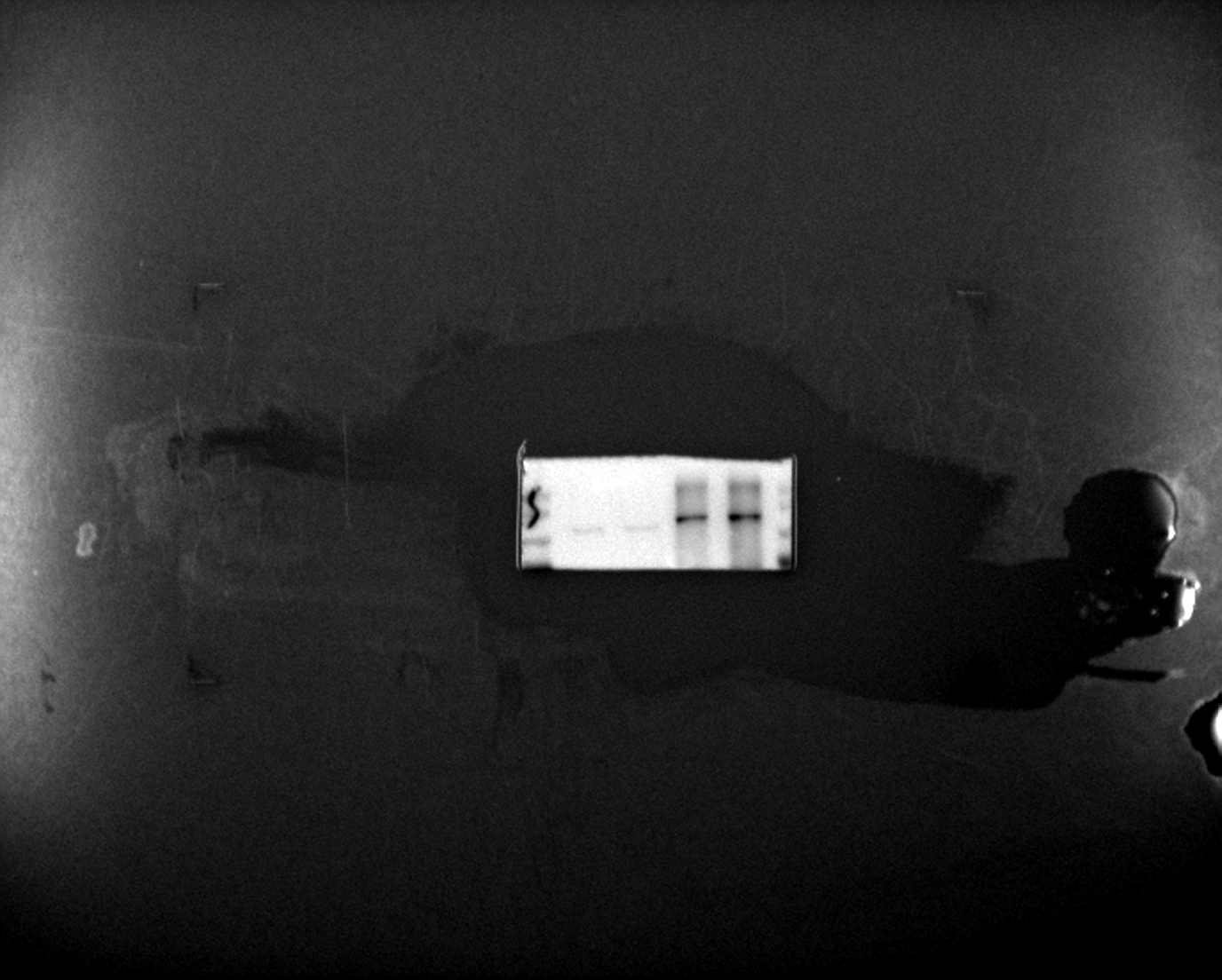

Supplement: Figure 5—source data 16. [file elife-96988-fig5-data16.zip › Figure 5-source data 16/1/IP-SND1.Tif]

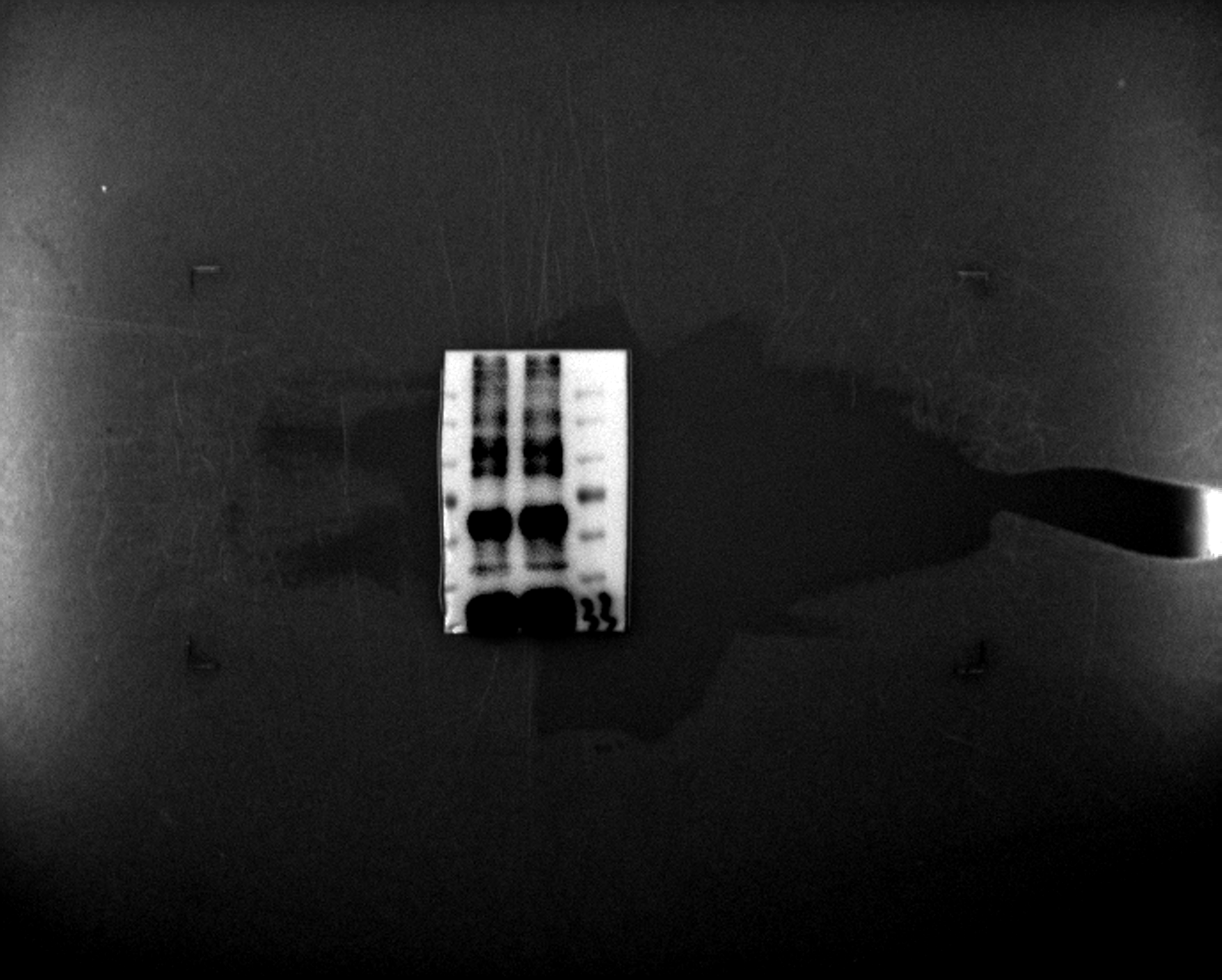

Supplement: Figure 5—source data 16. [file elife-96988-fig5-data16.zip › Figure 5-source data 16/1/K33.Tif]

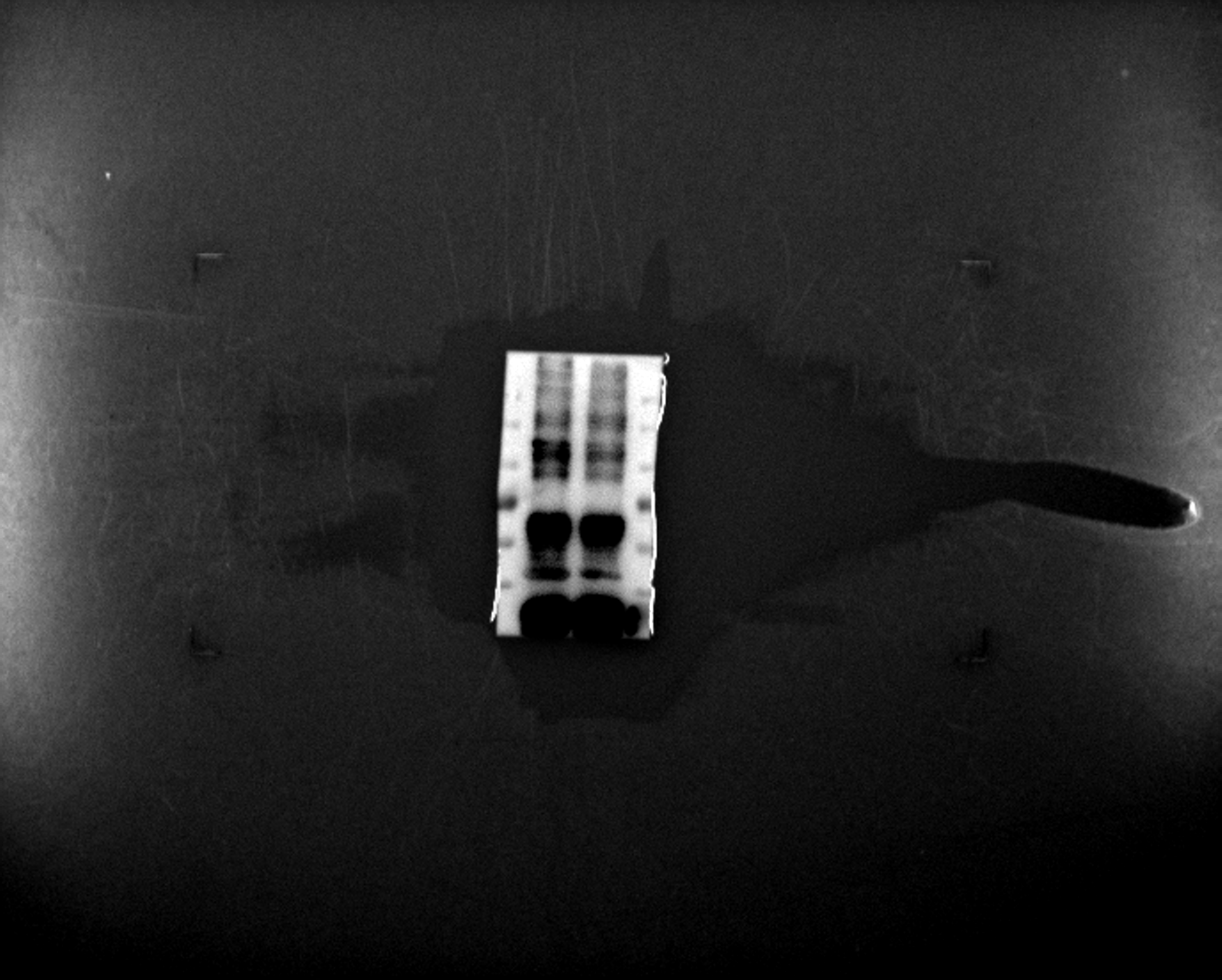

Supplement: Figure 5—source data 16. [file elife-96988-fig5-data16.zip › Figure 5-source data 16/1/K48.Tif]

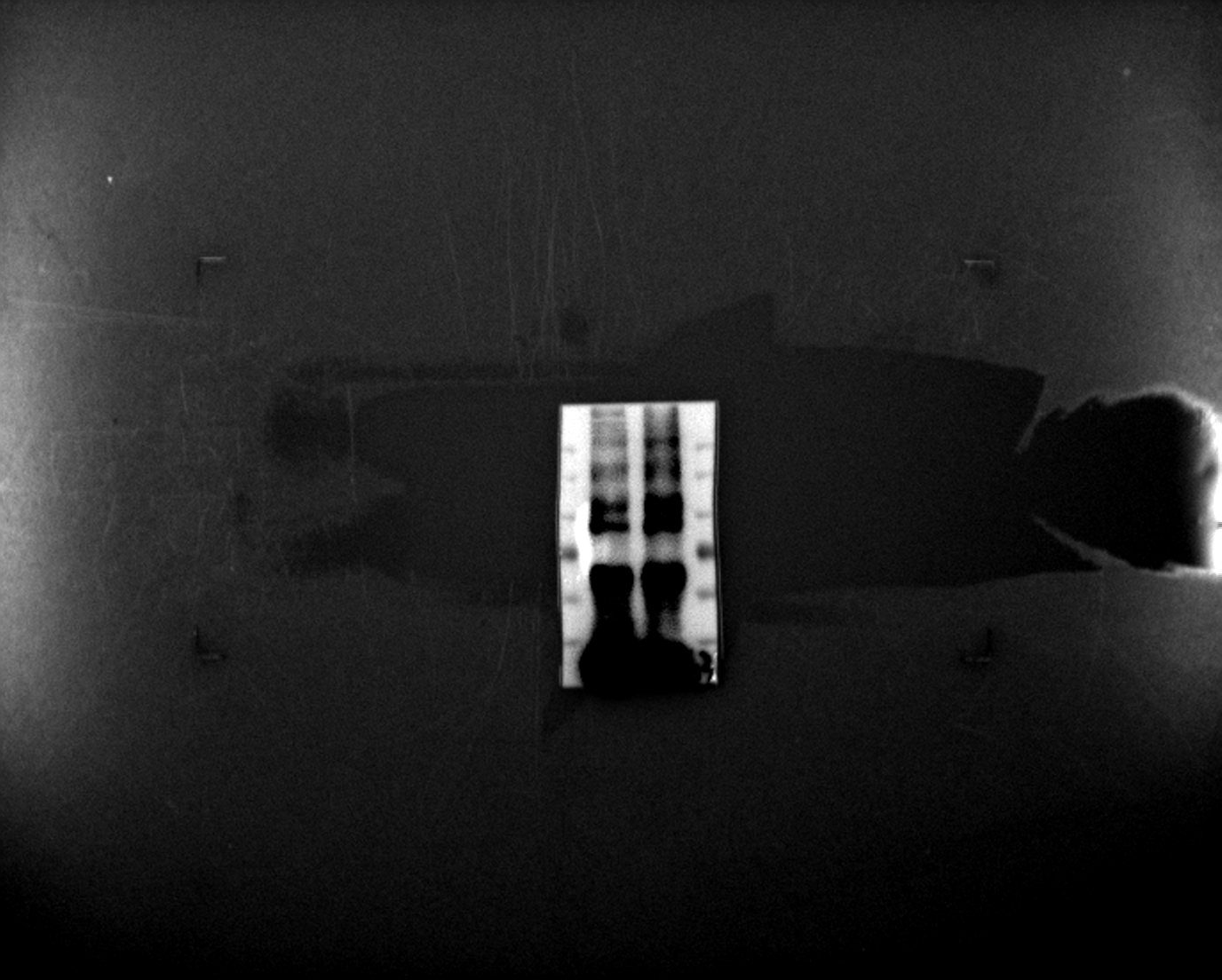

Supplement: Figure 5—source data 16. [file elife-96988-fig5-data16.zip › Figure 5-source data 16/1/K63.Tif]

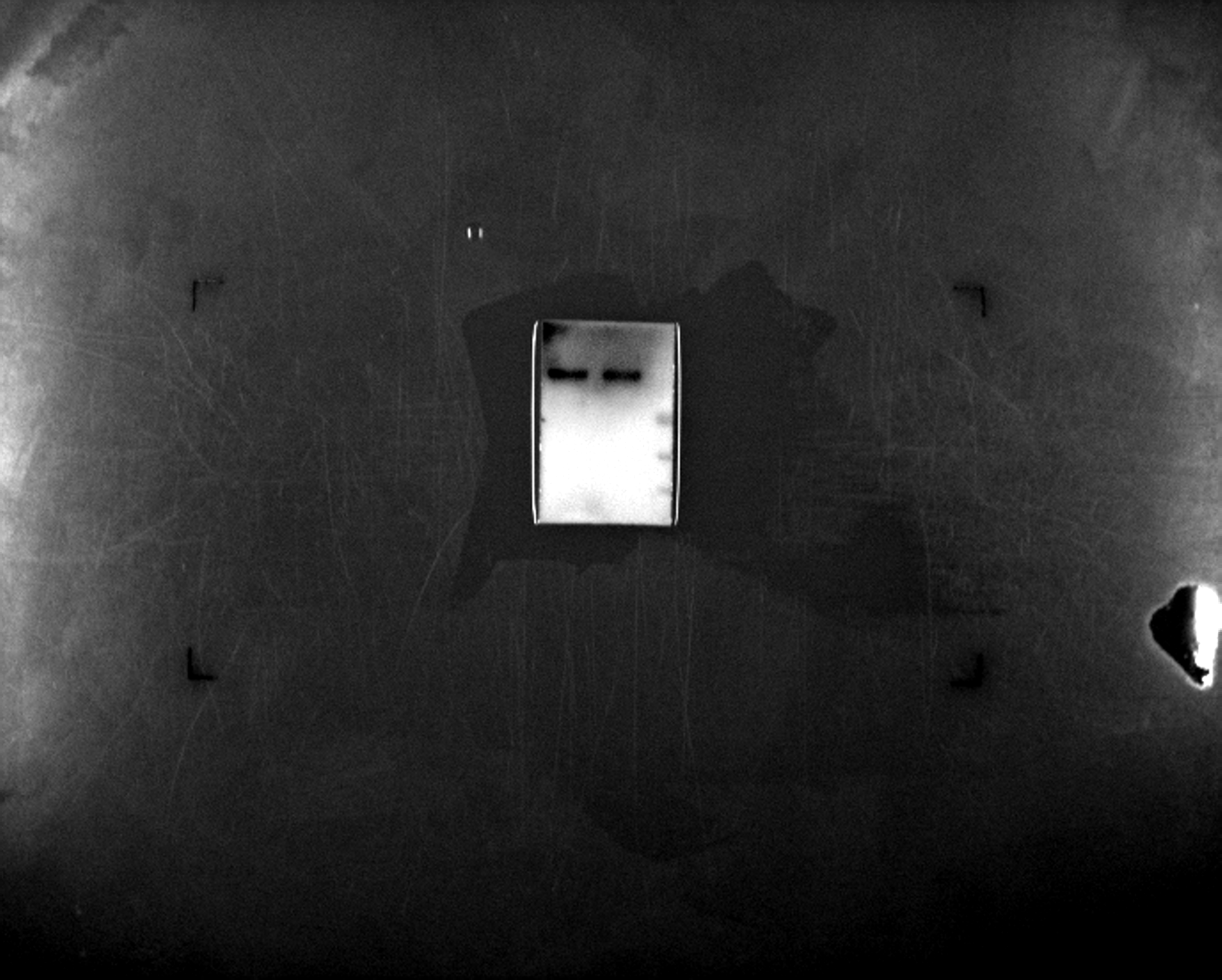

Supplement: Figure 5—source data 16. [file elife-96988-fig5-data16.zip › Figure 5-source data 16/2/IP-SND1.Tif]

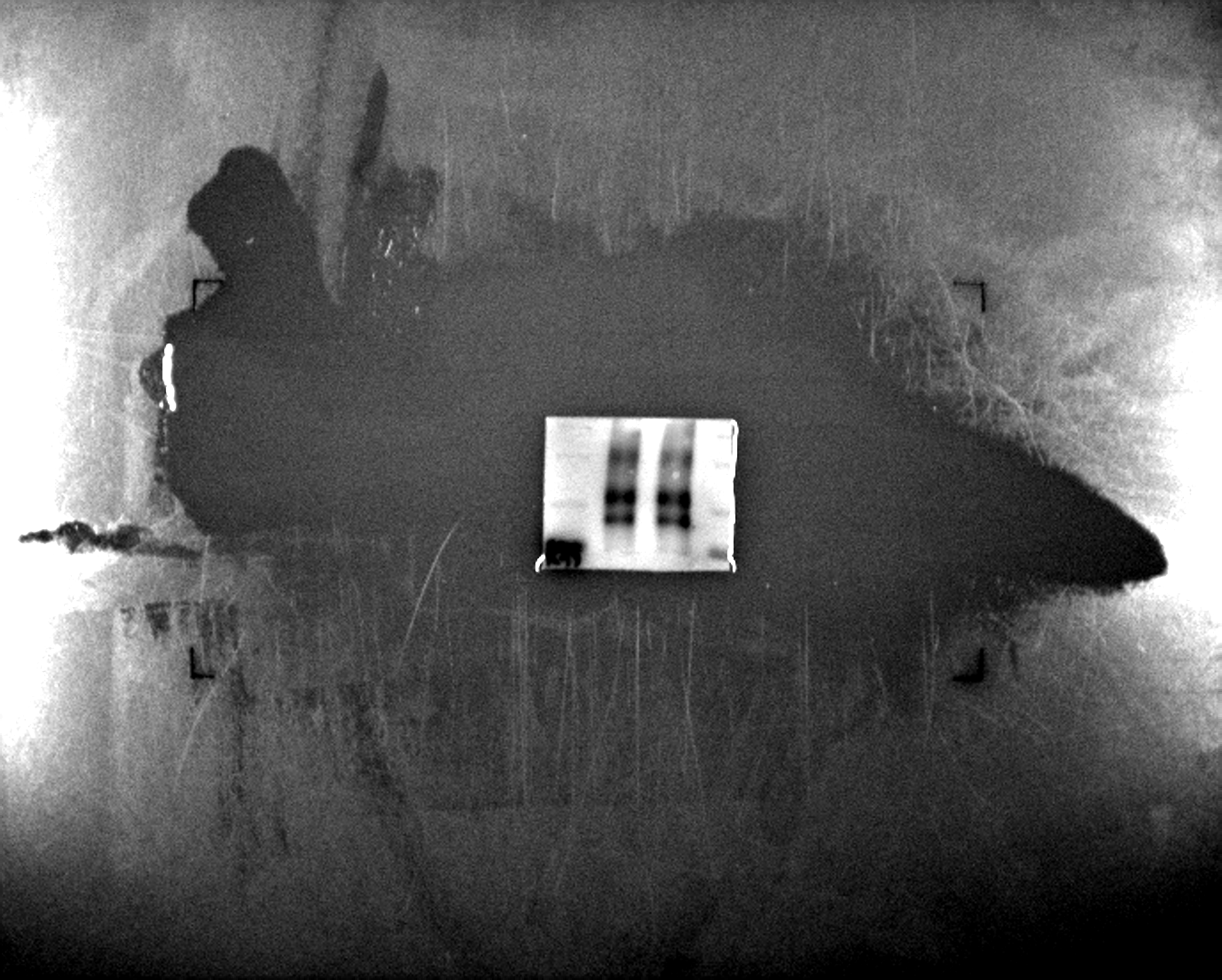

Supplement: Figure 5—source data 16. [file elife-96988-fig5-data16.zip › Figure 5-source data 16/2/K33.Tif]

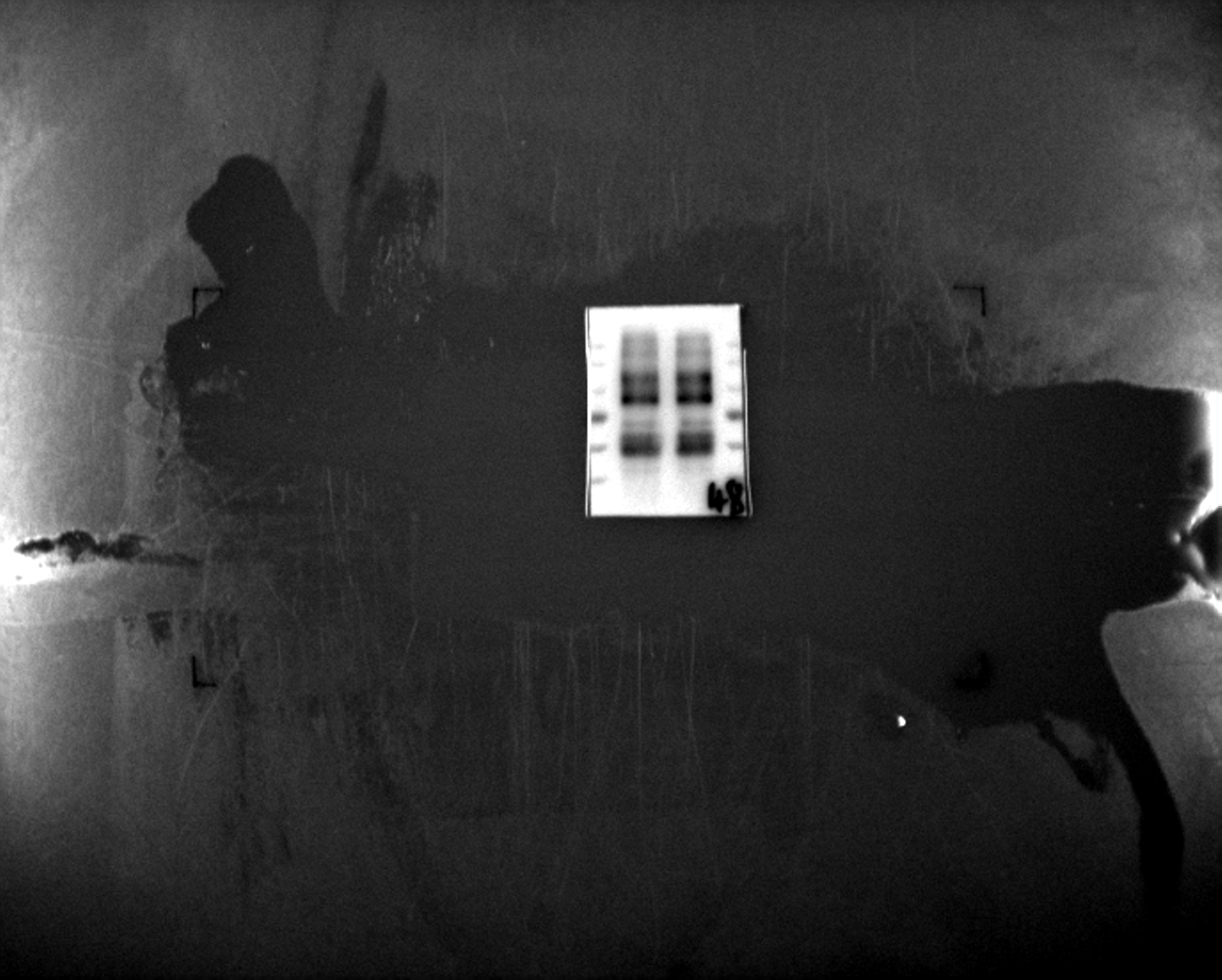

Supplement: Figure 5—source data 16. [file elife-96988-fig5-data16.zip › Figure 5-source data 16/2/K48.Tif]

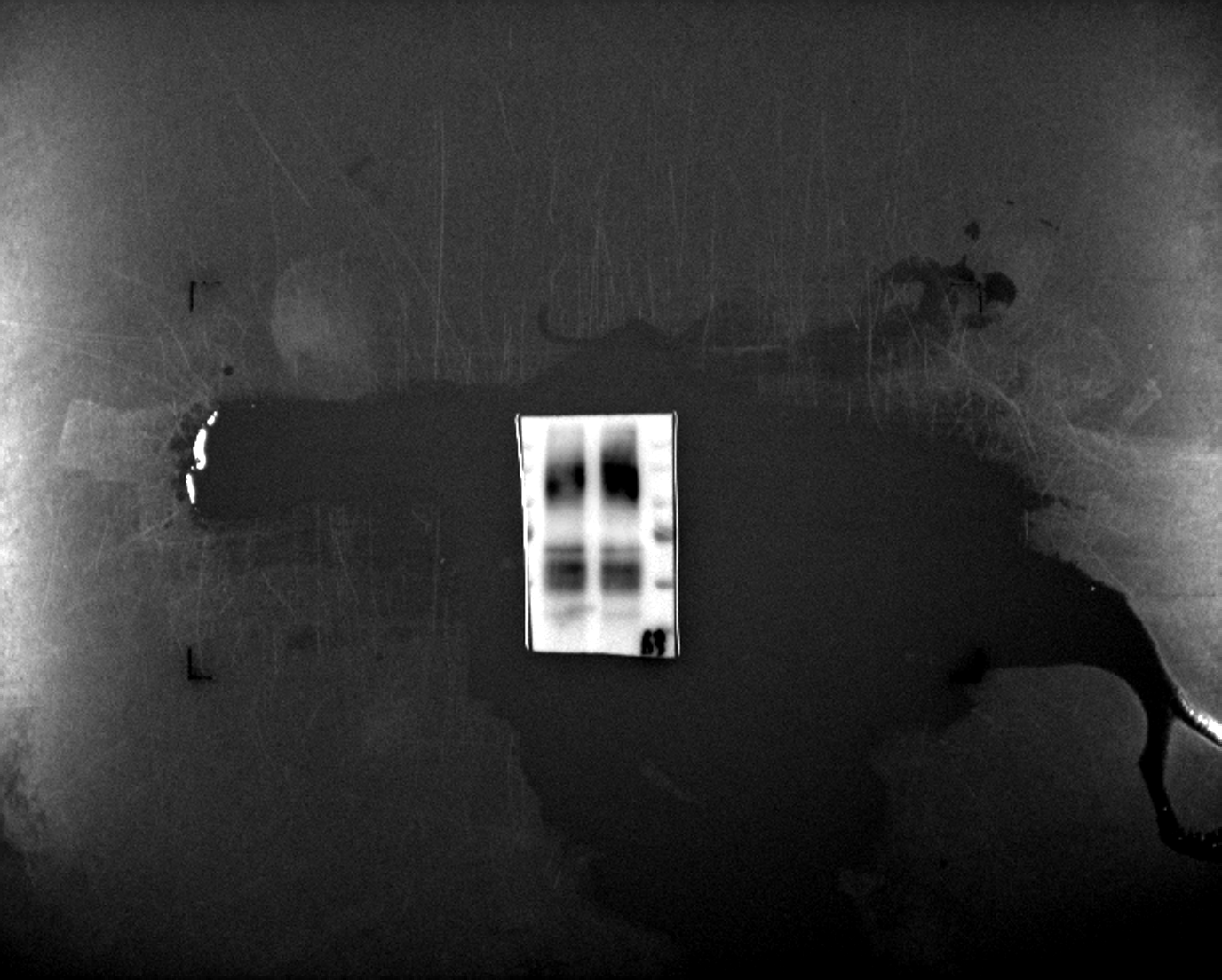

Supplement: Figure 5—source data 16. [file elife-96988-fig5-data16.zip › Figure 5-source data 16/2/K63.Tif]

Figure 5N

1.

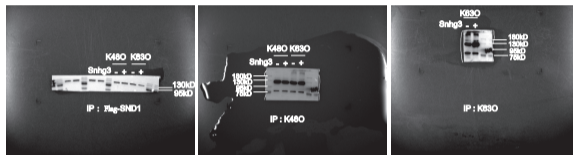

2.

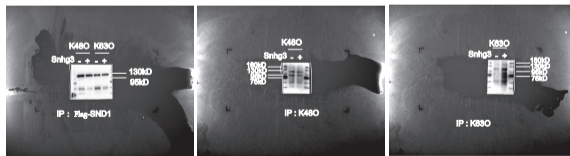

Supplement: Figure 5—source data 17. [file elife-96988-fig5-data17.pdf]

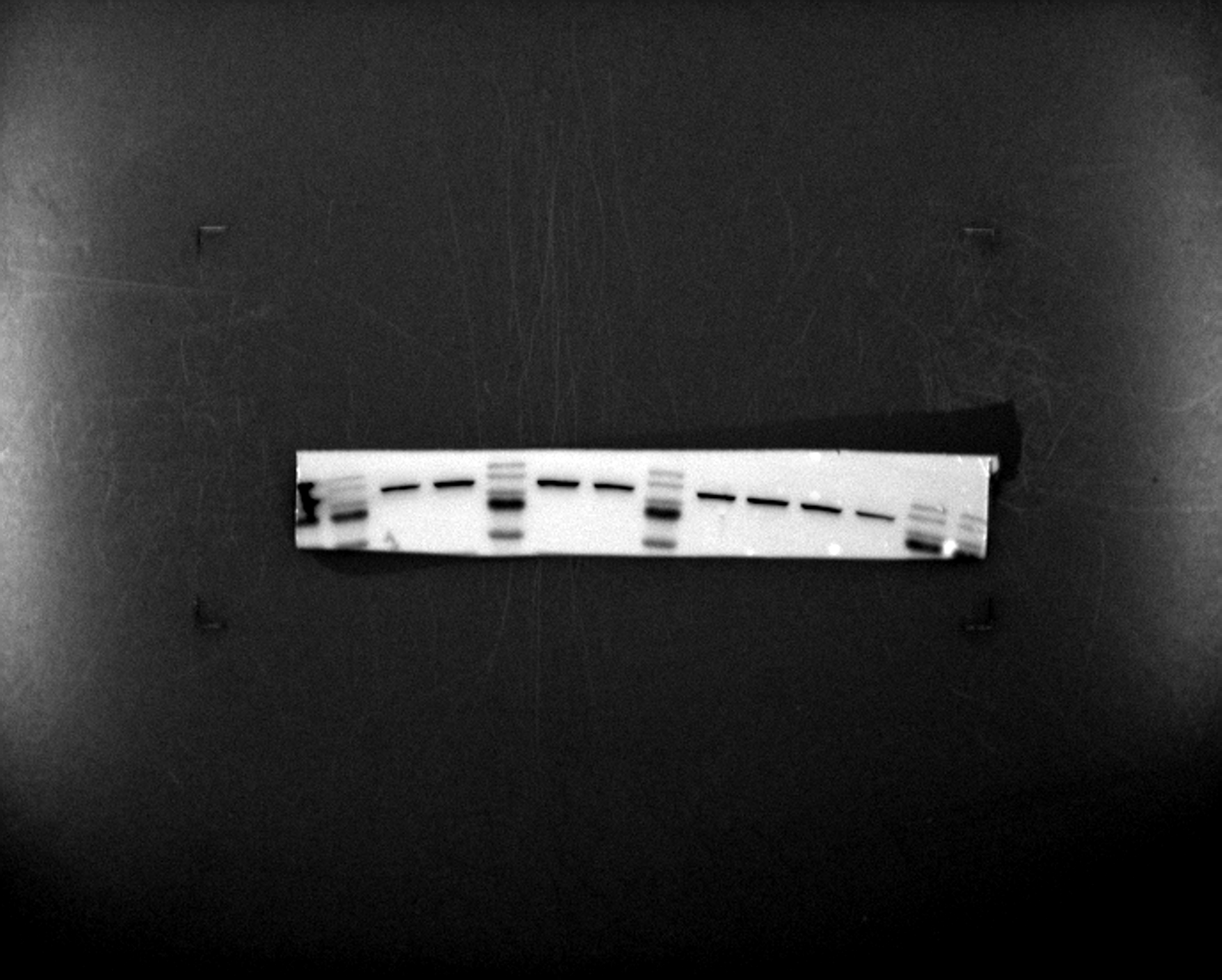

Supplement: Figure 5—source data 18. [file elife-96988-fig5-data18.zip › Figure 5-source data 18/1/IP-Flag-SND1.Tif]

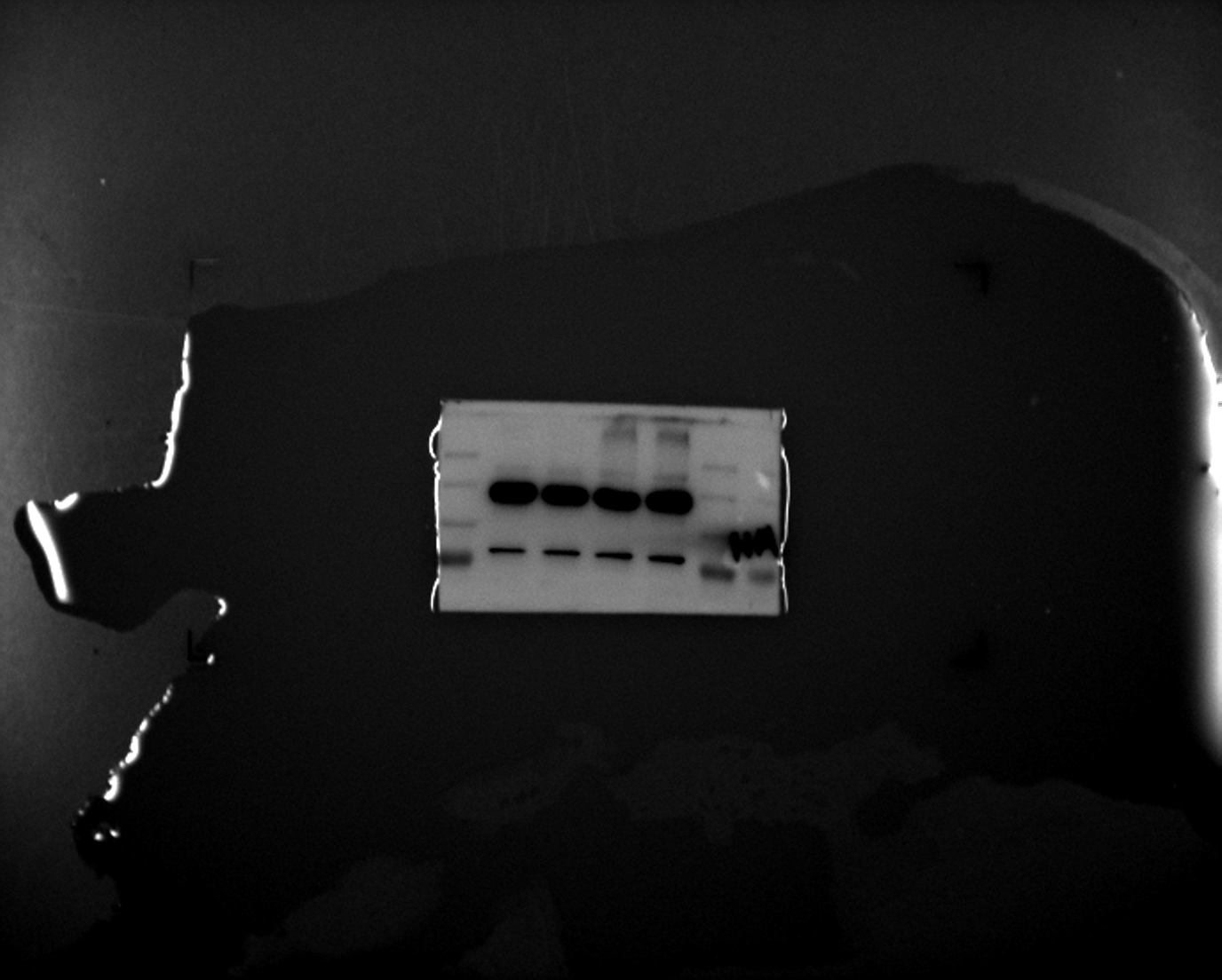

Supplement: Figure 5—source data 18. [file elife-96988-fig5-data18.zip › Figure 5-source data 18/1/K48O.Tif]

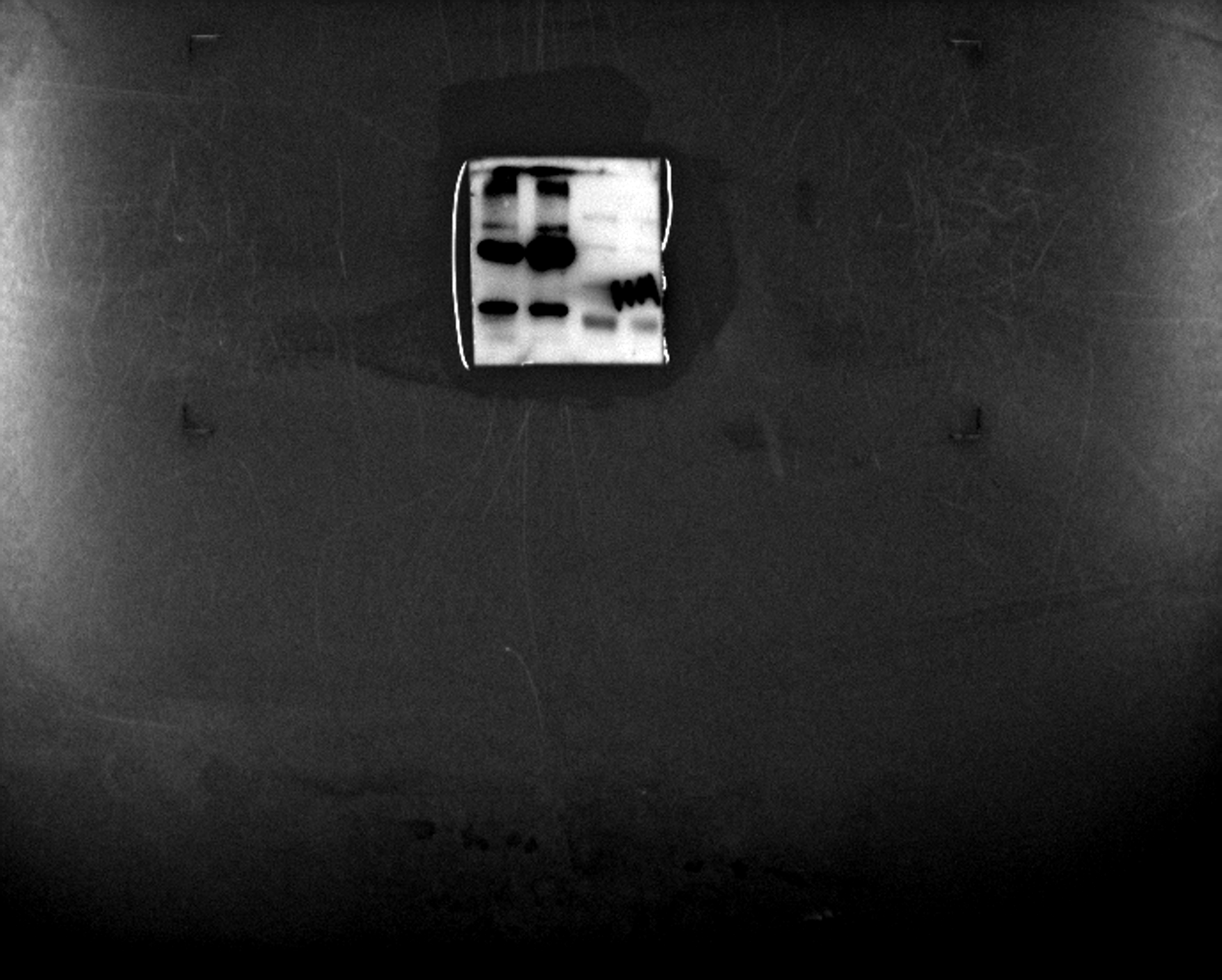

Supplement: Figure 5—source data 18. [file elife-96988-fig5-data18.zip › Figure 5-source data 18/1/K63O.Tif]

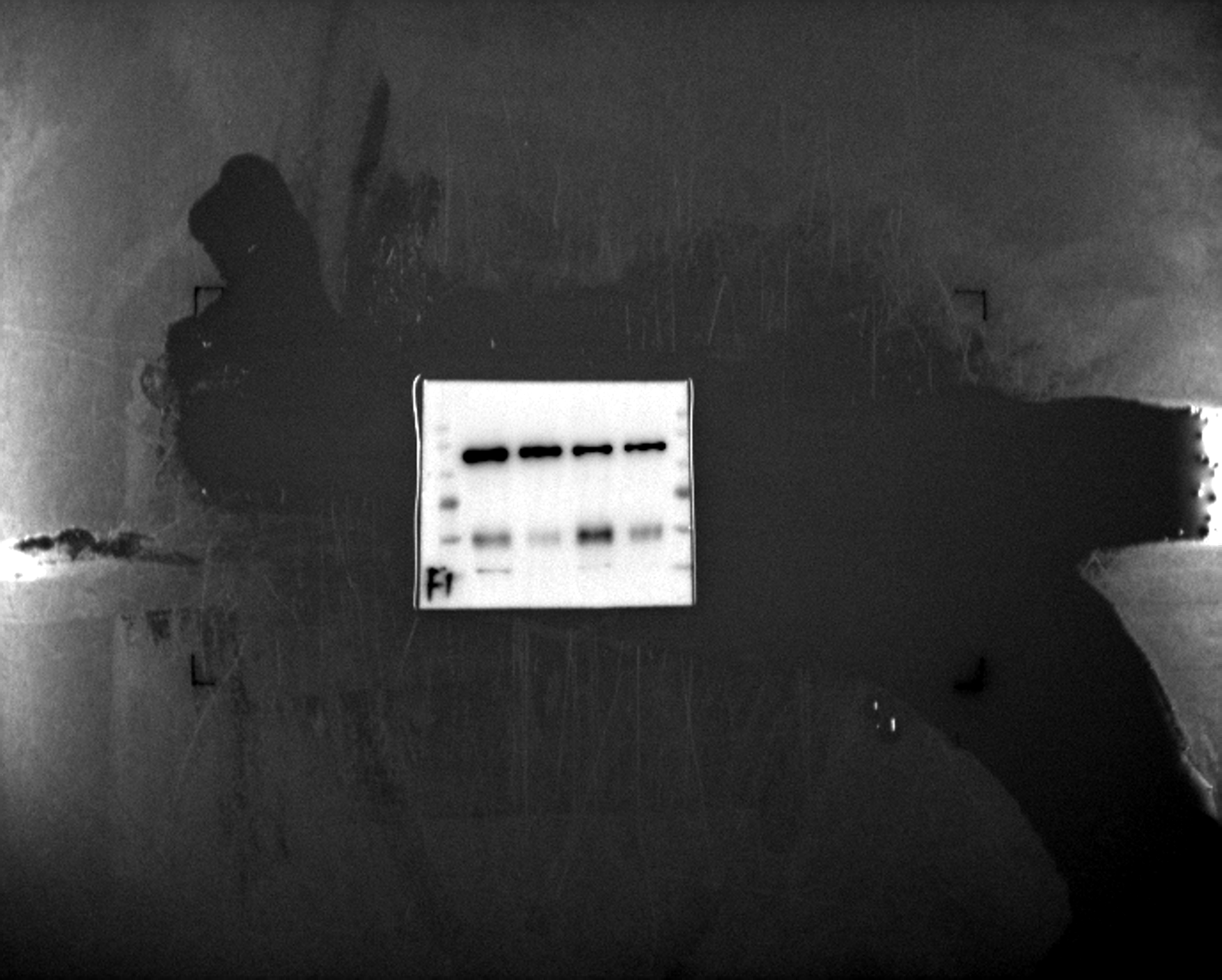

Supplement: Figure 5—source data 18. [file elife-96988-fig5-data18.zip › Figure 5-source data 18/2/IP-Flag-SND1.Tif]

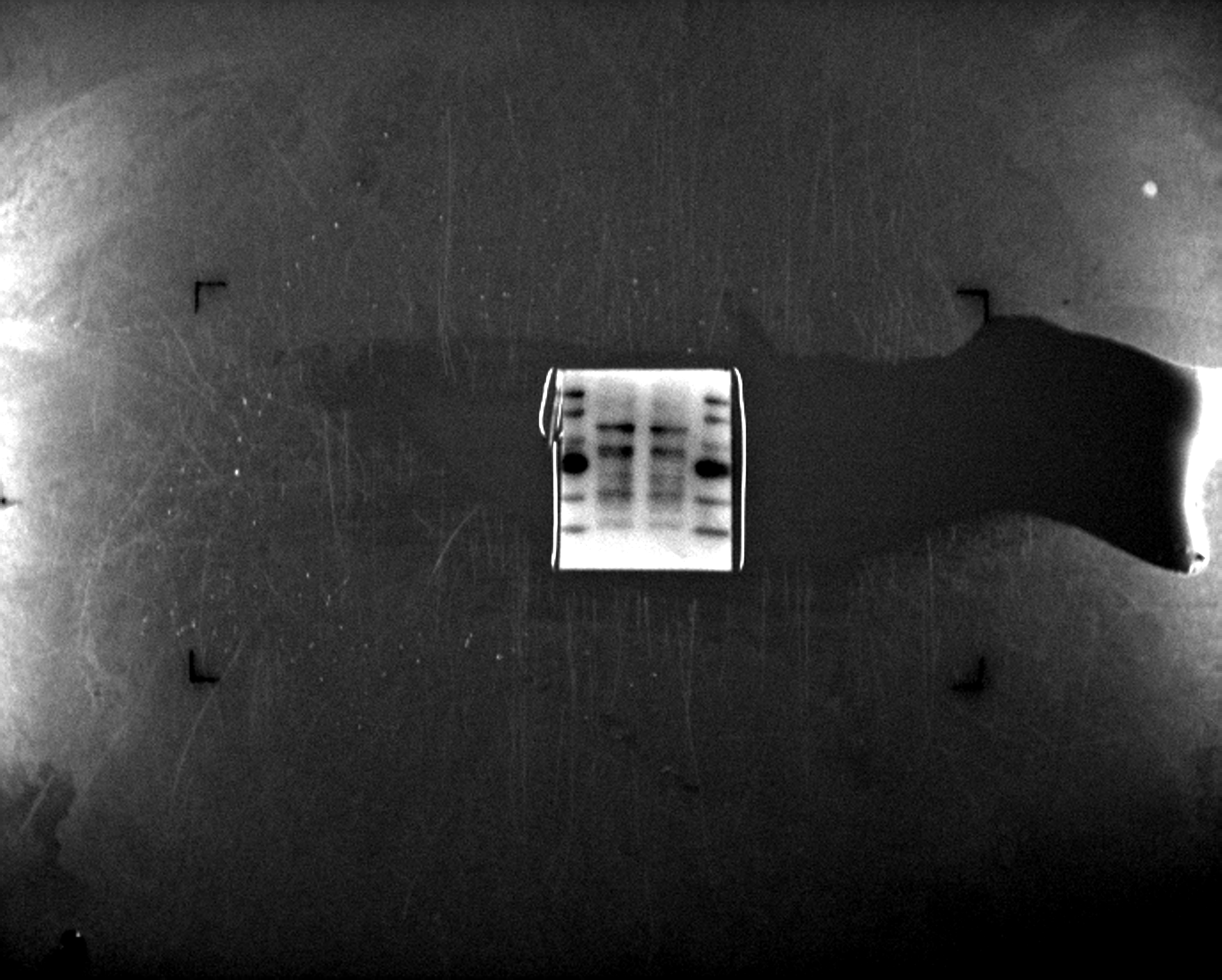

Supplement: Figure 5—source data 18. [file elife-96988-fig5-data18.zip › Figure 5-source data 18/2/K48O.Tif]

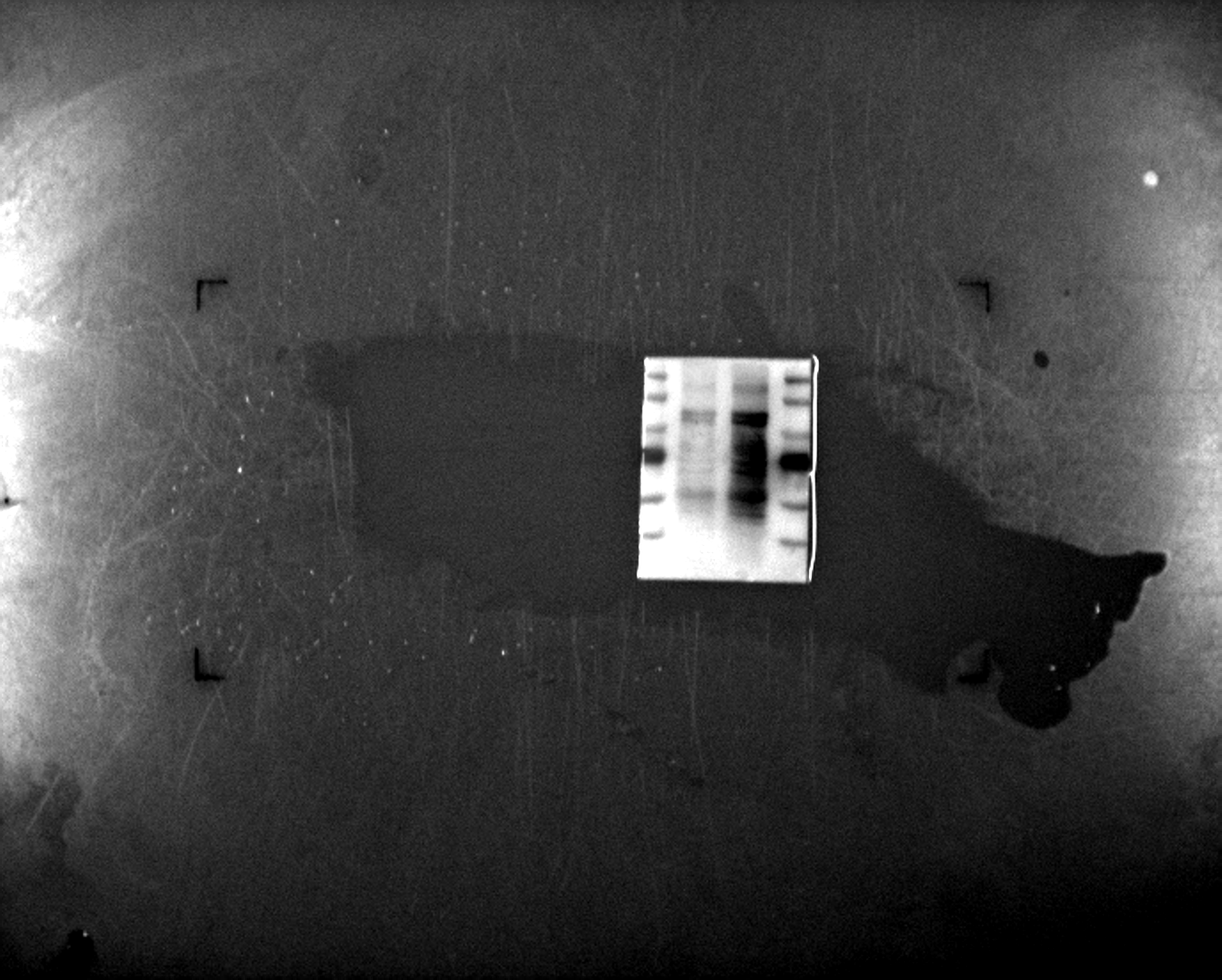

Supplement: Figure 5—source data 18. [file elife-96988-fig5-data18.zip › Figure 5-source data 18/2/K63O.Tif]

Figure 50

1.

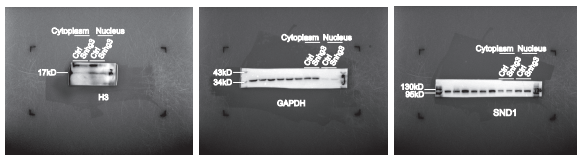

2.

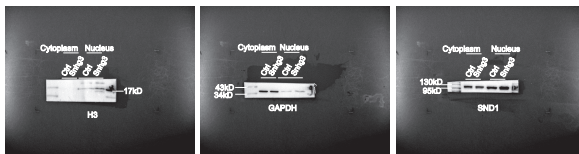

3.

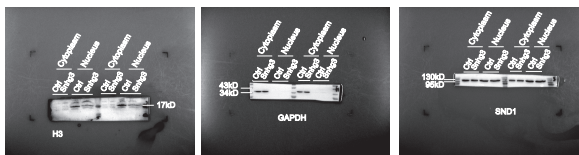

Supplement: Figure 5—source data 19. [file elife-96988-fig5-data19.pdf]

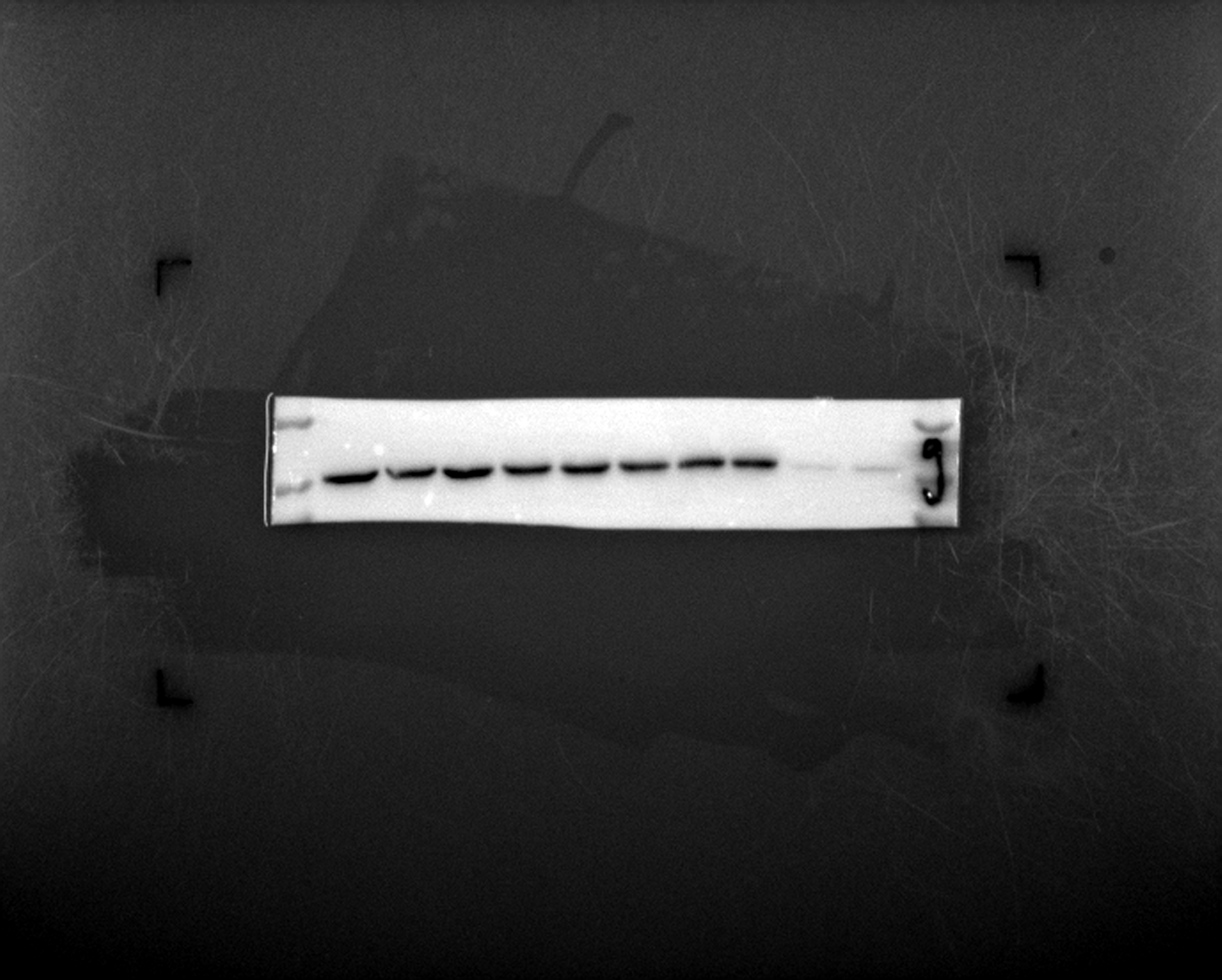

Supplement: Figure 5—source data 20. [file elife-96988-fig5-data20.zip › Figure 5-source data 20/1/GAPDH.tif]

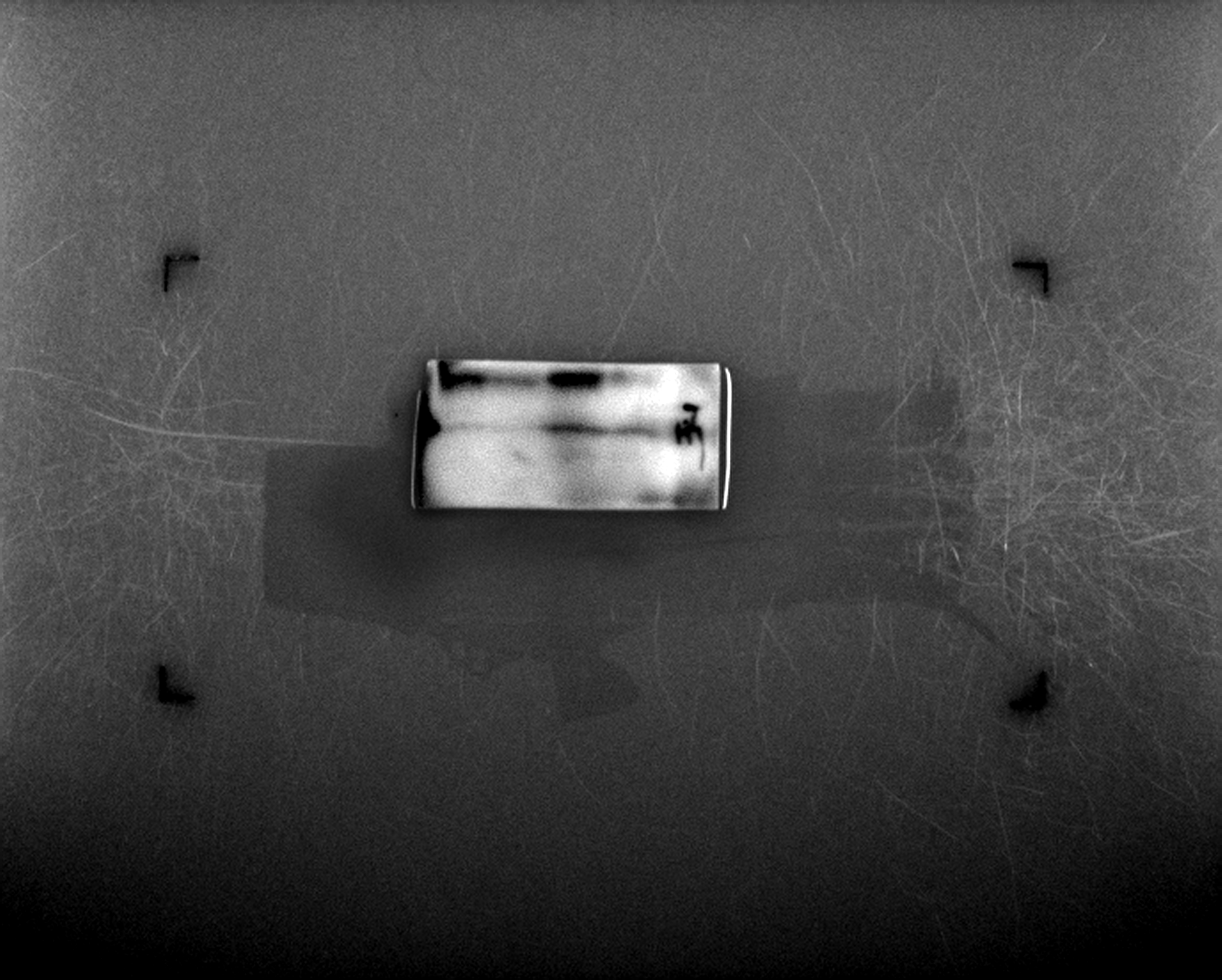

Supplement: Figure 5—source data 20. [file elife-96988-fig5-data20.zip › Figure 5-source data 20/1/H3.tif]

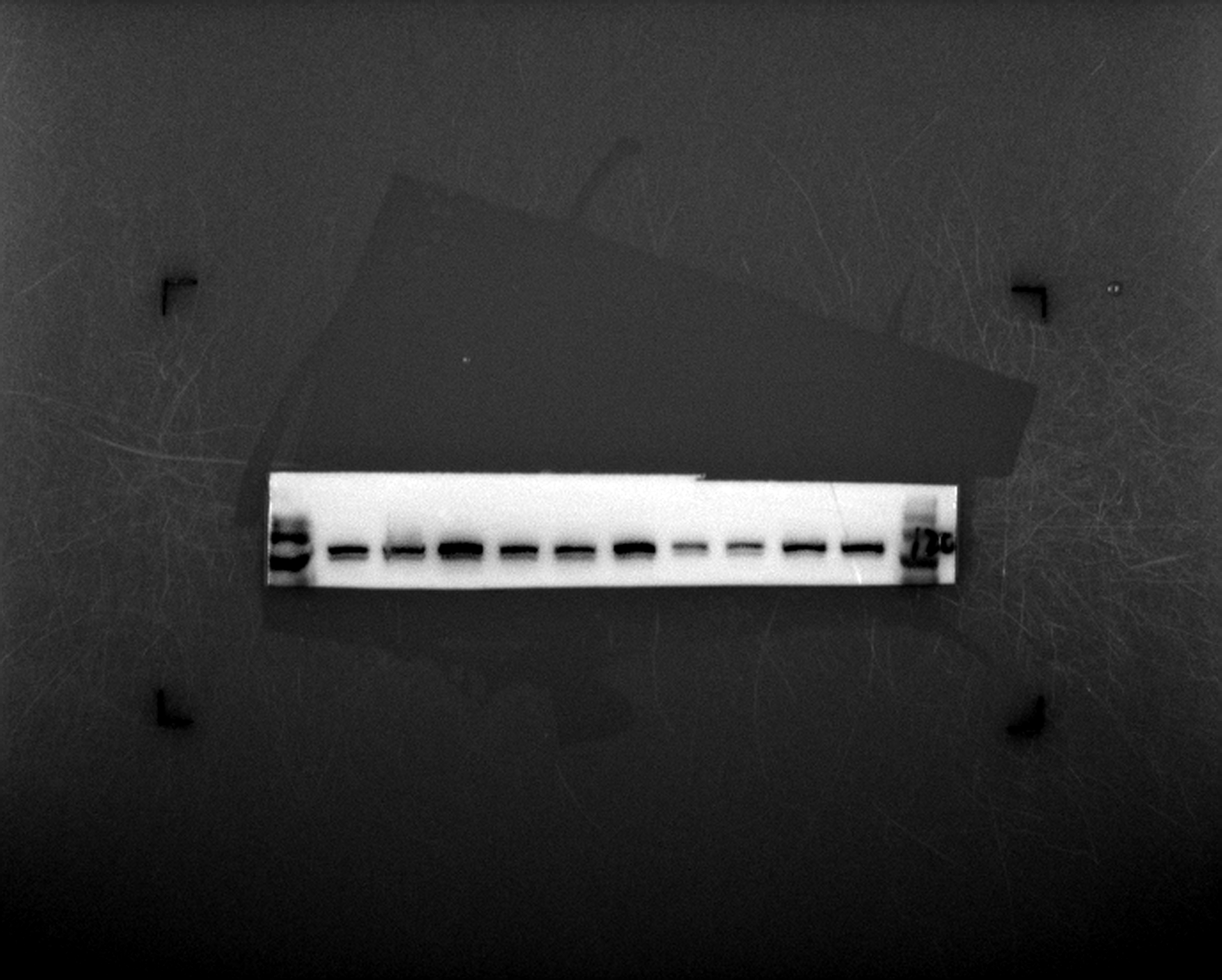

Supplement: Figure 5—source data 20. [file elife-96988-fig5-data20.zip › Figure 5-source data 20/1/SND1.tif]

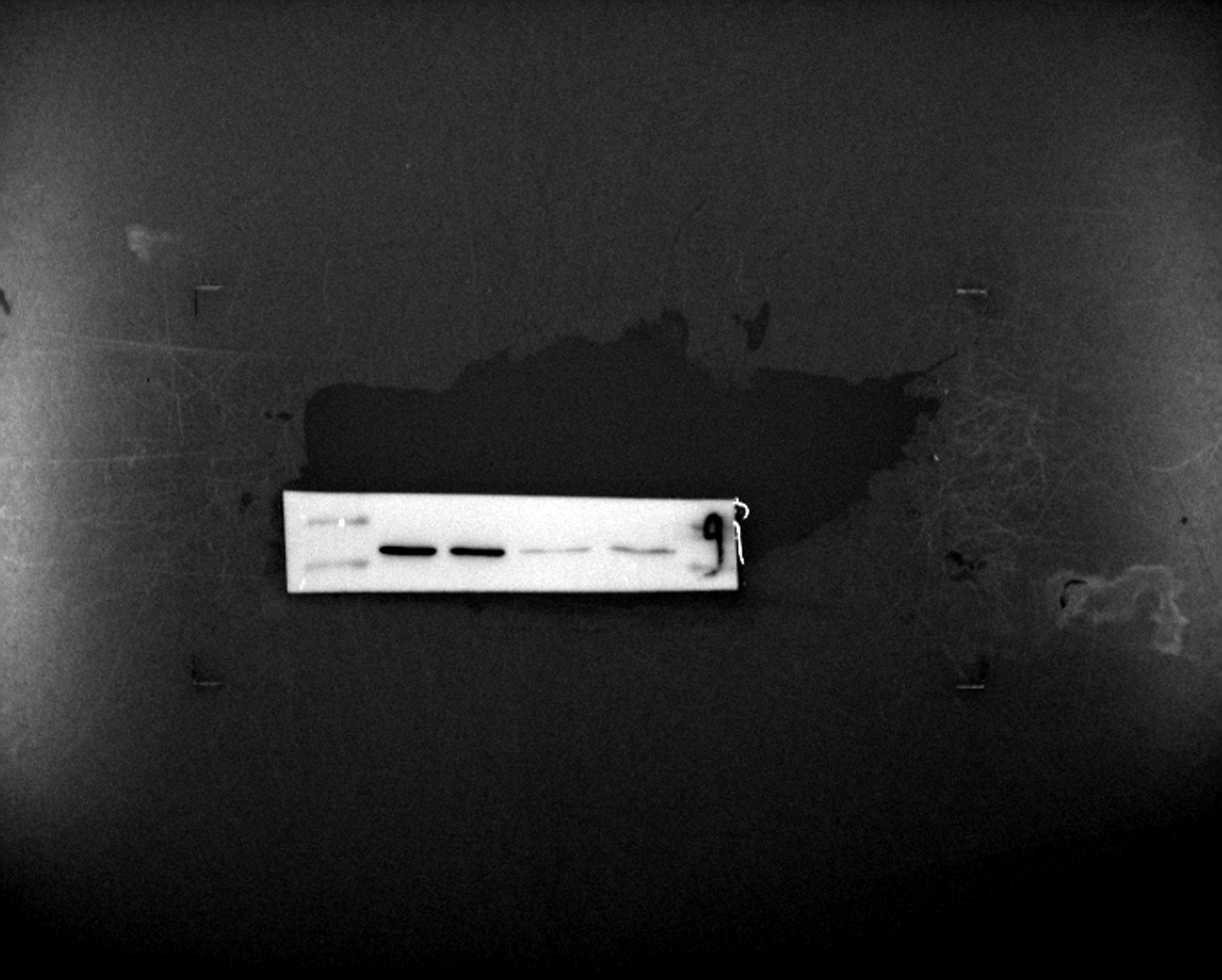

Supplement: Figure 5—source data 20. [file elife-96988-fig5-data20.zip › Figure 5-source data 20/2/GAPDH.Tif]

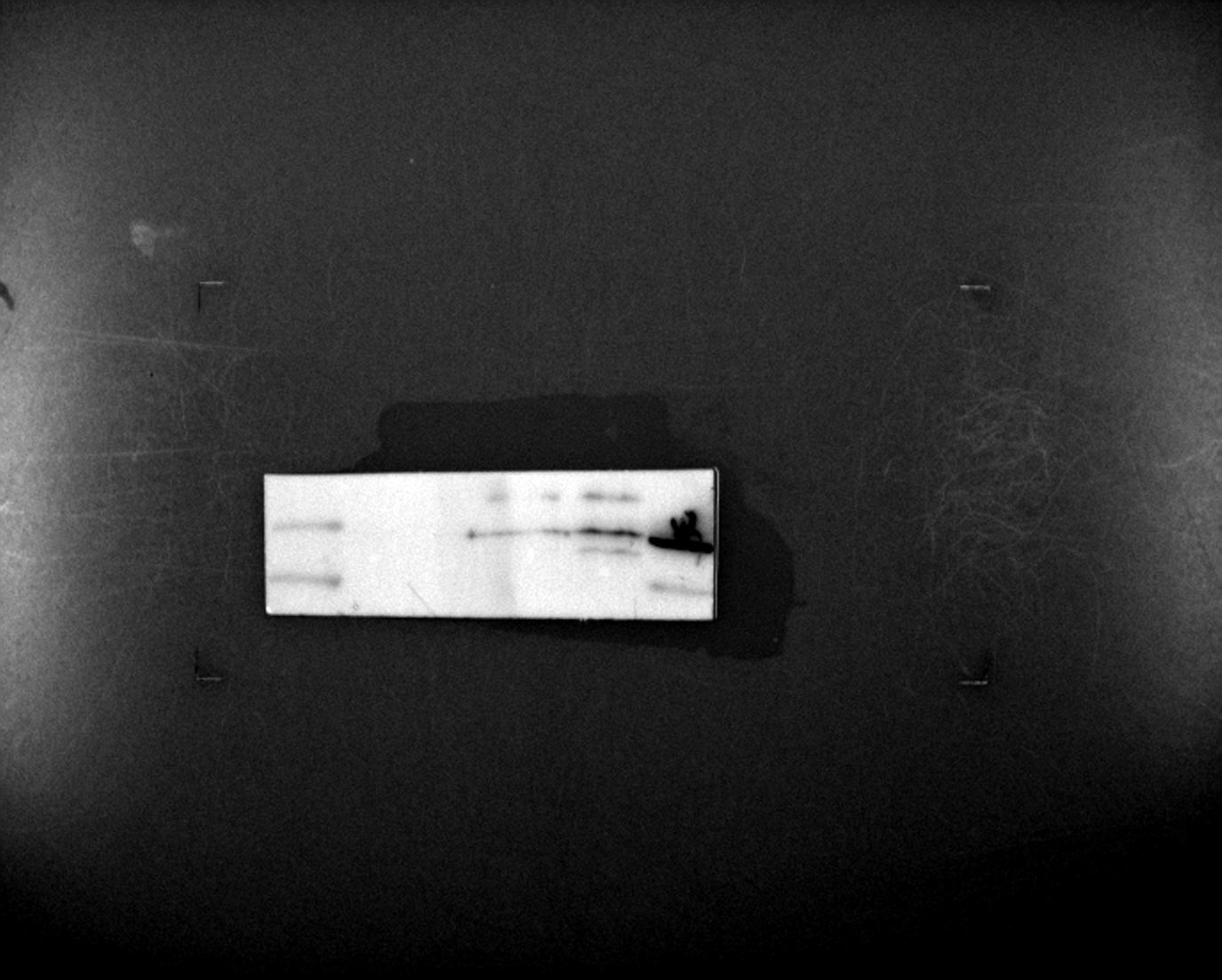

Supplement: Figure 5—source data 20. [file elife-96988-fig5-data20.zip › Figure 5-source data 20/2/H3.Tif]

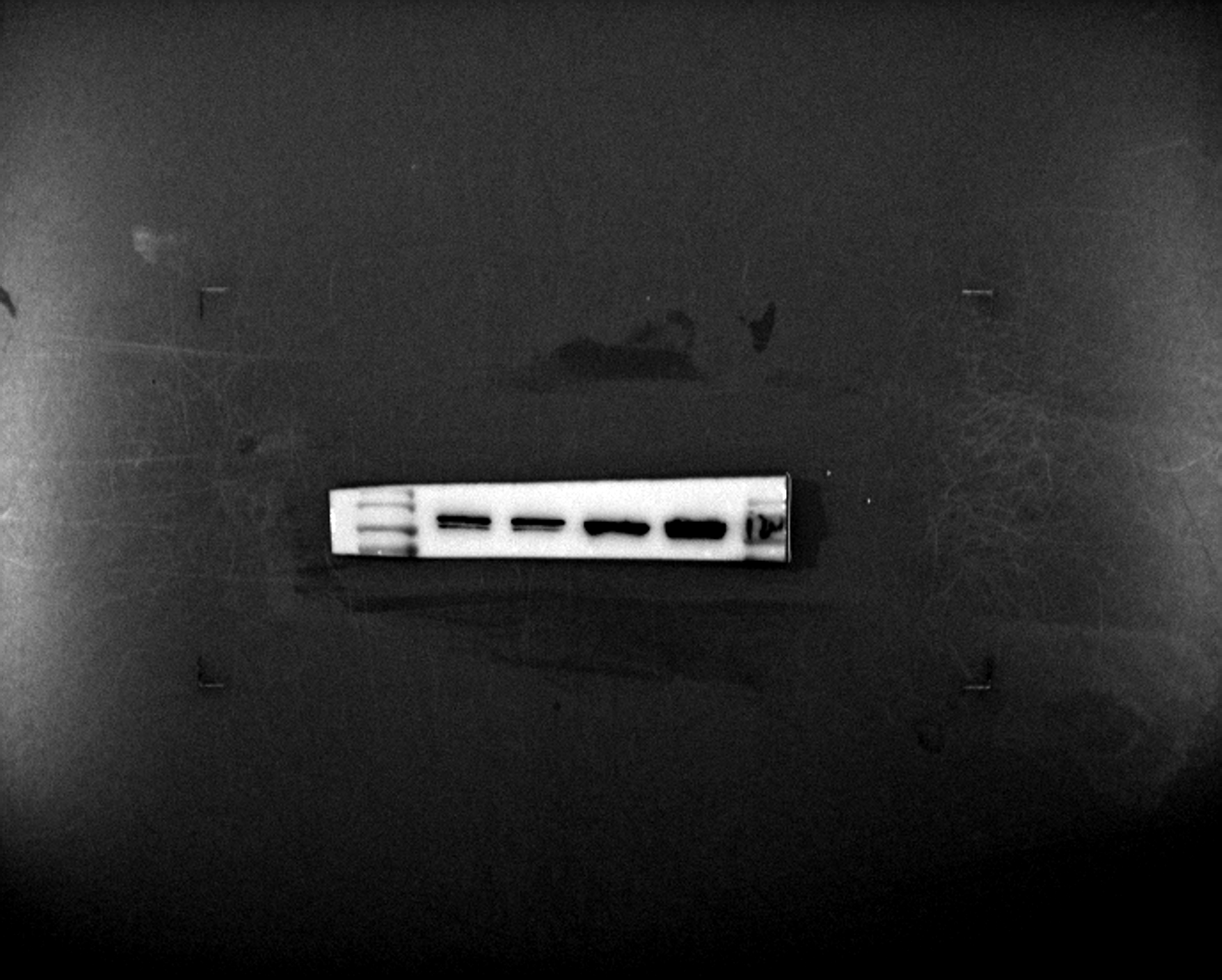

Supplement: Figure 5—source data 20. [file elife-96988-fig5-data20.zip › Figure 5-source data 20/2/SND1.Tif]

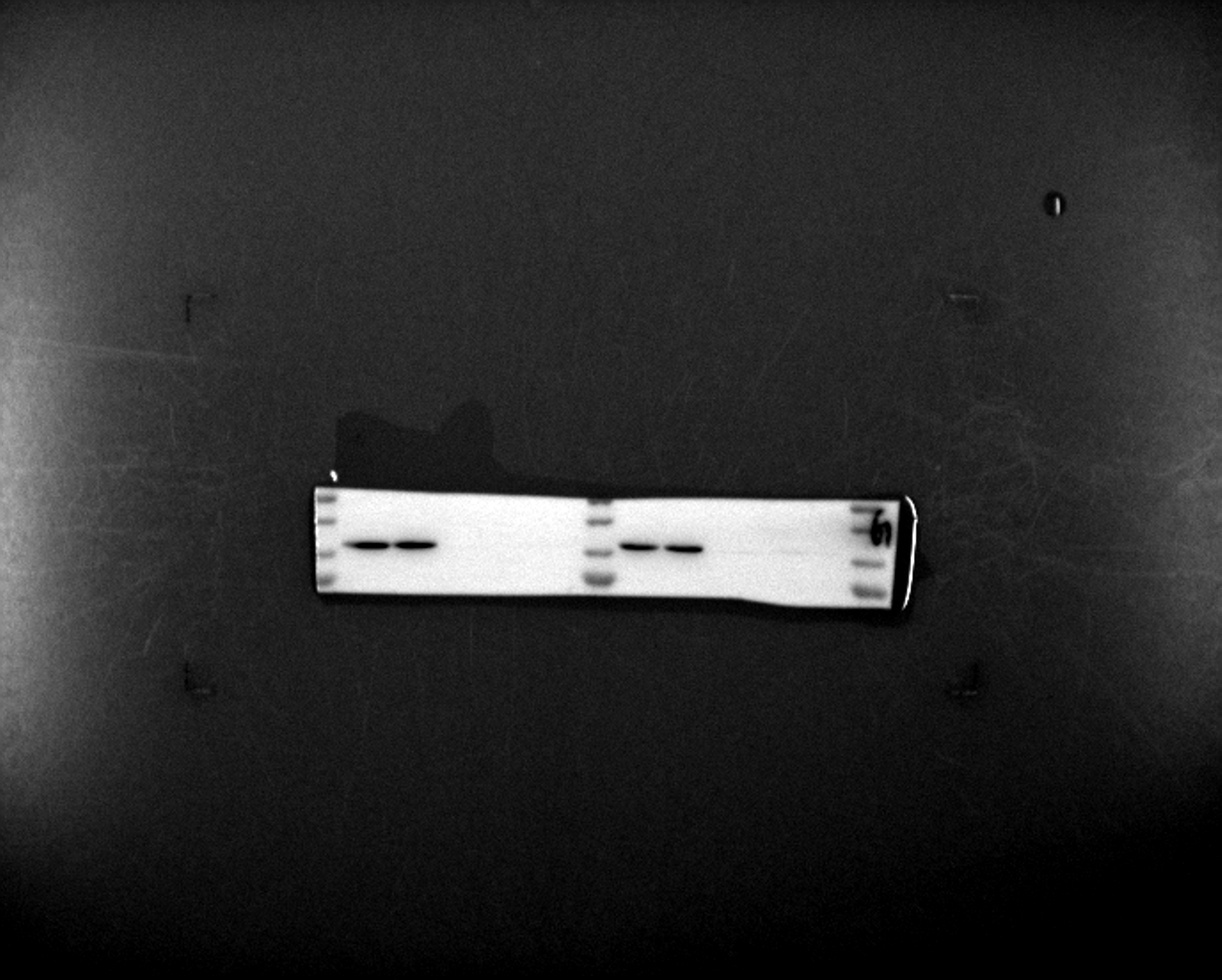

Supplement: Figure 5—source data 20. [file elife-96988-fig5-data20.zip › Figure 5-source data 20/3/GAPDH.Tif]

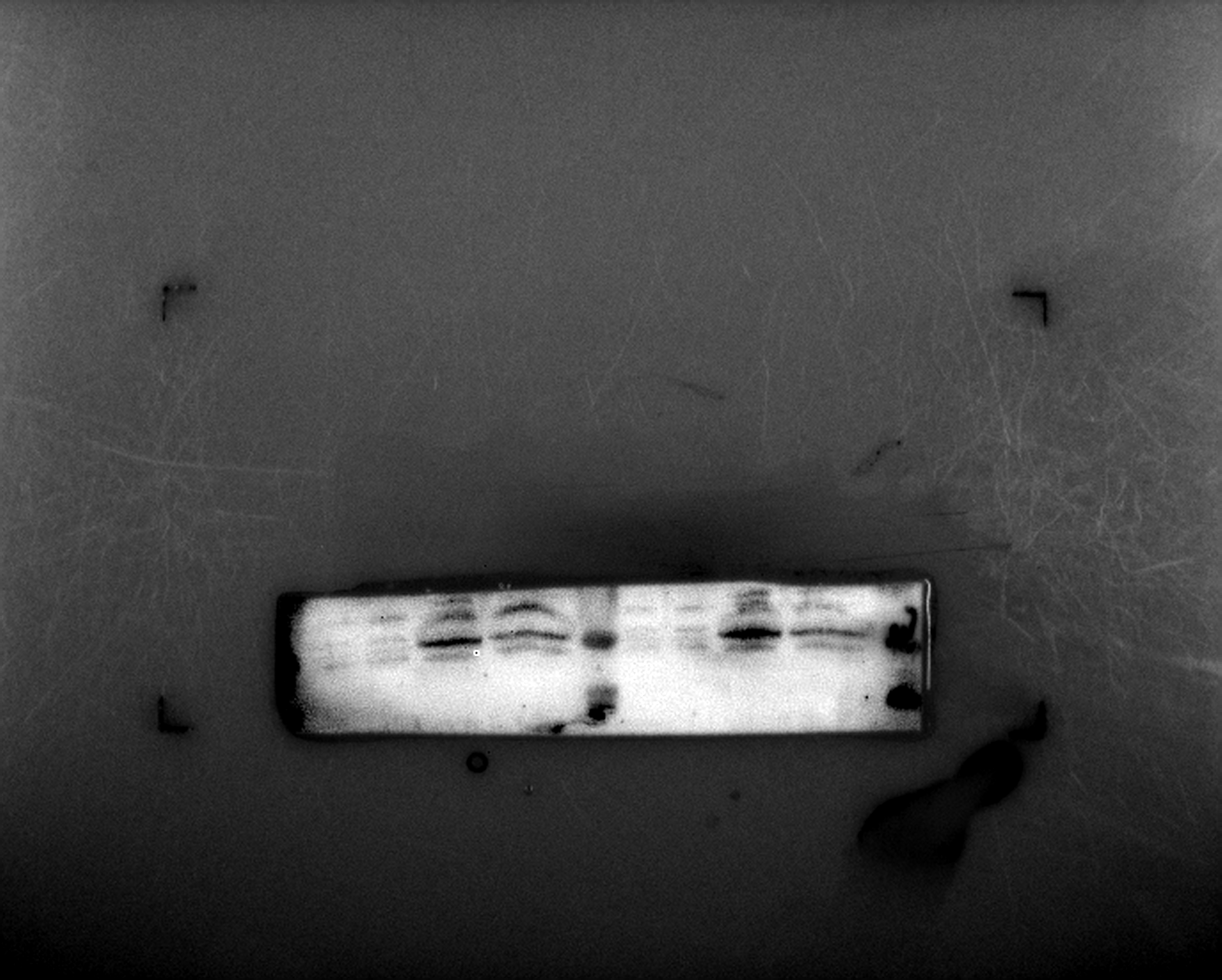

Supplement: Figure 5—source data 20. [file elife-96988-fig5-data20.zip › Figure 5-source data 20/3/H3.tif]

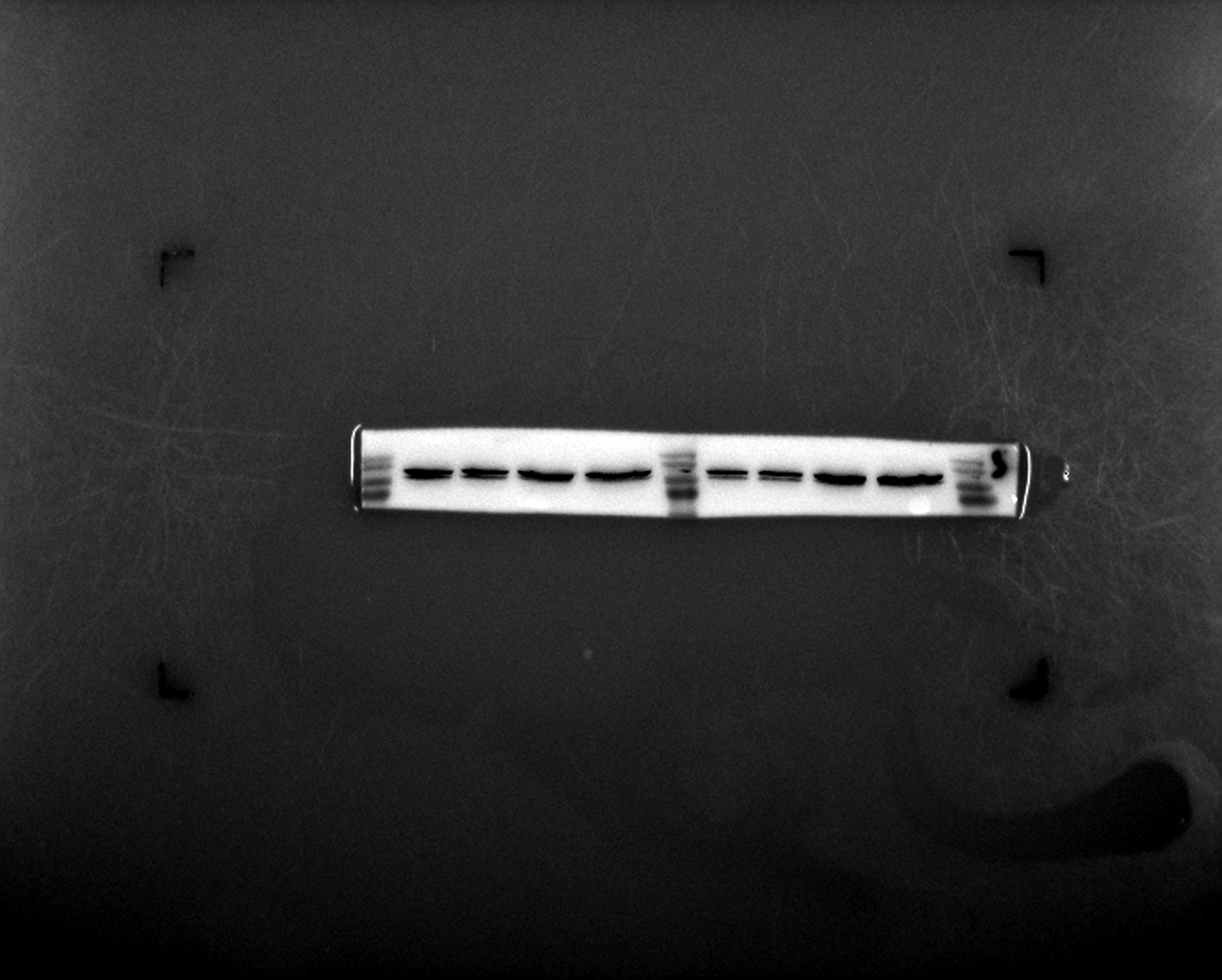

Supplement: Figure 5—source data 20. [file elife-96988-fig5-data20.zip › Figure 5-source data 20/3/SND1.tif]

Figure 6A

1.

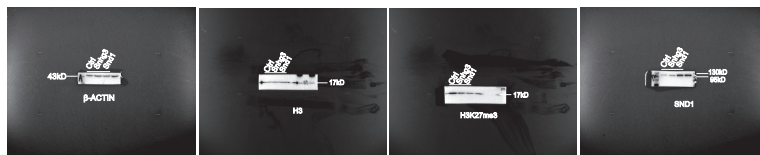

2.

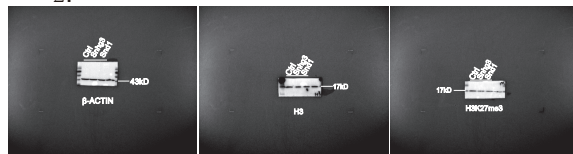

3.

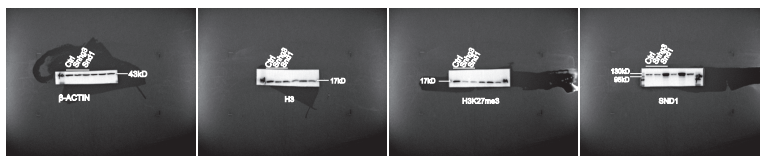

4.

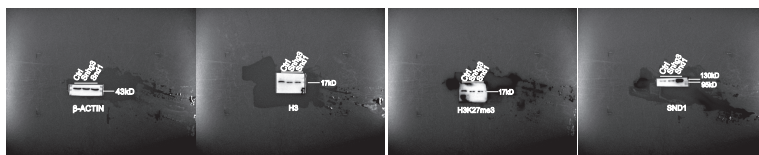

Supplement: Figure 6—source data 1. [file elife-96988-fig6-data1.pdf]

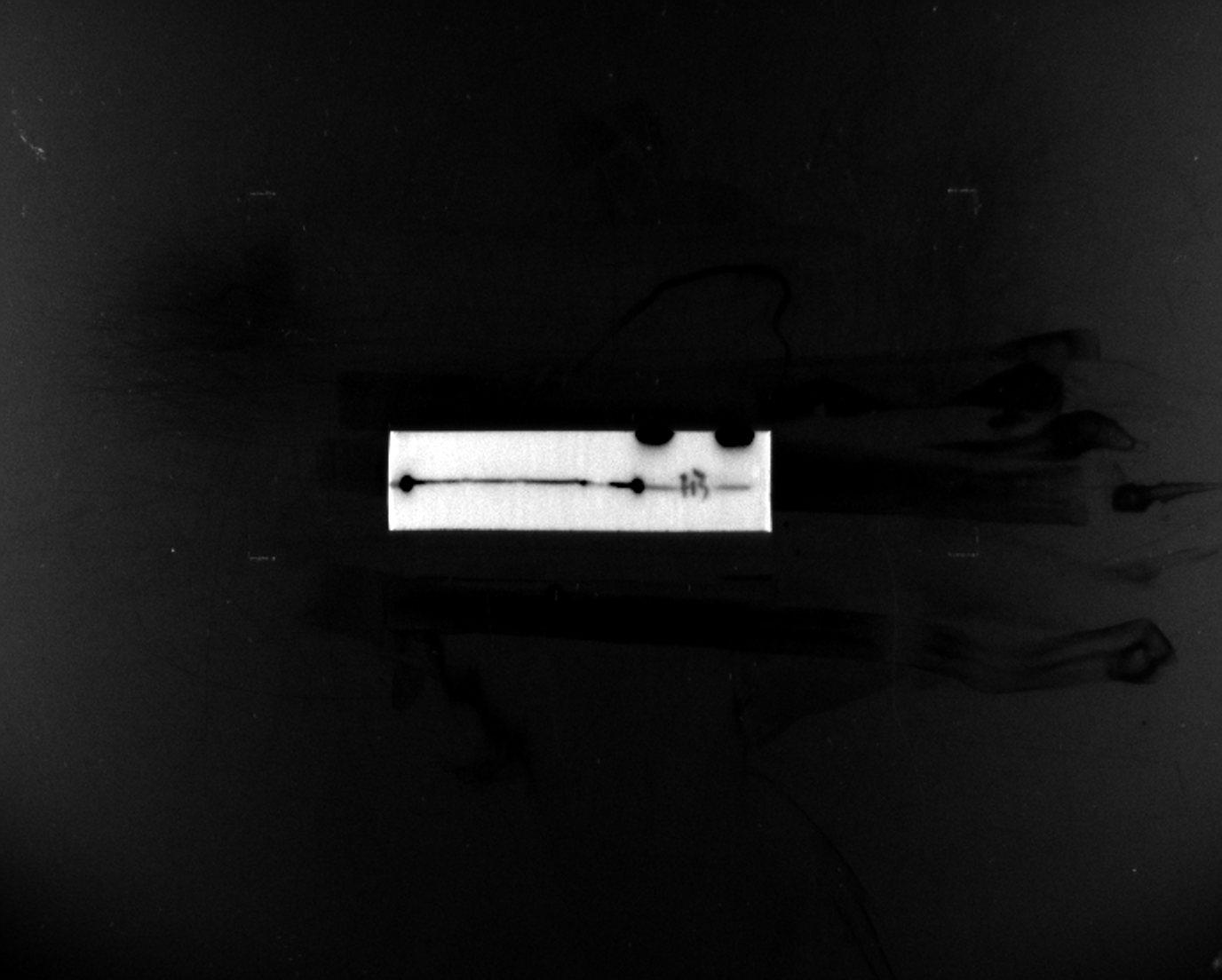

Supplement: Figure 6—source data 2. [file elife-96988-fig6-data2.zip › Figure 6-source data 2/1/H3.tif]

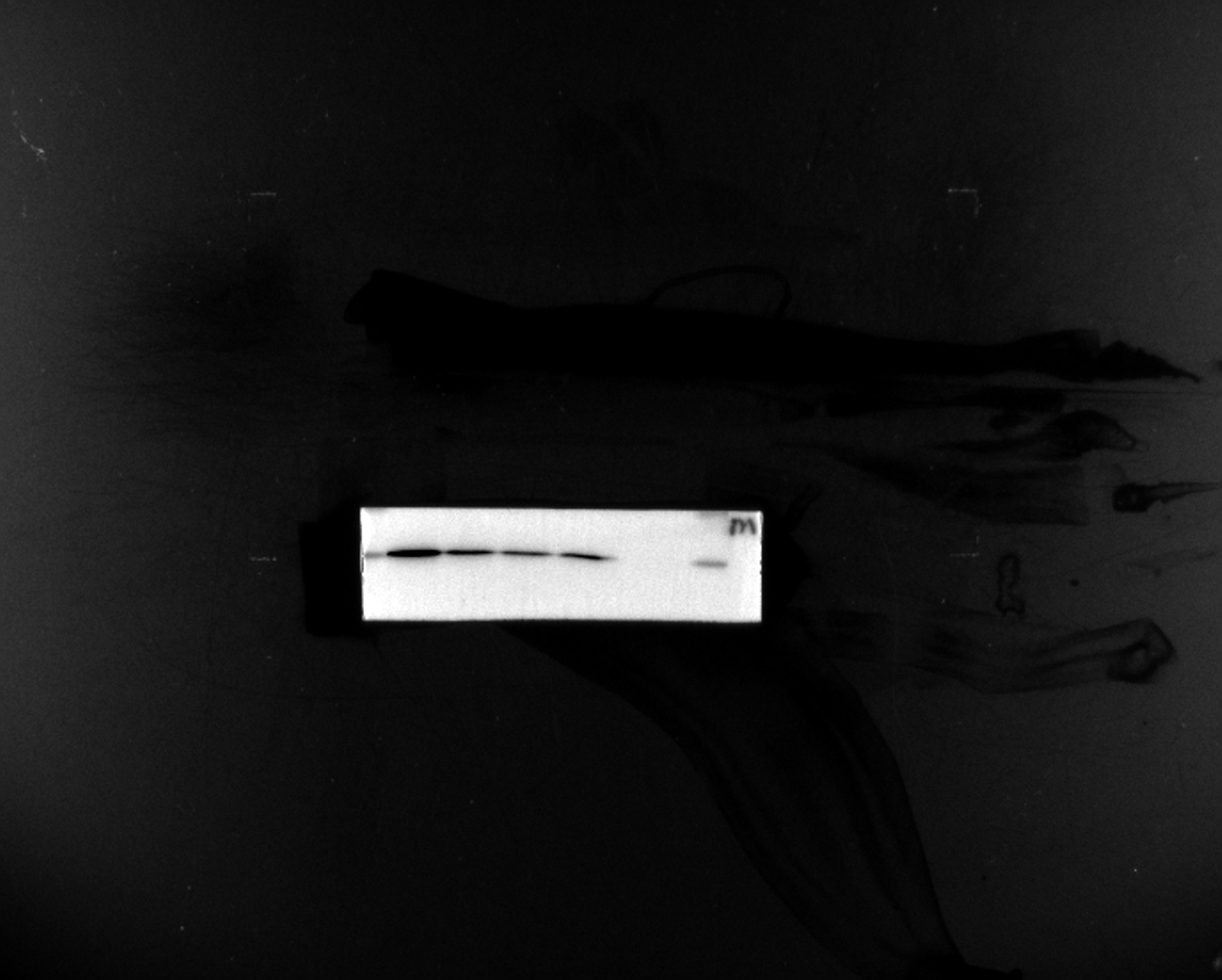

Supplement: Figure 6—source data 2. [file elife-96988-fig6-data2.zip › Figure 6-source data 2/1/H3K27me3.tif]

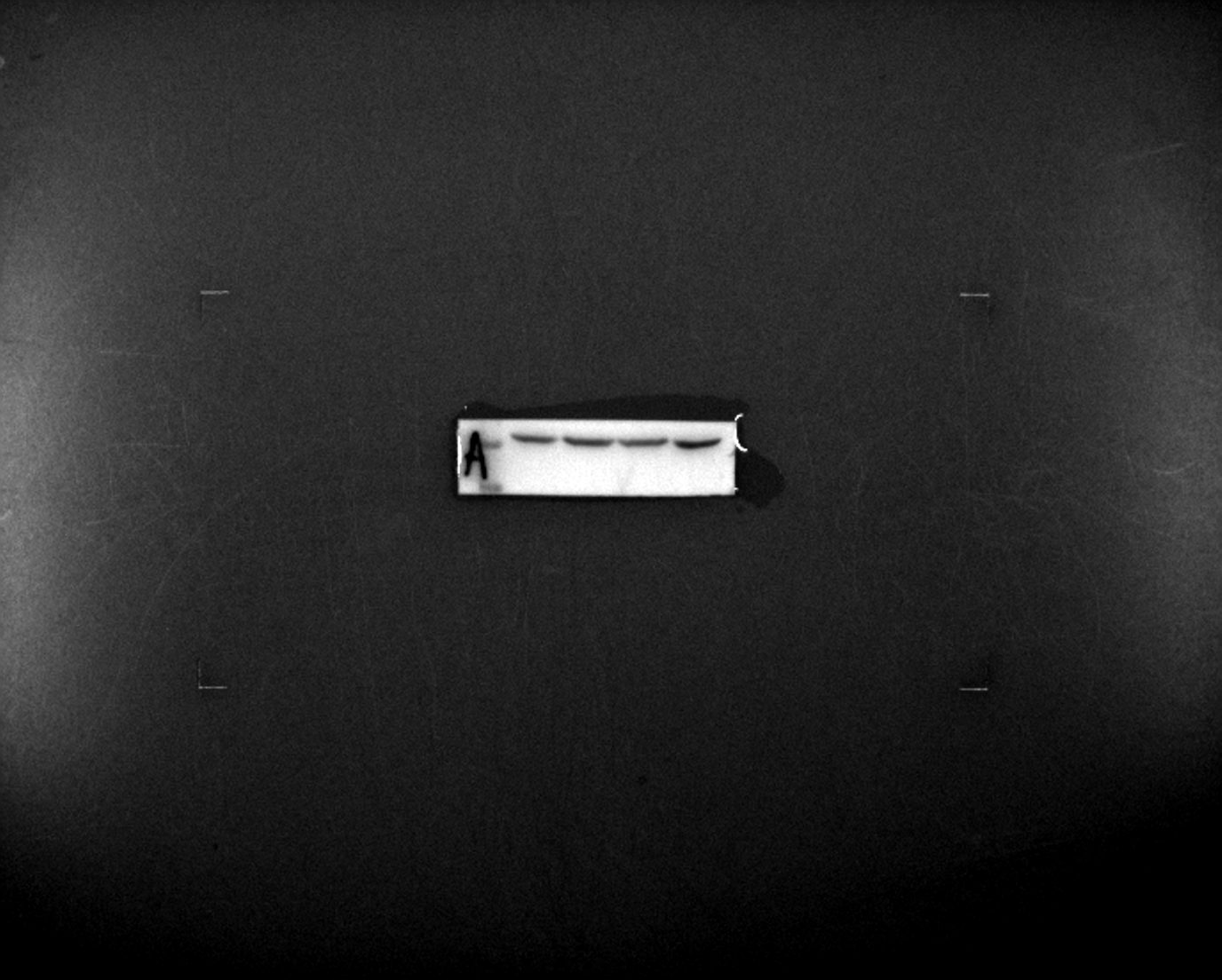

Supplement: Figure 6—source data 2. [file elife-96988-fig6-data2.zip › Figure 6-source data 2/1/β-ACTIN.Tif]

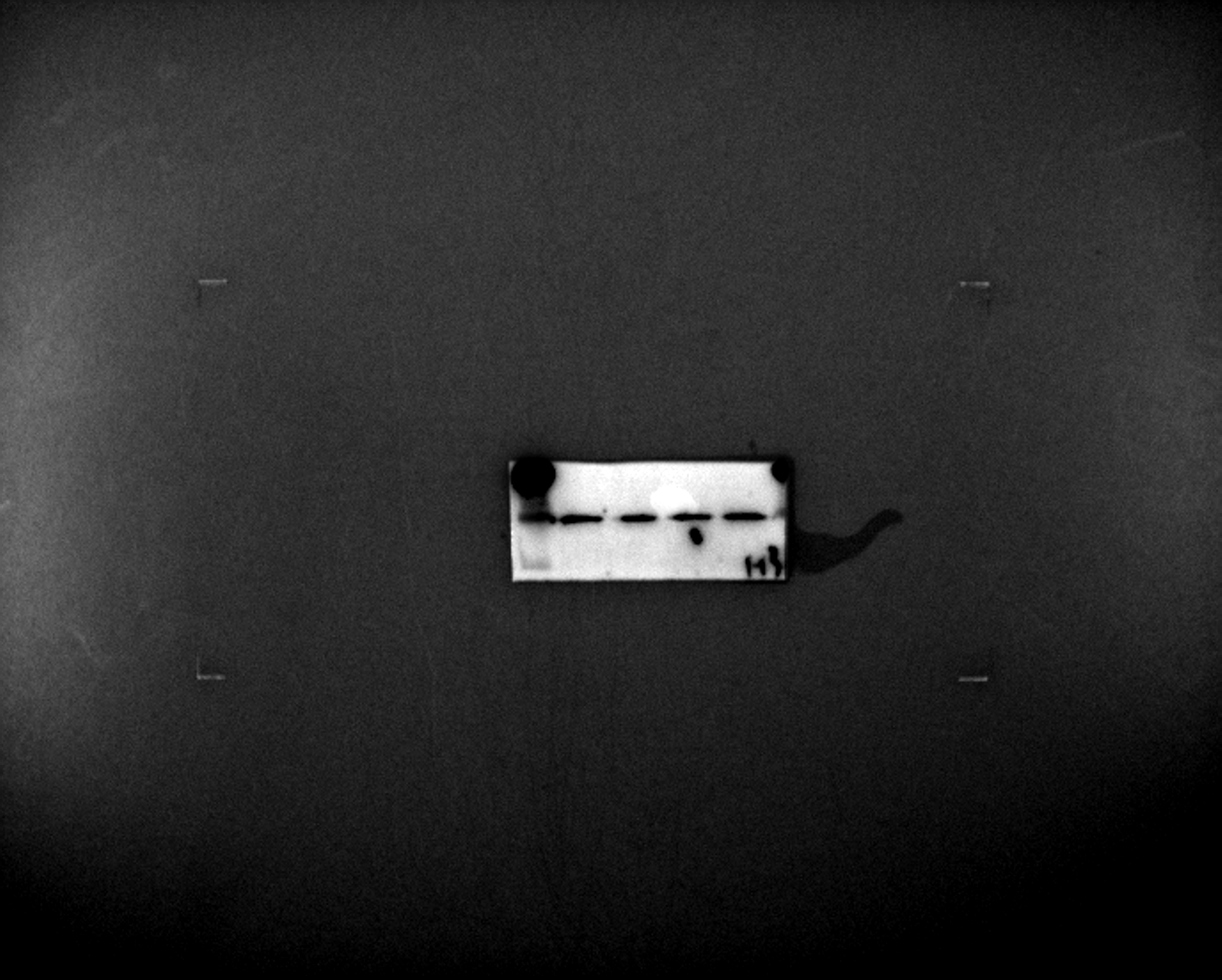

Supplement: Figure 6—source data 2. [file elife-96988-fig6-data2.zip › Figure 6-source data 2/2/H3.Tif]

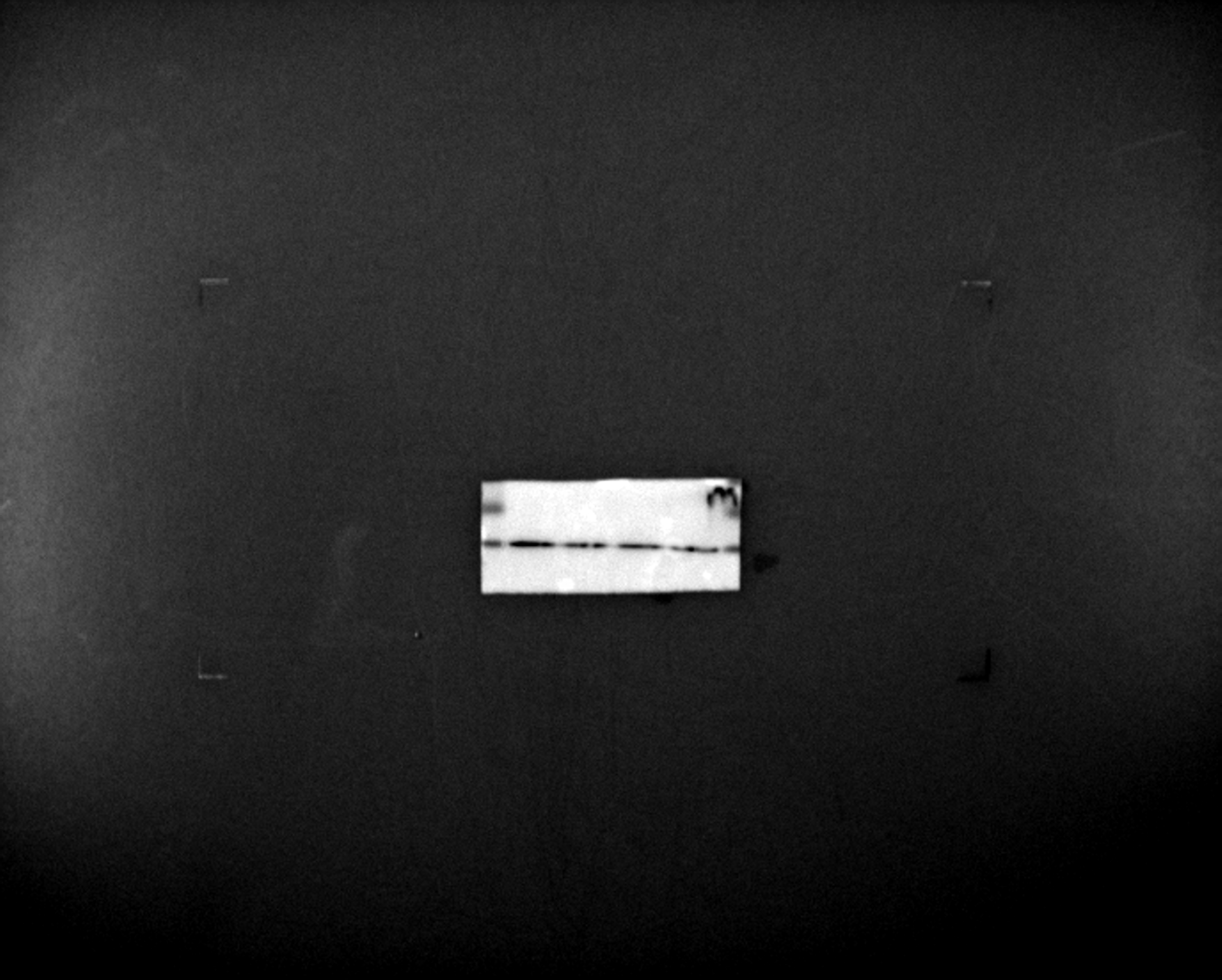

Supplement: Figure 6—source data 2. [file elife-96988-fig6-data2.zip › Figure 6-source data 2/2/H3K27me3.Tif]

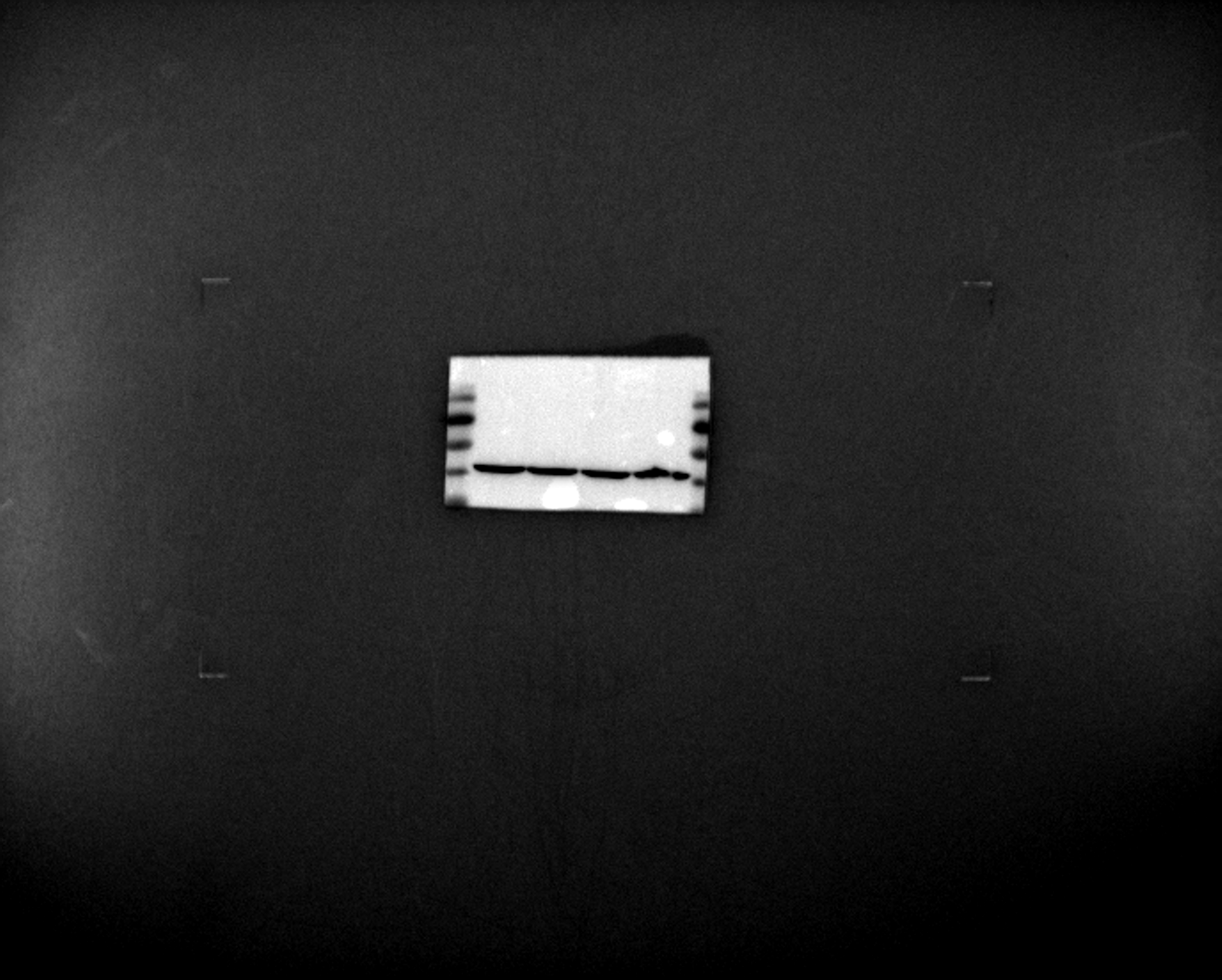

Supplement: Figure 6—source data 2. [file elife-96988-fig6-data2.zip › Figure 6-source data 2/2/β-ACTIN.Tif]

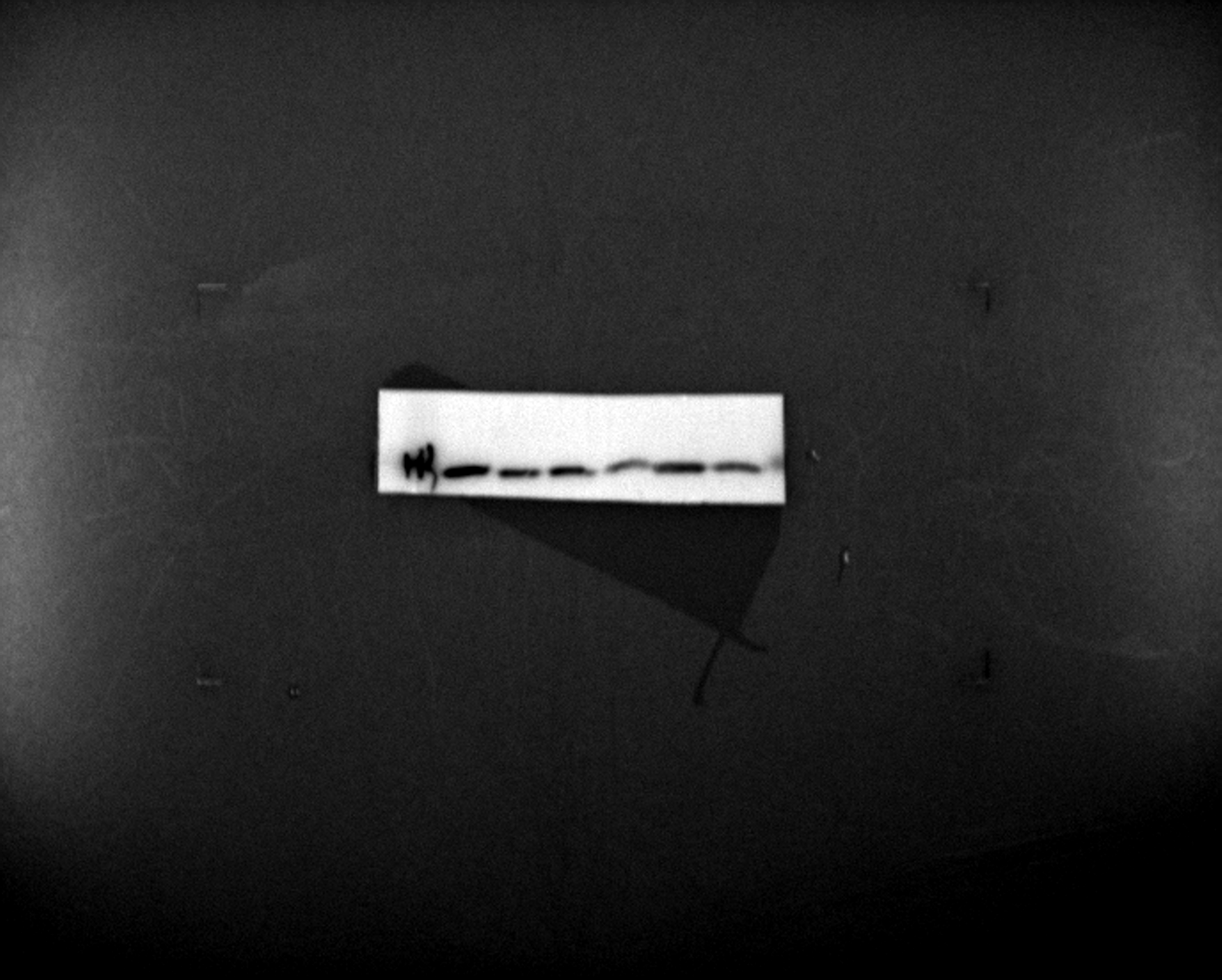

Supplement: Figure 6—source data 2. [file elife-96988-fig6-data2.zip › Figure 6-source data 2/3/H3.Tif]

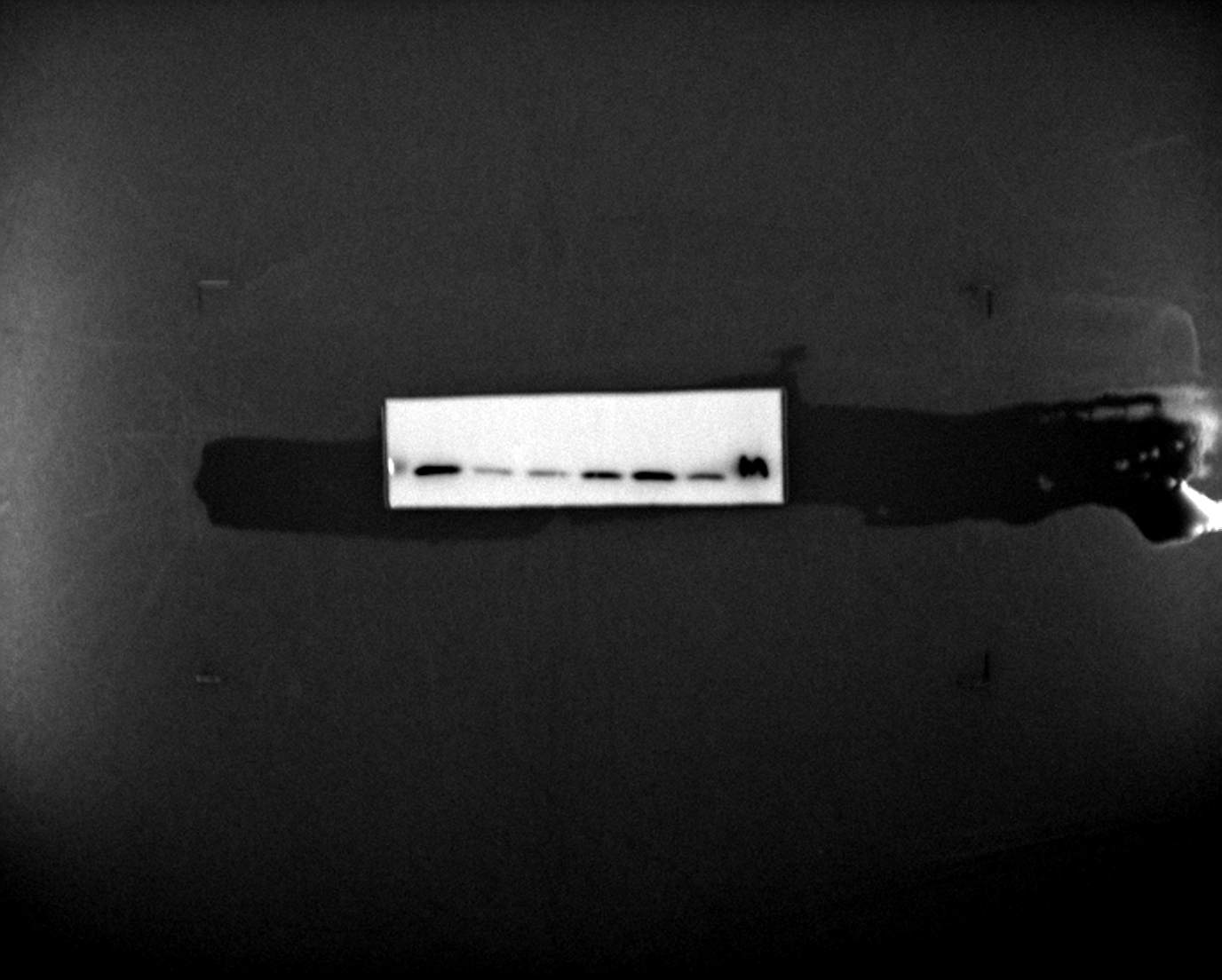

Supplement: Figure 6—source data 2. [file elife-96988-fig6-data2.zip › Figure 6-source data 2/3/H3K27.Tif]

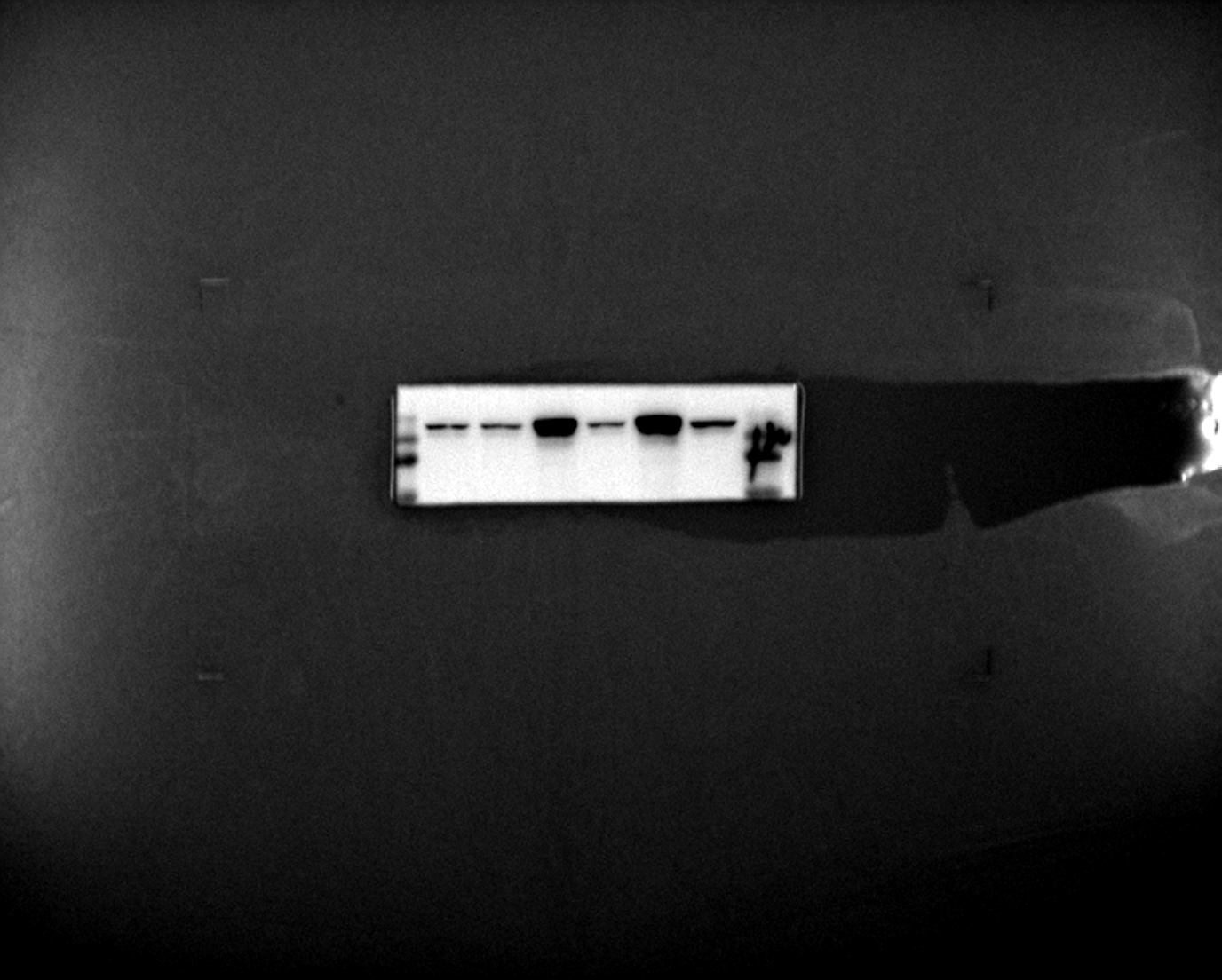

Supplement: Figure 6—source data 2. [file elife-96988-fig6-data2.zip › Figure 6-source data 2/3/SND1.Tif]

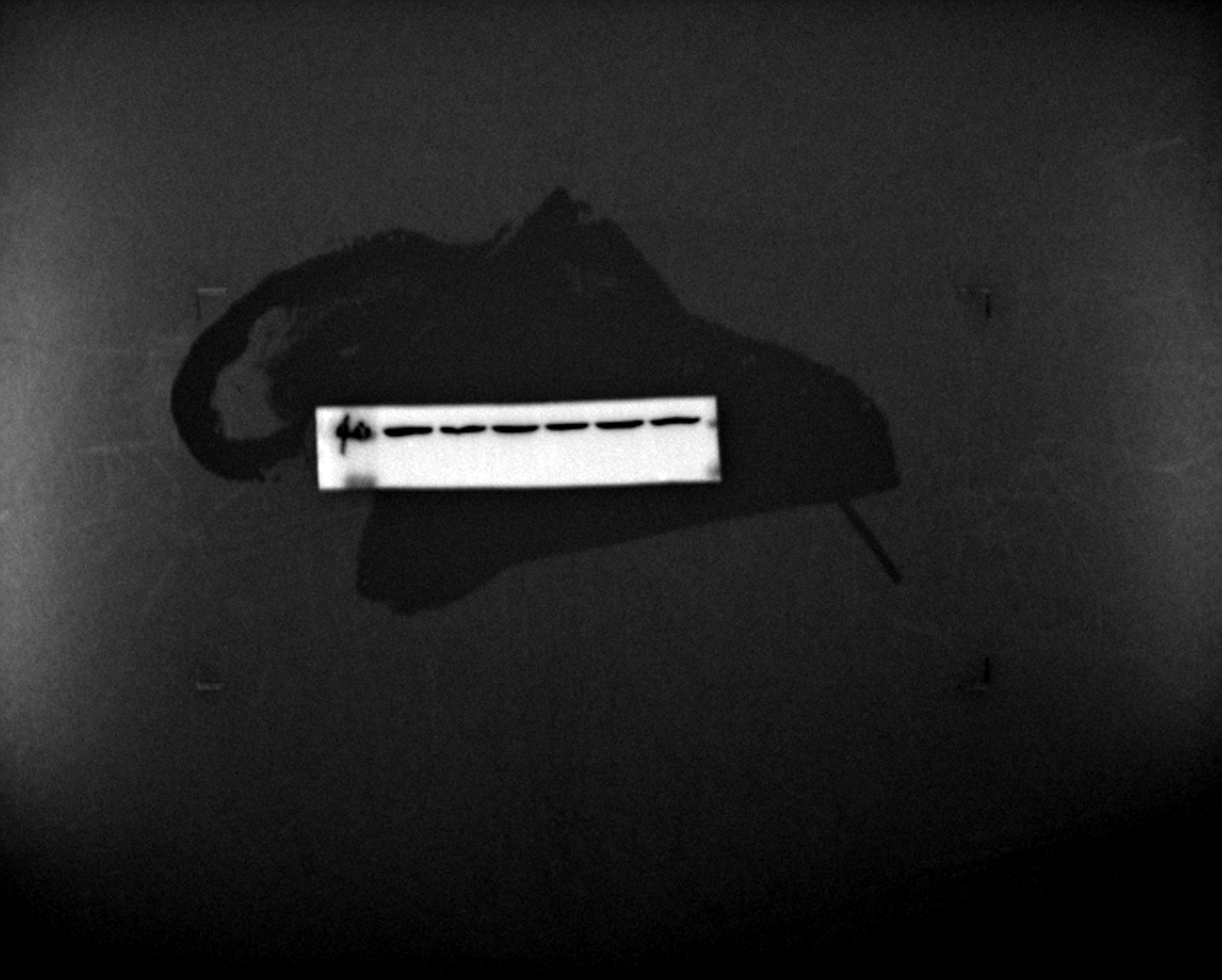

Supplement: Figure 6—source data 2. [file elife-96988-fig6-data2.zip › Figure 6-source data 2/3/β-ACTIN.Tif]

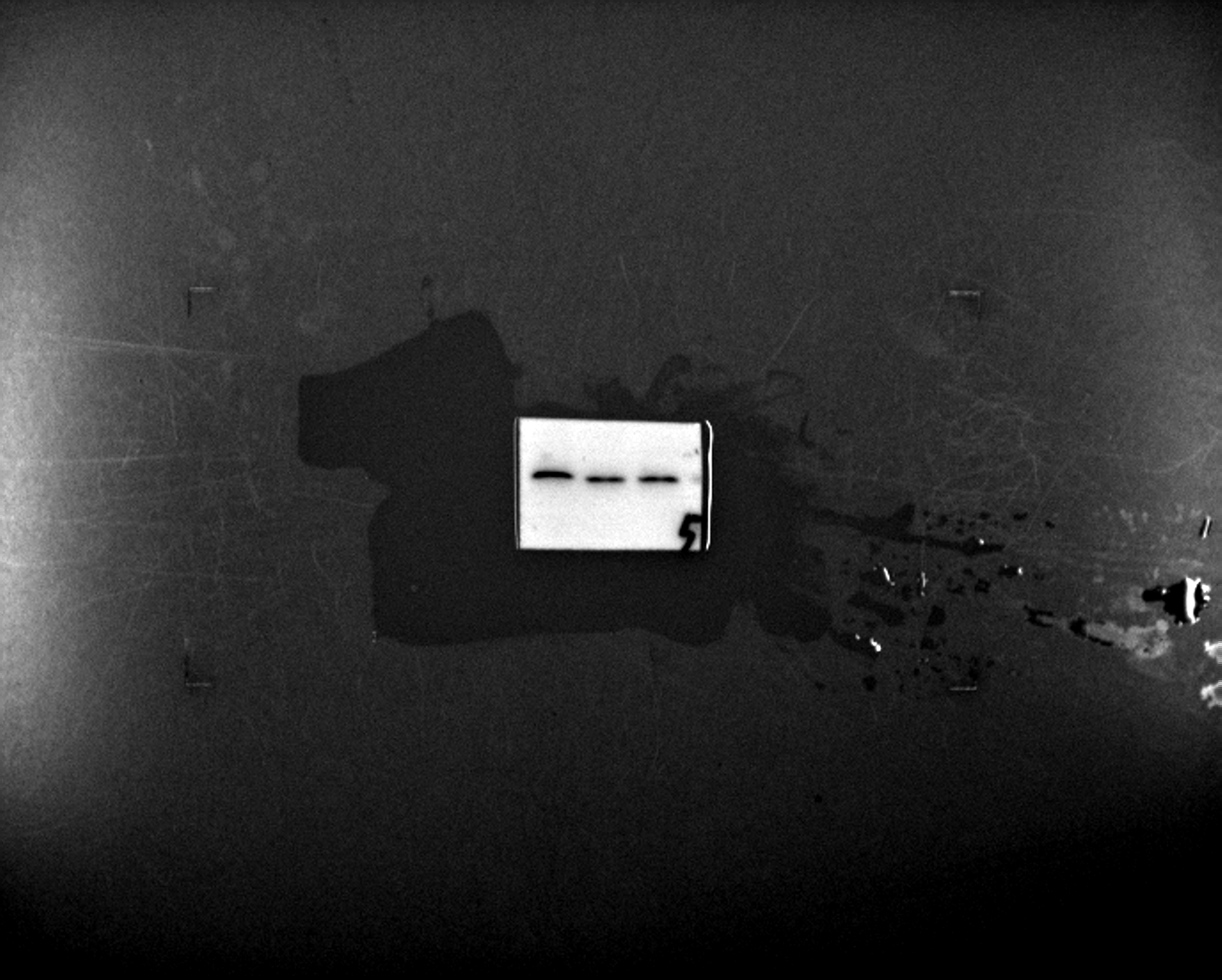

Supplement: Figure 6—source data 2. [file elife-96988-fig6-data2.zip › Figure 6-source data 2/4/H3.Tif]

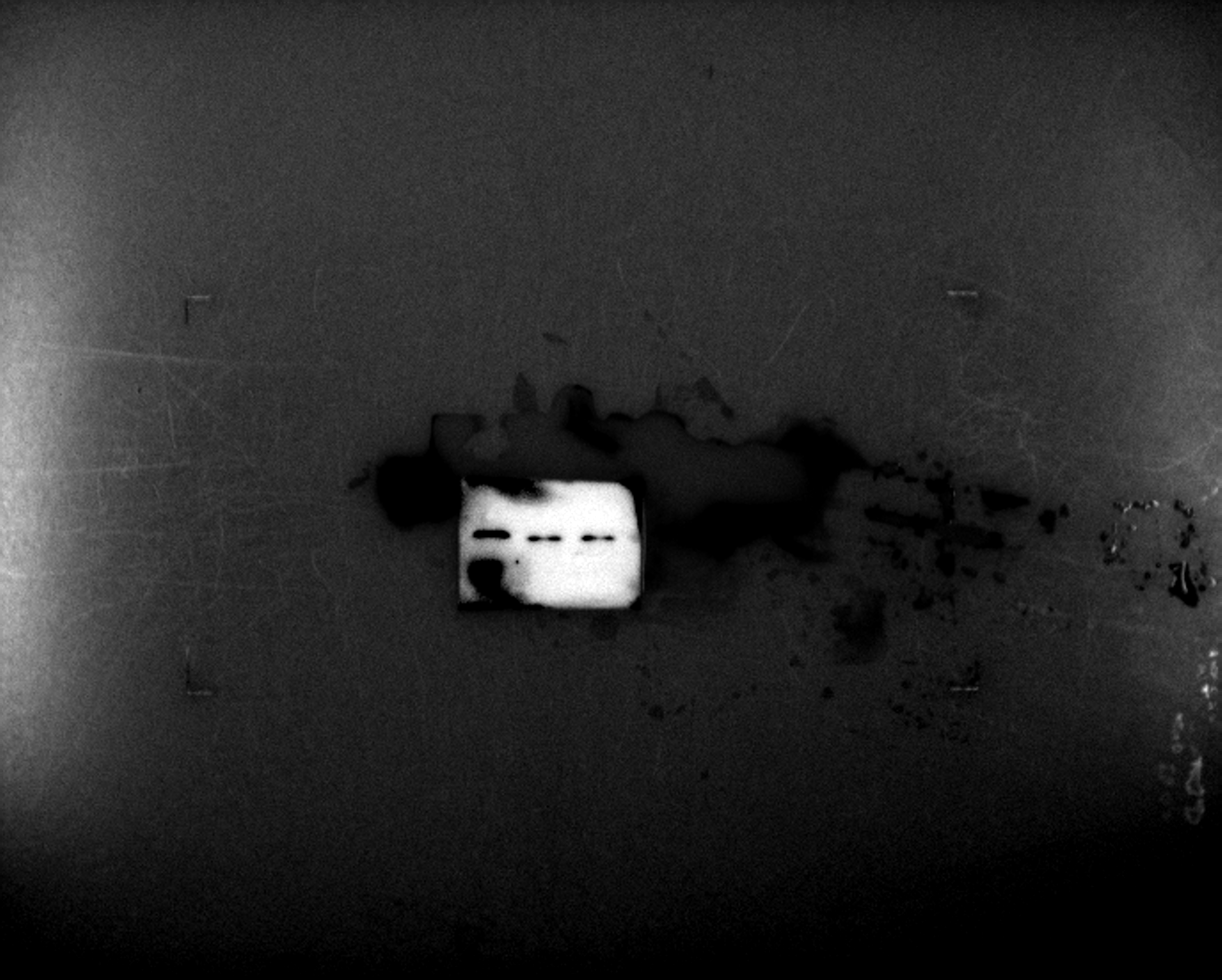

Supplement: Figure 6—source data 2. [file elife-96988-fig6-data2.zip › Figure 6-source data 2/4/H3K27me3.Tif]

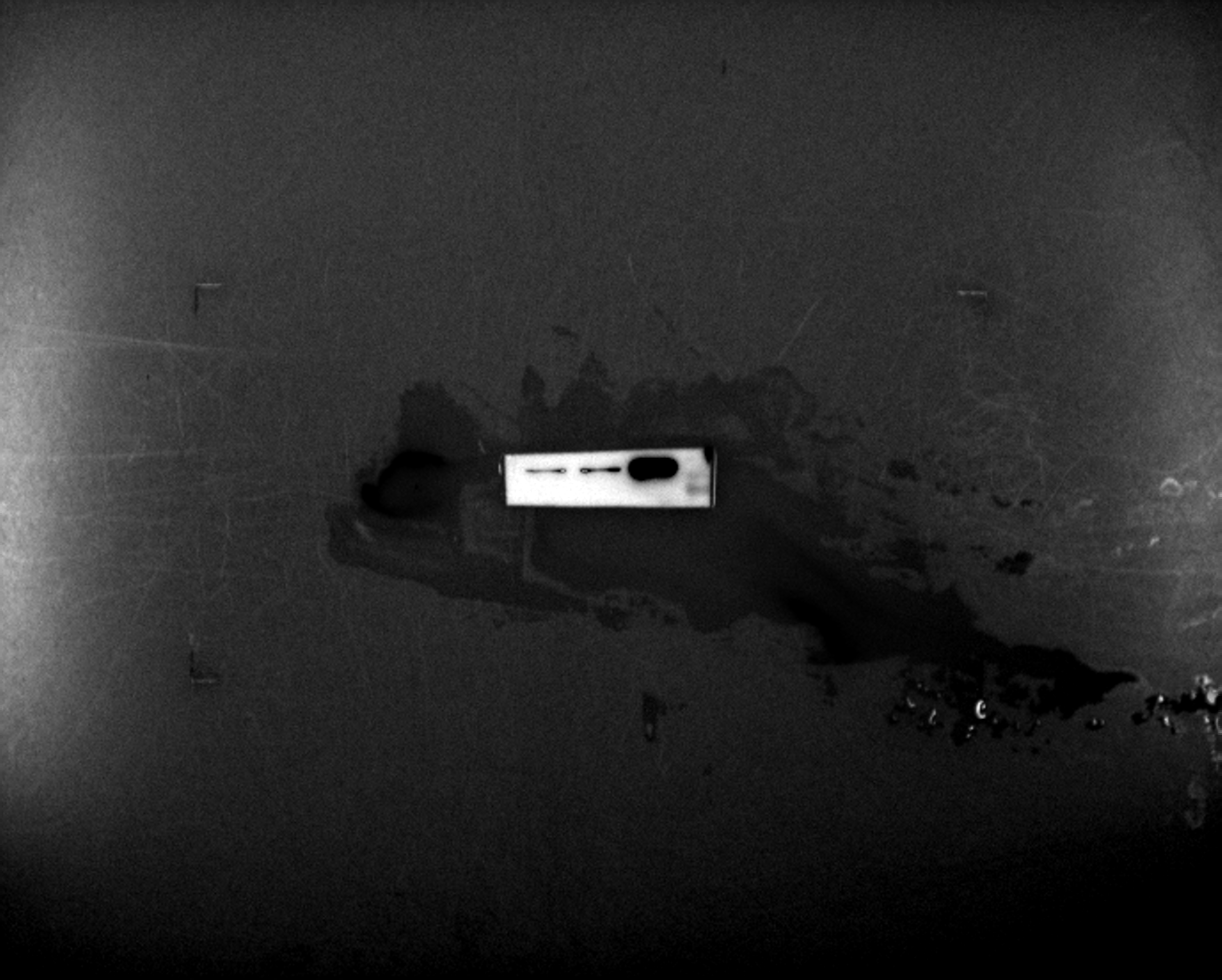

Supplement: Figure 6—source data 2. [file elife-96988-fig6-data2.zip › Figure 6-source data 2/4/SND1-merge.Tif]

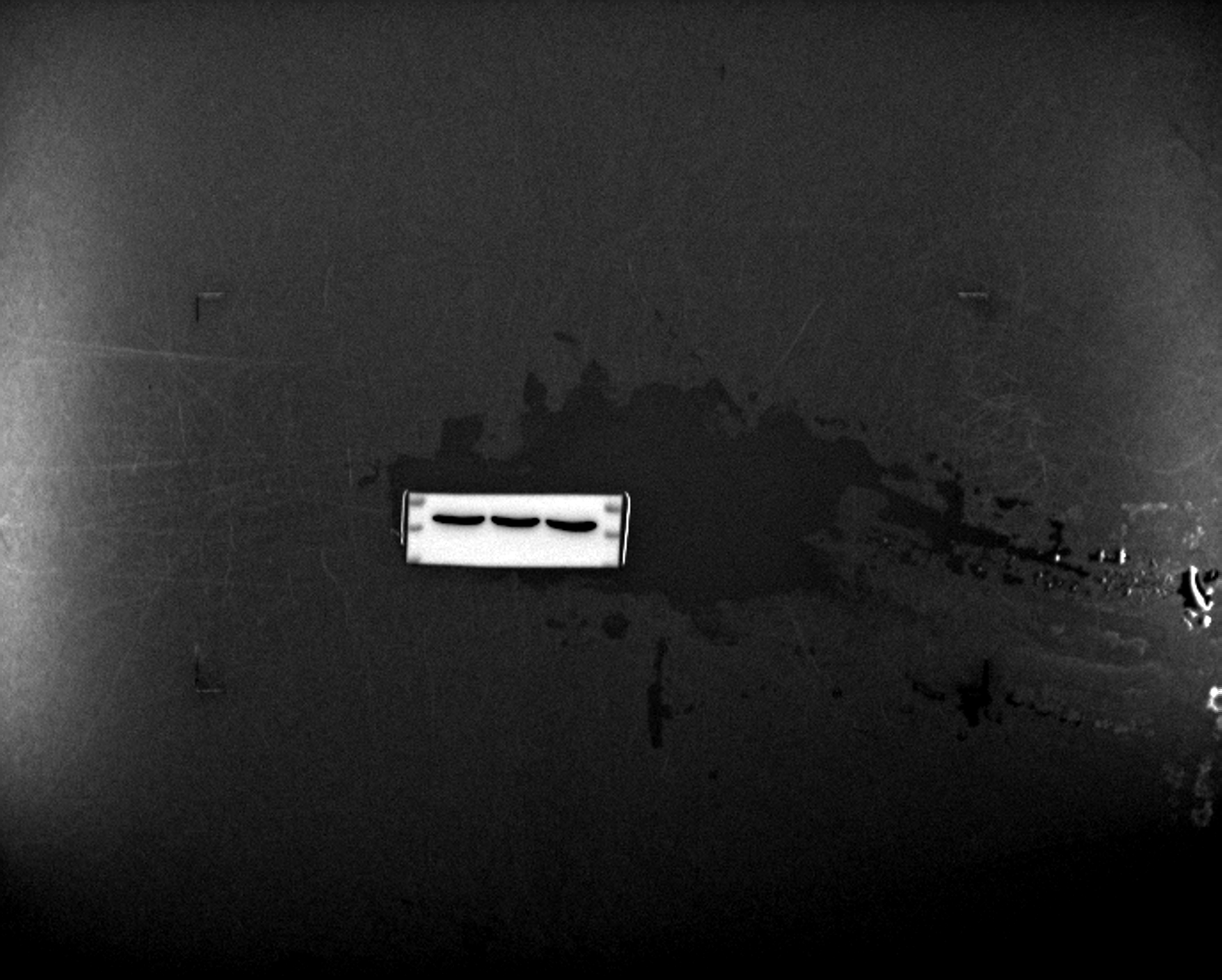

Supplement: Figure 6—source data 2. [file elife-96988-fig6-data2.zip › Figure 6-source data 2/4/β-ACTIN-merge.Tif]

Figure 6B

1.

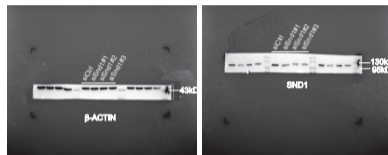

2.

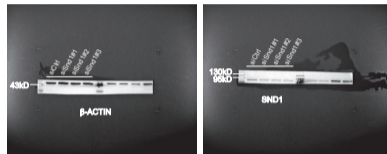

3.

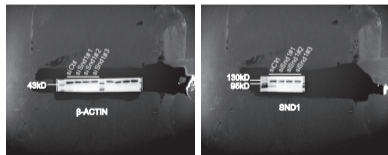

4.

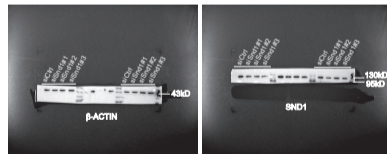

Supplement: Figure 6—source data 3. [file elife-96988-fig6-data3.pdf]

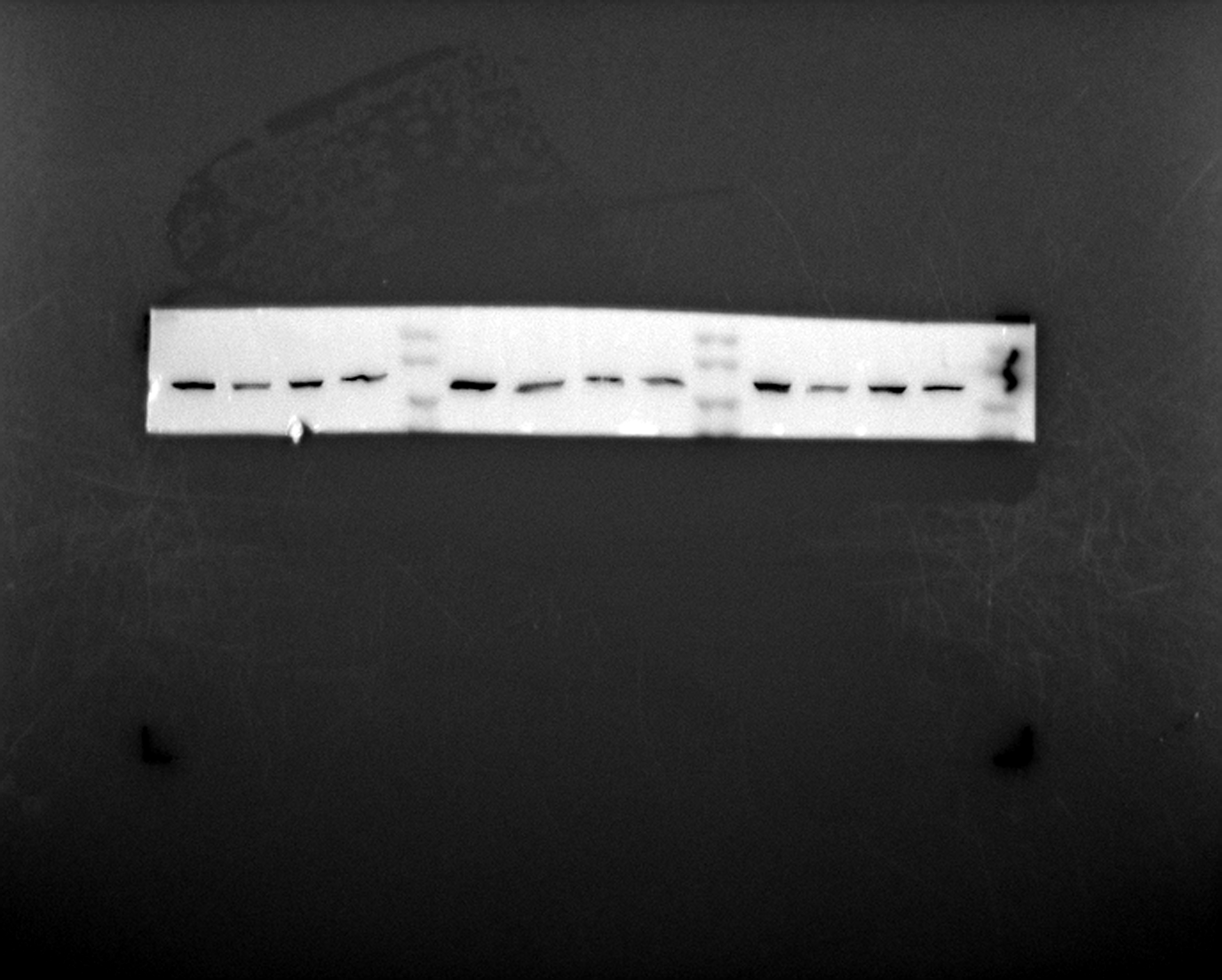

Supplement: Figure 6—source data 4. [file elife-96988-fig6-data4.zip › Figure 6-source data 4/1/SND1.tif]

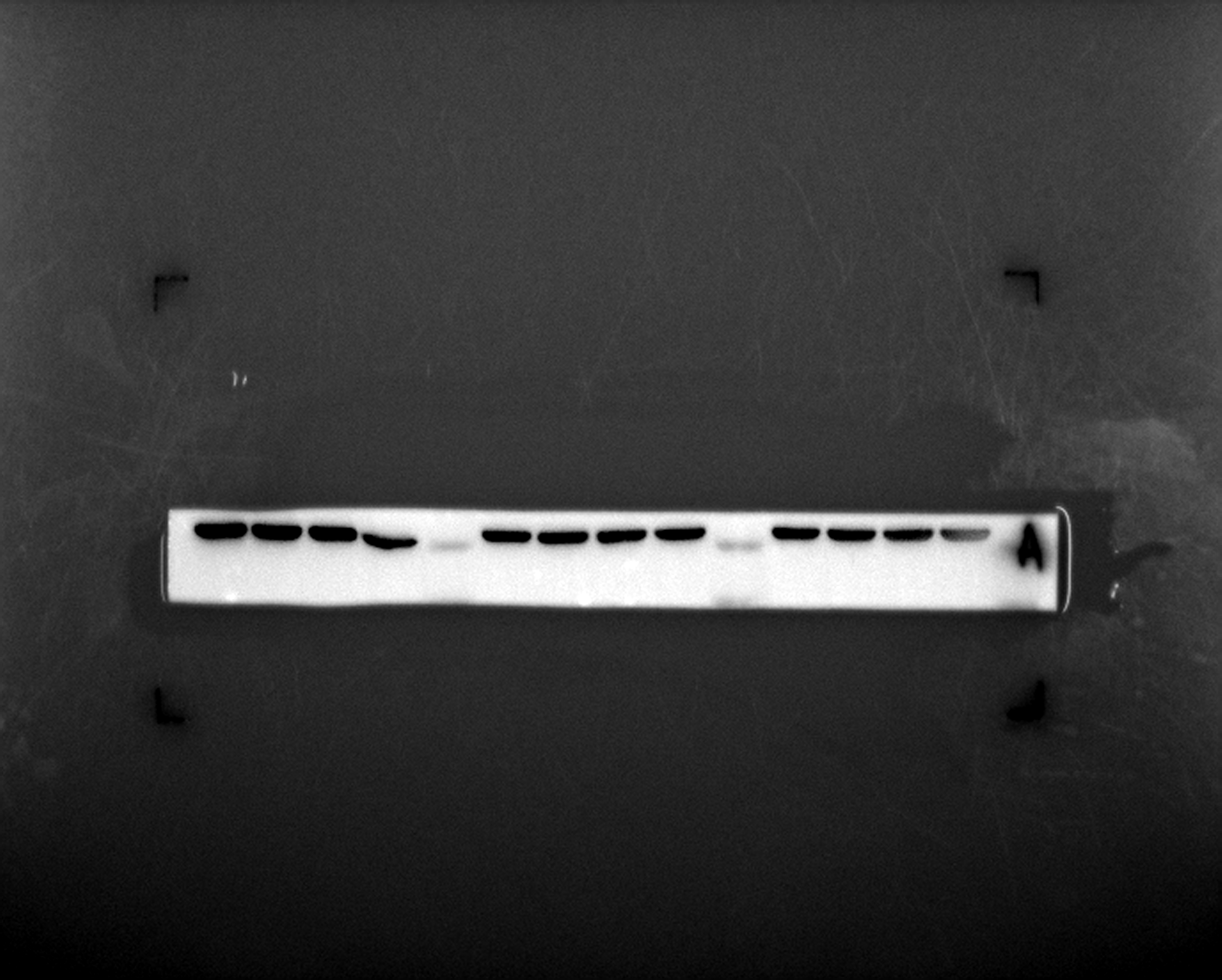

Supplement: Figure 6—source data 4. [file elife-96988-fig6-data4.zip › Figure 6-source data 4/1/β-ACTIN.tif]

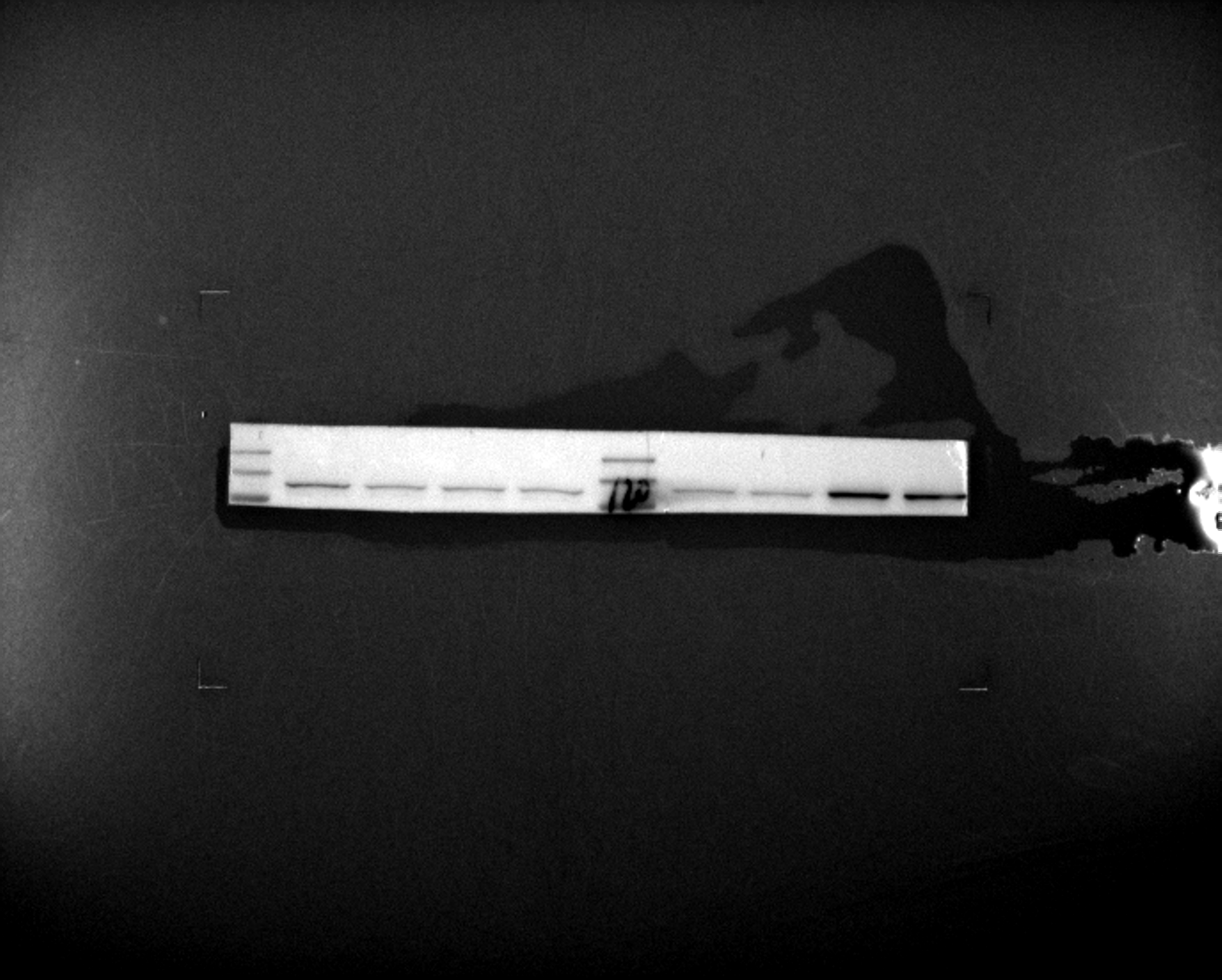

Supplement: Figure 6—source data 4. [file elife-96988-fig6-data4.zip › Figure 6-source data 4/2/SND1.Tif]

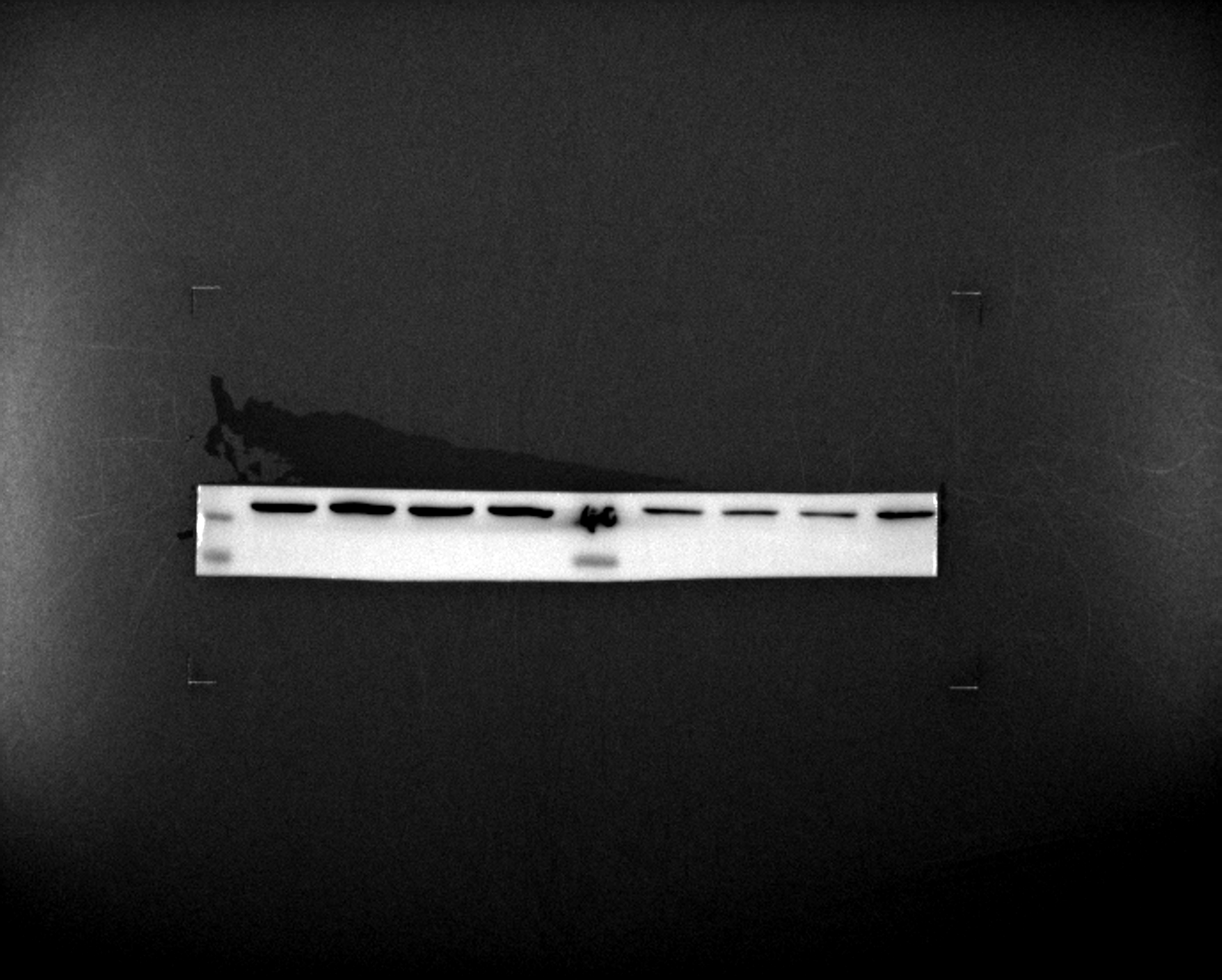

Supplement: Figure 6—source data 4. [file elife-96988-fig6-data4.zip › Figure 6-source data 4/2/β-ACTIN.Tif]

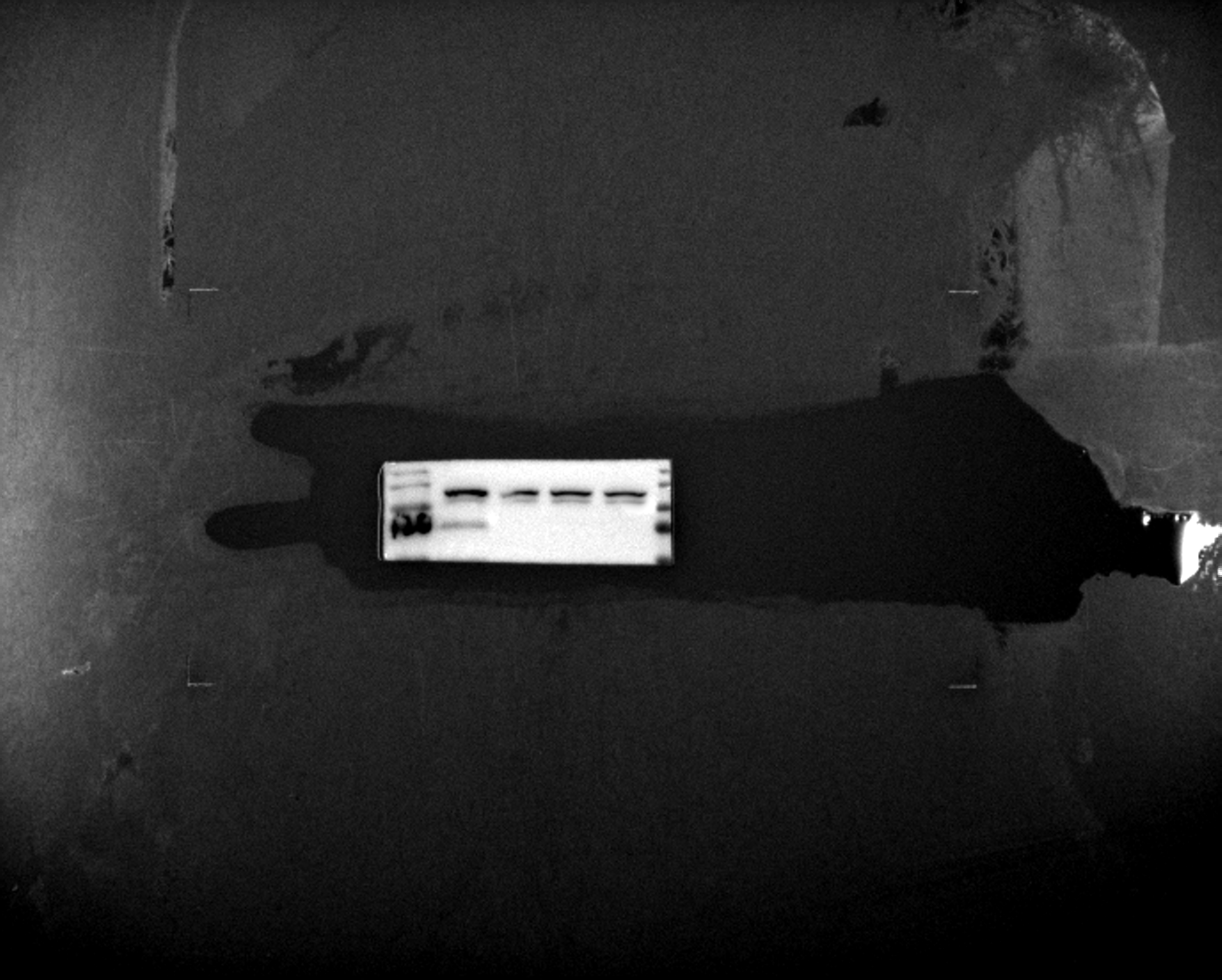

Supplement: Figure 6—source data 4. [file elife-96988-fig6-data4.zip › Figure 6-source data 4/3/SND1.Tif]

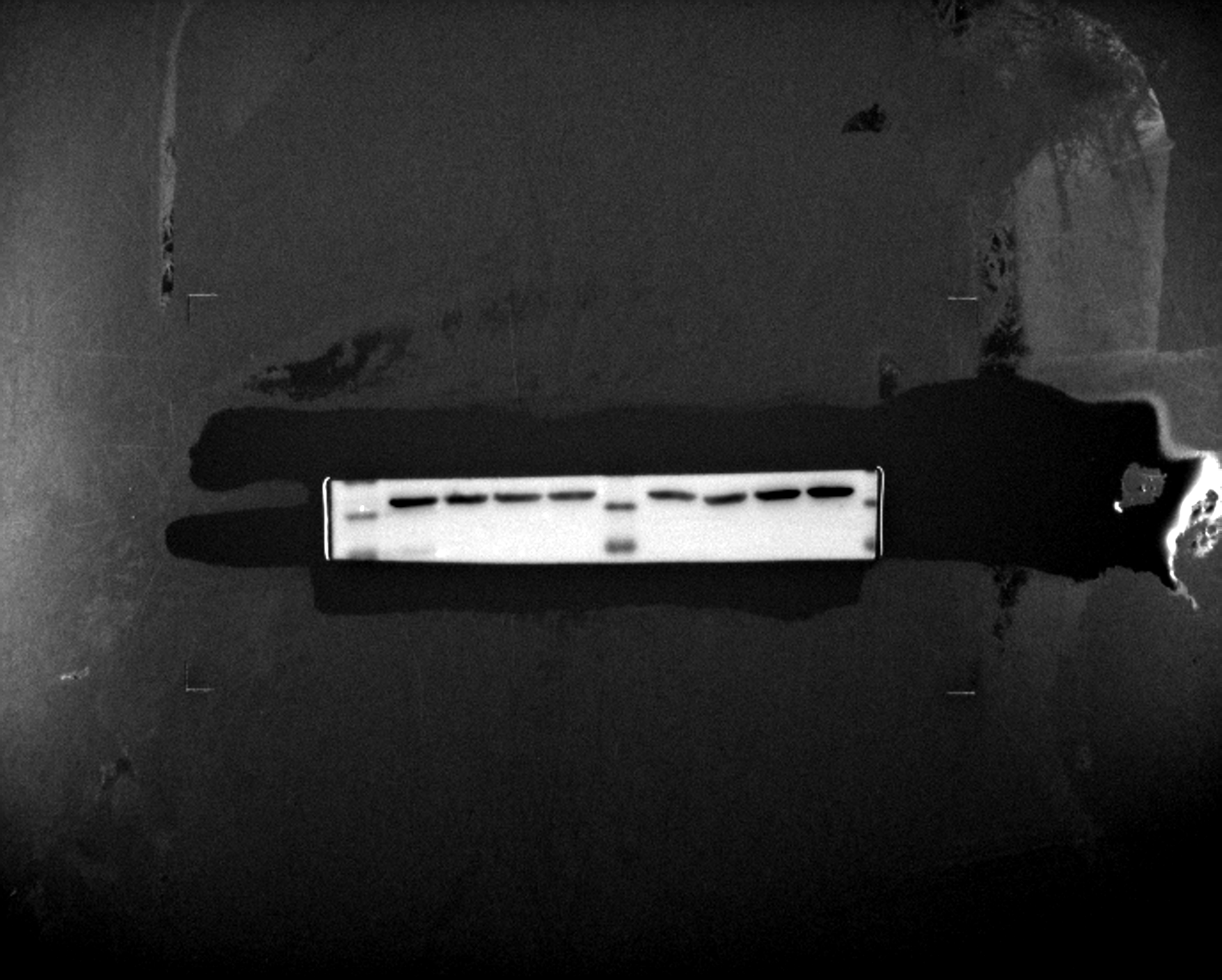

Supplement: Figure 6—source data 4. [file elife-96988-fig6-data4.zip › Figure 6-source data 4/3/β-ACTIN.Tif]

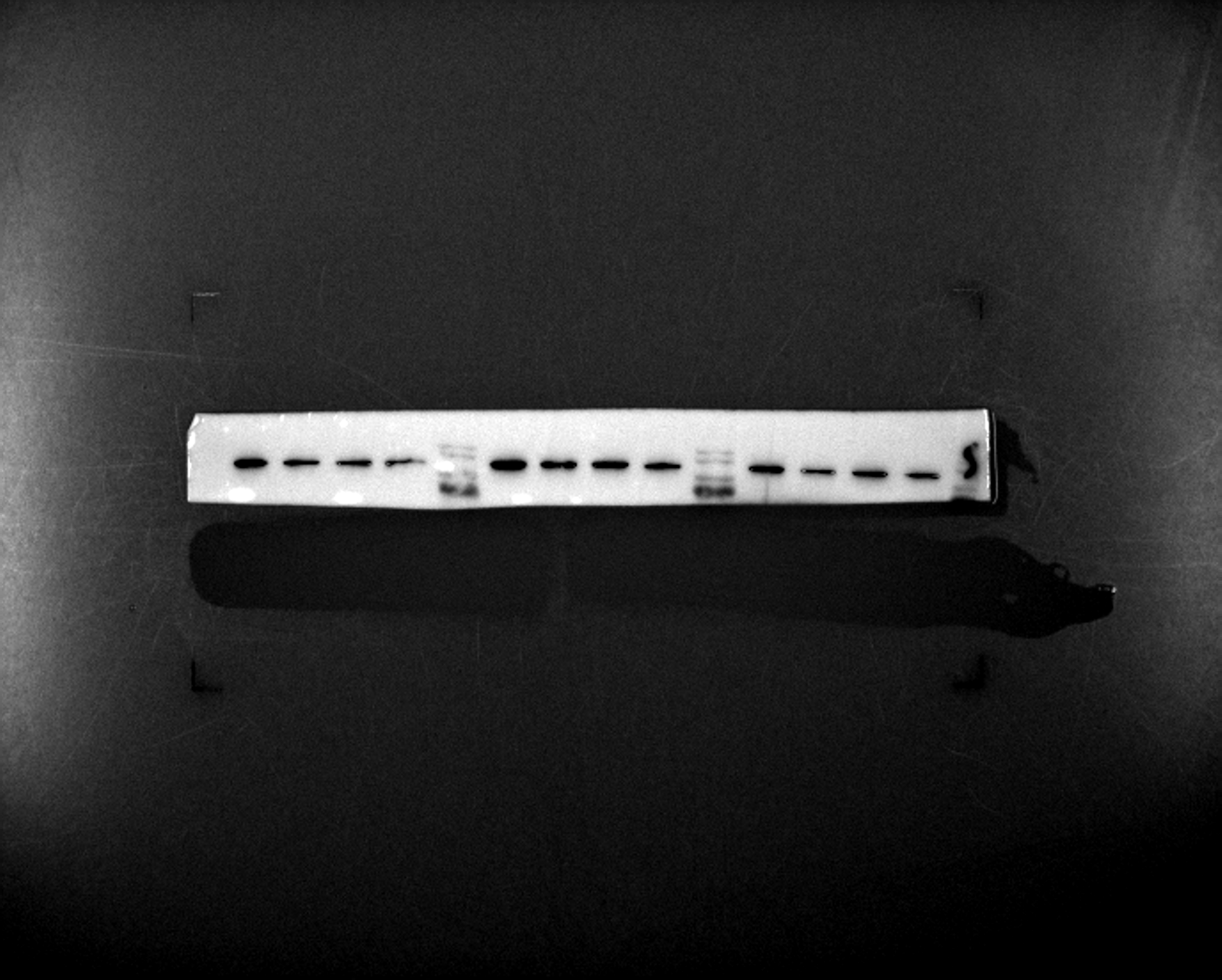

Supplement: Figure 6—source data 4. [file elife-96988-fig6-data4.zip › Figure 6-source data 4/4/SND1.Tif]

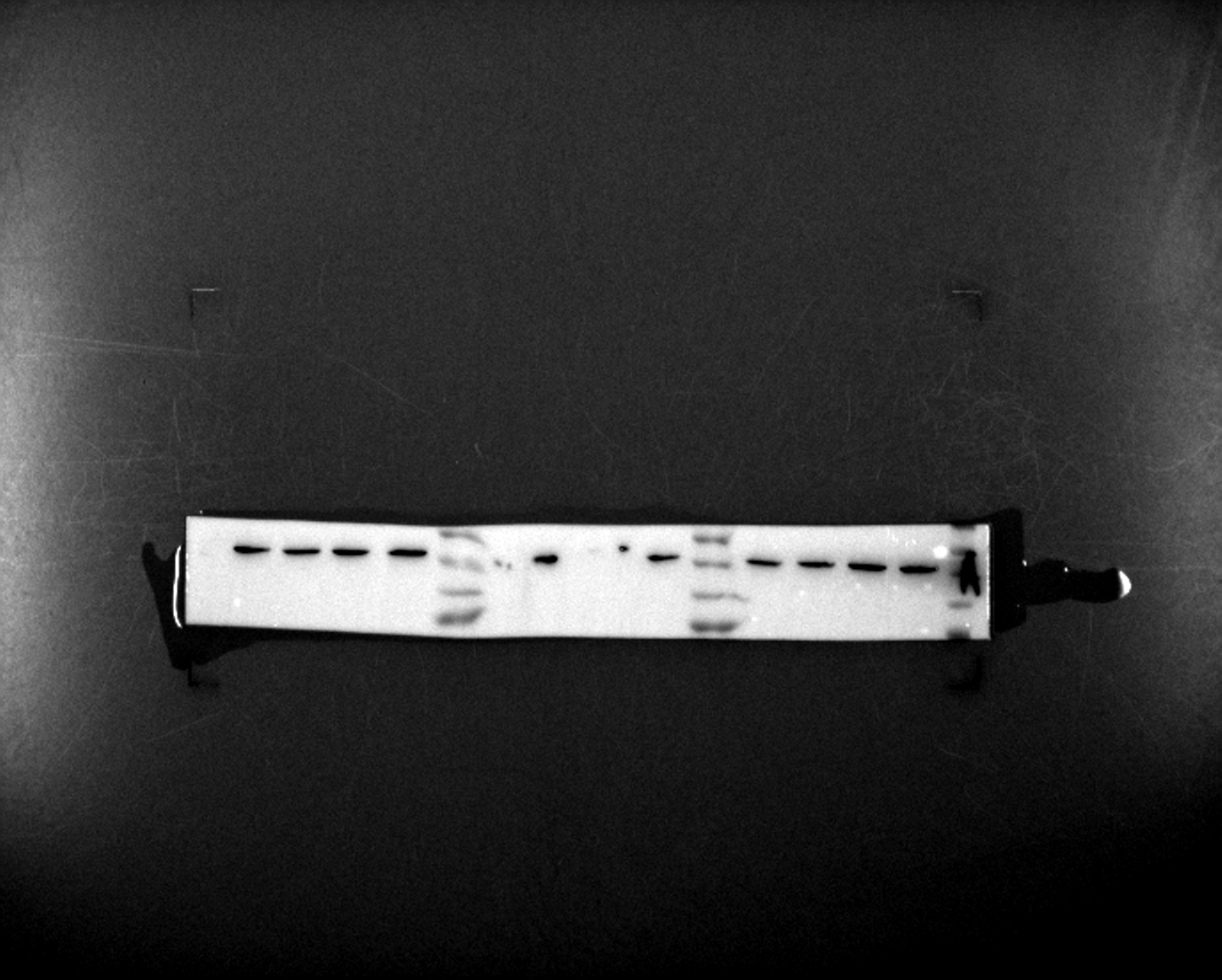

Supplement: Figure 6—source data 4. [file elife-96988-fig6-data4.zip › Figure 6-source data 4/4/β-ACTIN.Tif]

Figure 6C

1.

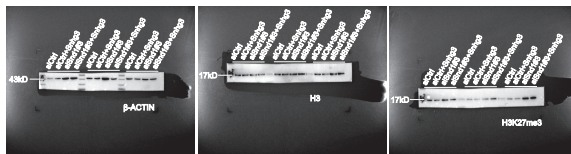

2.

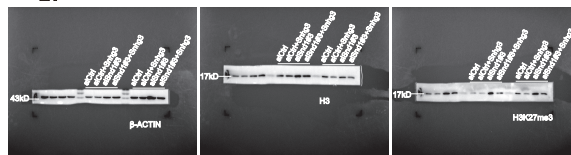

3.

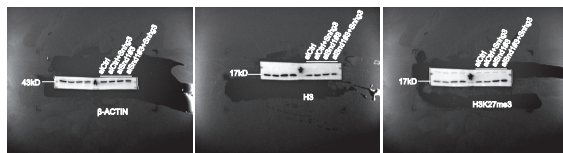

Supplement: Figure 6—source data 5. [file elife-96988-fig6-data5.pdf]

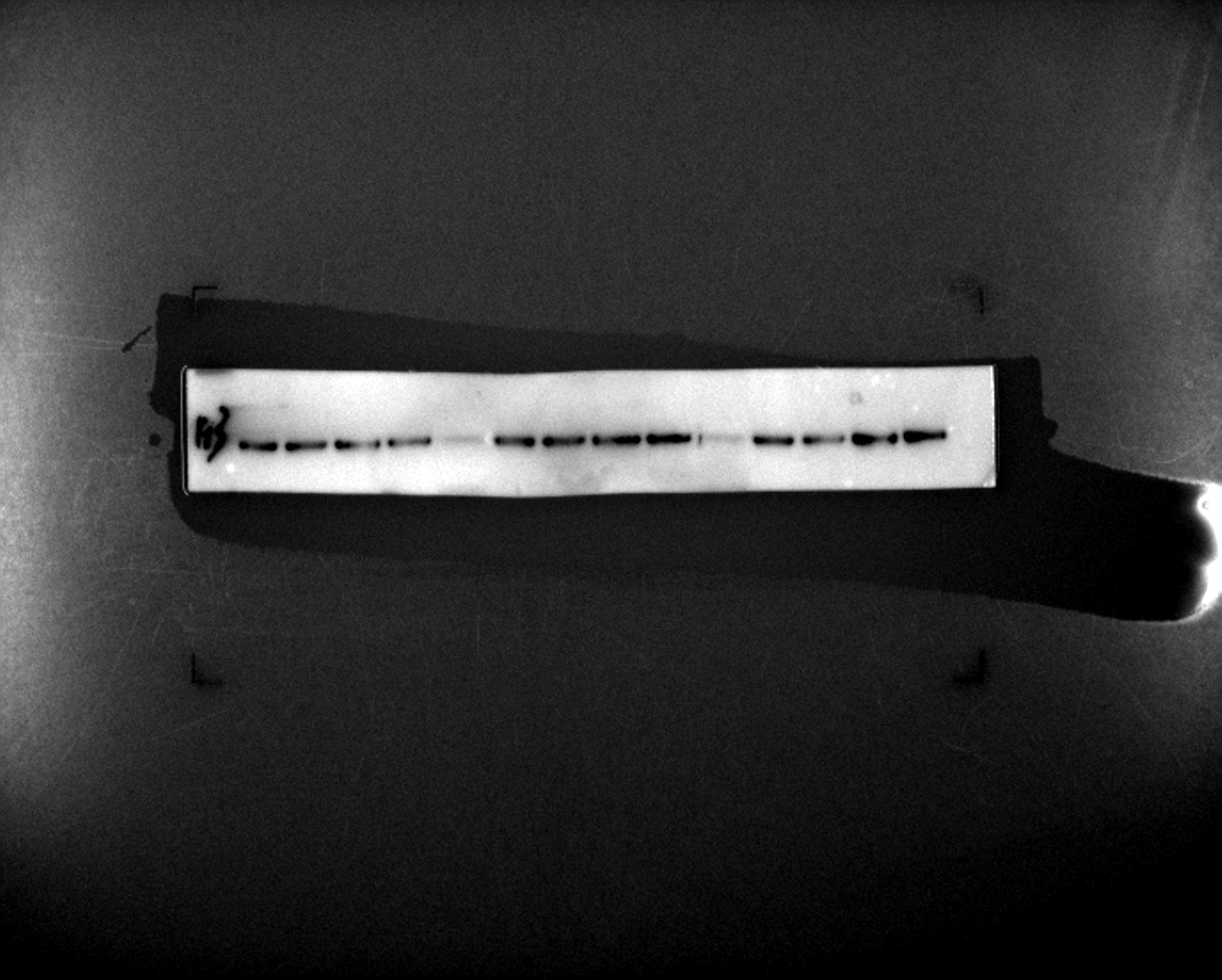

Supplement: Figure 6—source data 6. [file elife-96988-fig6-data6.zip › Figure 6-source data 6/1/H3.Tif]

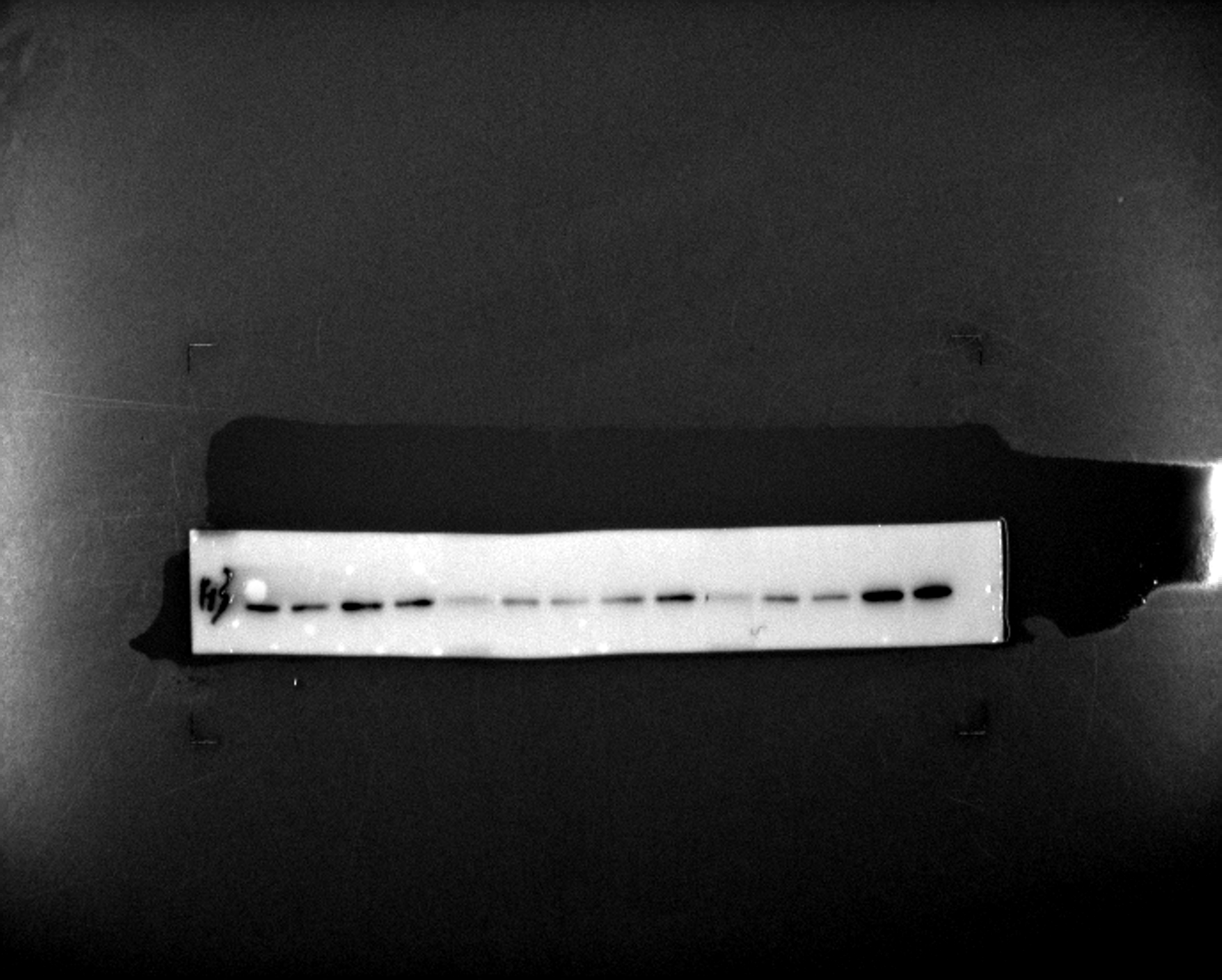

Supplement: Figure 6—source data 6. [file elife-96988-fig6-data6.zip › Figure 6-source data 6/1/H3K27me3.Tif]
