# Supplementary figures and images for: LncRNA Snhg3 aggravates hepatic steatosis via PPARγ signaling (part 2 of 2)
Source: eLife. 2024 Oct 22;13:RP96988. doi: 10.7554/eLife.96988 (PMC11495842; doi:10.7554/eLife.96988)

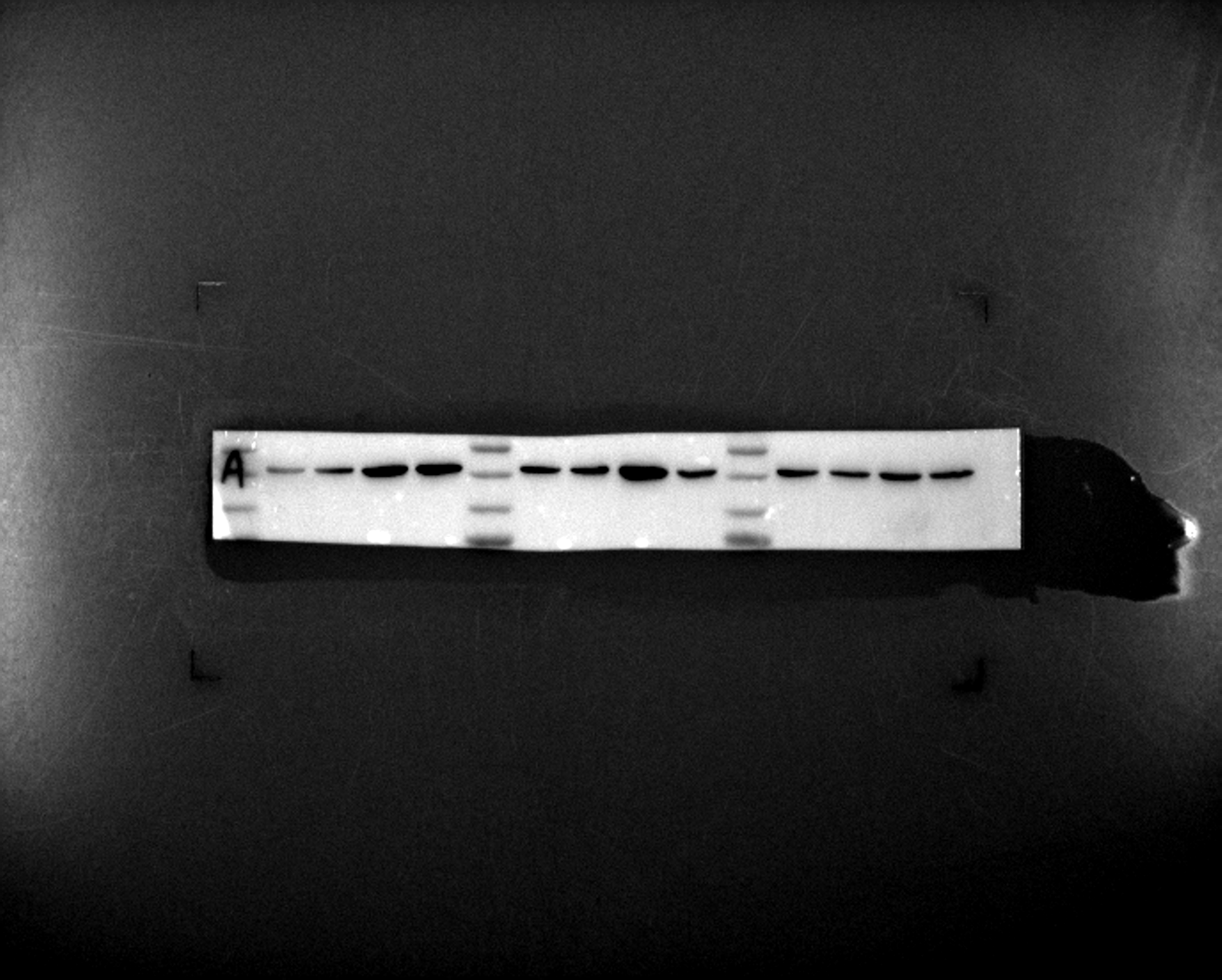

Supplement: Figure 6—source data 6. [file elife-96988-fig6-data6.zip › Figure 6-source data 6/1/β-ACTIN.Tif]

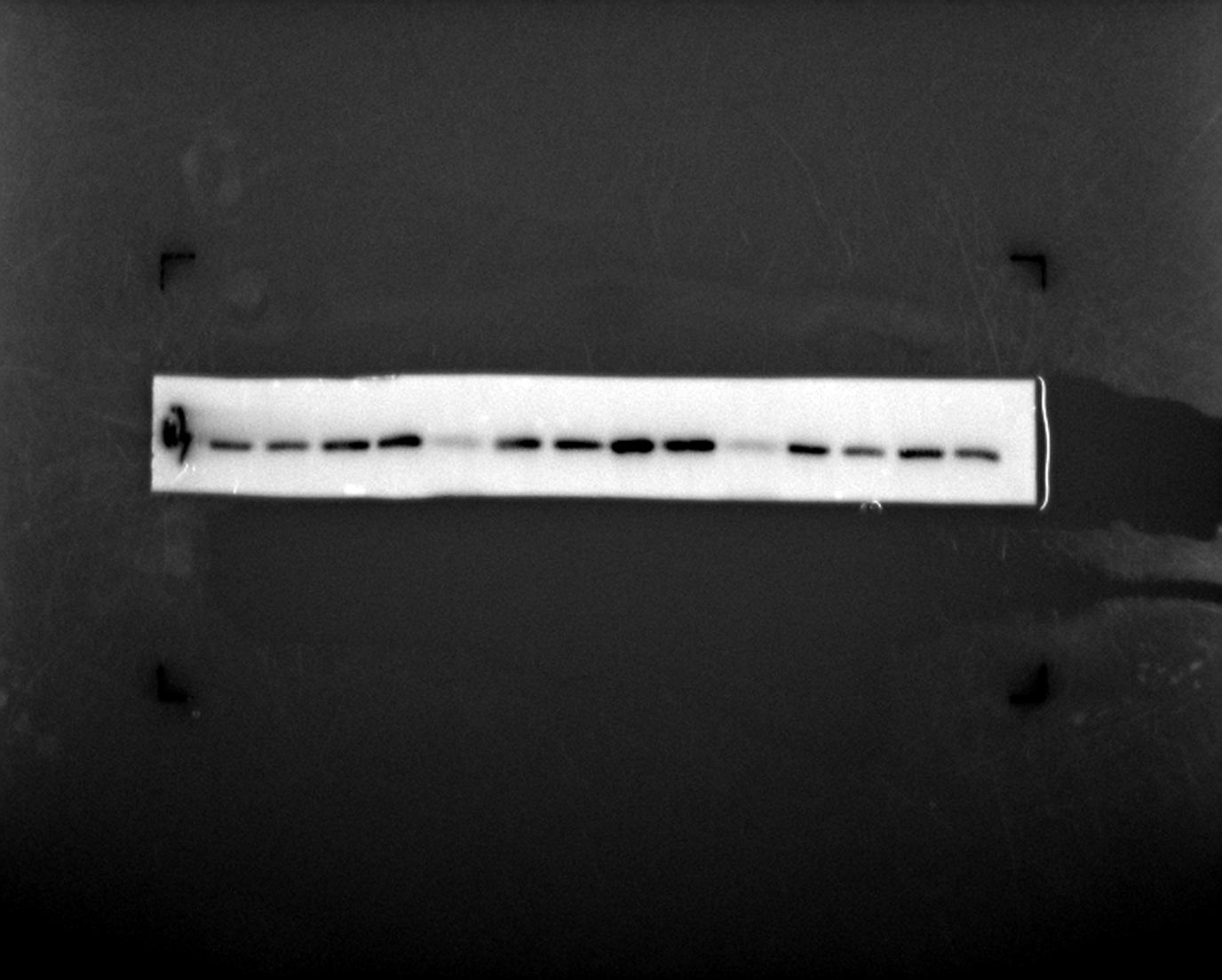

Supplement: Figure 6—source data 6. [file elife-96988-fig6-data6.zip › Figure 6-source data 6/2/H3.tif]

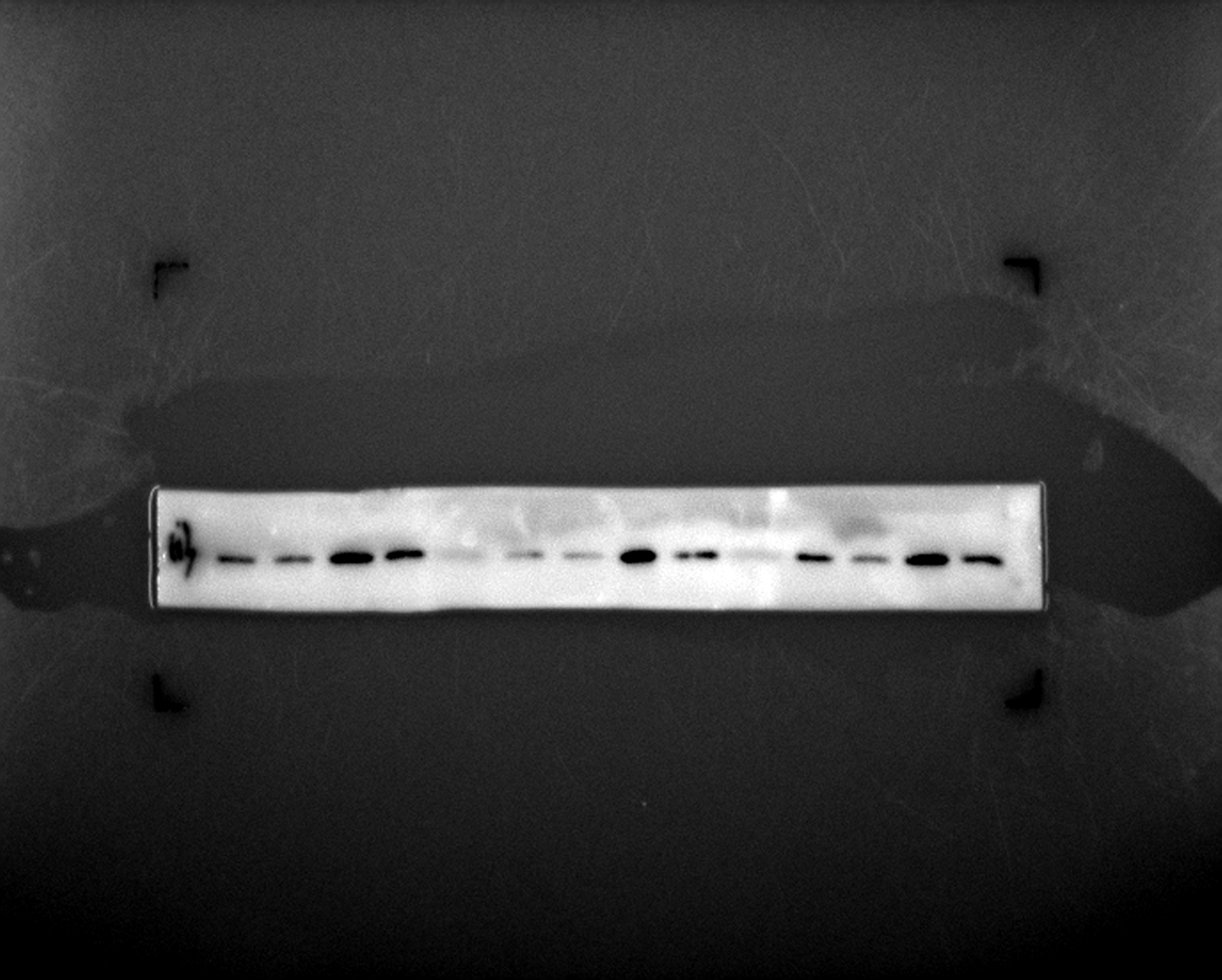

Supplement: Figure 6—source data 6. [file elife-96988-fig6-data6.zip › Figure 6-source data 6/2/H3K27me3.tif]

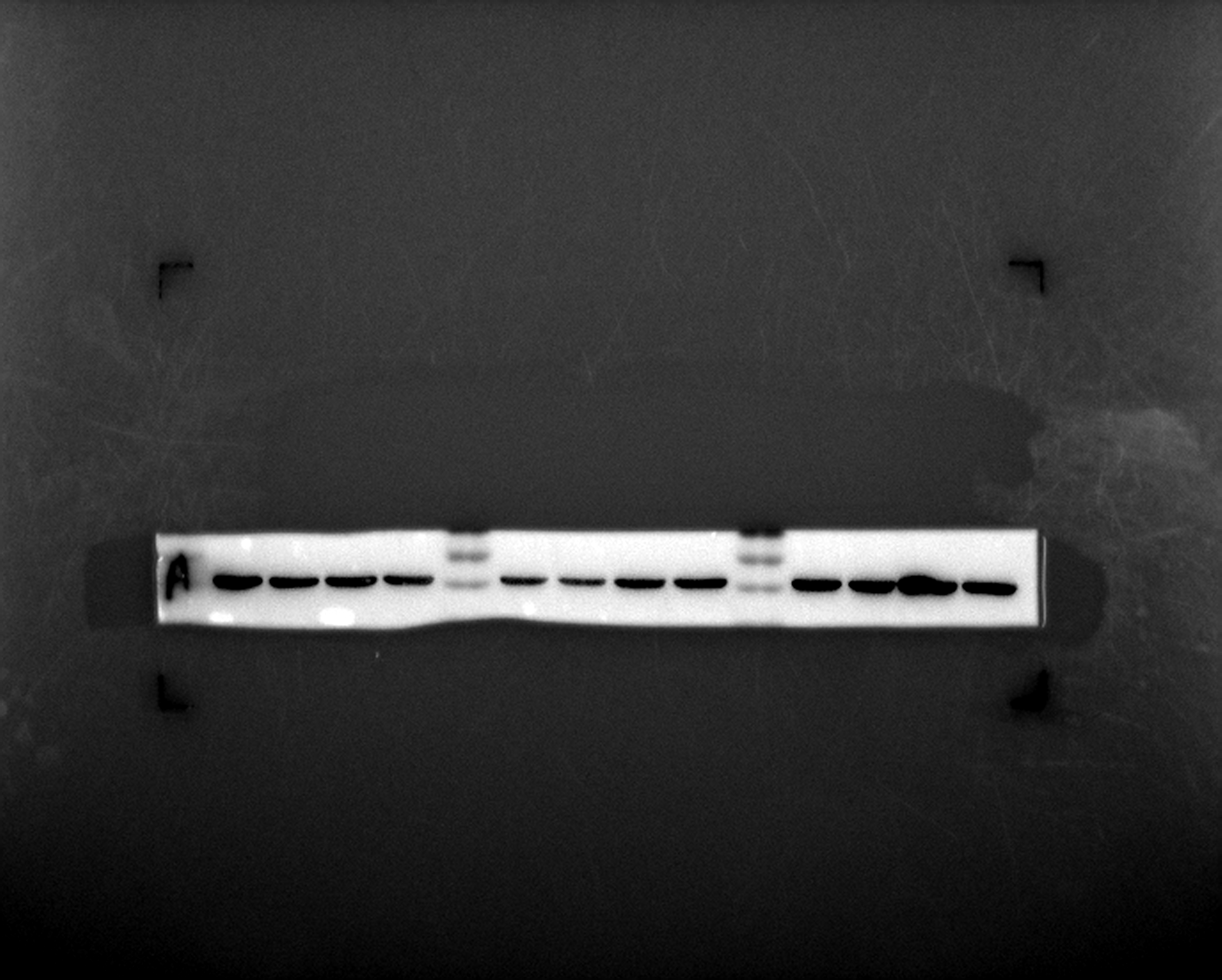

Supplement: Figure 6—source data 6. [file elife-96988-fig6-data6.zip › Figure 6-source data 6/2/β-ACTIN.tif]

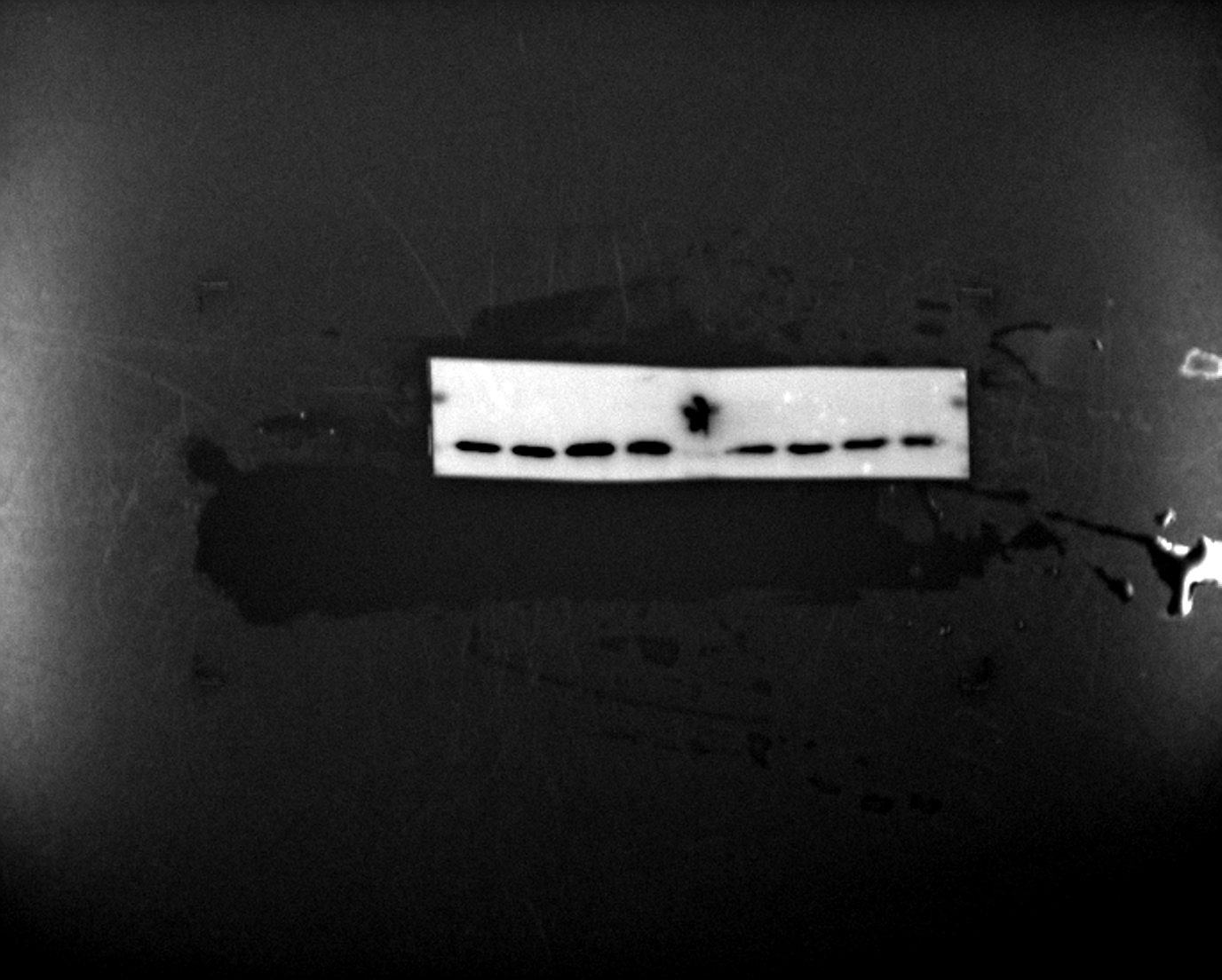

Supplement: Figure 6—source data 6. [file elife-96988-fig6-data6.zip › Figure 6-source data 6/3/H3.Tif]

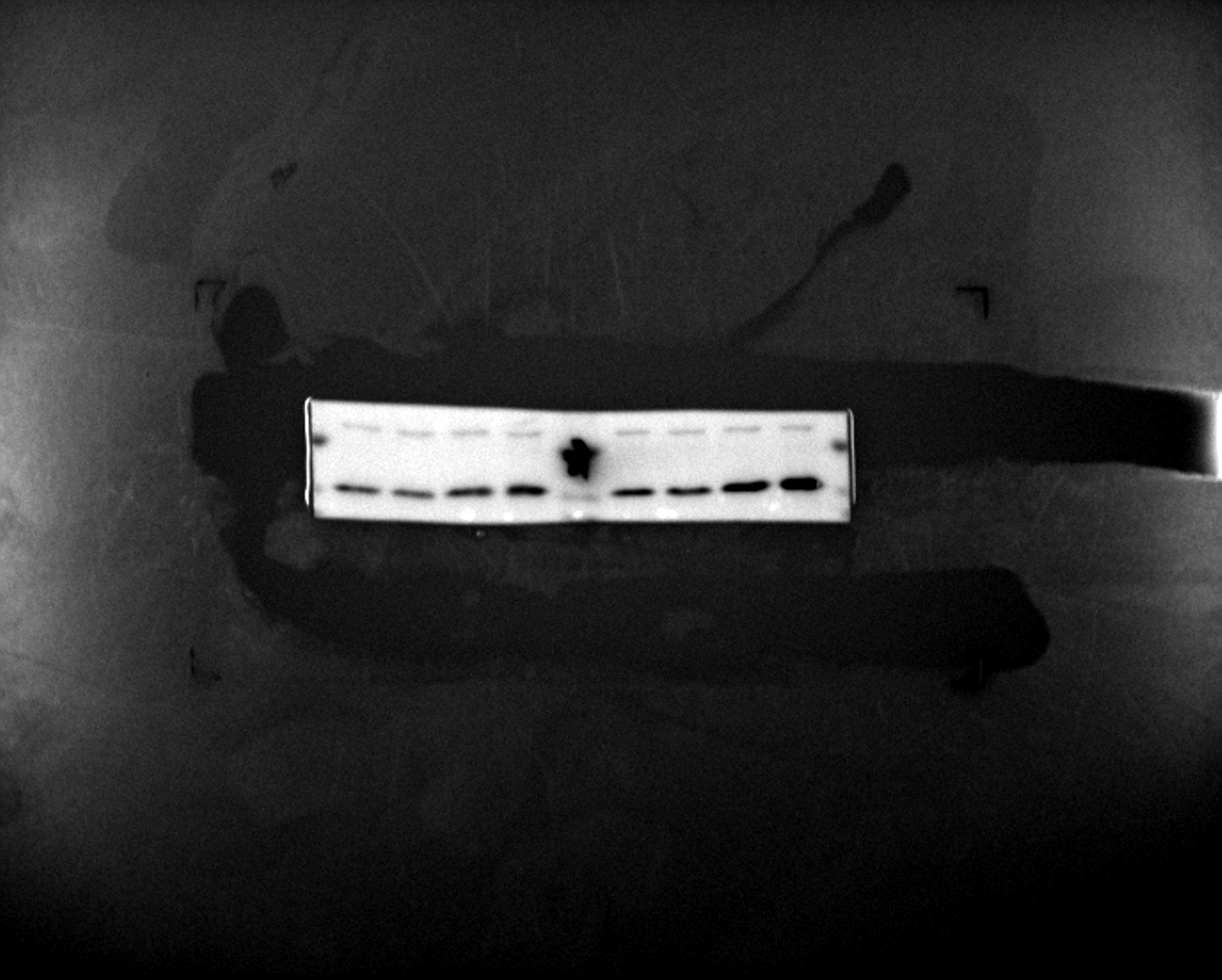

Supplement: Figure 6—source data 6. [file elife-96988-fig6-data6.zip › Figure 6-source data 6/3/H3K27me3.Tif]

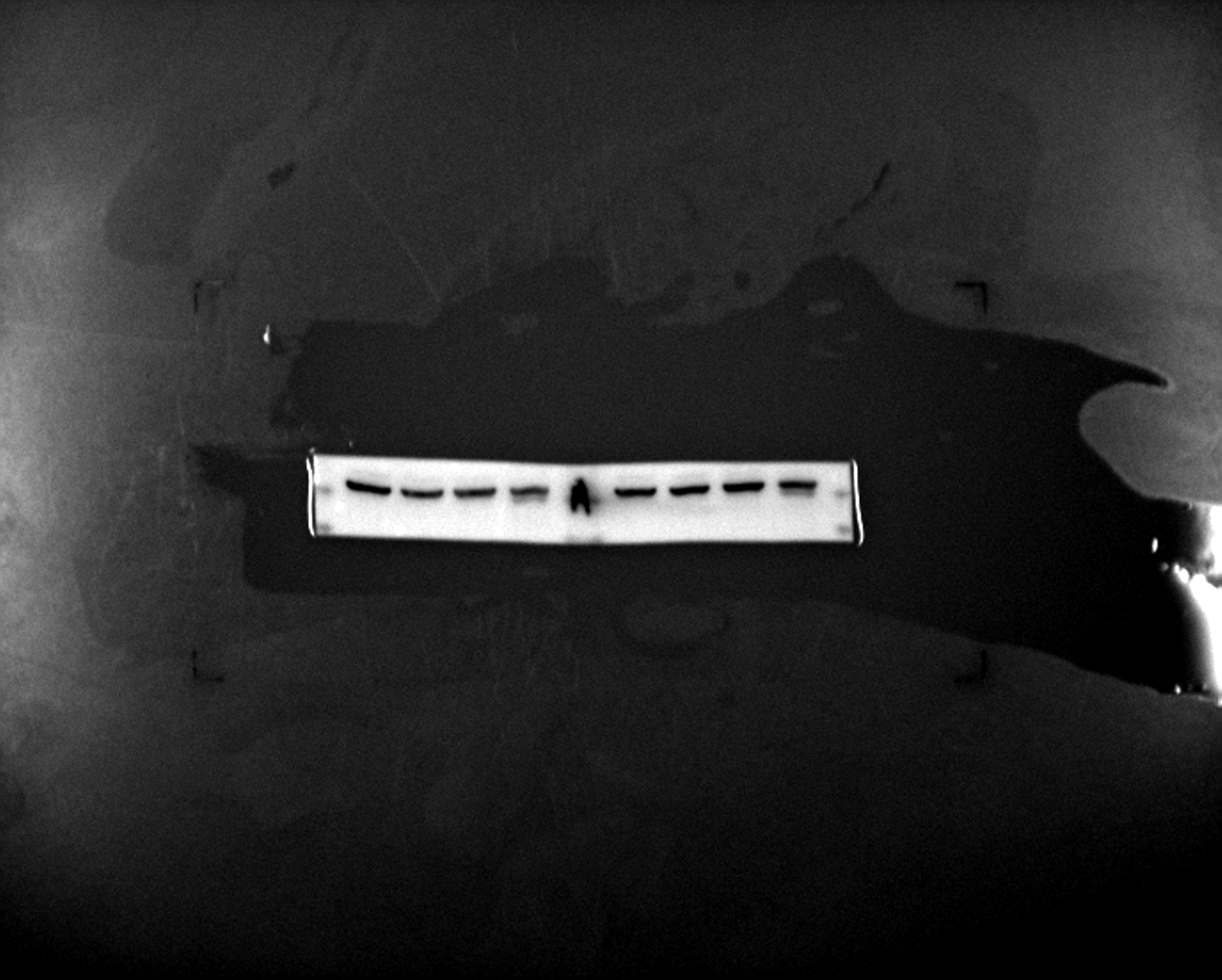

Supplement: Figure 6—source data 6. [file elife-96988-fig6-data6.zip › Figure 6-source data 6/3/β-ACTIN.Tif]

Figure 6D

1.

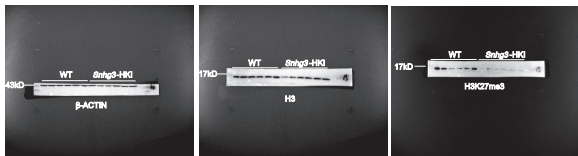

1.

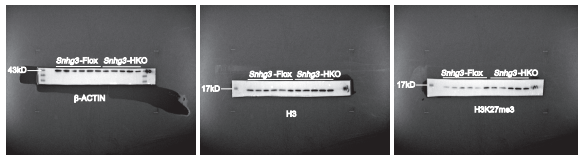

Supplement: Figure 6—source data 7. [file elife-96988-fig6-data7.pdf]

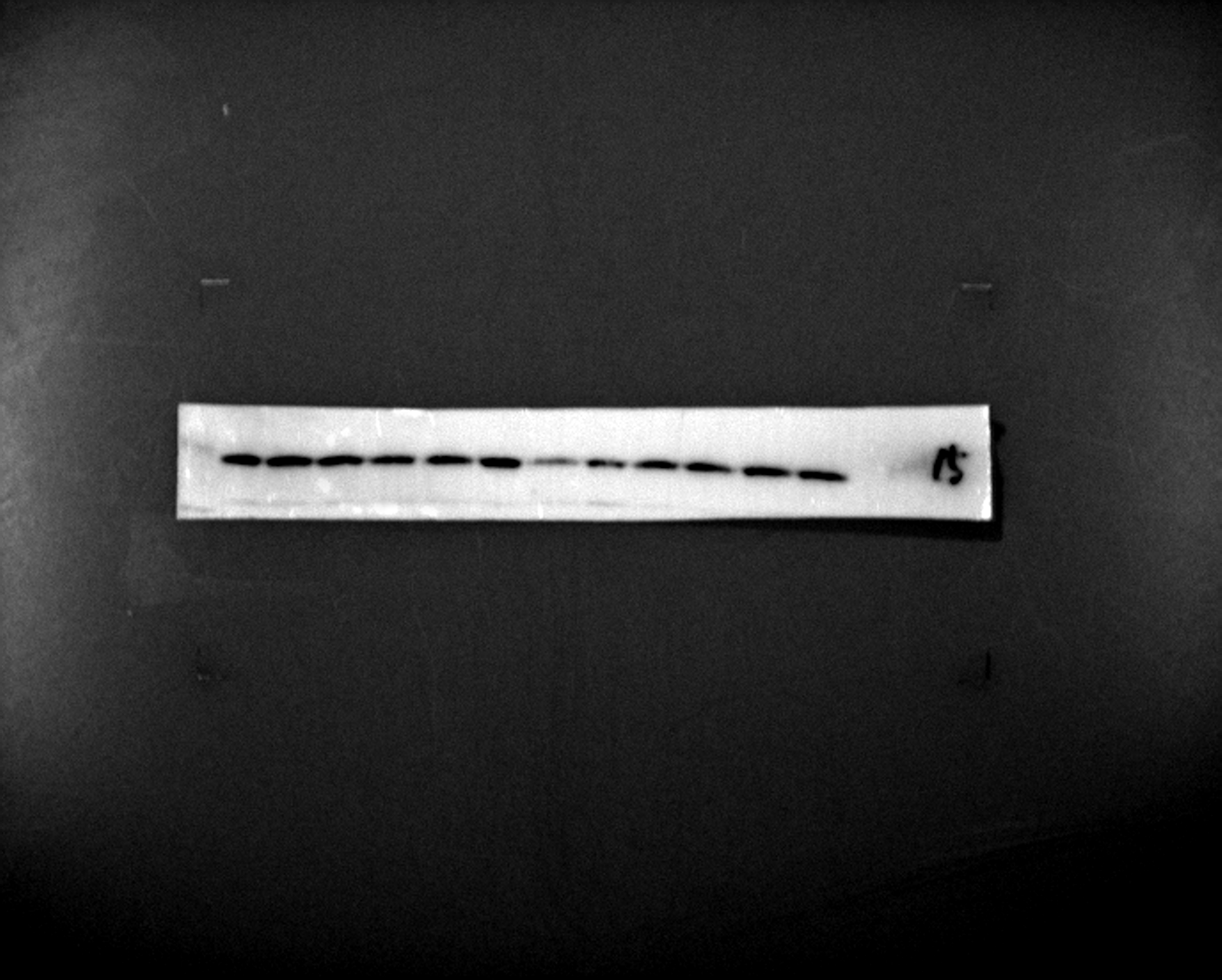

Supplement: Figure 6—source data 8. [file elife-96988-fig6-data8.zip › Figure 6-source data 8/H3K27me3 in Snhg3-HKI mice/H3.Tif]

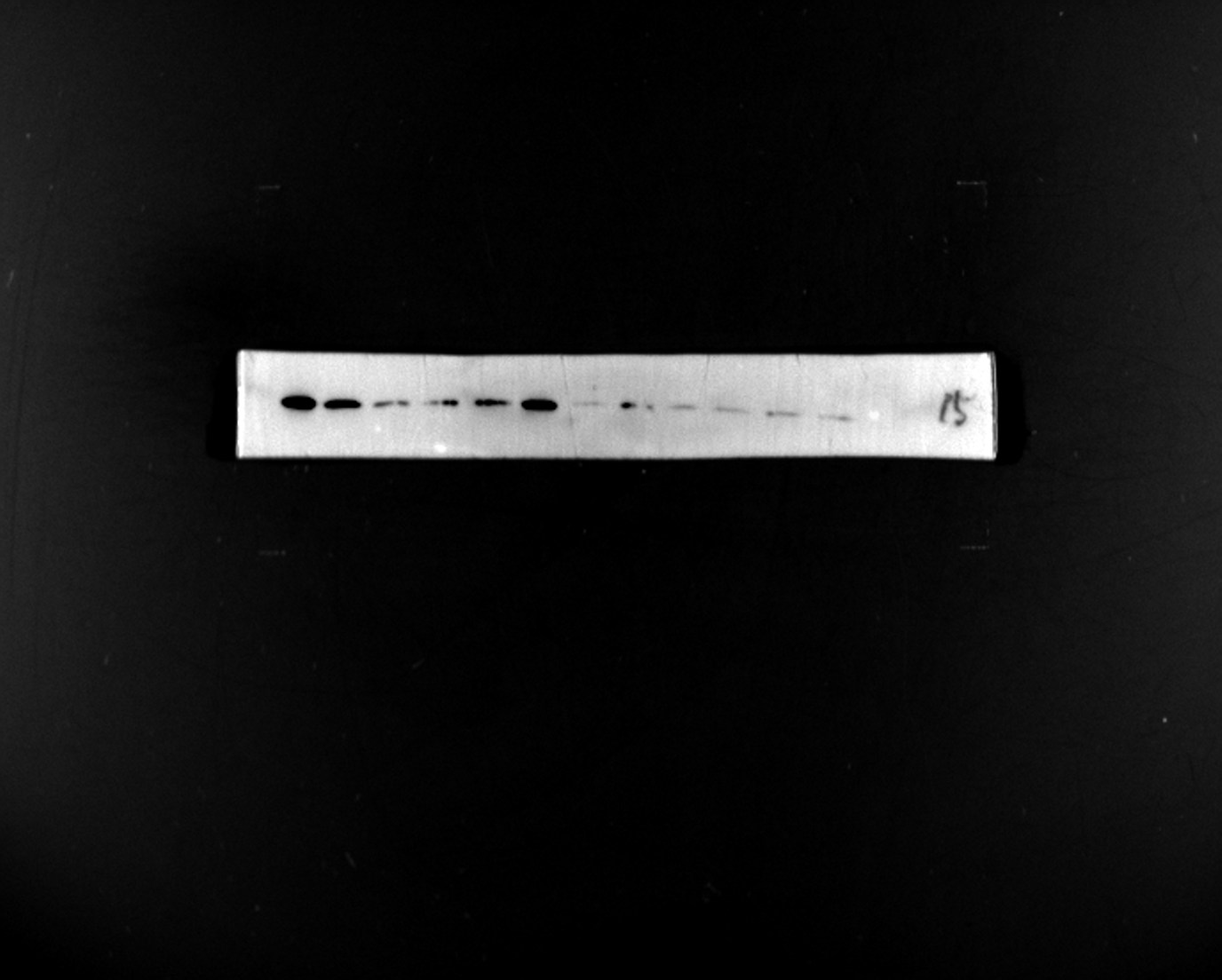

Supplement: Figure 6—source data 8. [file elife-96988-fig6-data8.zip › Figure 6-source data 8/H3K27me3 in Snhg3-HKI mice/H3K27me3.tif]

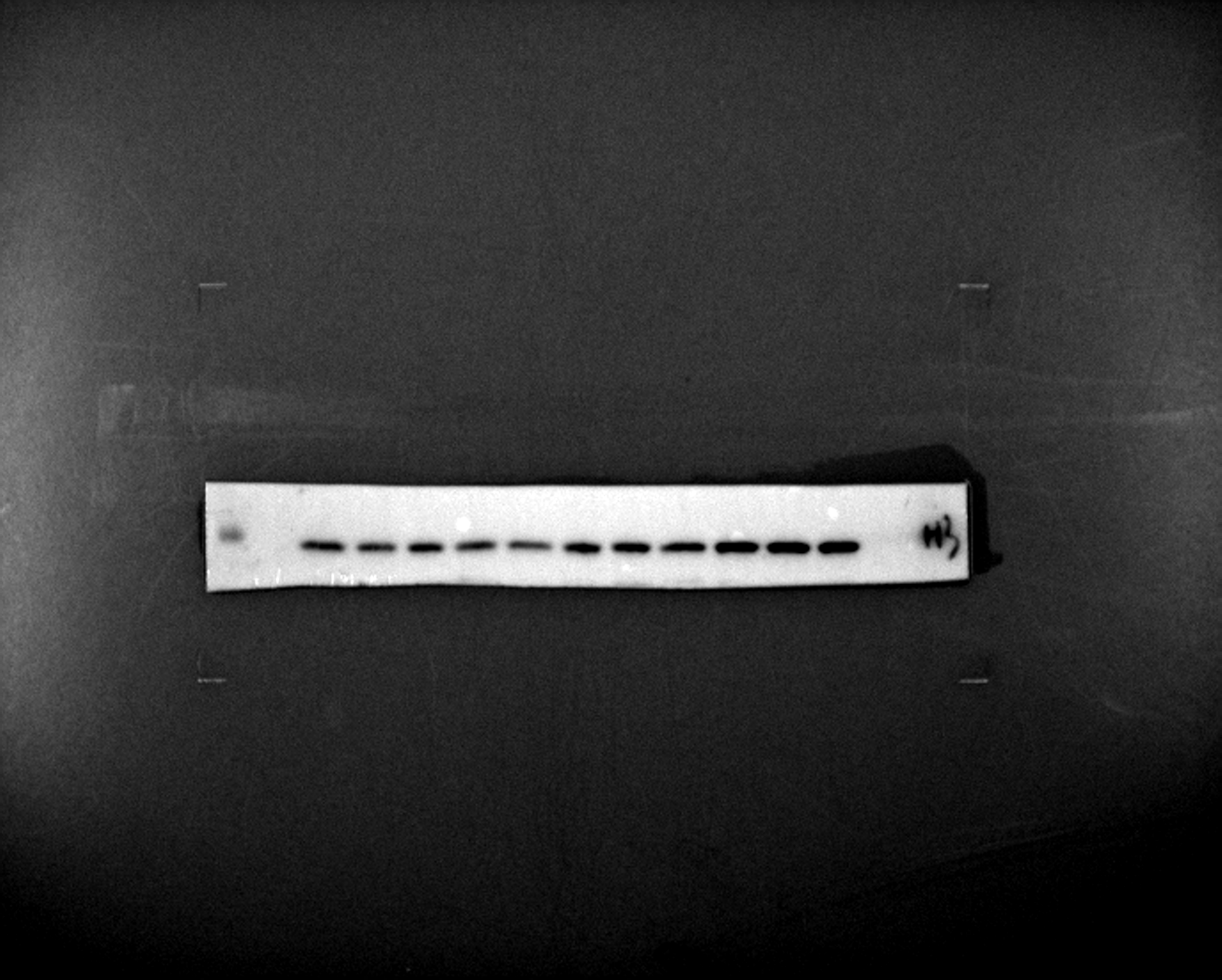

Supplement: Figure 6—source data 8. [file elife-96988-fig6-data8.zip › Figure 6-source data 8/H3K27me3 in Snhg3-HKO mice/H3.Tif]

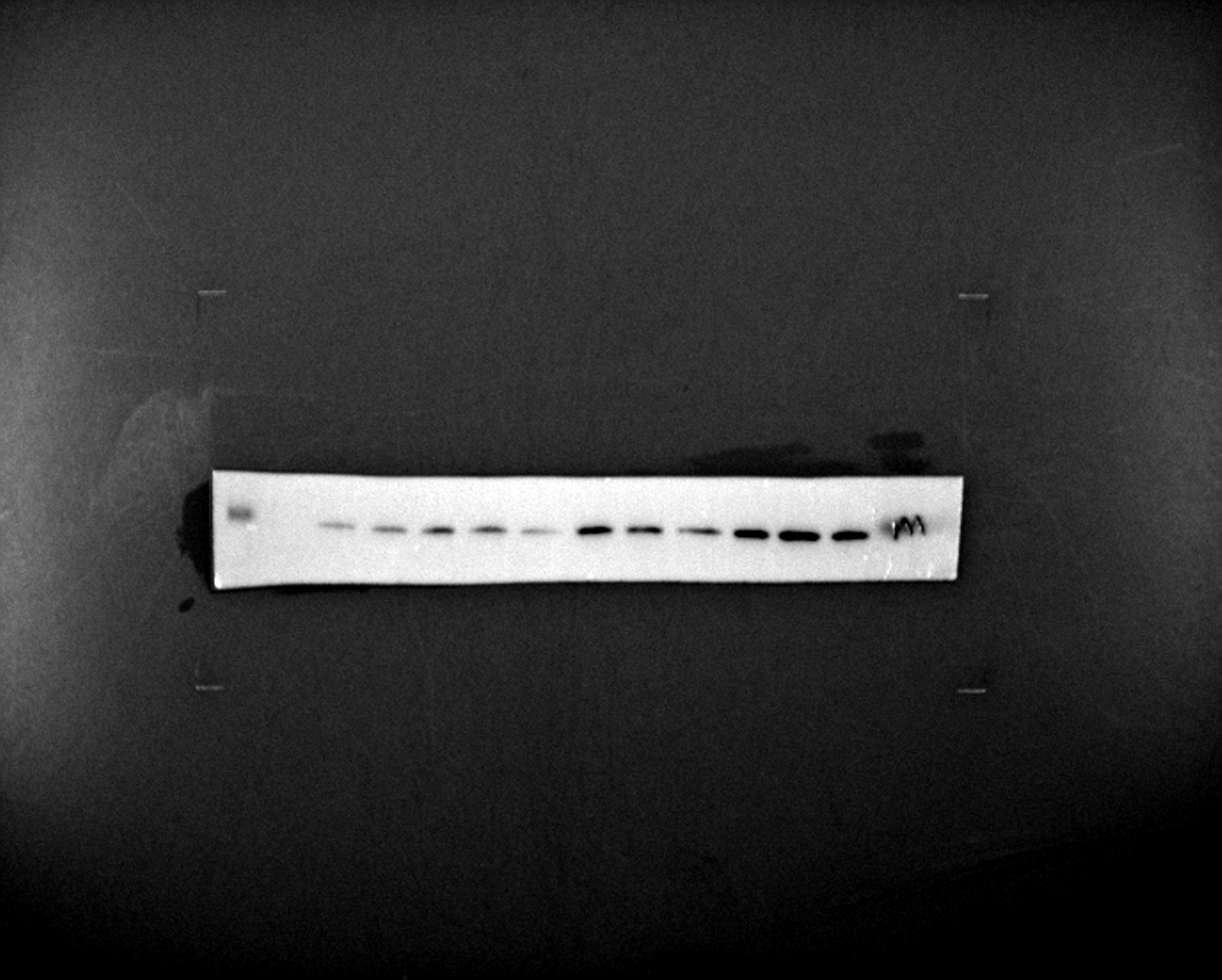

Supplement: Figure 6—source data 8. [file elife-96988-fig6-data8.zip › Figure 6-source data 8/H3K27me3 in Snhg3-HKO mice/H3K27me3.Tif]

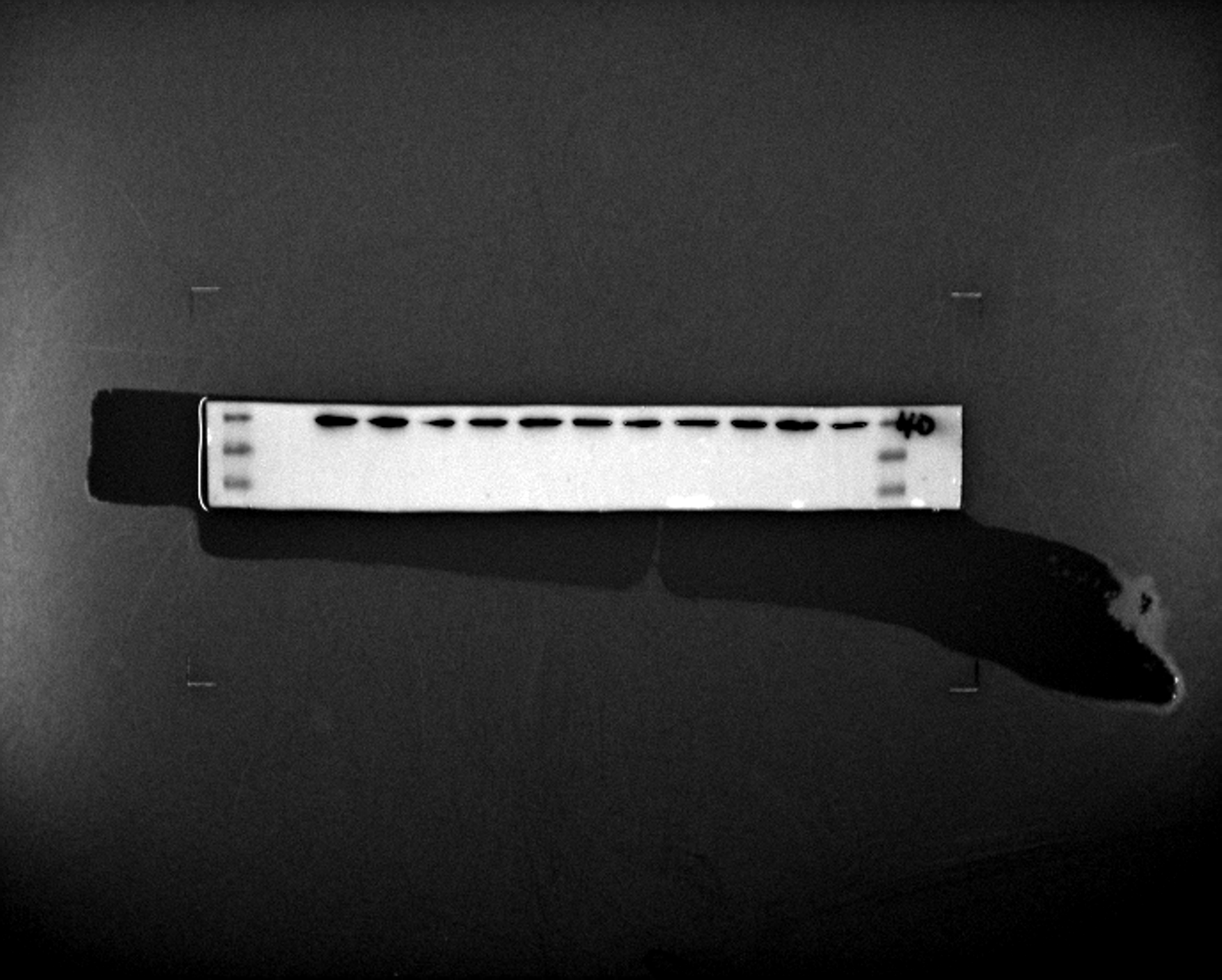

Supplement: Figure 6—source data 8. [file elife-96988-fig6-data8.zip › Figure 6-source data 8/H3K27me3 in Snhg3-HKO mice/β-ACTIN.Tif]

Figure 7B

1.

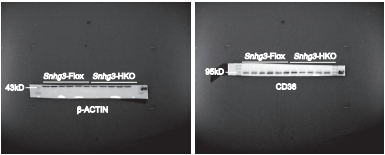

1.

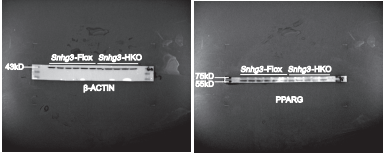

2.

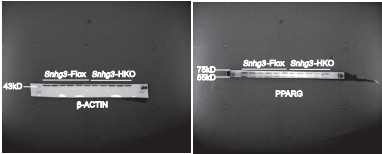

3.

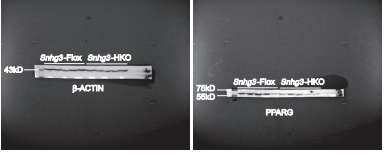

4.

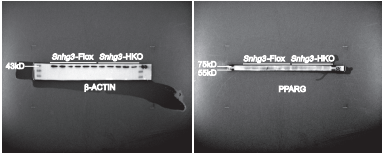

5.

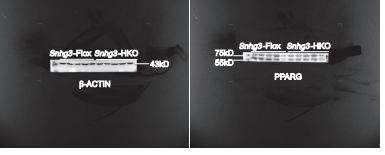

Supplement: Figure 7—source data 1. [file elife-96988-fig7-data1.pdf]

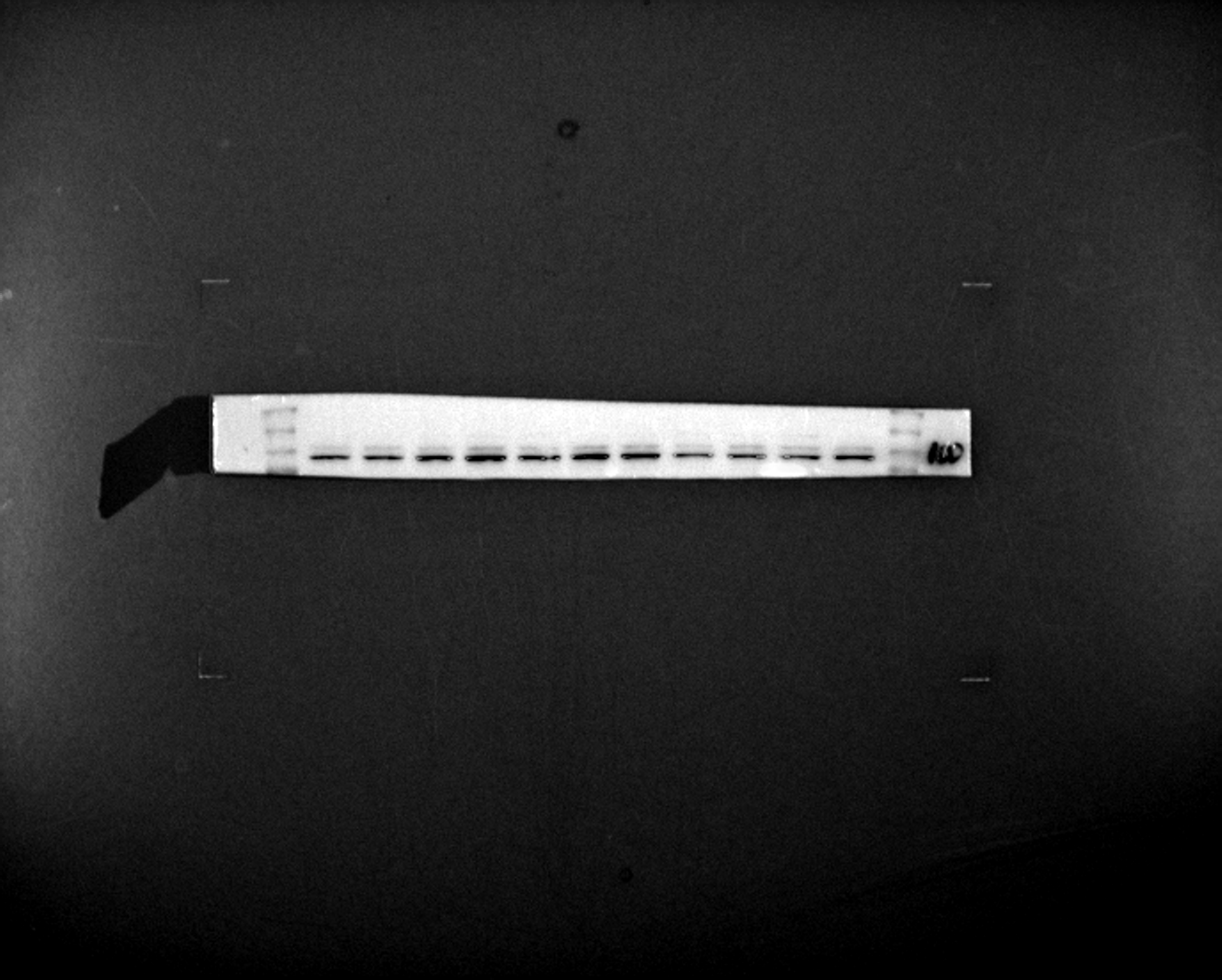

Supplement: Figure 7—source data 2. [file elife-96988-fig7-data2.zip › Figure 7-source data 2/CD36 in Snhg3-HKO mice/1/CD36.Tif]

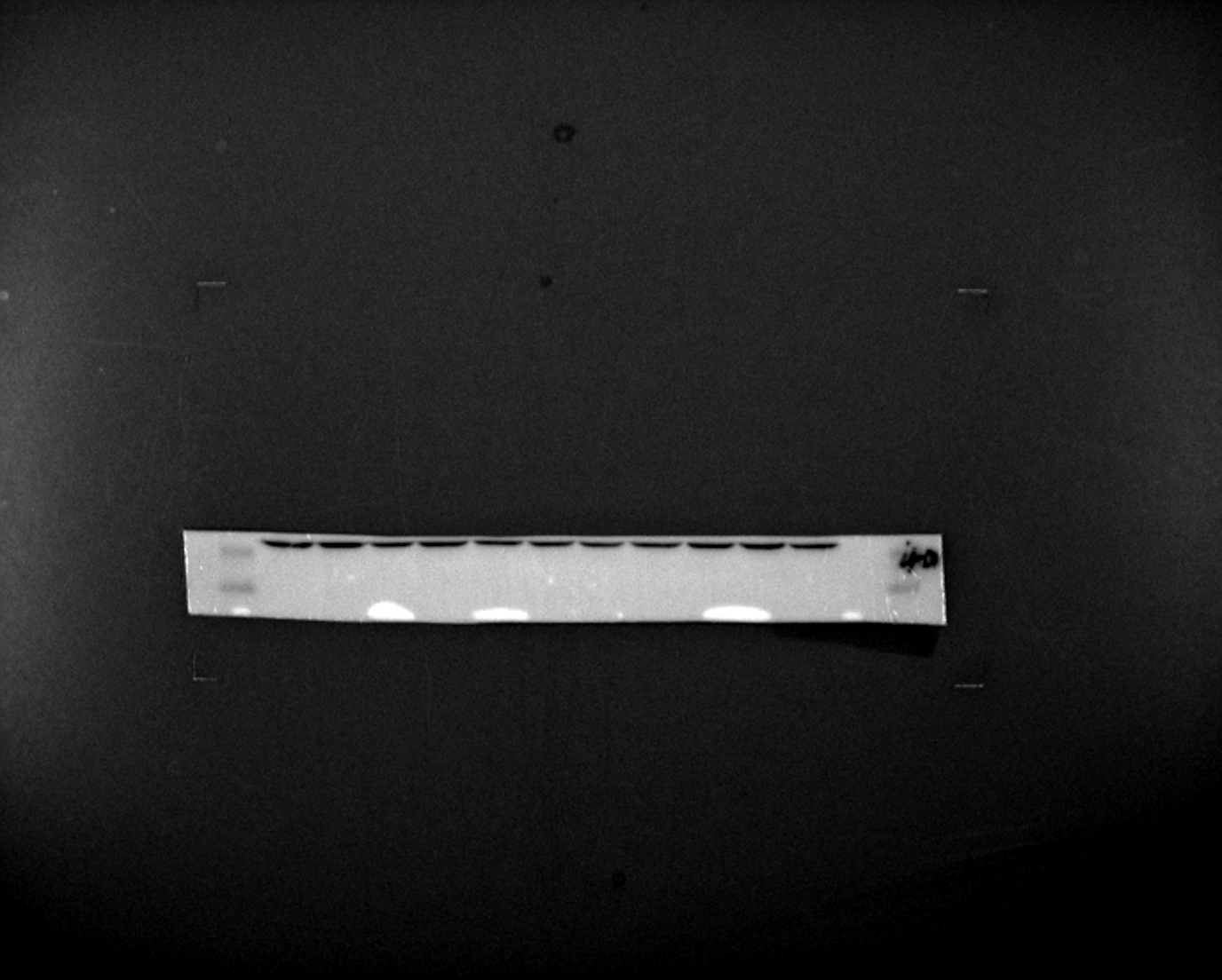

Supplement: Figure 7—source data 2. [file elife-96988-fig7-data2.zip › Figure 7-source data 2/CD36 in Snhg3-HKO mice/1/β-ACTIN.Tif]

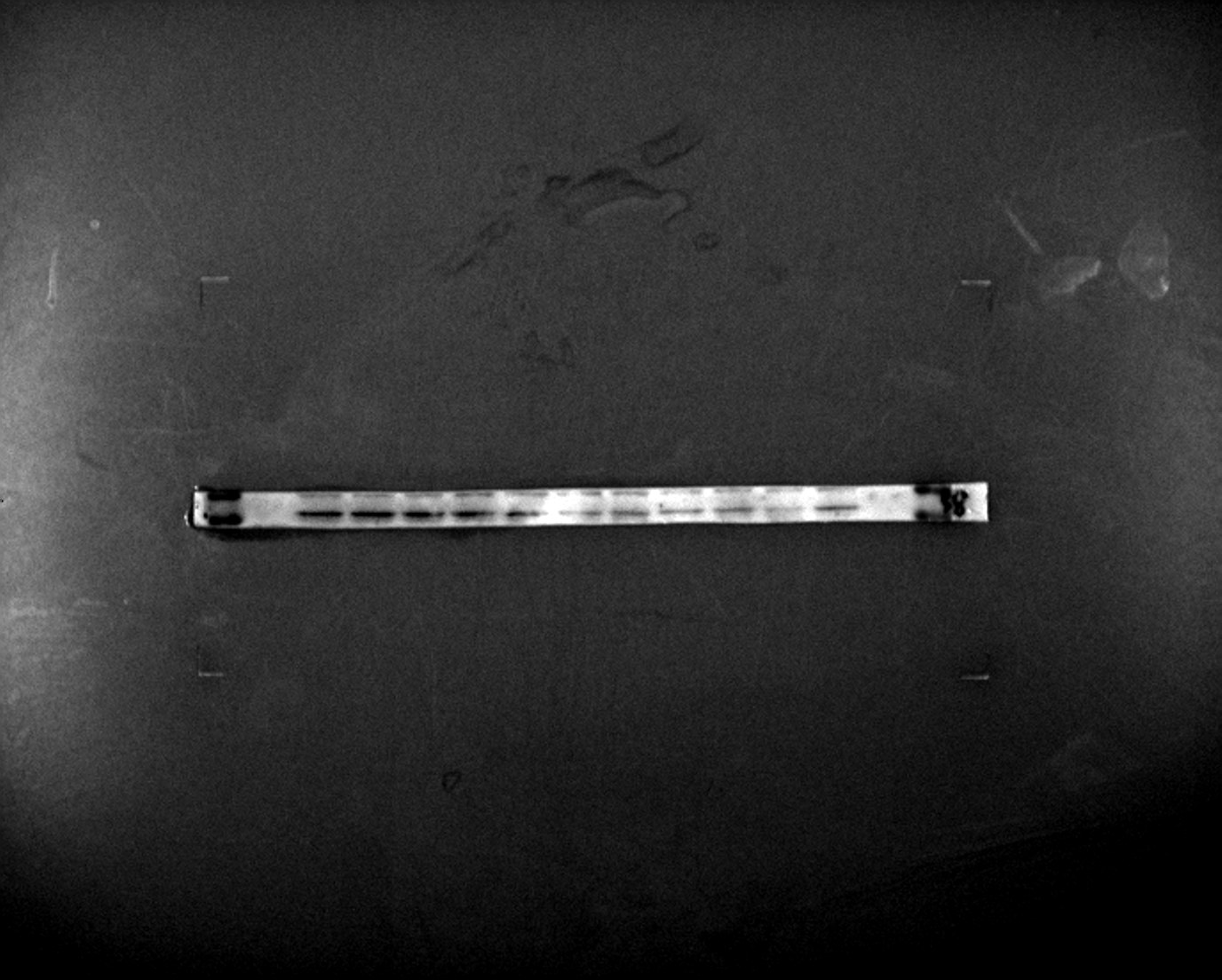

Supplement: Figure 7—source data 2. [file elife-96988-fig7-data2.zip › Figure 7-source data 2/PPARG in Snhg3-HKO mice/1/PPARG.Tif]

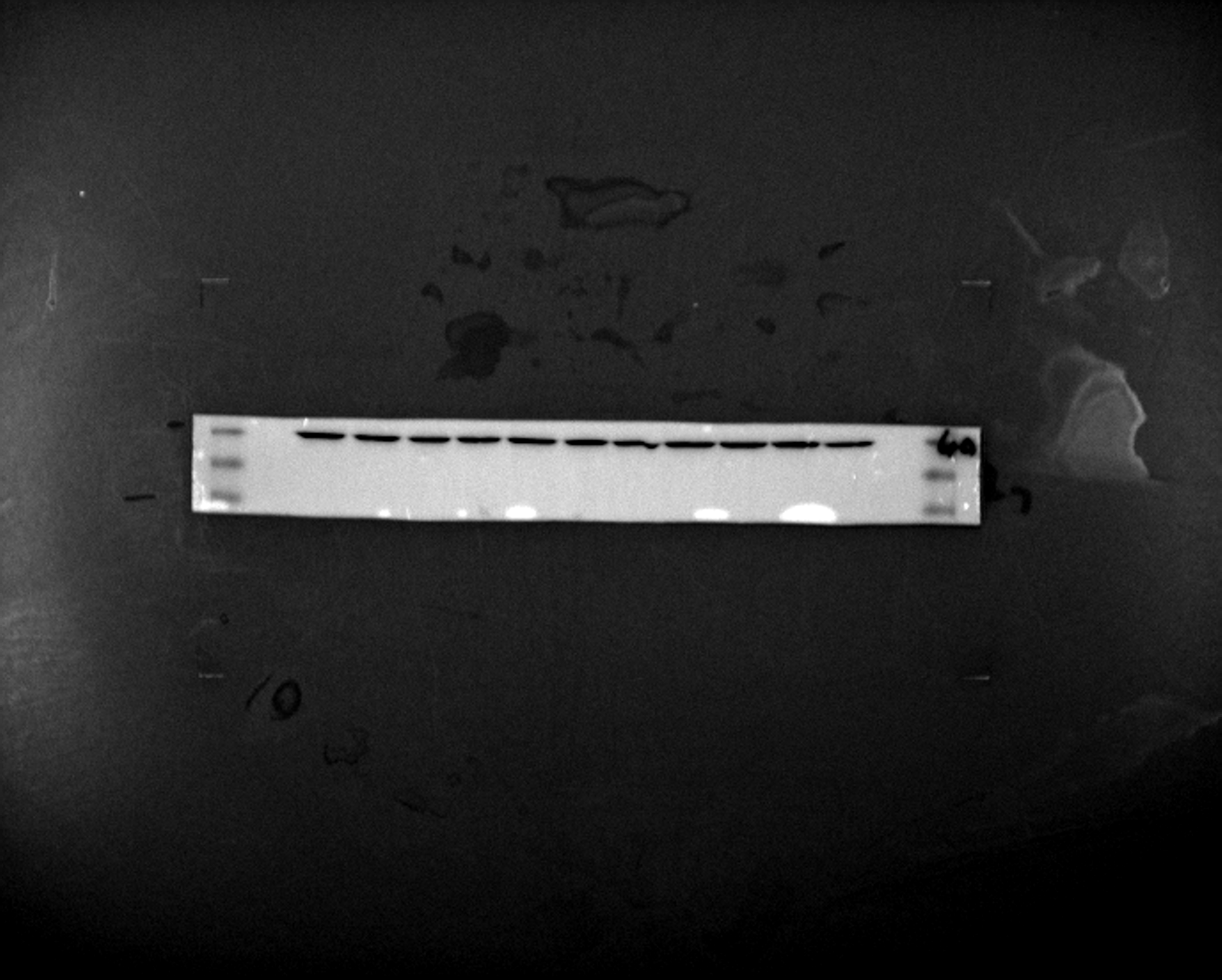

Supplement: Figure 7—source data 2. [file elife-96988-fig7-data2.zip › Figure 7-source data 2/PPARG in Snhg3-HKO mice/1/β-ACTIN.Tif]

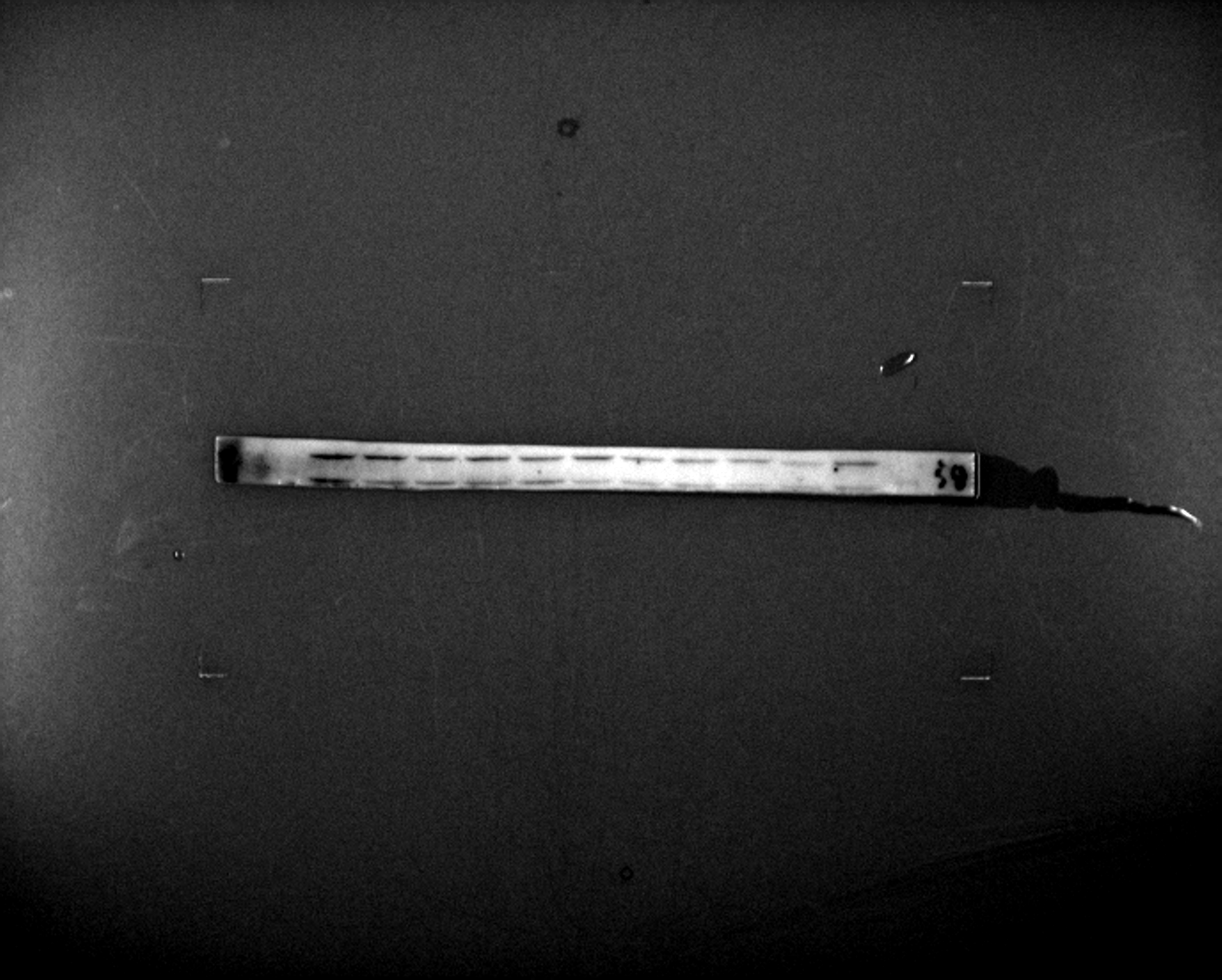

Supplement: Figure 7—source data 2. [file elife-96988-fig7-data2.zip › Figure 7-source data 2/PPARG in Snhg3-HKO mice/2/PPARG.Tif]

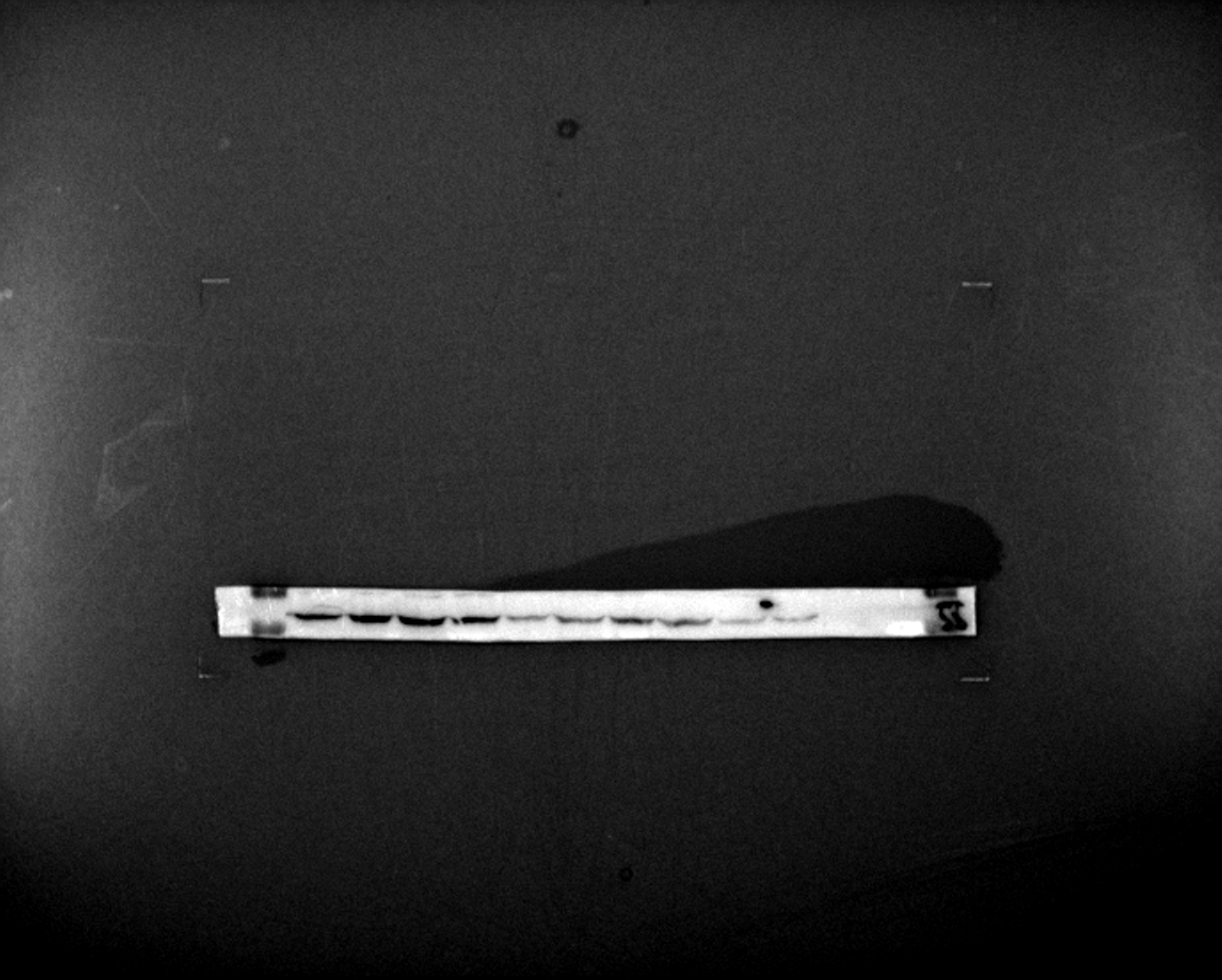

Supplement: Figure 7—source data 2. [file elife-96988-fig7-data2.zip › Figure 7-source data 2/PPARG in Snhg3-HKO mice/3/PPARG.Tif]

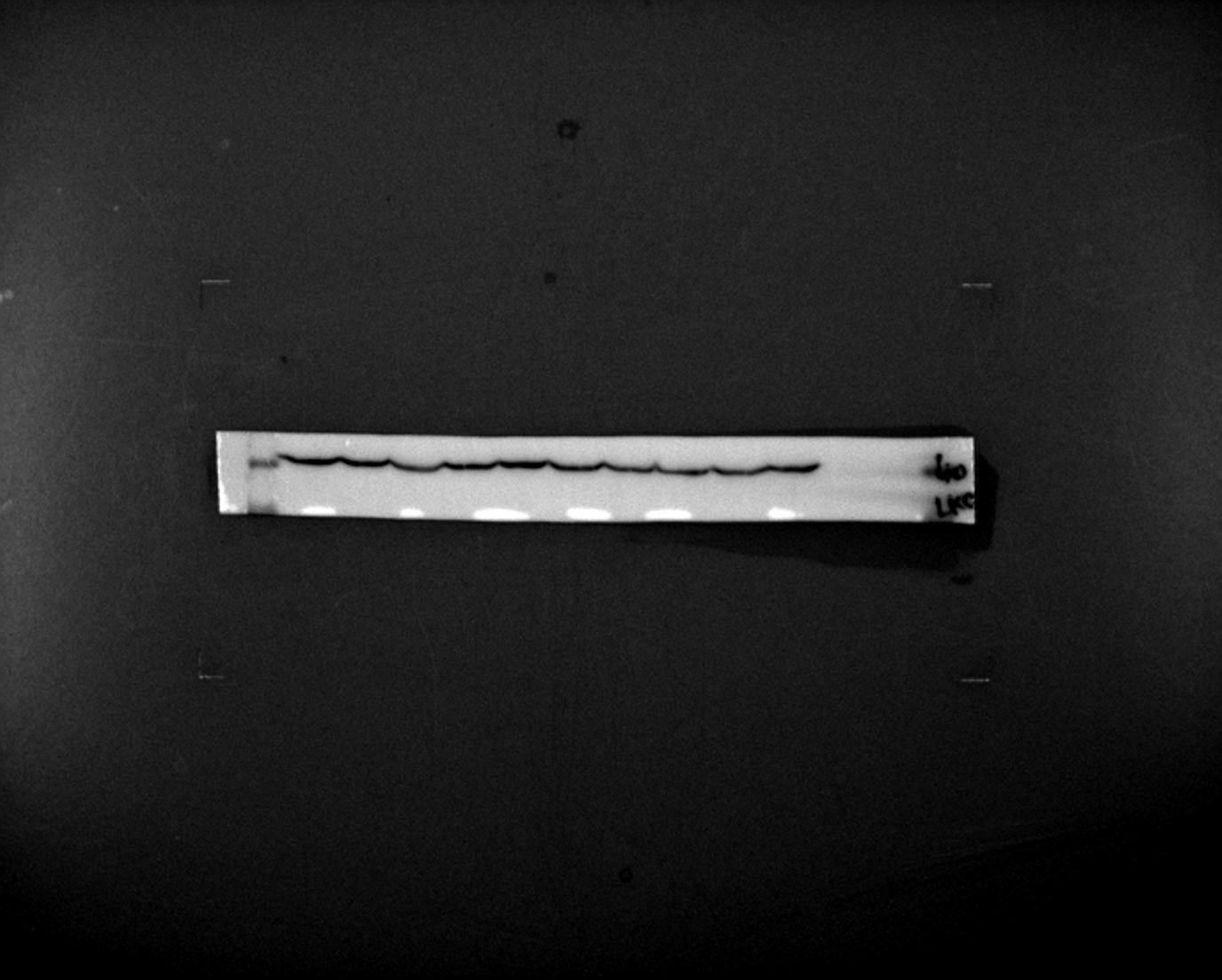

Supplement: Figure 7—source data 2. [file elife-96988-fig7-data2.zip › Figure 7-source data 2/PPARG in Snhg3-HKO mice/3/β-ACTIN.Tif]

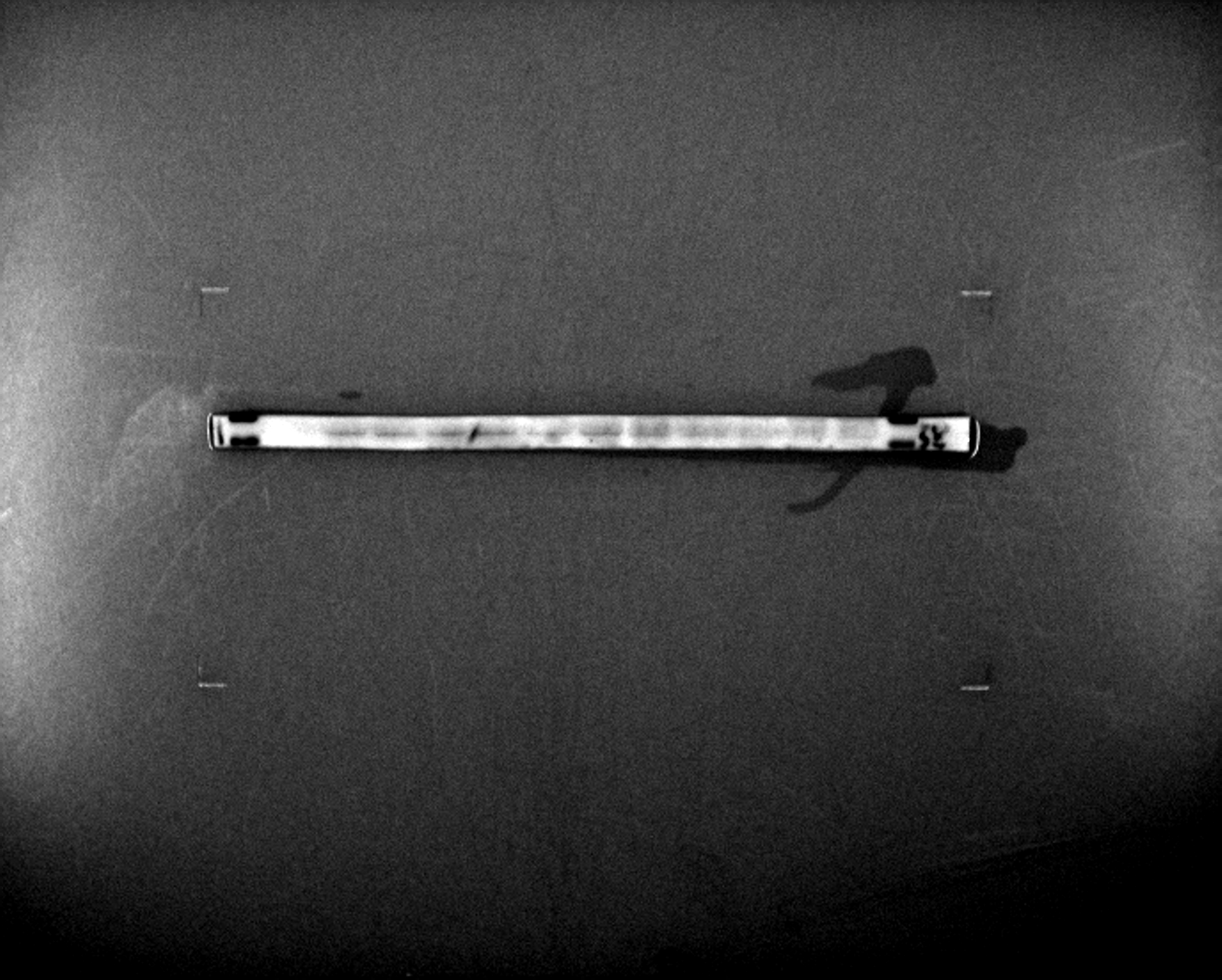

Supplement: Figure 7—source data 2. [file elife-96988-fig7-data2.zip › Figure 7-source data 2/PPARG in Snhg3-HKO mice/4/PPARG.Tif]

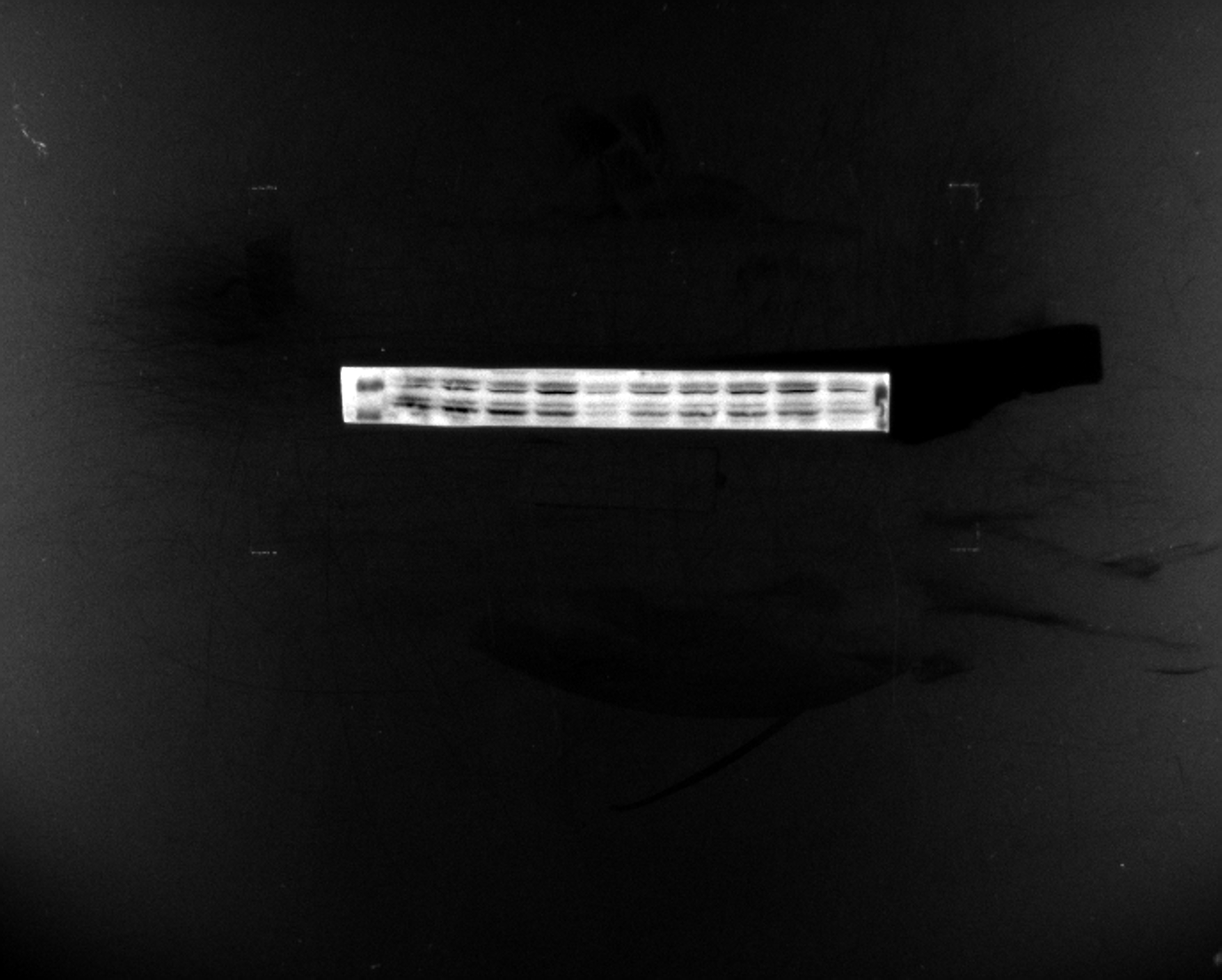

Supplement: Figure 7—source data 2. [file elife-96988-fig7-data2.zip › Figure 7-source data 2/PPARG in Snhg3-HKO mice/5/PPARG.tif]

Figure 7C

1.

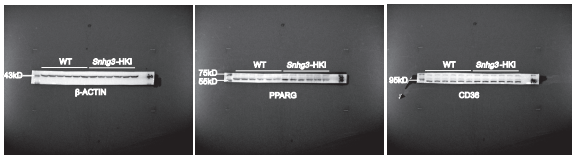

2.

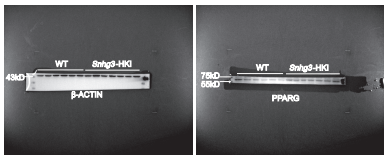

3.

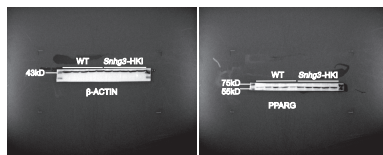

Supplement: Figure 7—source data 3. [file elife-96988-fig7-data3.pdf]

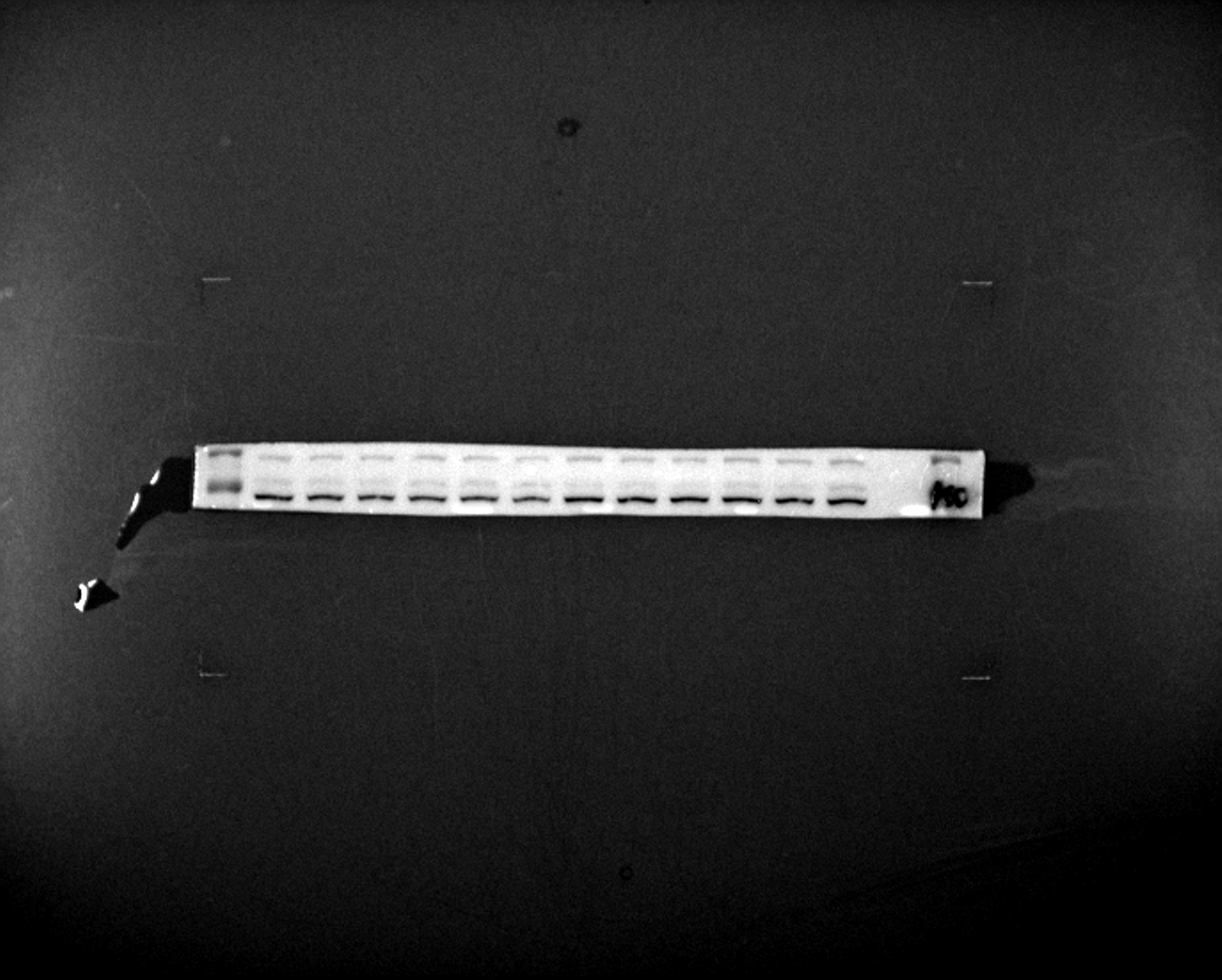

Supplement: Figure 7—source data 4. [file elife-96988-fig7-data4.zip › Figure 7-source data 4/1/CD36.Tif]

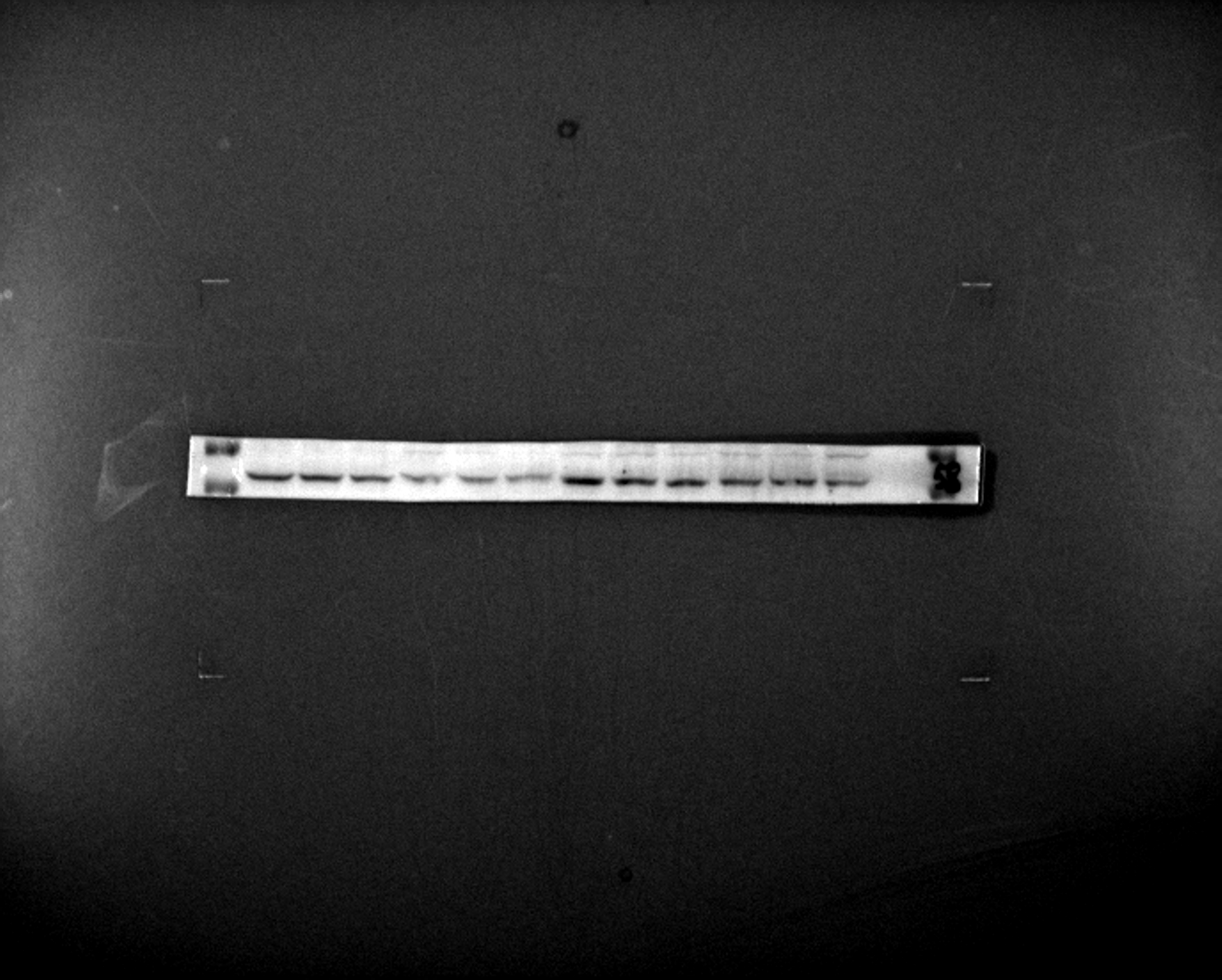

Supplement: Figure 7—source data 4. [file elife-96988-fig7-data4.zip › Figure 7-source data 4/1/PPARg.Tif]

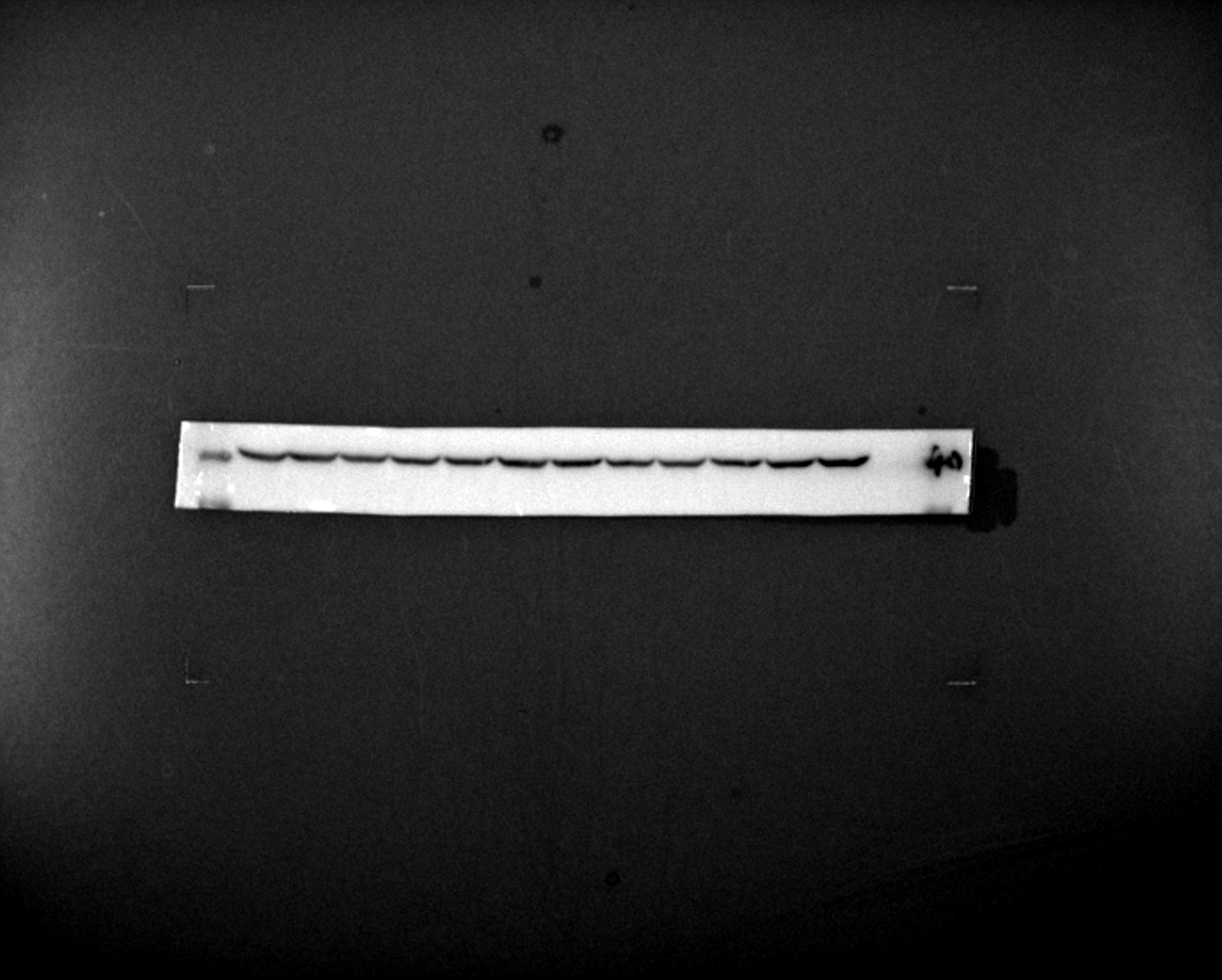

Supplement: Figure 7—source data 4. [file elife-96988-fig7-data4.zip › Figure 7-source data 4/1/β-ACTIN.Tif]

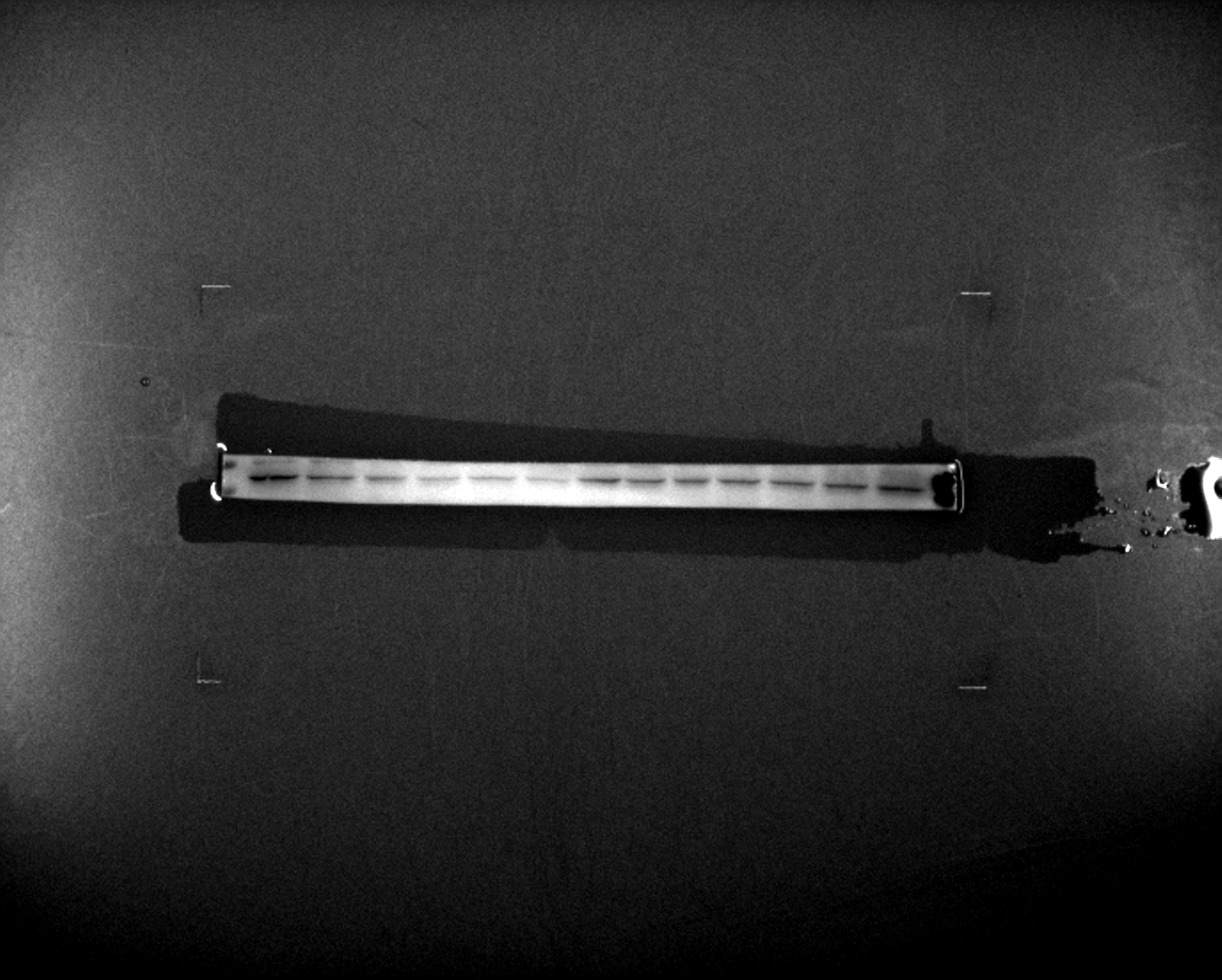

Supplement: Figure 7—source data 4. [file elife-96988-fig7-data4.zip › Figure 7-source data 4/2/PPARG.Tif]

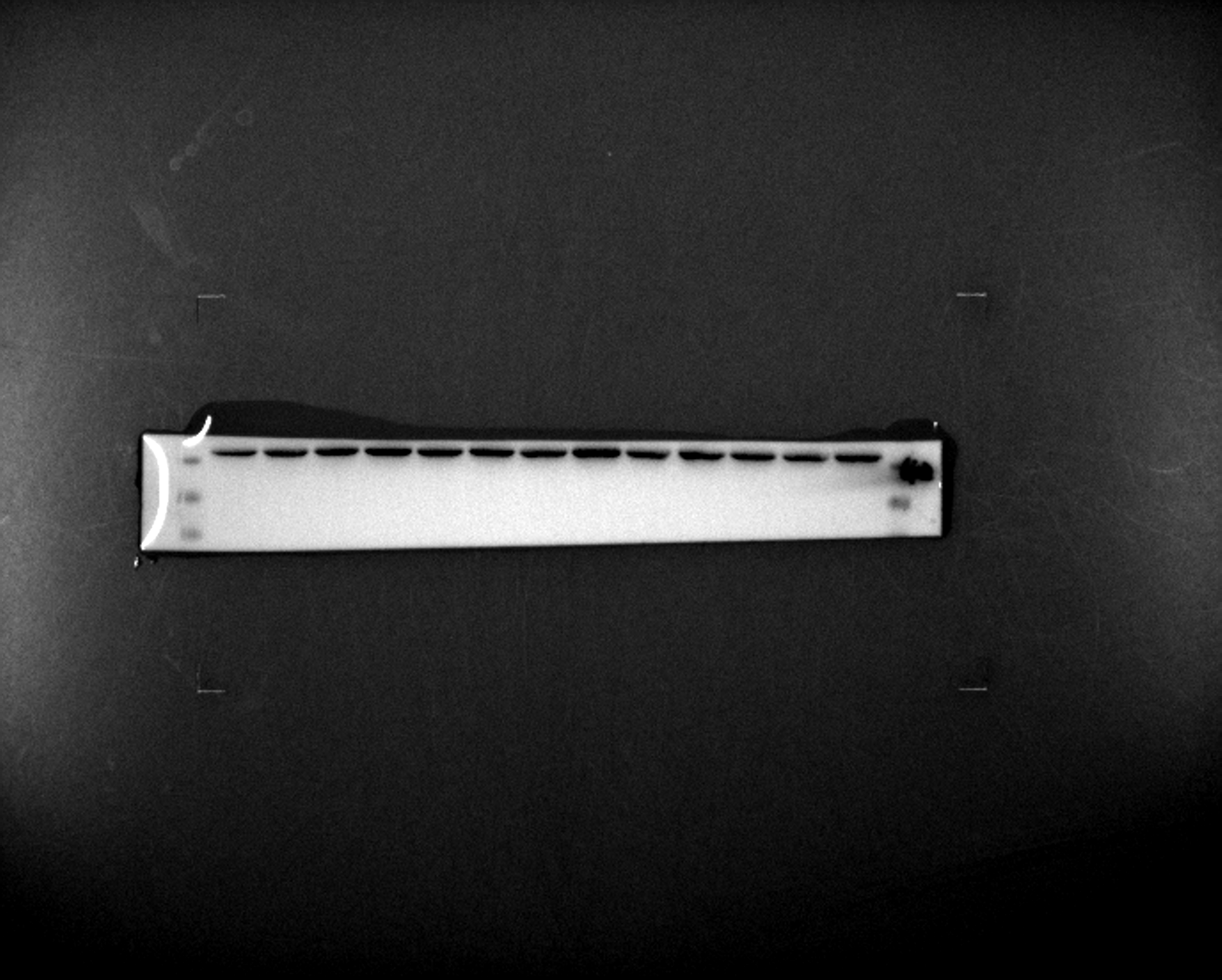

Supplement: Figure 7—source data 4. [file elife-96988-fig7-data4.zip › Figure 7-source data 4/2/β-ACTIN.Tif]

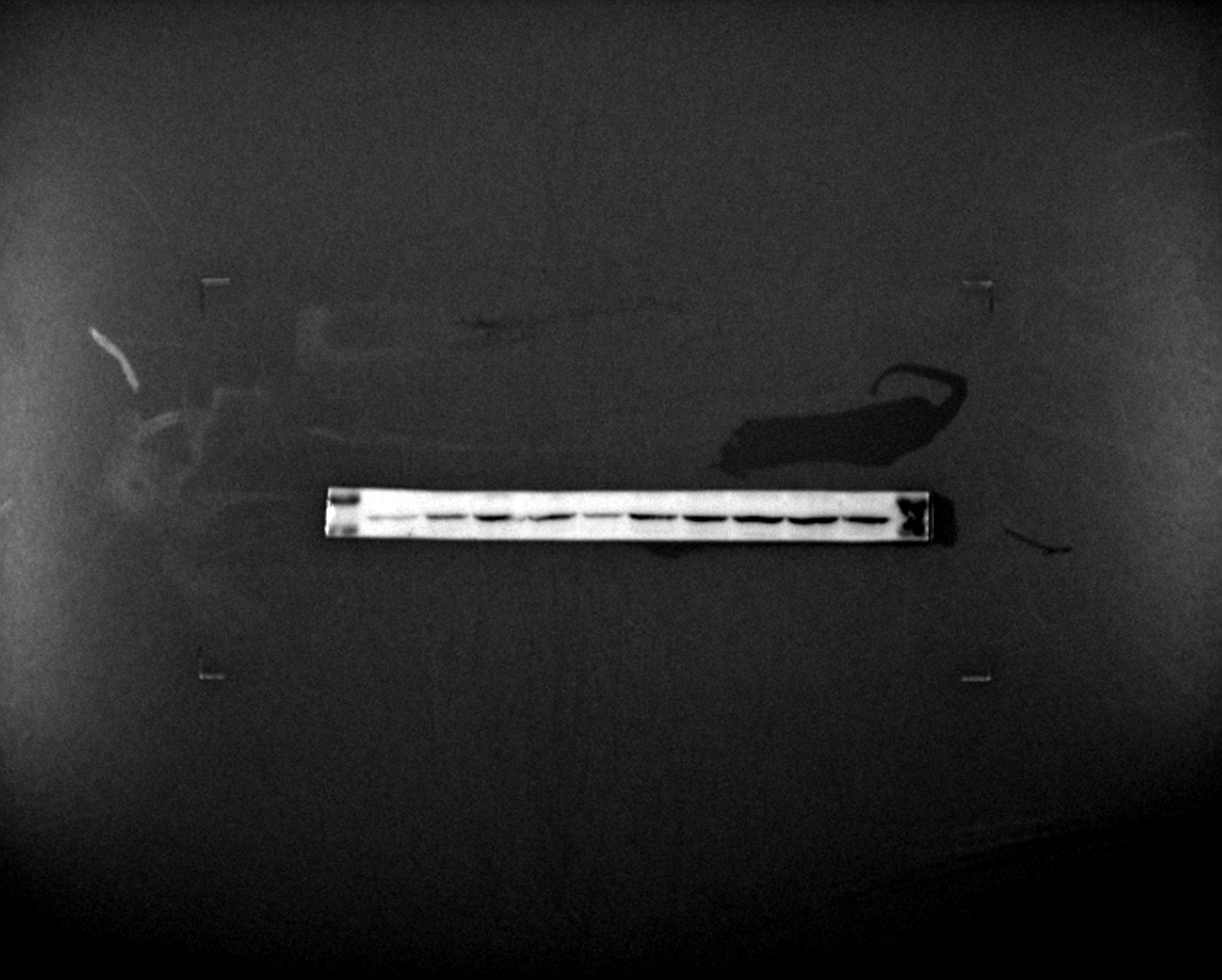

Supplement: Figure 7—source data 4. [file elife-96988-fig7-data4.zip › Figure 7-source data 4/3/PPARG.Tif]

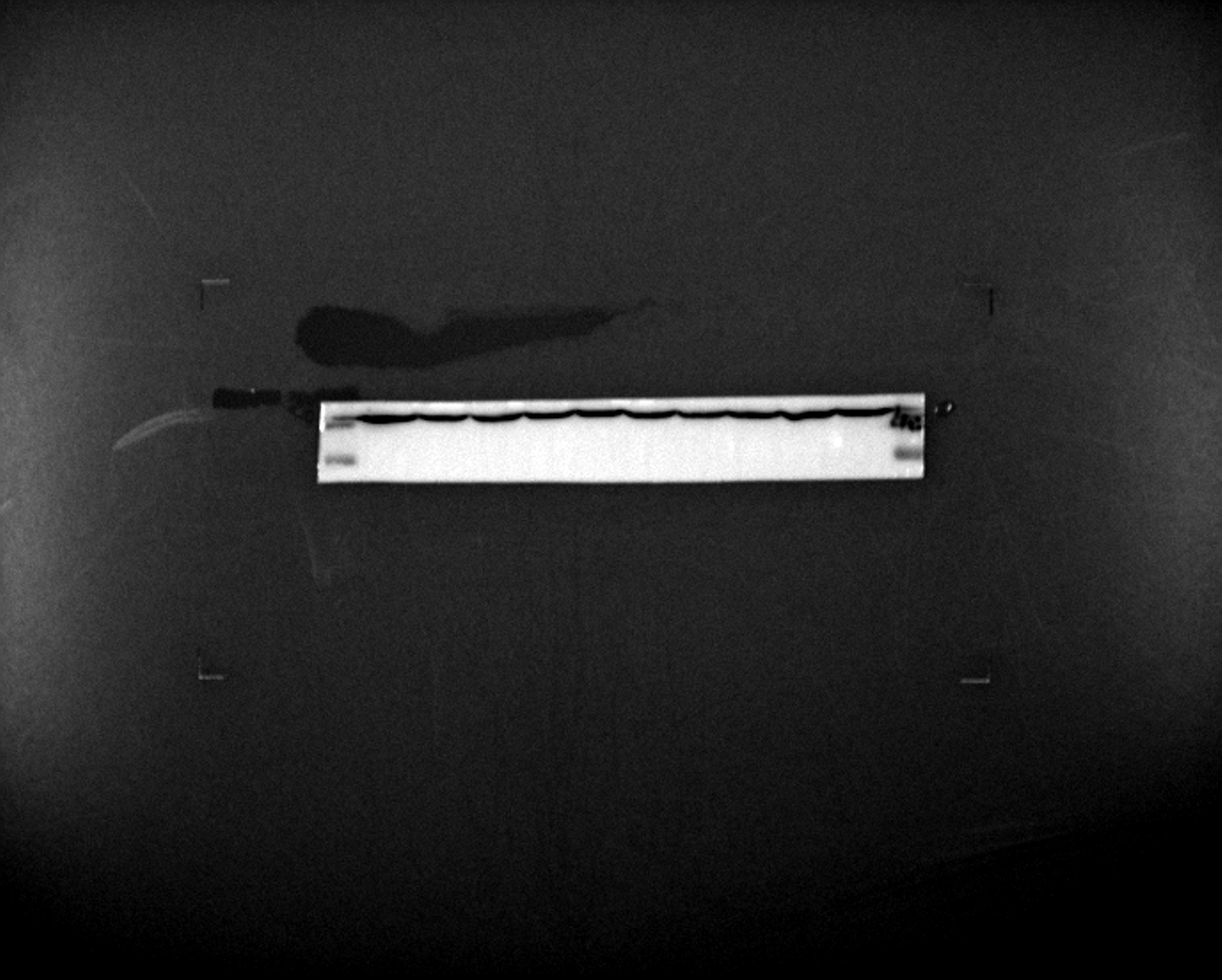

Supplement: Figure 7—source data 4. [file elife-96988-fig7-data4.zip › Figure 7-source data 4/3/β-ACTIN.Tif]

Figure 7F

1.

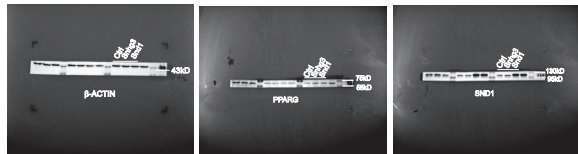

2.

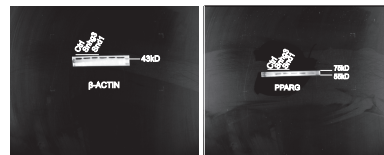

3.

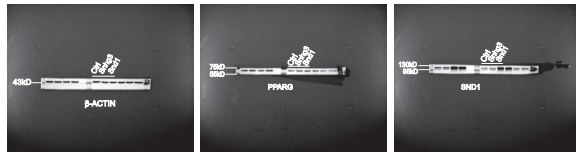

4.

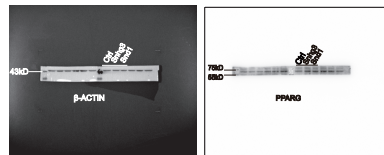

Supplement: Figure 7—source data 5. [file elife-96988-fig7-data5.pdf]

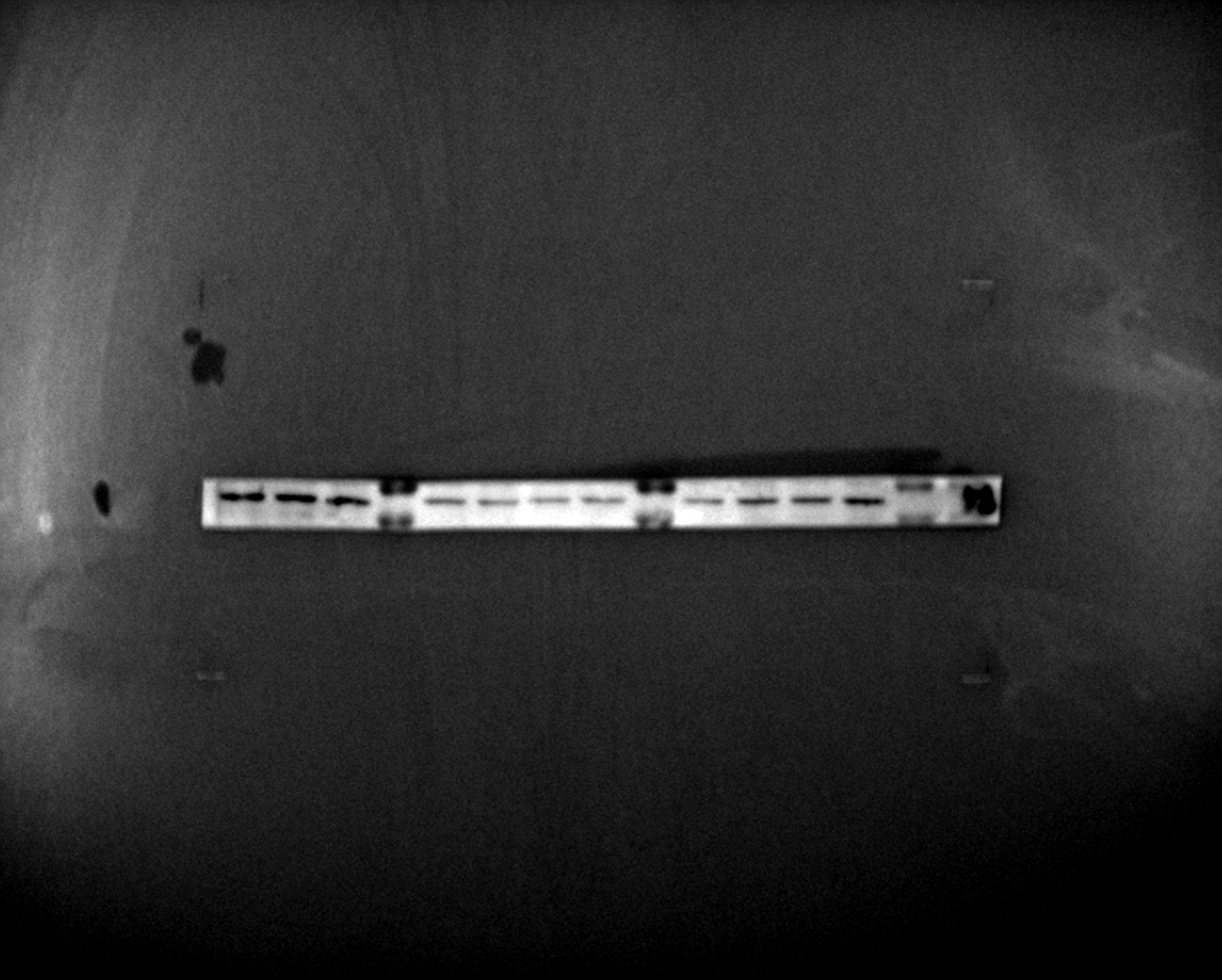

Supplement: Figure 7—source data 6. [file elife-96988-fig7-data6.zip › Figure 7-source data 6/1/PPARG.Tif]

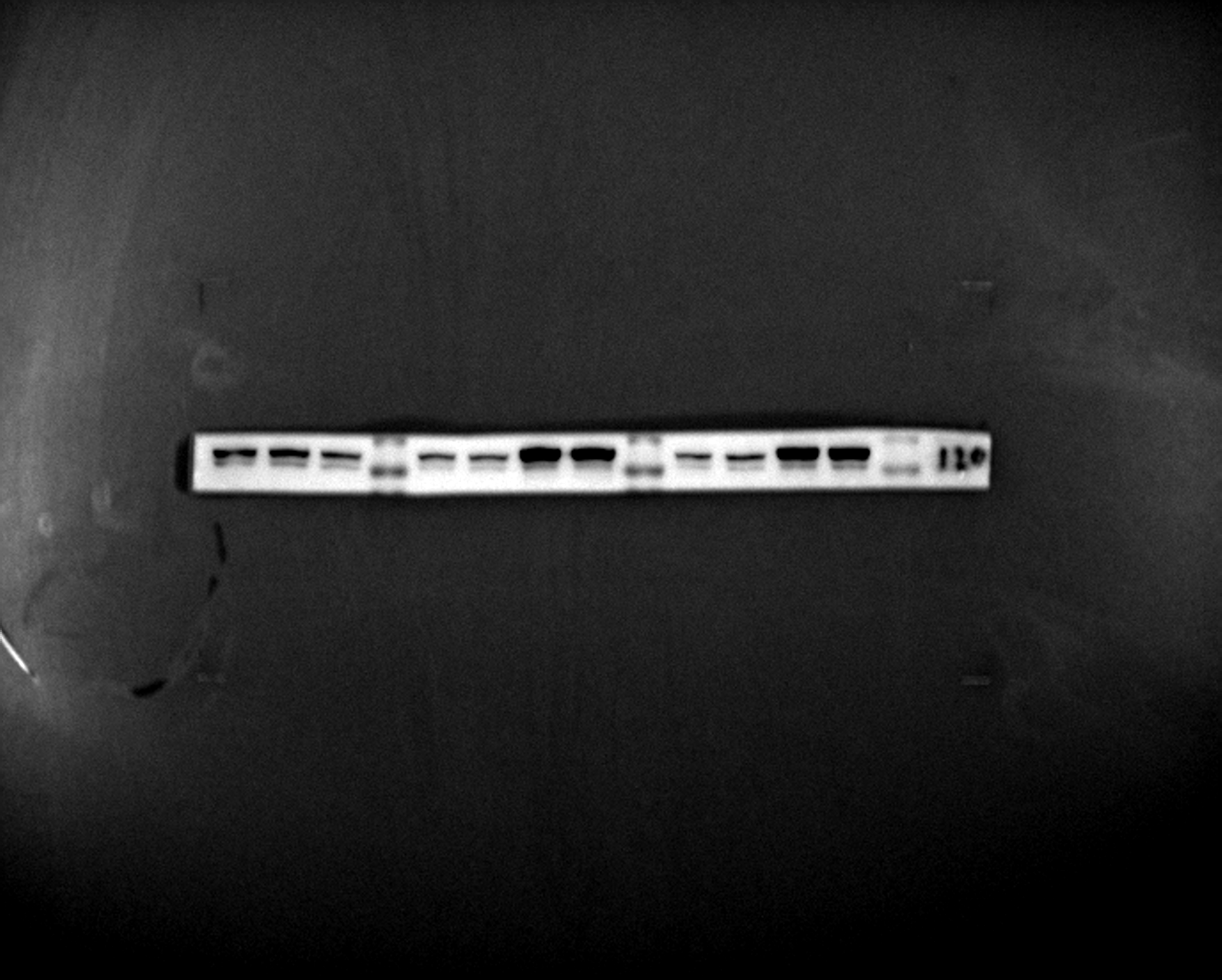

Supplement: Figure 7—source data 6. [file elife-96988-fig7-data6.zip › Figure 7-source data 6/1/SND1.Tif]

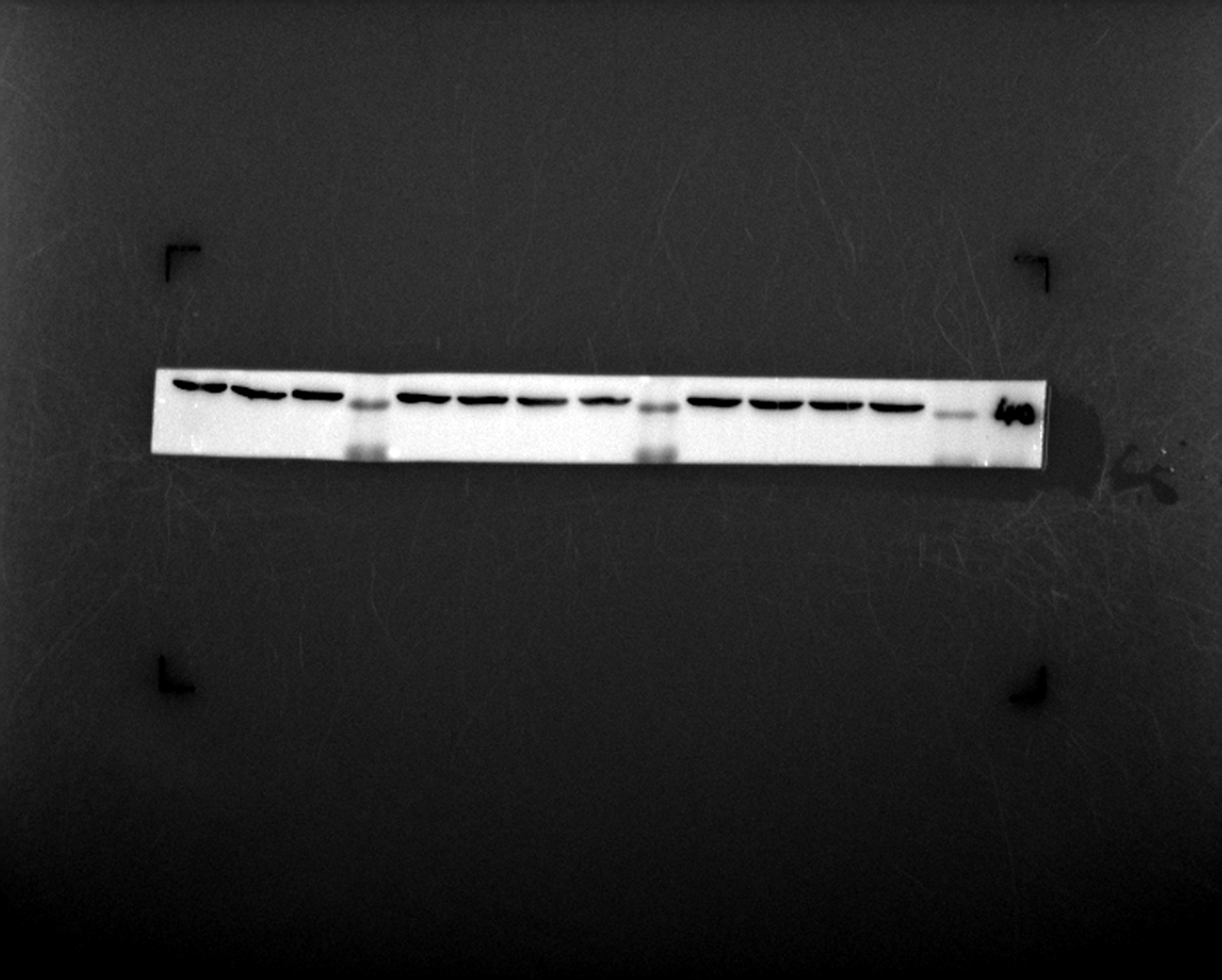

Supplement: Figure 7—source data 6. [file elife-96988-fig7-data6.zip › Figure 7-source data 6/1/β-ACTIN.tif]

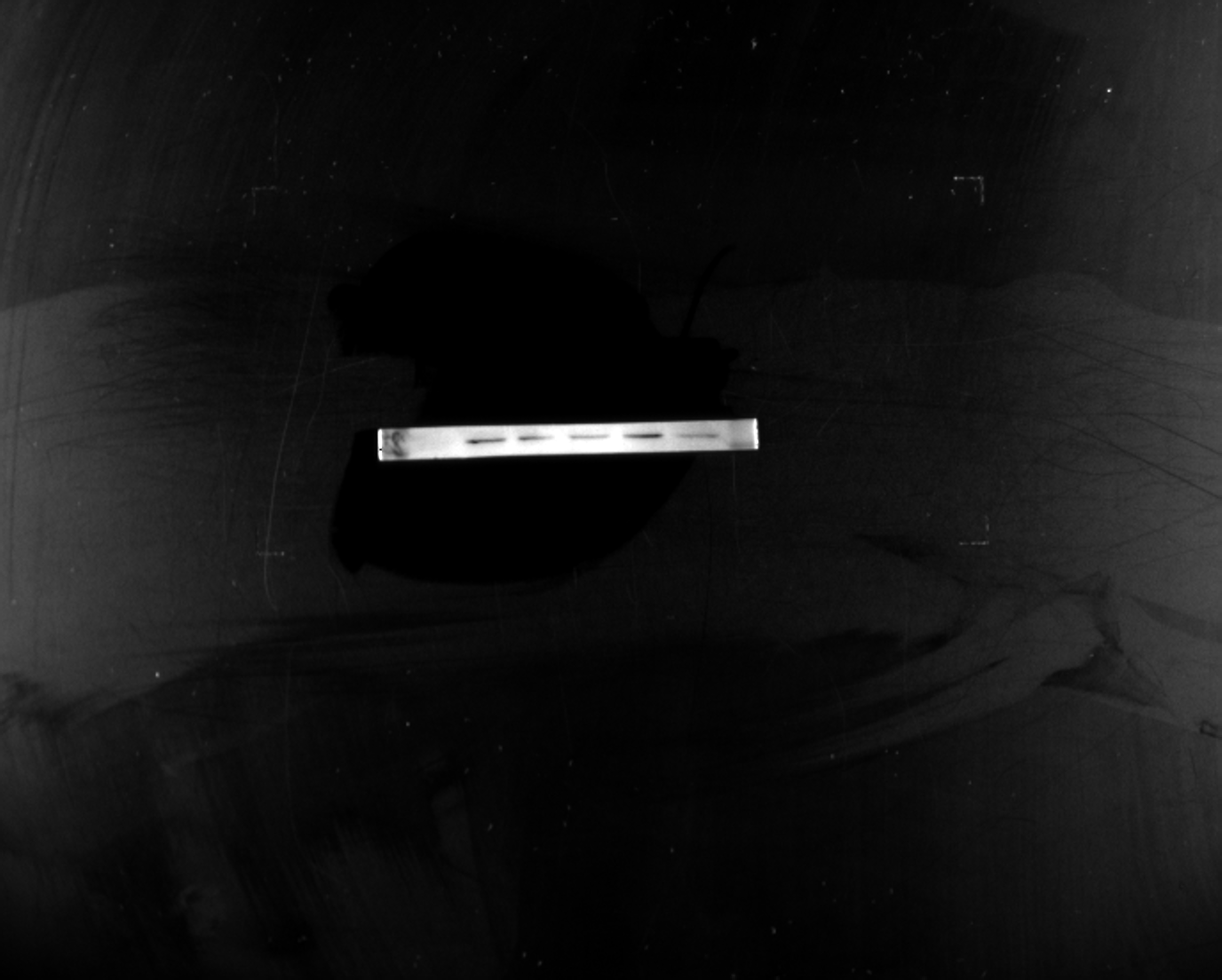

Supplement: Figure 7—source data 6. [file elife-96988-fig7-data6.zip › Figure 7-source data 6/2/PPARG.tif]

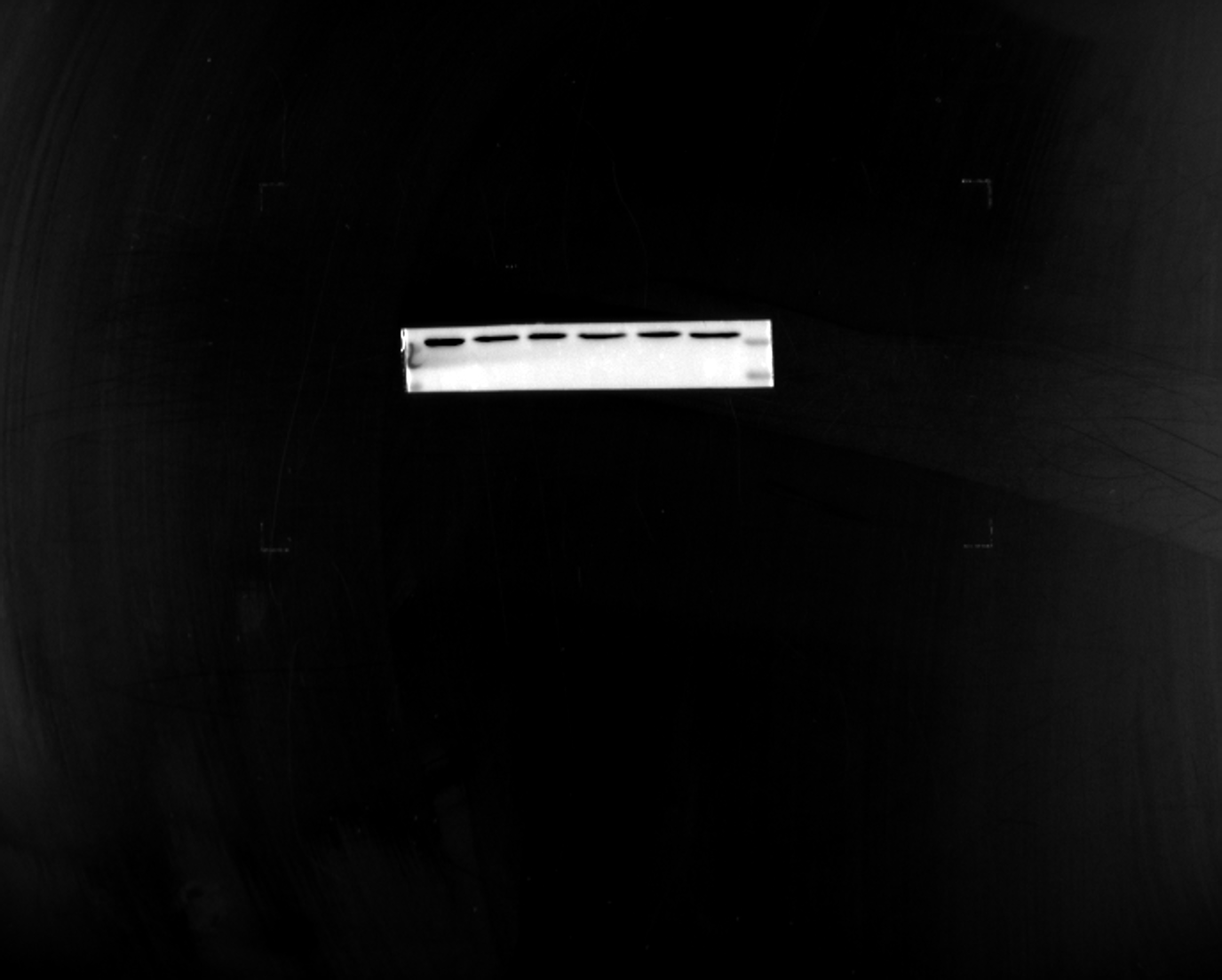

Supplement: Figure 7—source data 6. [file elife-96988-fig7-data6.zip › Figure 7-source data 6/2/β-ACTIN.tif]

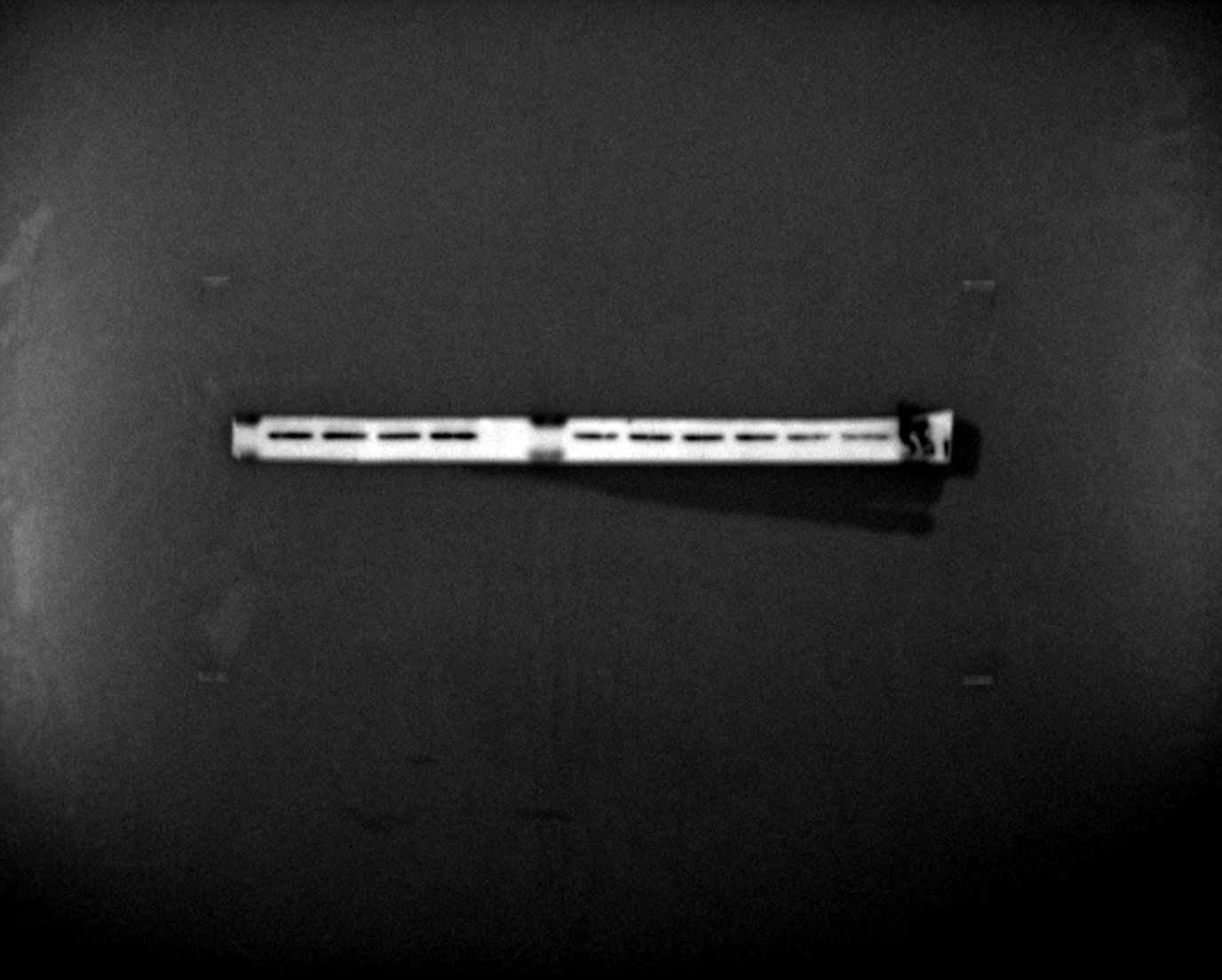

Supplement: Figure 7—source data 6. [file elife-96988-fig7-data6.zip › Figure 7-source data 6/3/PPARG.Tif]

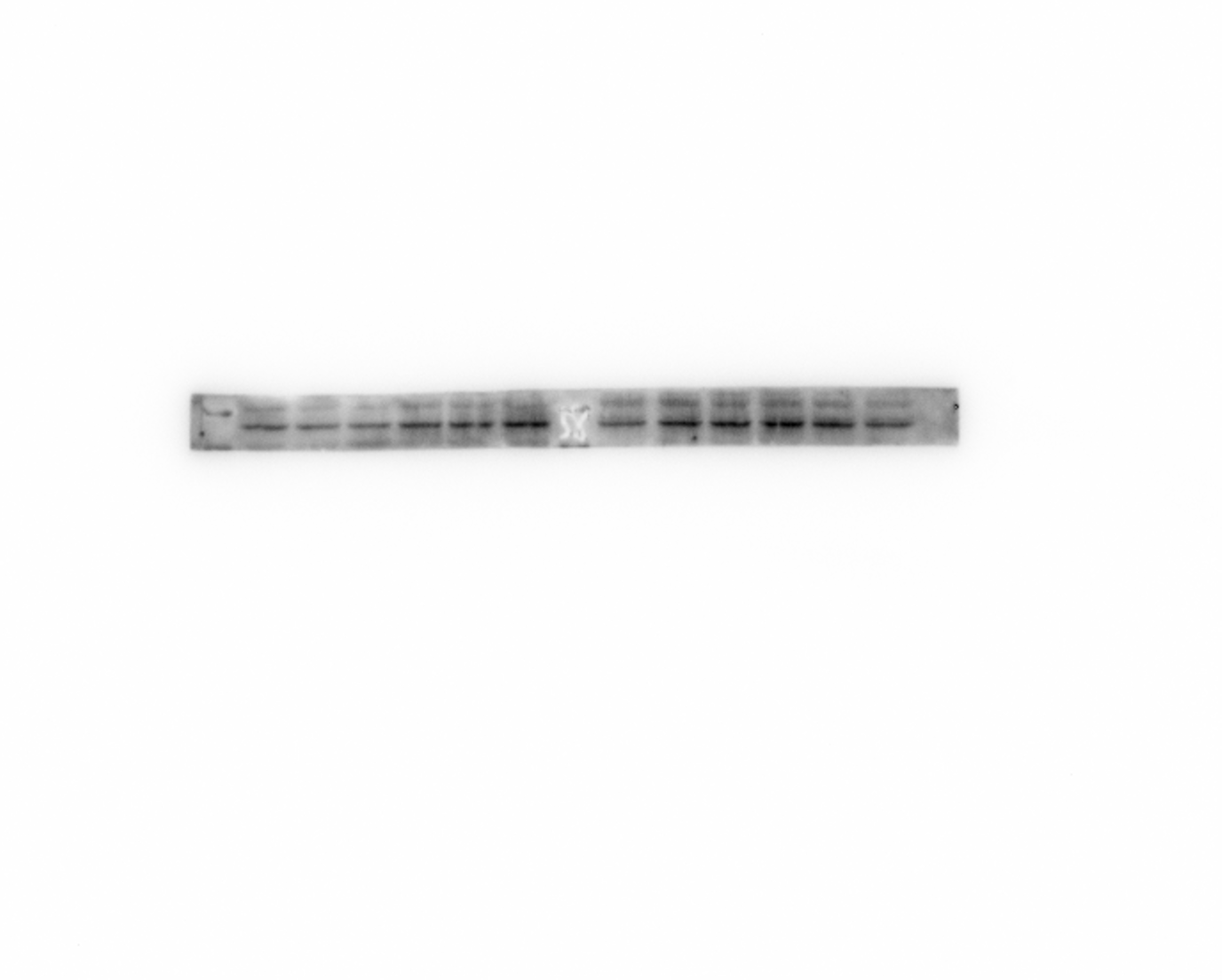

Supplement: Figure 7—source data 6. [file elife-96988-fig7-data6.zip › Figure 7-source data 6/4/PPARG.Tif]

Figure 7G

MPH

1.

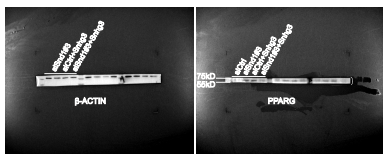

2.

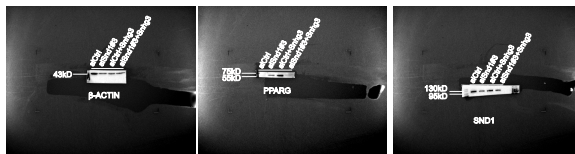

3.

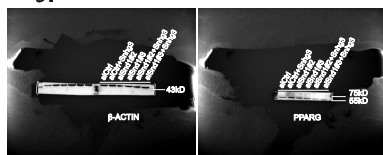

4.

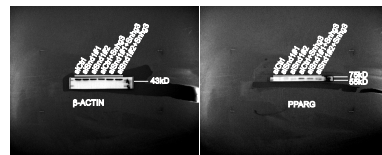

Hepa1-6 cells

1.

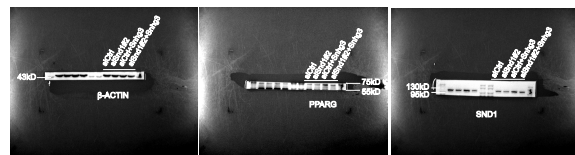

2.

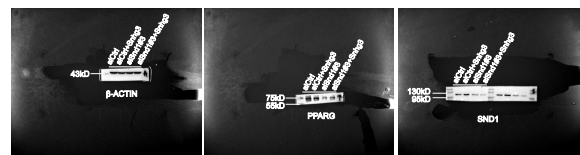

3.

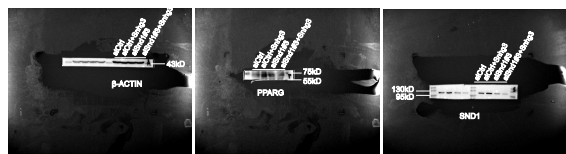

4.

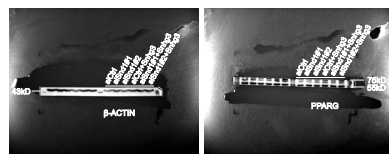

5.

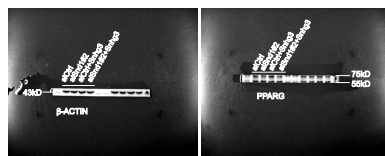

6.

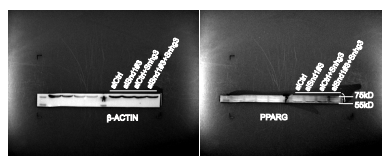

Supplement: Figure 7—source data 7. [file elife-96988-fig7-data7.pdf]

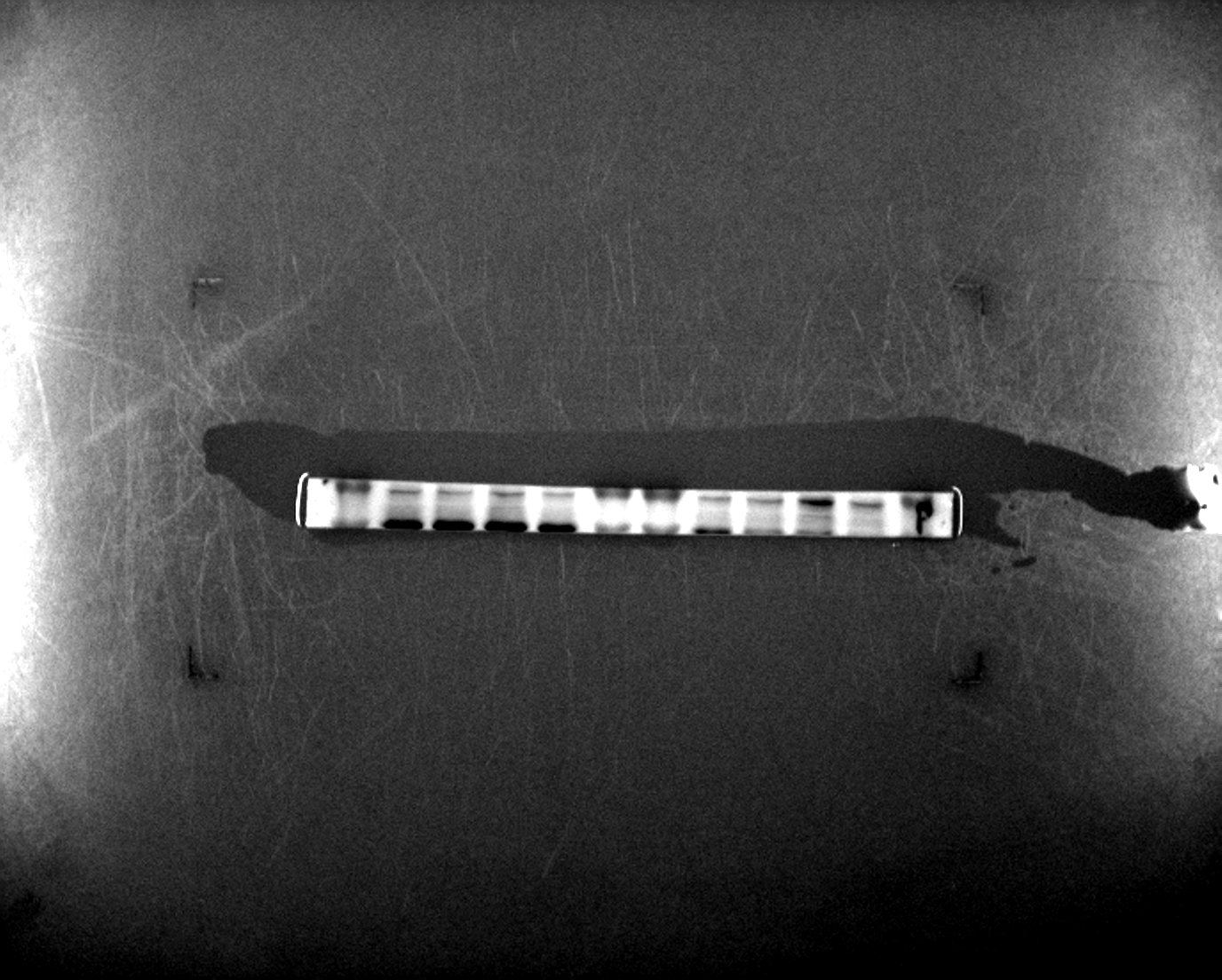

Supplement: Figure 7—source data 8. [file elife-96988-fig7-data8.zip › Figure 7-source data 8/Hepa1-6/1/PPARG.Tif]

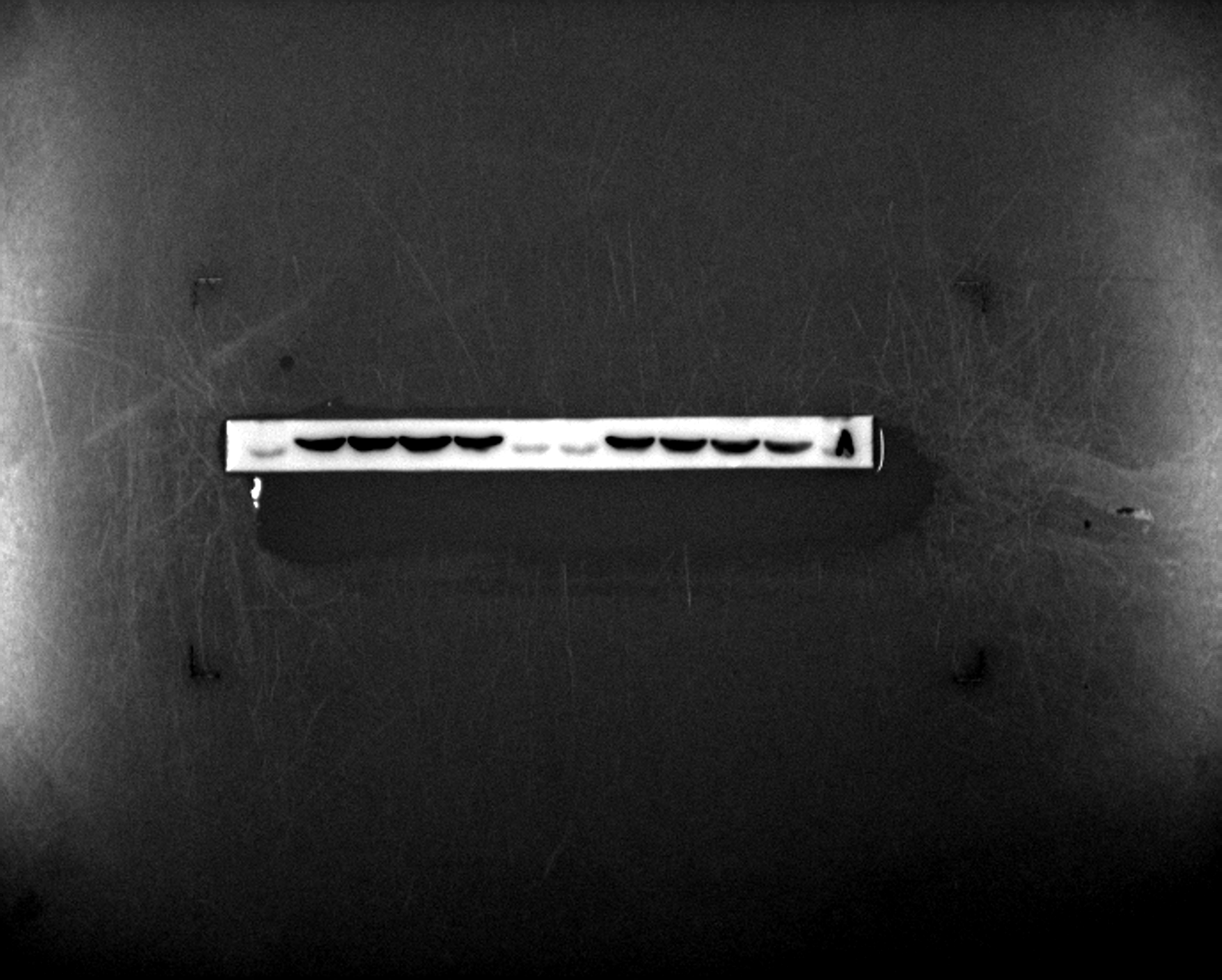

Supplement: Figure 7—source data 8. [file elife-96988-fig7-data8.zip › Figure 7-source data 8/Hepa1-6/1/β-ACTIN.Tif]

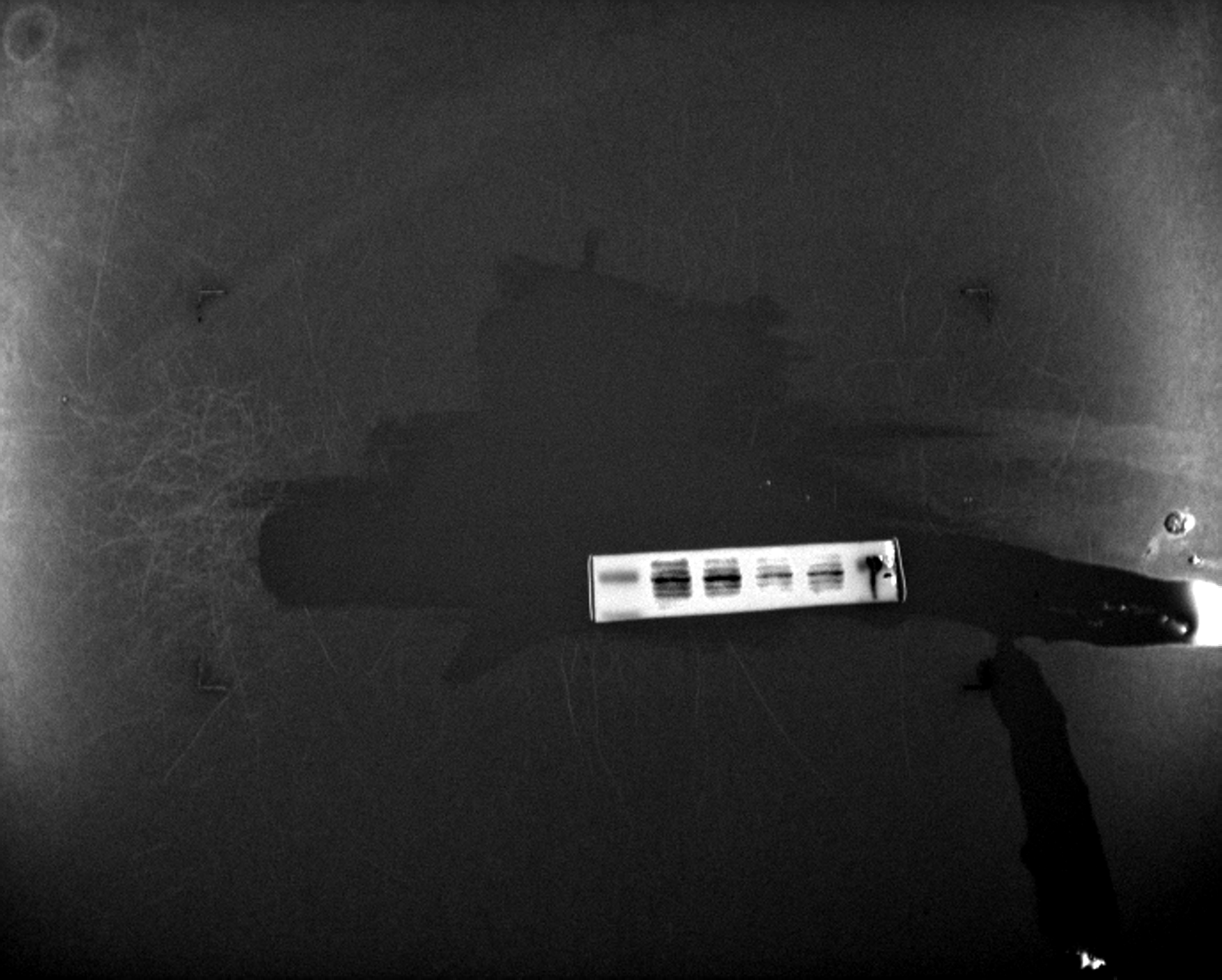

Supplement: Figure 7—source data 8. [file elife-96988-fig7-data8.zip › Figure 7-source data 8/Hepa1-6/2/PPARG.Tif]

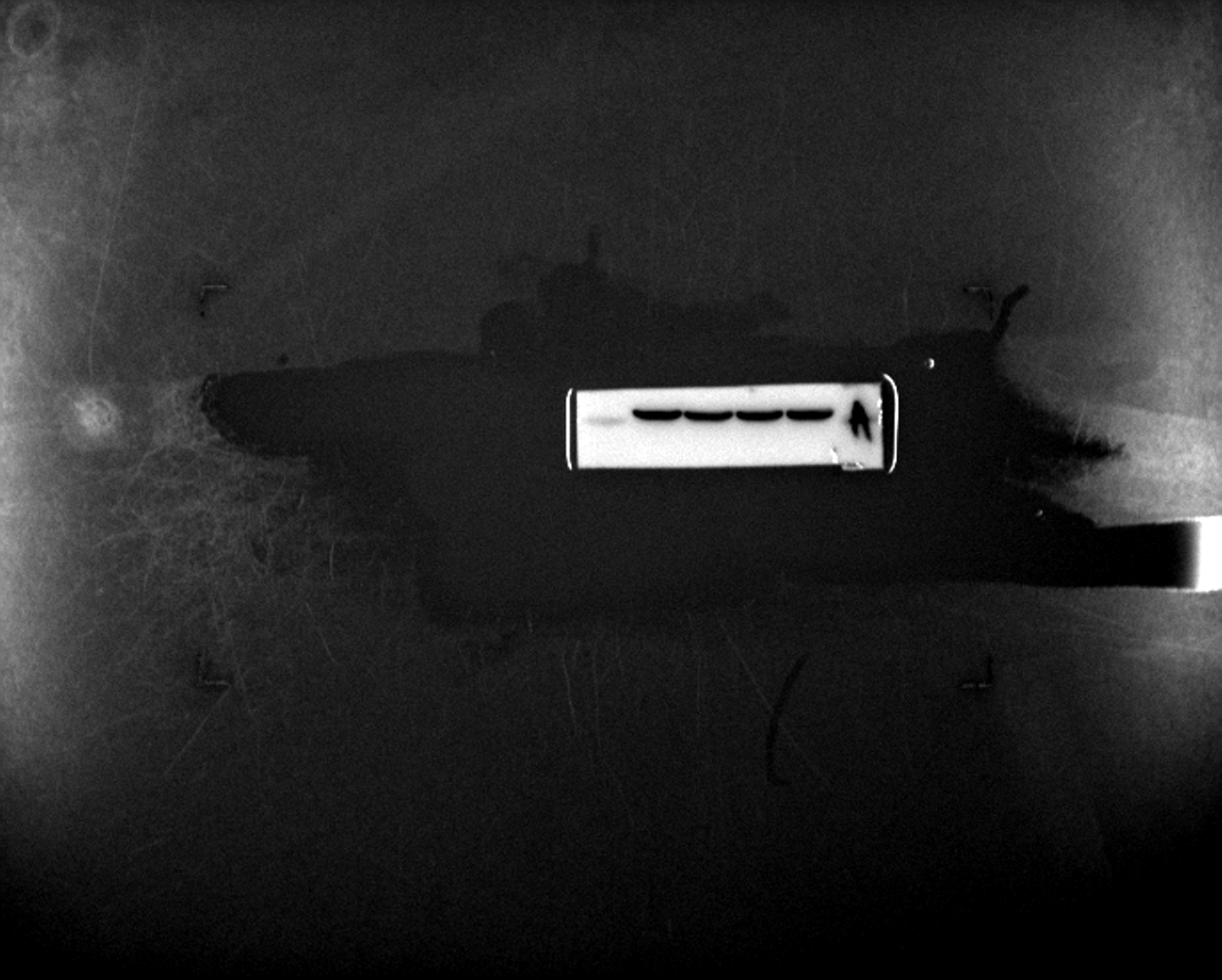

Supplement: Figure 7—source data 8. [file elife-96988-fig7-data8.zip › Figure 7-source data 8/Hepa1-6/2/β-ACTIN.Tif]

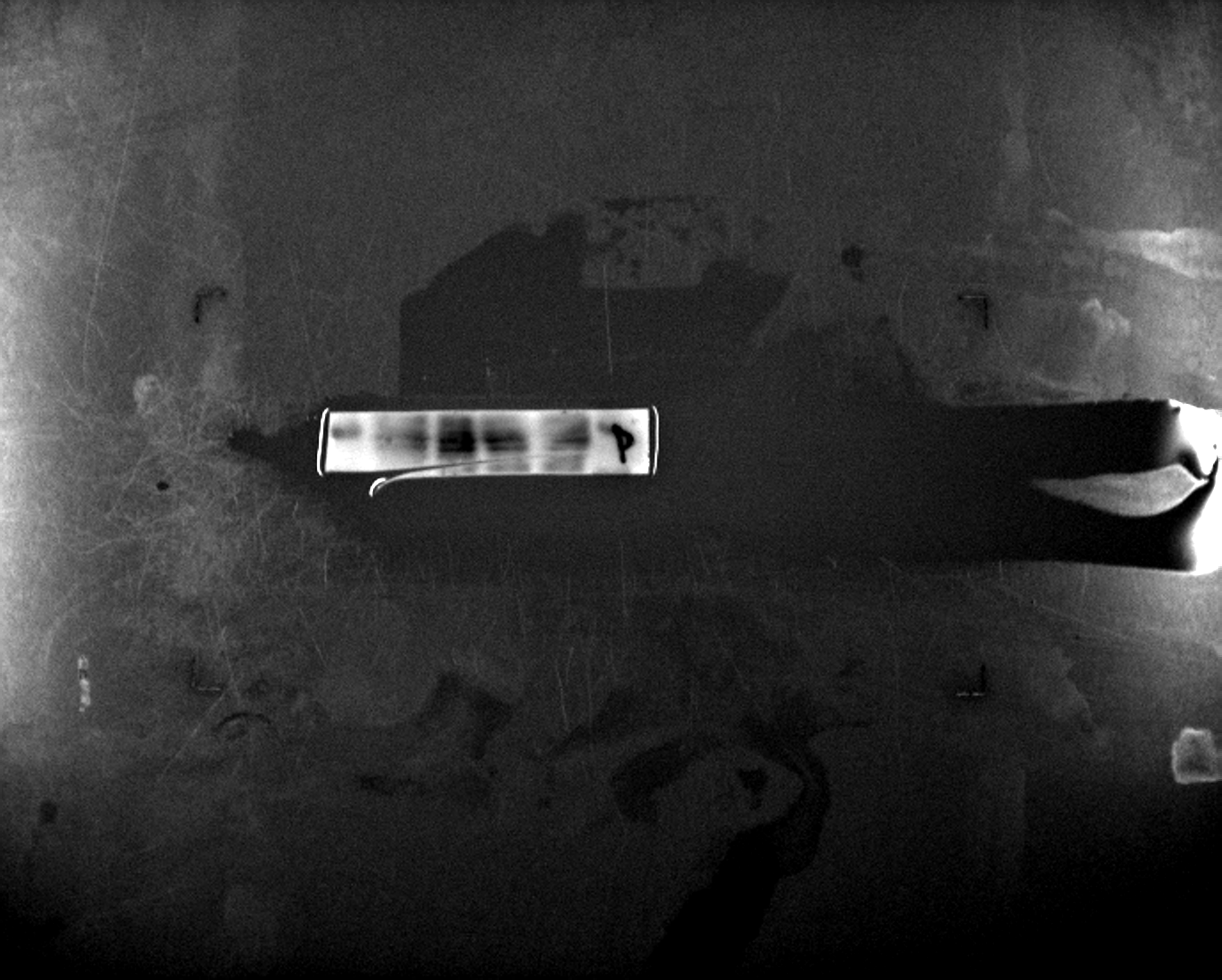

Supplement: Figure 7—source data 8. [file elife-96988-fig7-data8.zip › Figure 7-source data 8/Hepa1-6/3/PPARG.Tif]

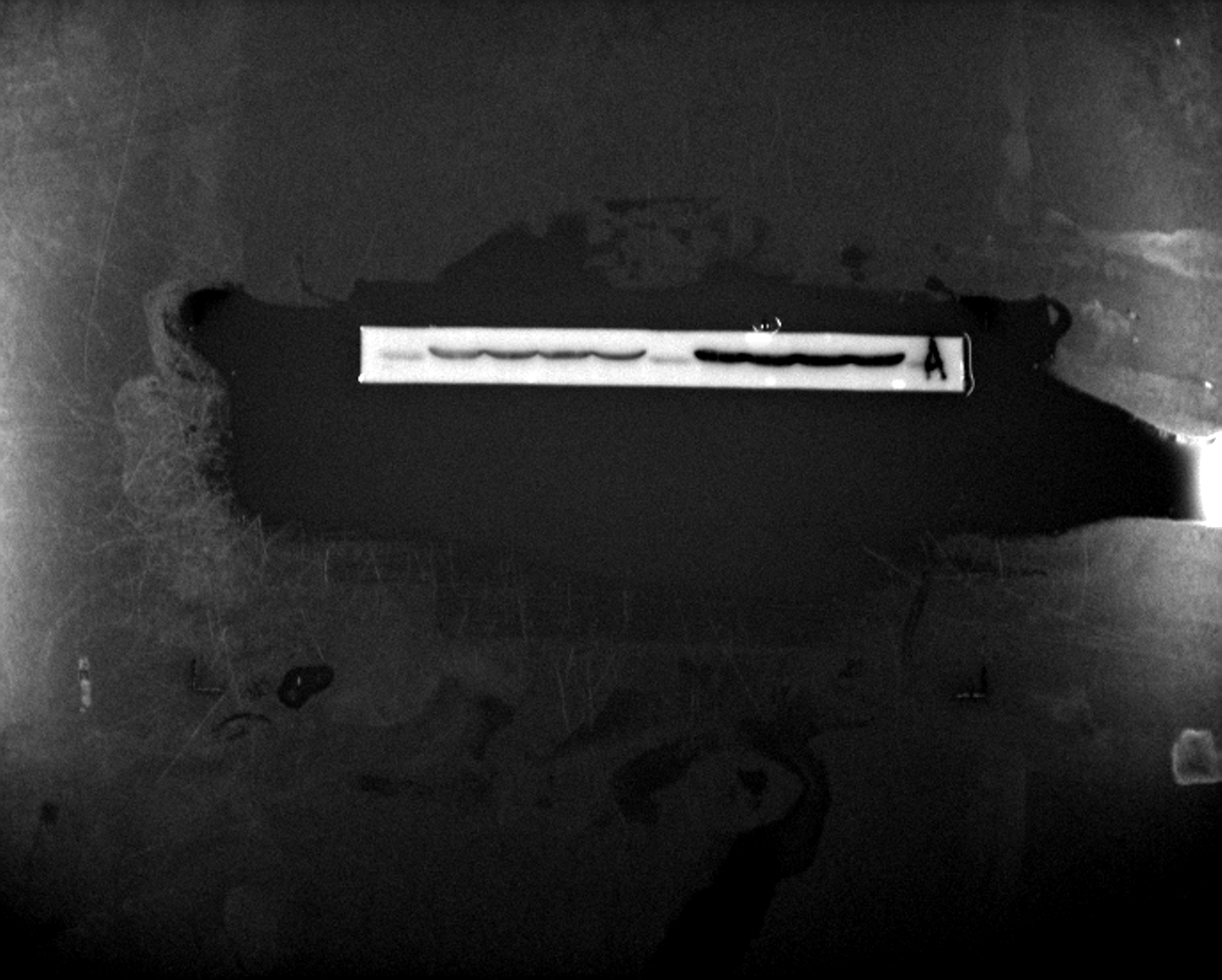

Supplement: Figure 7—source data 8. [file elife-96988-fig7-data8.zip › Figure 7-source data 8/Hepa1-6/3/β-ACTIN.Tif]

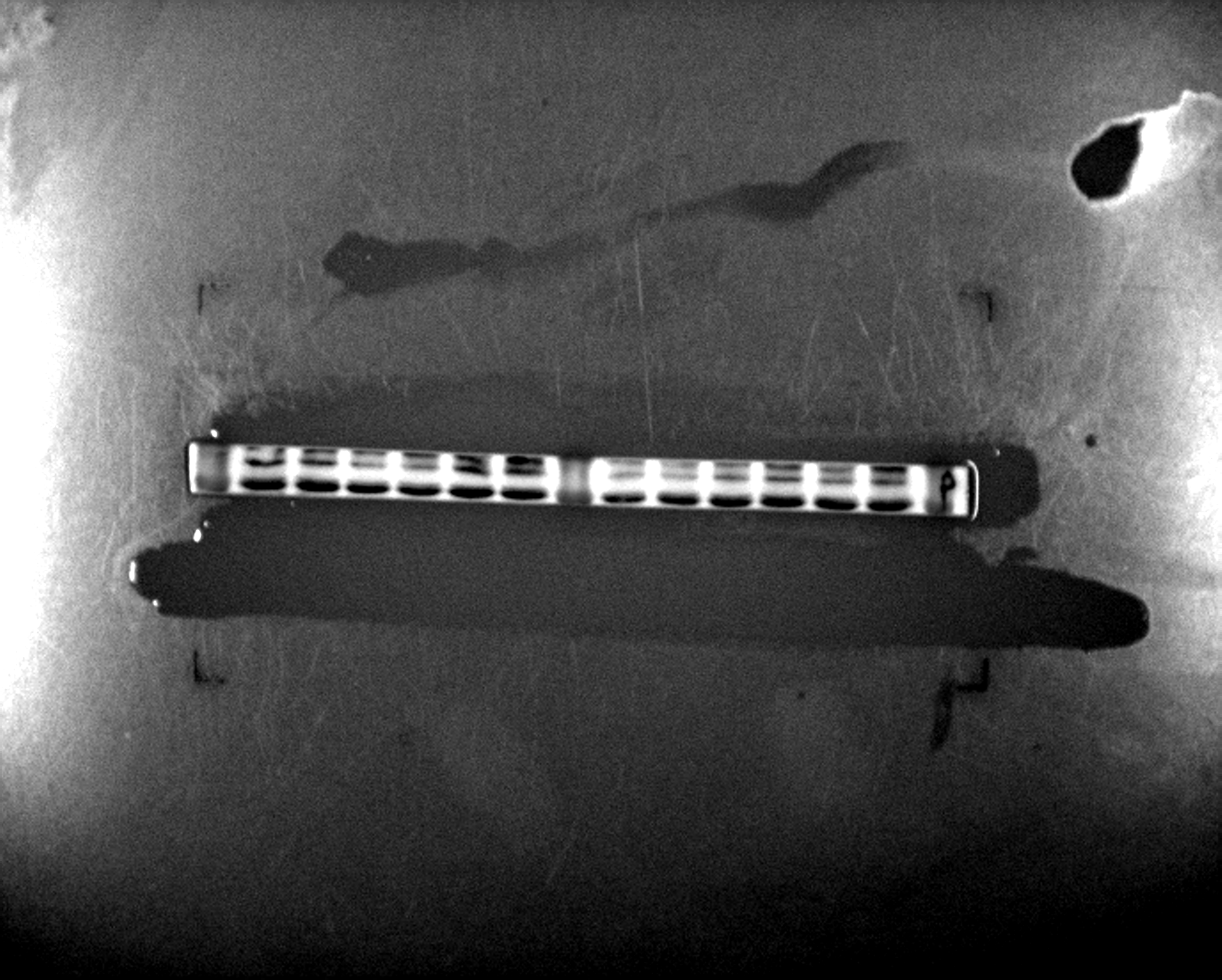

Supplement: Figure 7—source data 8. [file elife-96988-fig7-data8.zip › Figure 7-source data 8/Hepa1-6/4/PPARG.Tif]

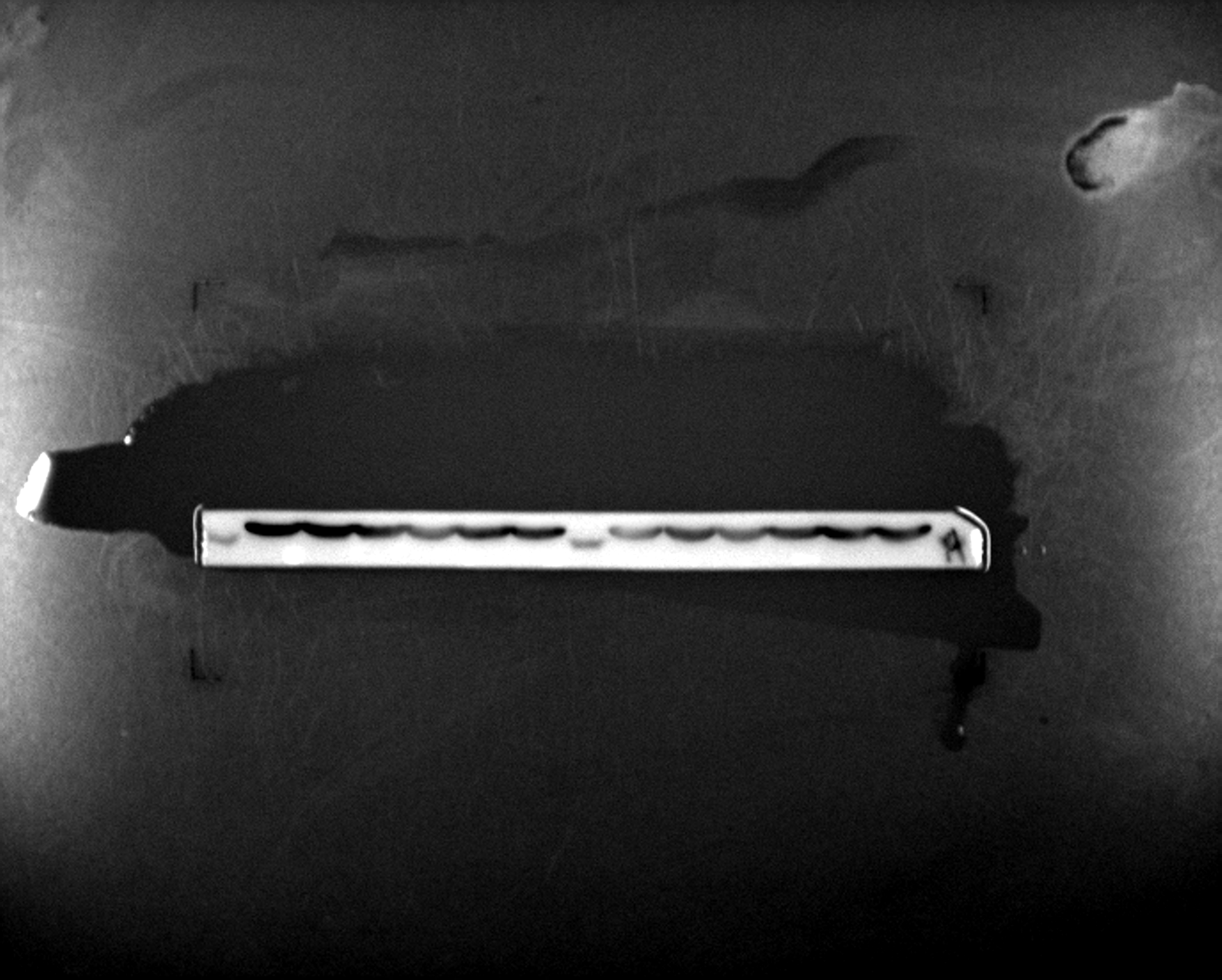

Supplement: Figure 7—source data 8. [file elife-96988-fig7-data8.zip › Figure 7-source data 8/Hepa1-6/4/β-ACTIN.Tif]

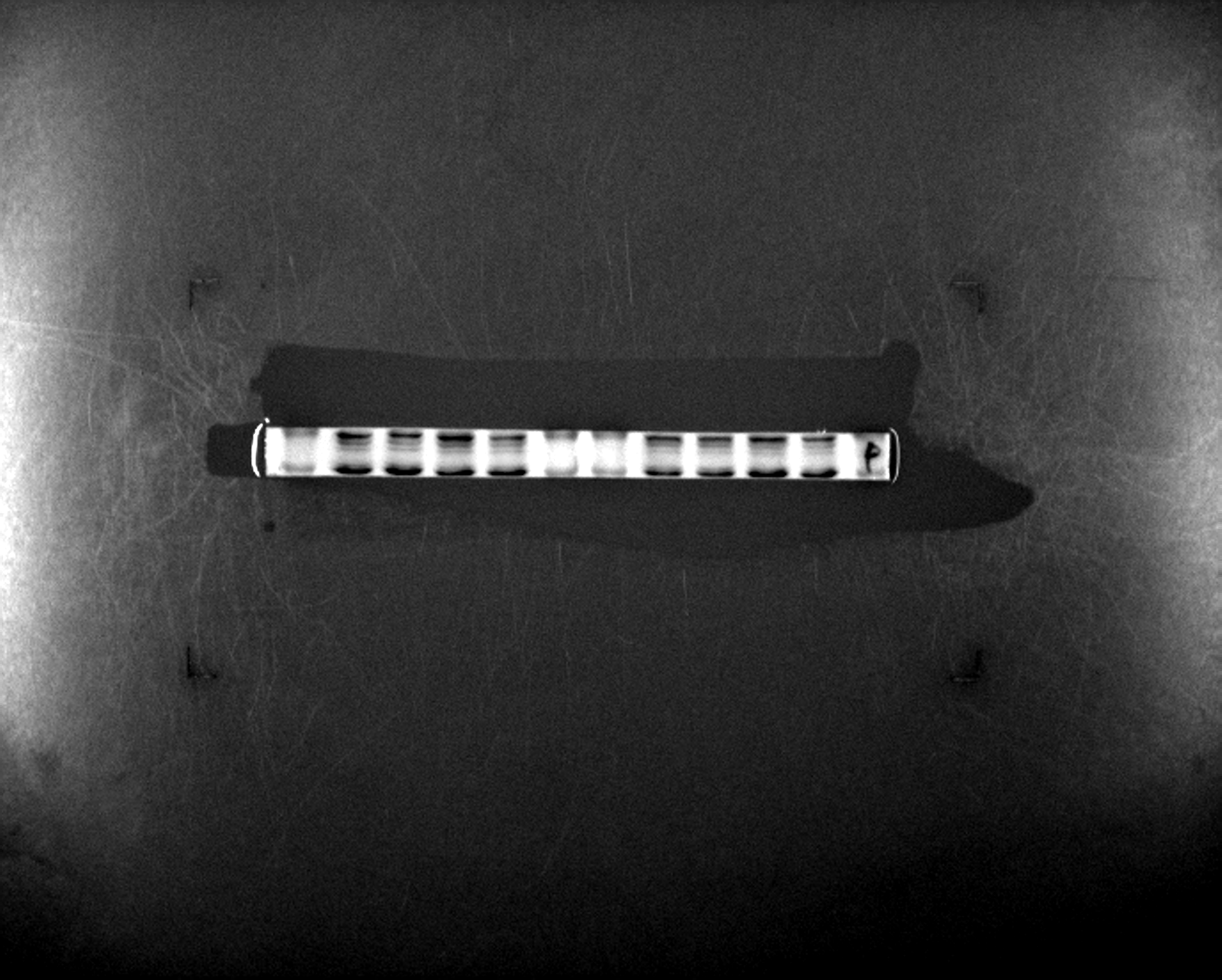

Supplement: Figure 7—source data 8. [file elife-96988-fig7-data8.zip › Figure 7-source data 8/Hepa1-6/5/PPARG.Tif]

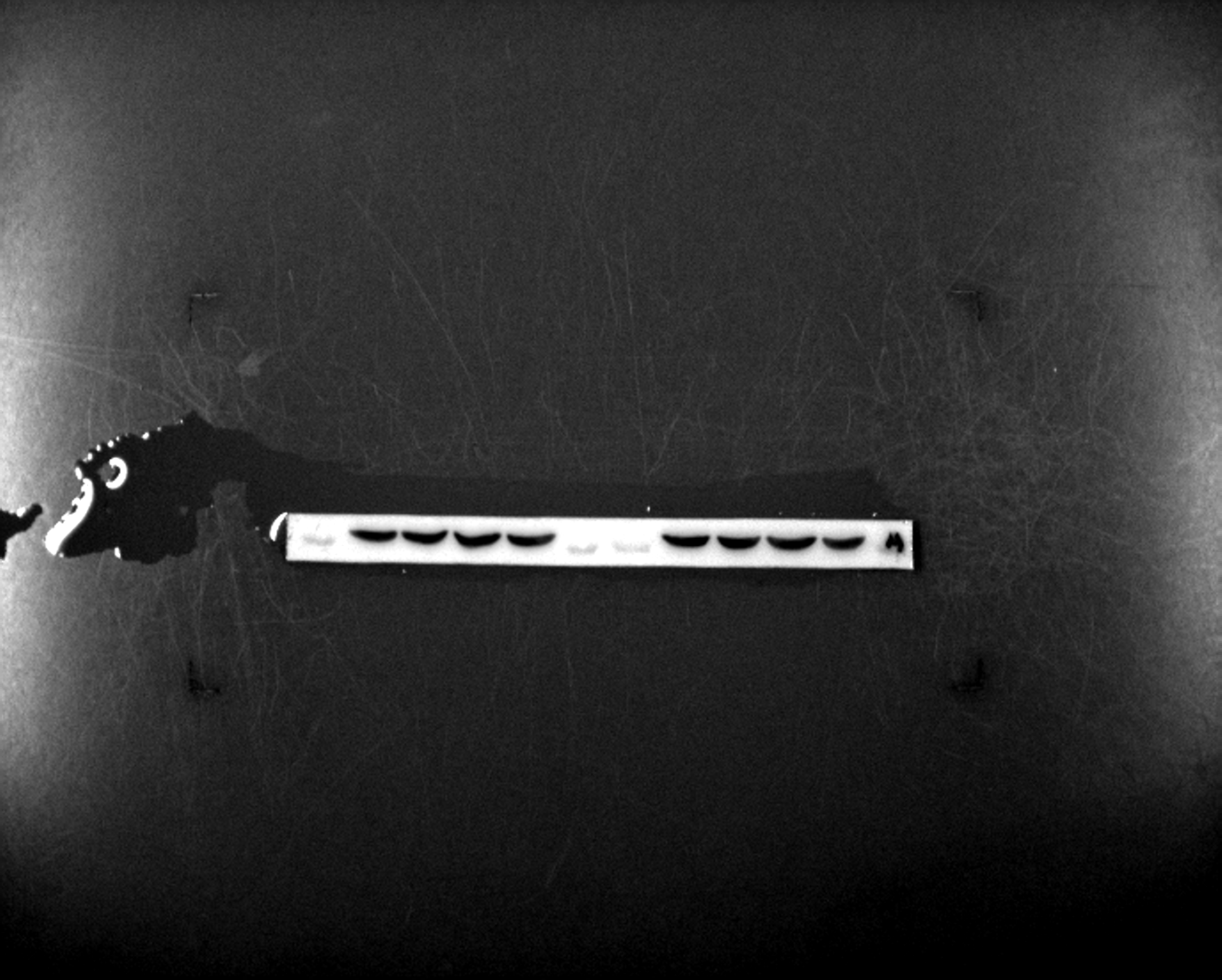

Supplement: Figure 7—source data 8. [file elife-96988-fig7-data8.zip › Figure 7-source data 8/Hepa1-6/5/β-ACTIN.Tif]

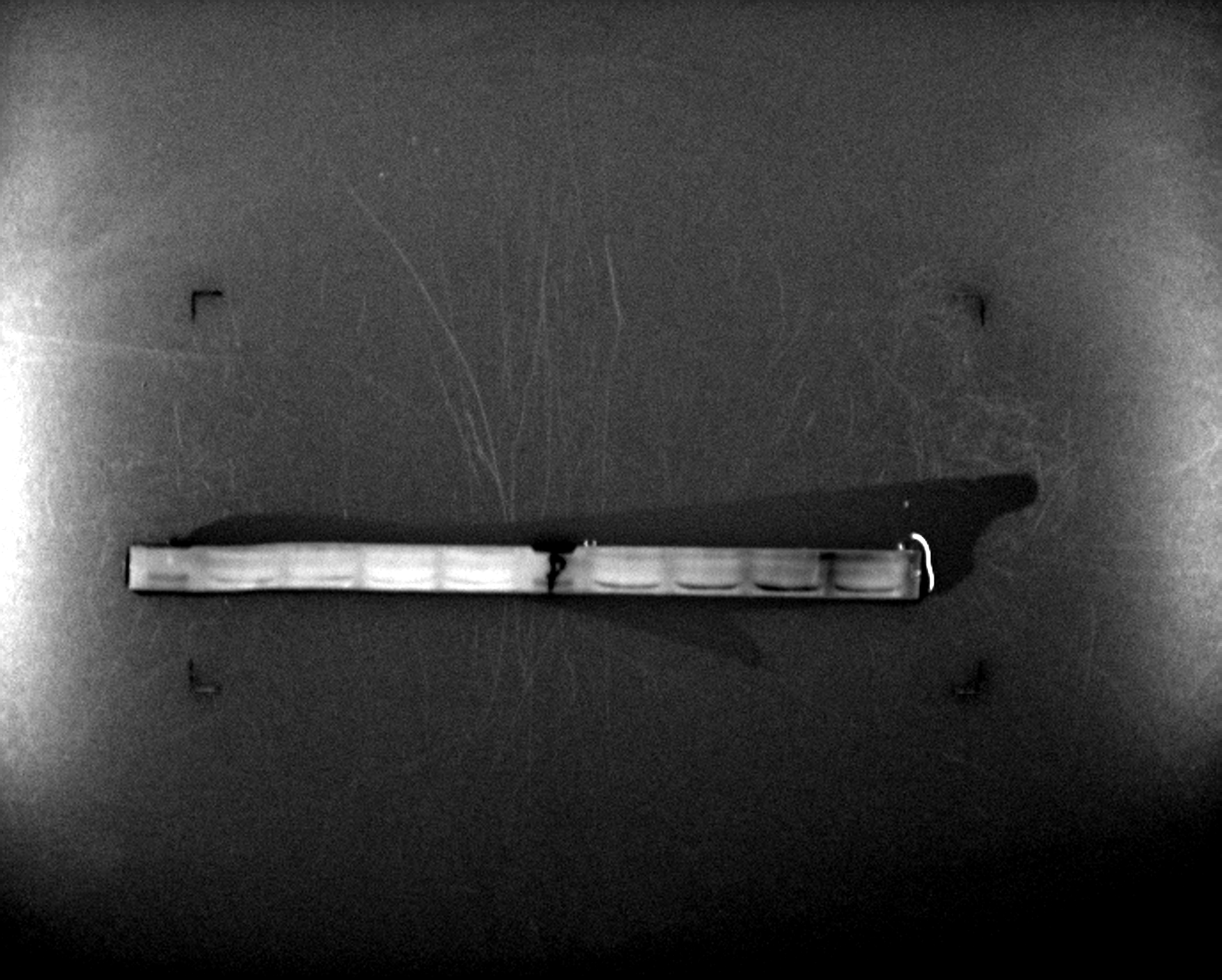

Supplement: Figure 7—source data 8. [file elife-96988-fig7-data8.zip › Figure 7-source data 8/Hepa1-6/6/PPARG.Tif]

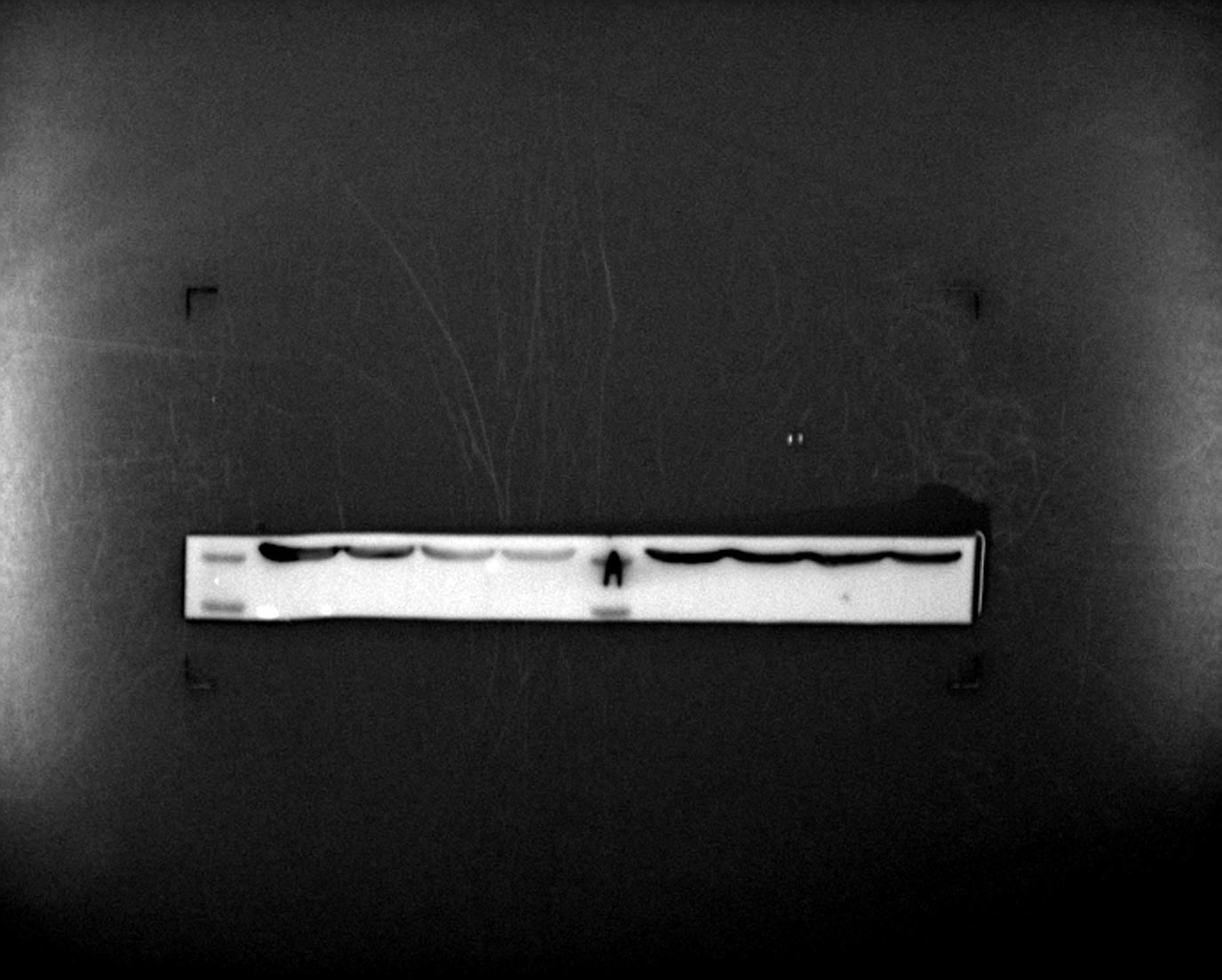

Supplement: Figure 7—source data 8. [file elife-96988-fig7-data8.zip › Figure 7-source data 8/Hepa1-6/6/β-ACTIN.Tif]

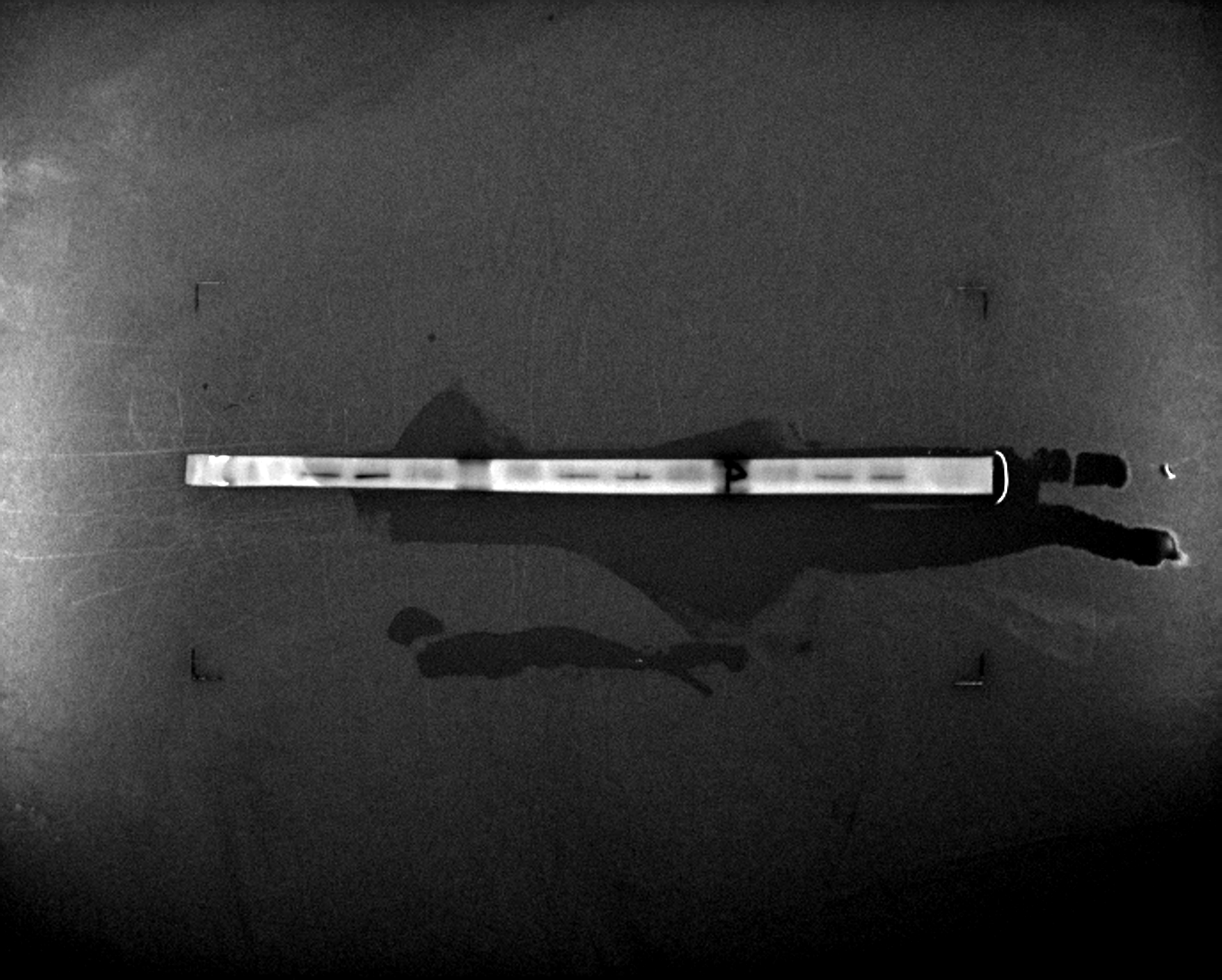

Supplement: Figure 7—source data 8. [file elife-96988-fig7-data8.zip › Figure 7-source data 8/MPH/1/PPARG.Tif]

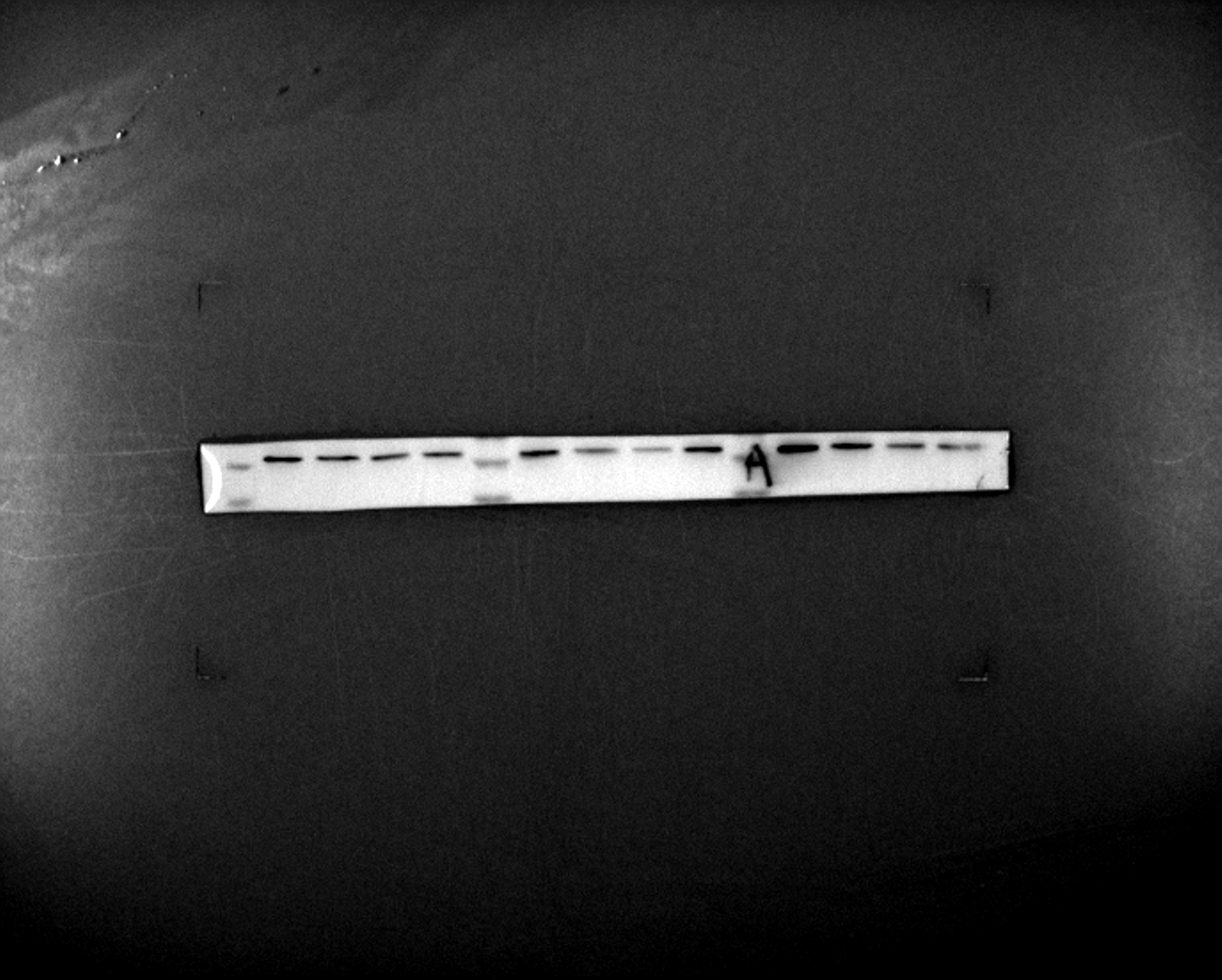

Supplement: Figure 7—source data 8. [file elife-96988-fig7-data8.zip › Figure 7-source data 8/MPH/1/β-ACTIN.Tif]

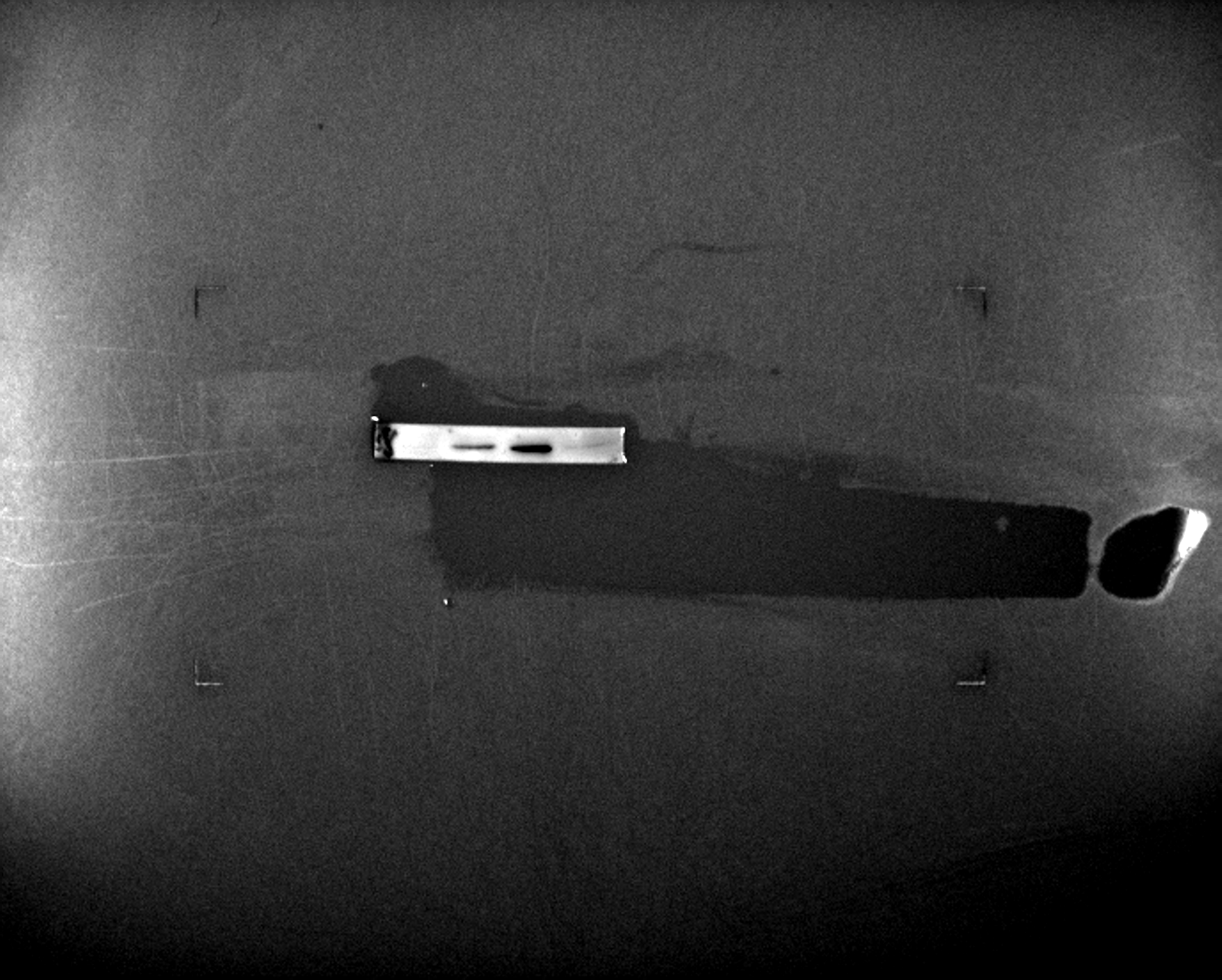

Supplement: Figure 7—source data 8. [file elife-96988-fig7-data8.zip › Figure 7-source data 8/MPH/2/PPARG.Tif]

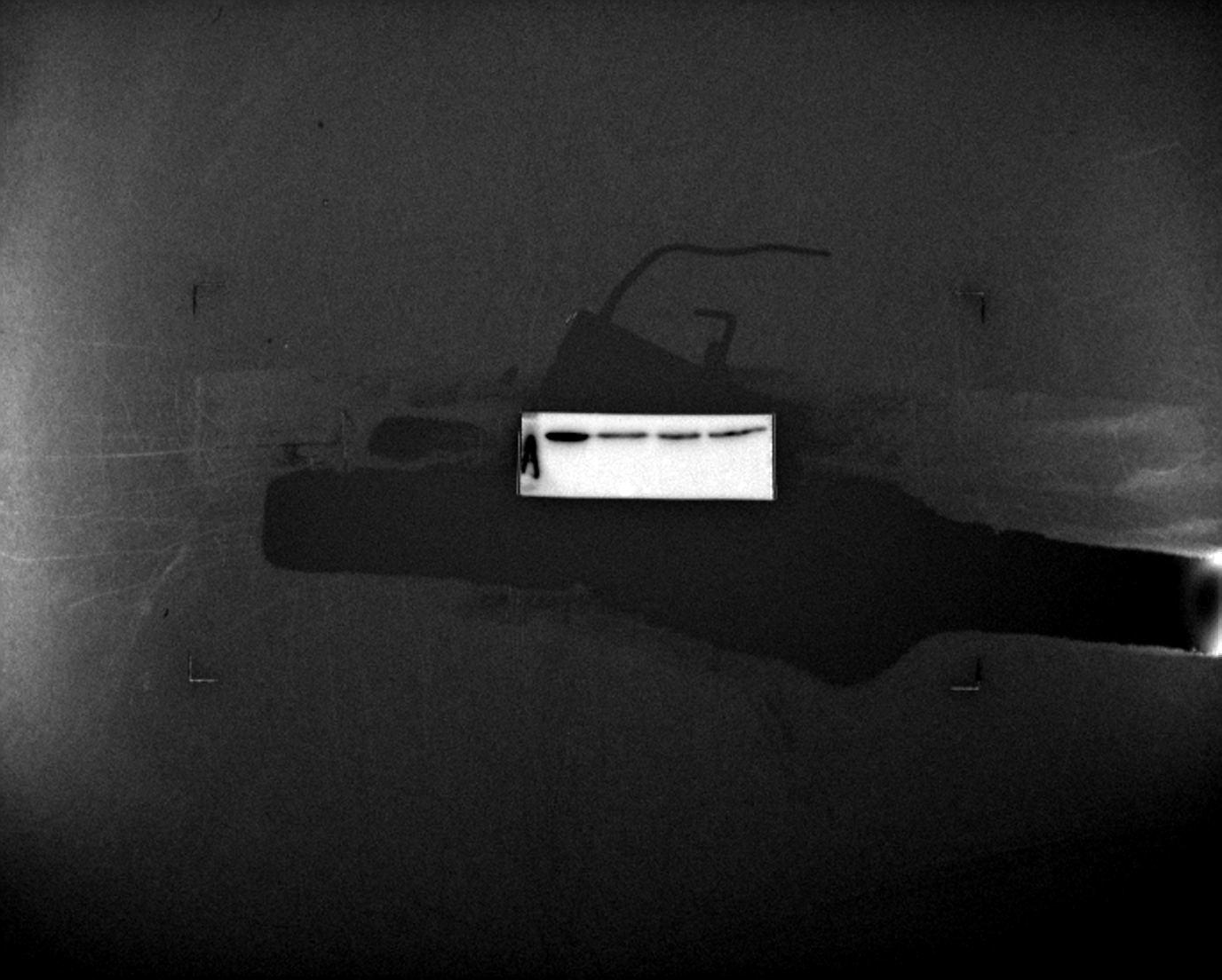

Supplement: Figure 7—source data 8. [file elife-96988-fig7-data8.zip › Figure 7-source data 8/MPH/2/β-ACTIN.Tif]

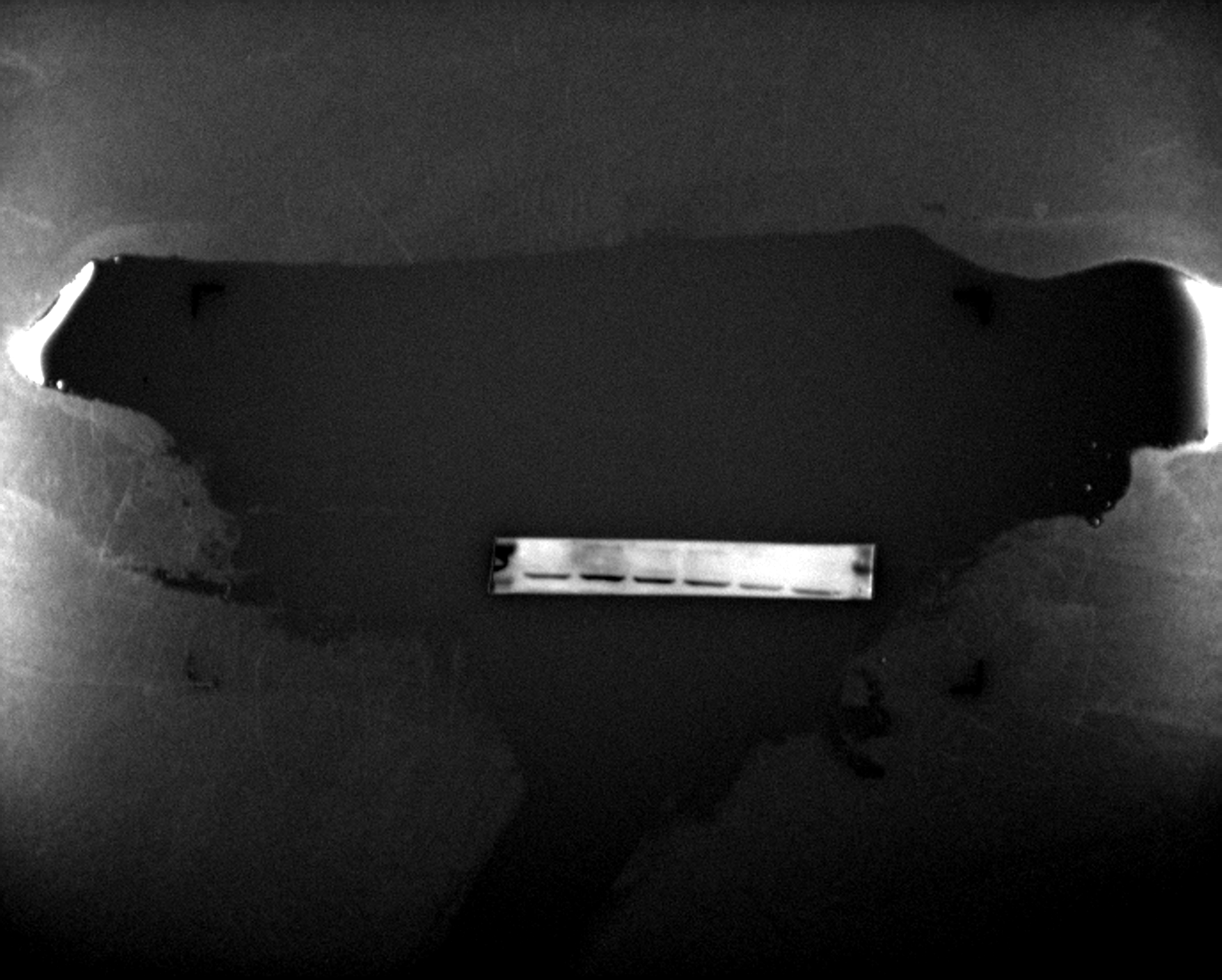

Supplement: Figure 7—source data 8. [file elife-96988-fig7-data8.zip › Figure 7-source data 8/MPH/3/PPARG.Tif]

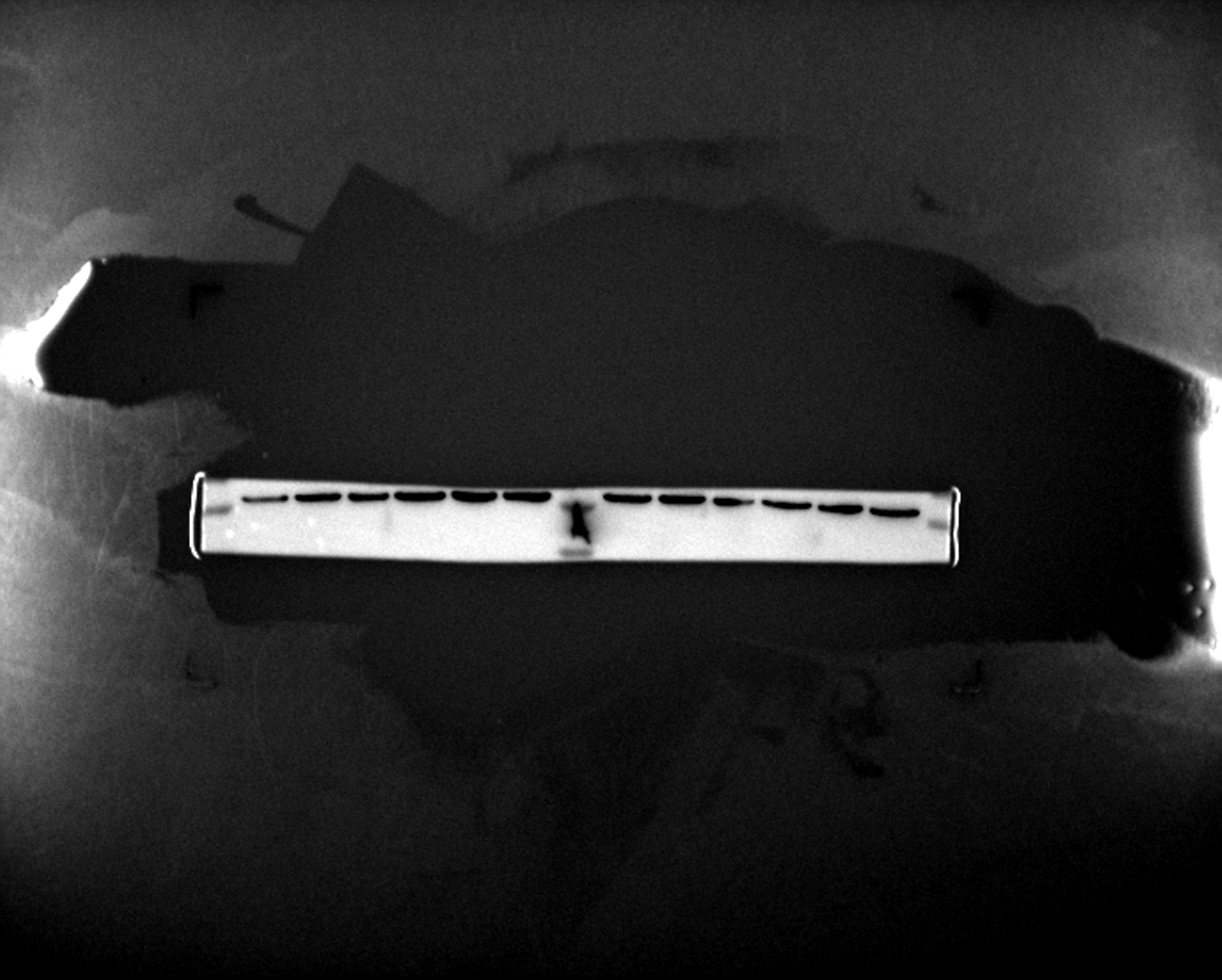

Supplement: Figure 7—source data 8. [file elife-96988-fig7-data8.zip › Figure 7-source data 8/MPH/3/β-ACTIN.Tif]

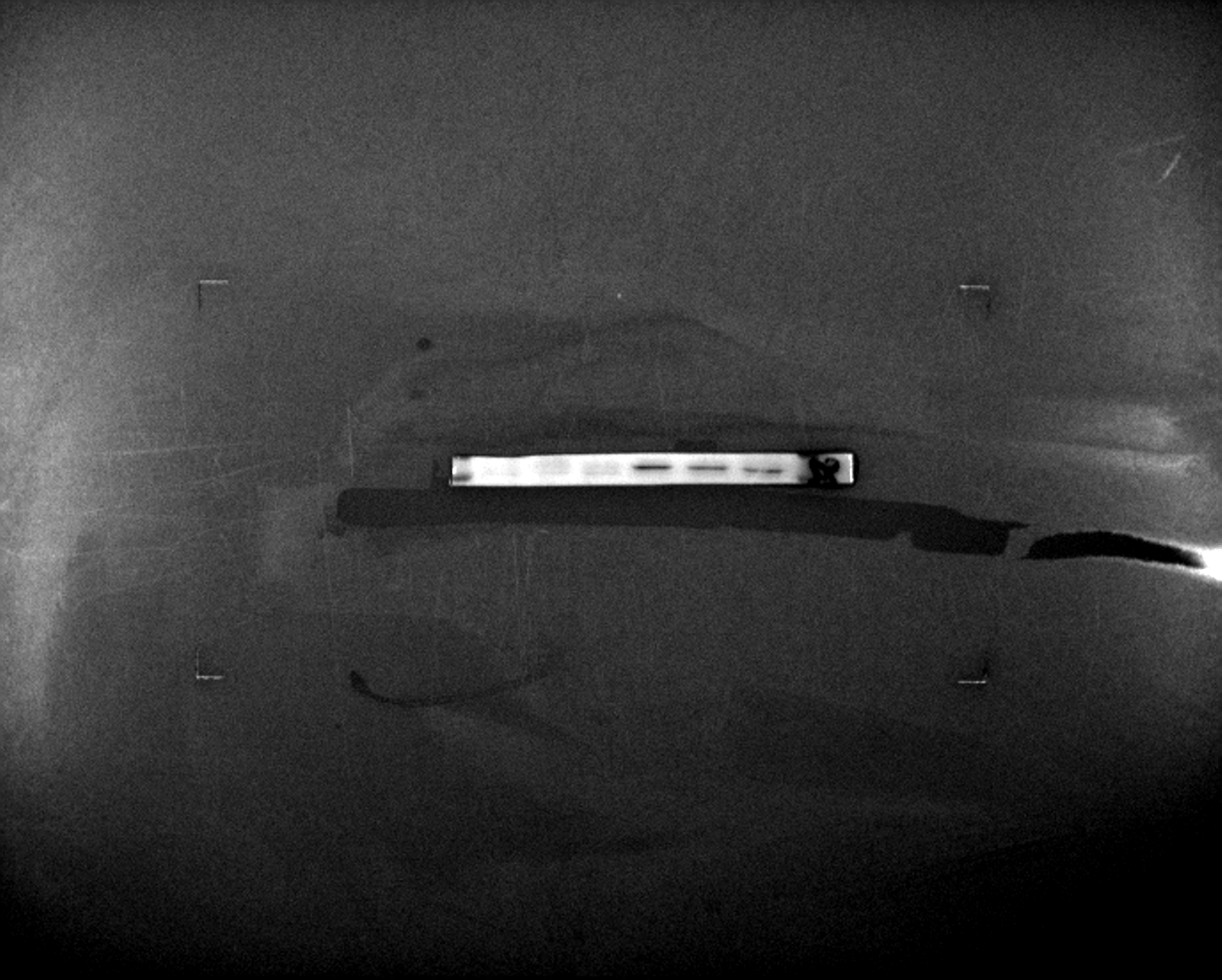

Supplement: Figure 7—source data 8. [file elife-96988-fig7-data8.zip › Figure 7-source data 8/MPH/4/PPARG.Tif]

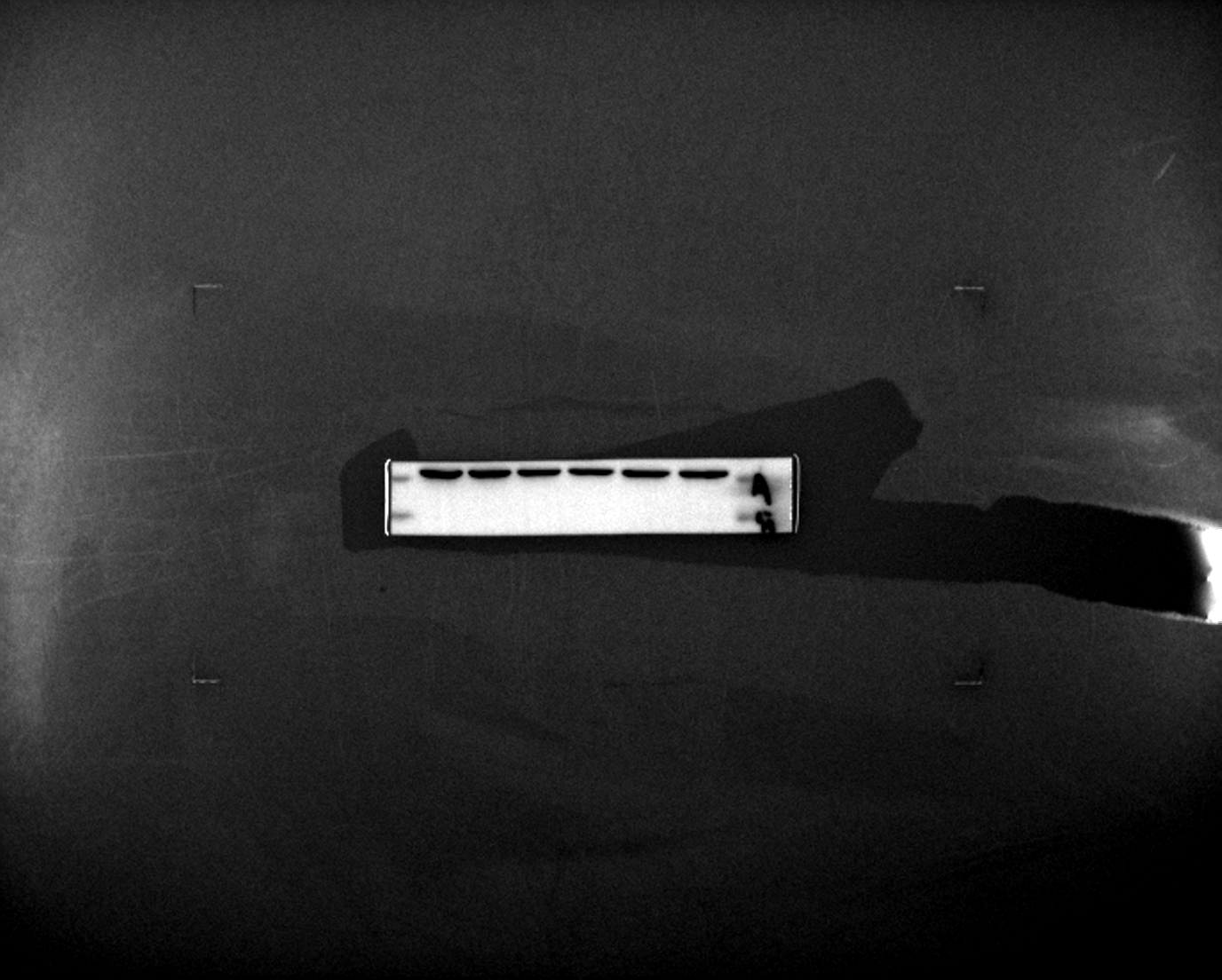

Supplement: Figure 7—source data 8. [file elife-96988-fig7-data8.zip › Figure 7-source data 8/MPH/4/β-ACTIN.Tif]

Figure suppl. 2C

1.

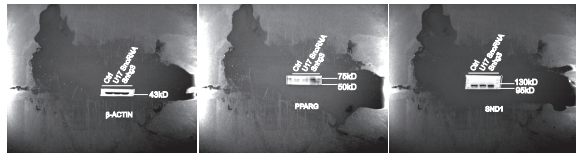

2.

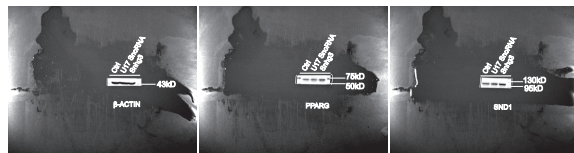

3.

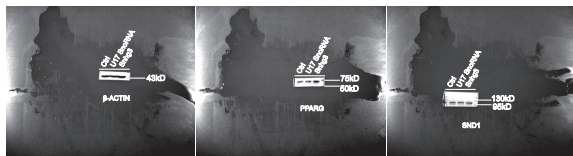

4.

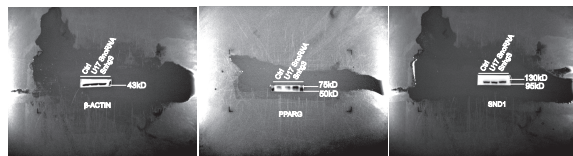

Supplement: Figure 7—figure supplement 1—source data 1. [file elife-96988-fig7-figsupp1-data1.zip › Figure 7-figure supplement 1-source data 1/Figure 7-figure suppl. 1-source data.pdf]

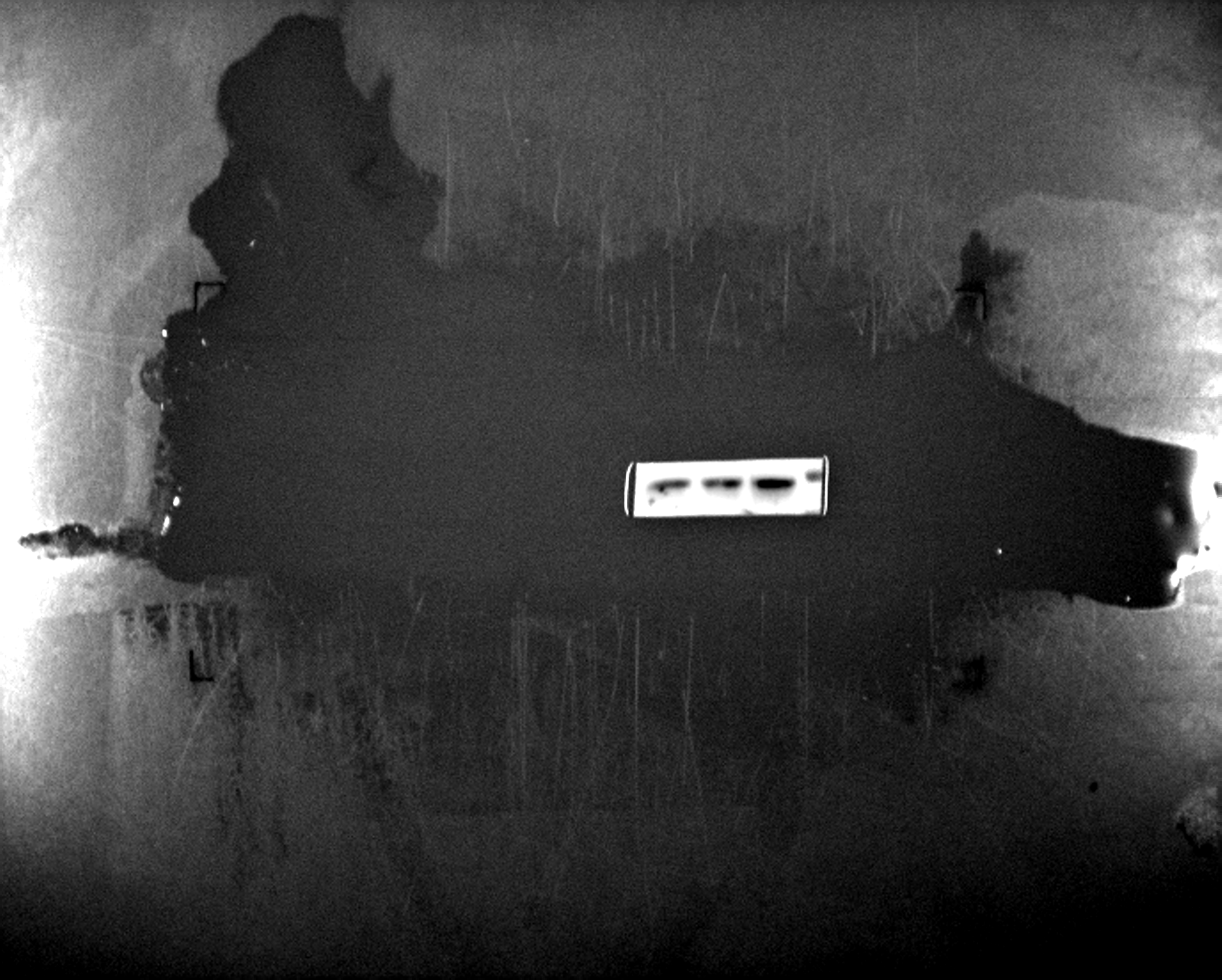

Supplement: Figure 7—figure supplement 1—source data 2. [file elife-96988-fig7-figsupp1-data2.zip › Figure 7-figure supplement 1-source data 2/1/PPARG.Tif]

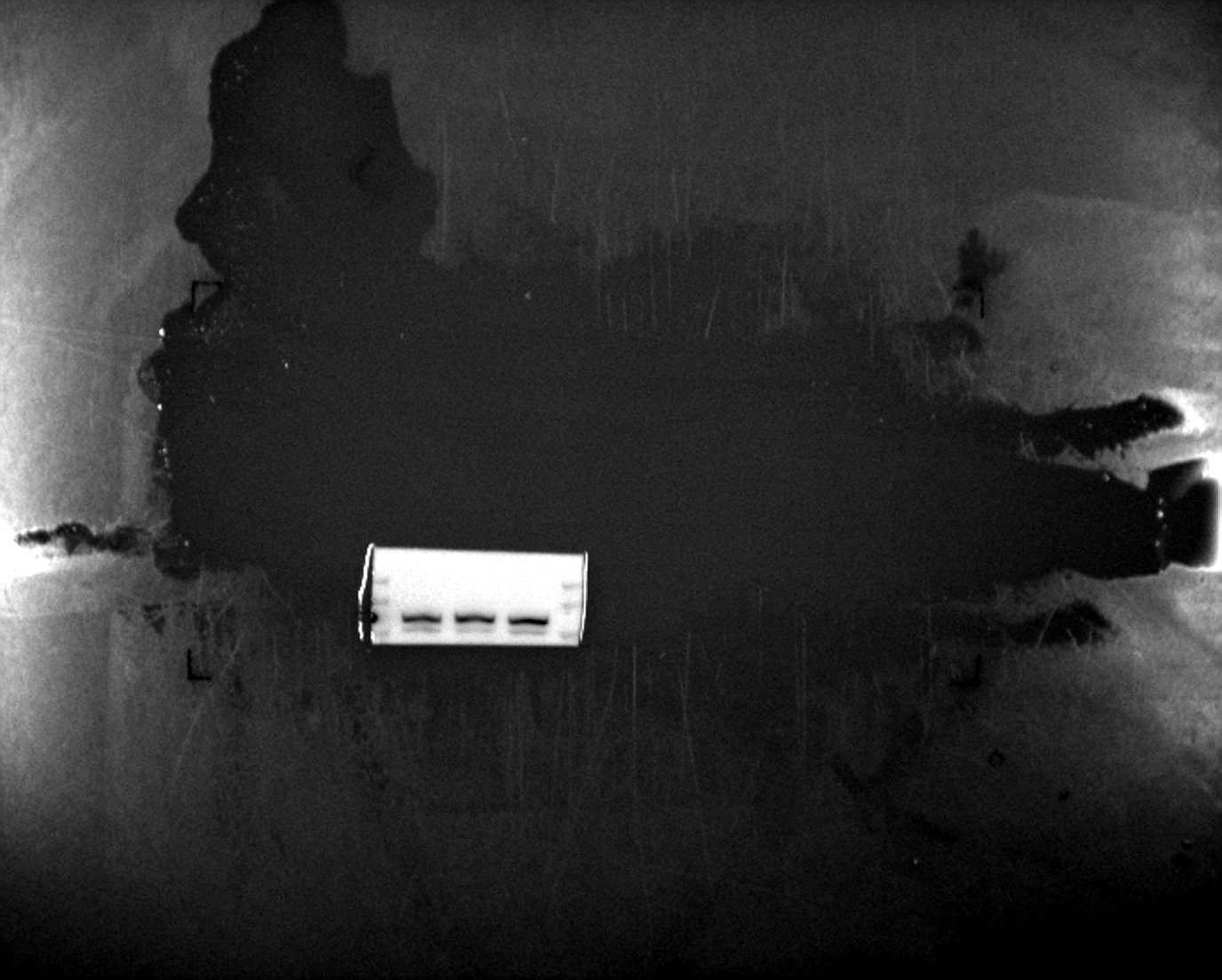

Supplement: Figure 7—figure supplement 1—source data 2. [file elife-96988-fig7-figsupp1-data2.zip › Figure 7-figure supplement 1-source data 2/1/SND1.Tif]

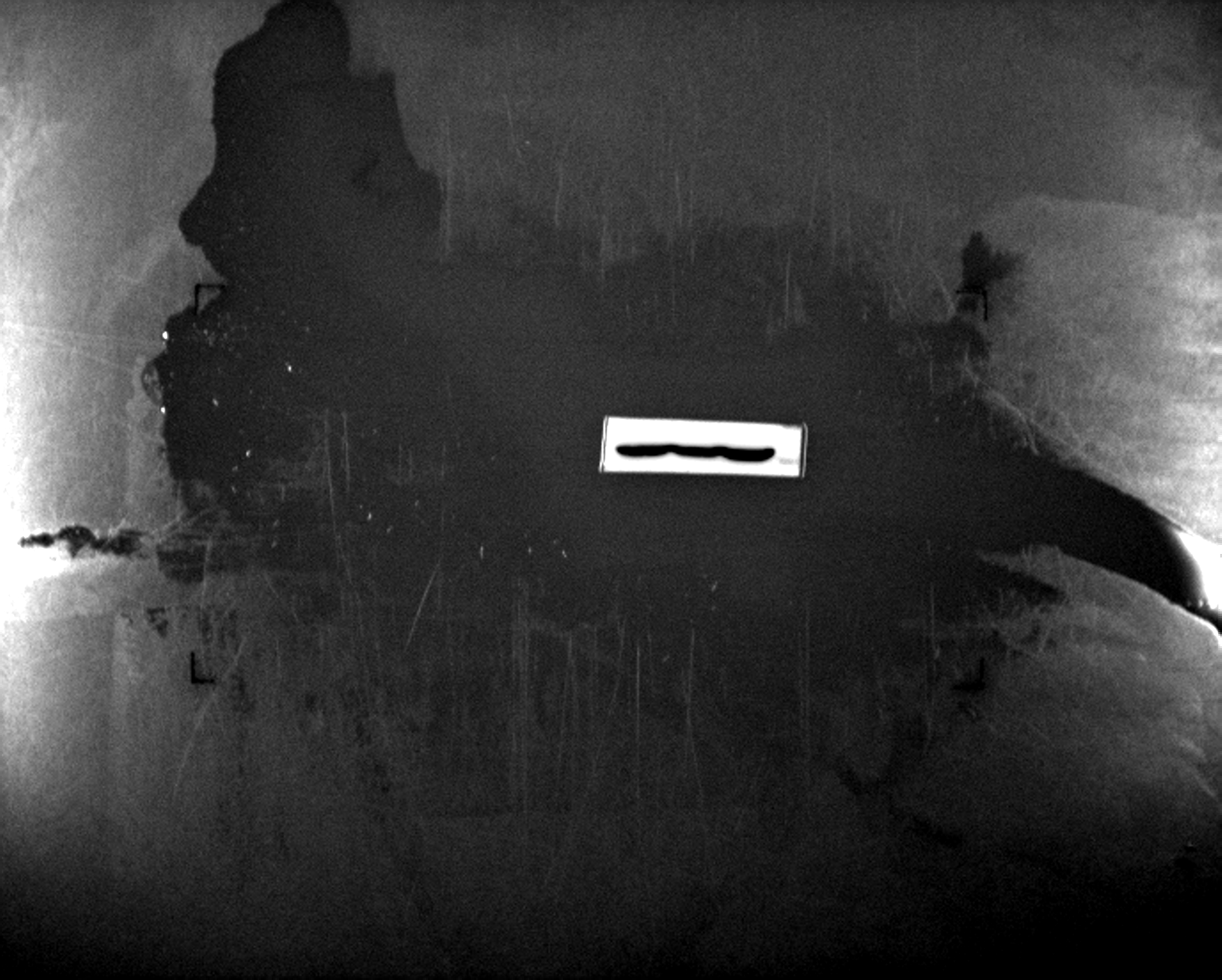

Supplement: Figure 7—figure supplement 1—source data 2. [file elife-96988-fig7-figsupp1-data2.zip › Figure 7-figure supplement 1-source data 2/1/β-ACTIN.Tif]

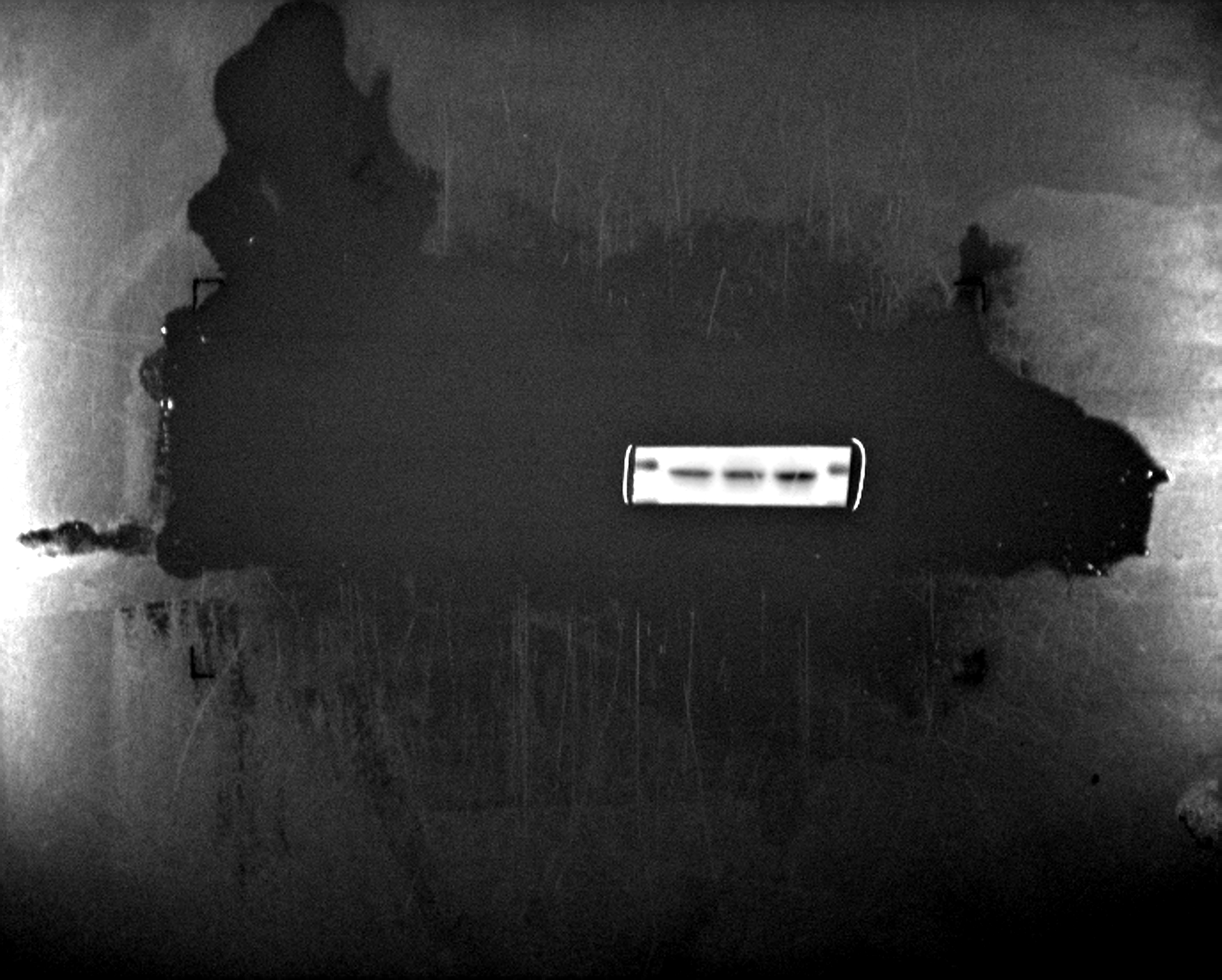

Supplement: Figure 7—figure supplement 1—source data 2. [file elife-96988-fig7-figsupp1-data2.zip › Figure 7-figure supplement 1-source data 2/2/PPARG.Tif]

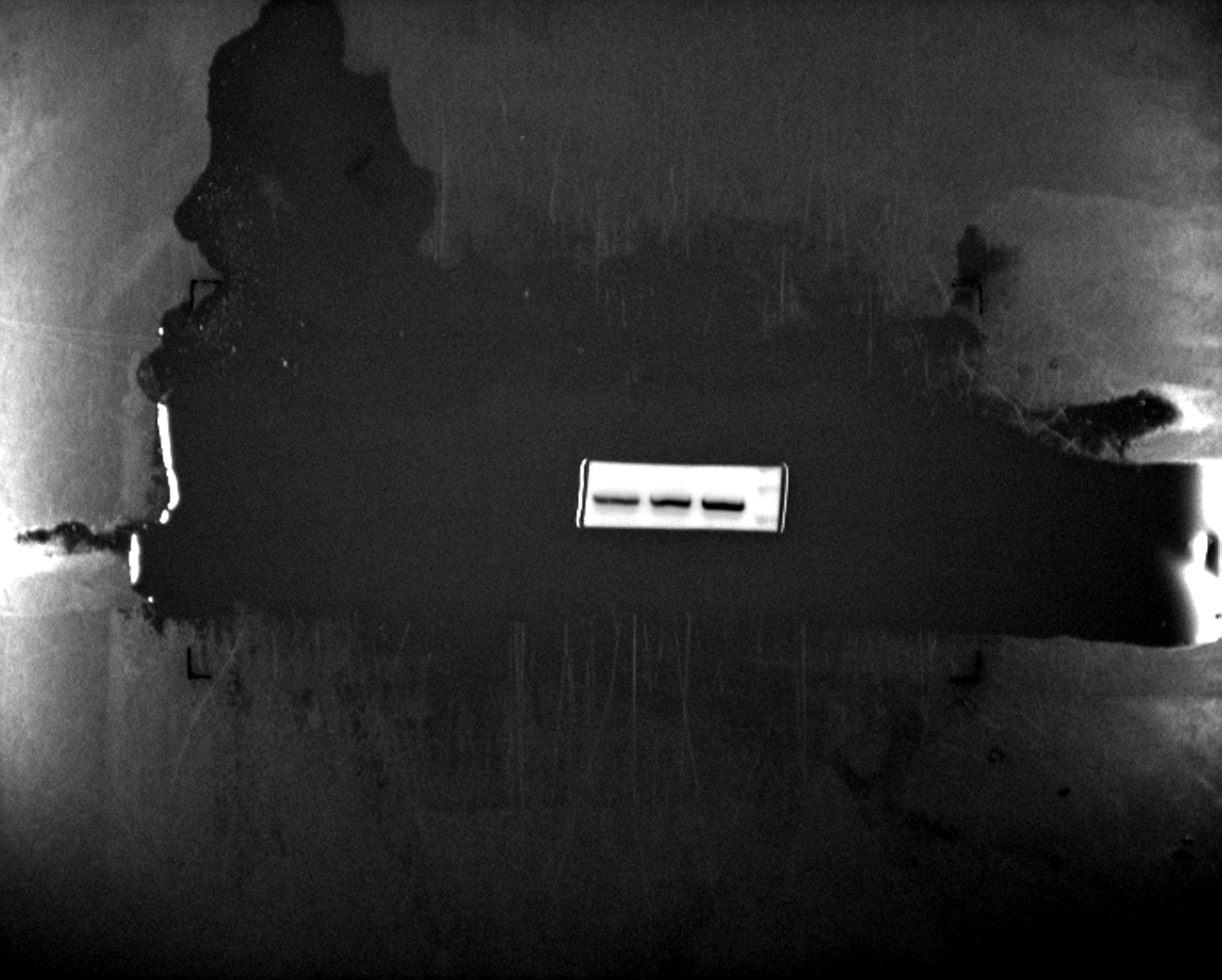

Supplement: Figure 7—figure supplement 1—source data 2. [file elife-96988-fig7-figsupp1-data2.zip › Figure 7-figure supplement 1-source data 2/2/SND1.Tif]

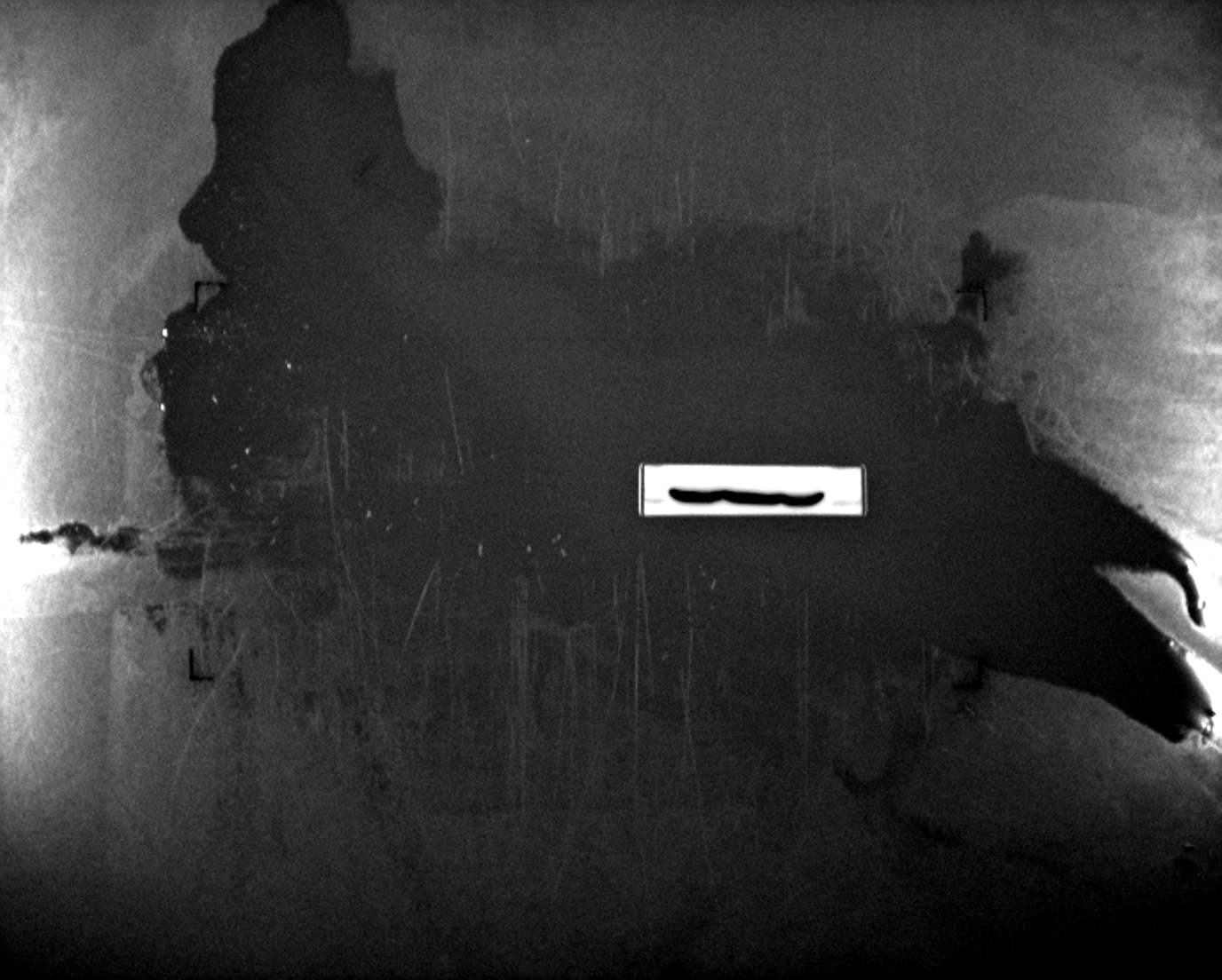

Supplement: Figure 7—figure supplement 1—source data 2. [file elife-96988-fig7-figsupp1-data2.zip › Figure 7-figure supplement 1-source data 2/2/β-ACTIN.Tif]

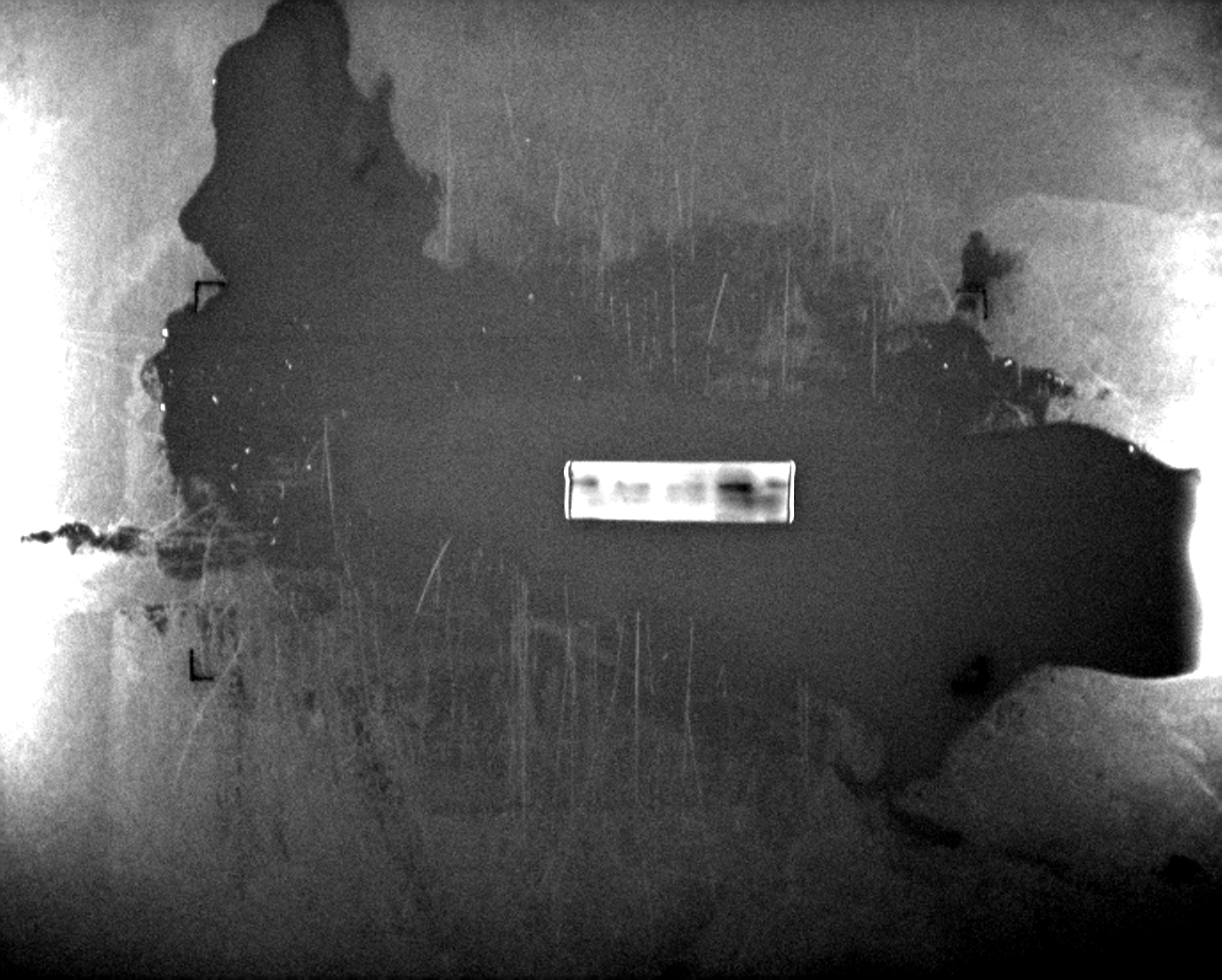

Supplement: Figure 7—figure supplement 1—source data 2. [file elife-96988-fig7-figsupp1-data2.zip › Figure 7-figure supplement 1-source data 2/3/PPARG.Tif]

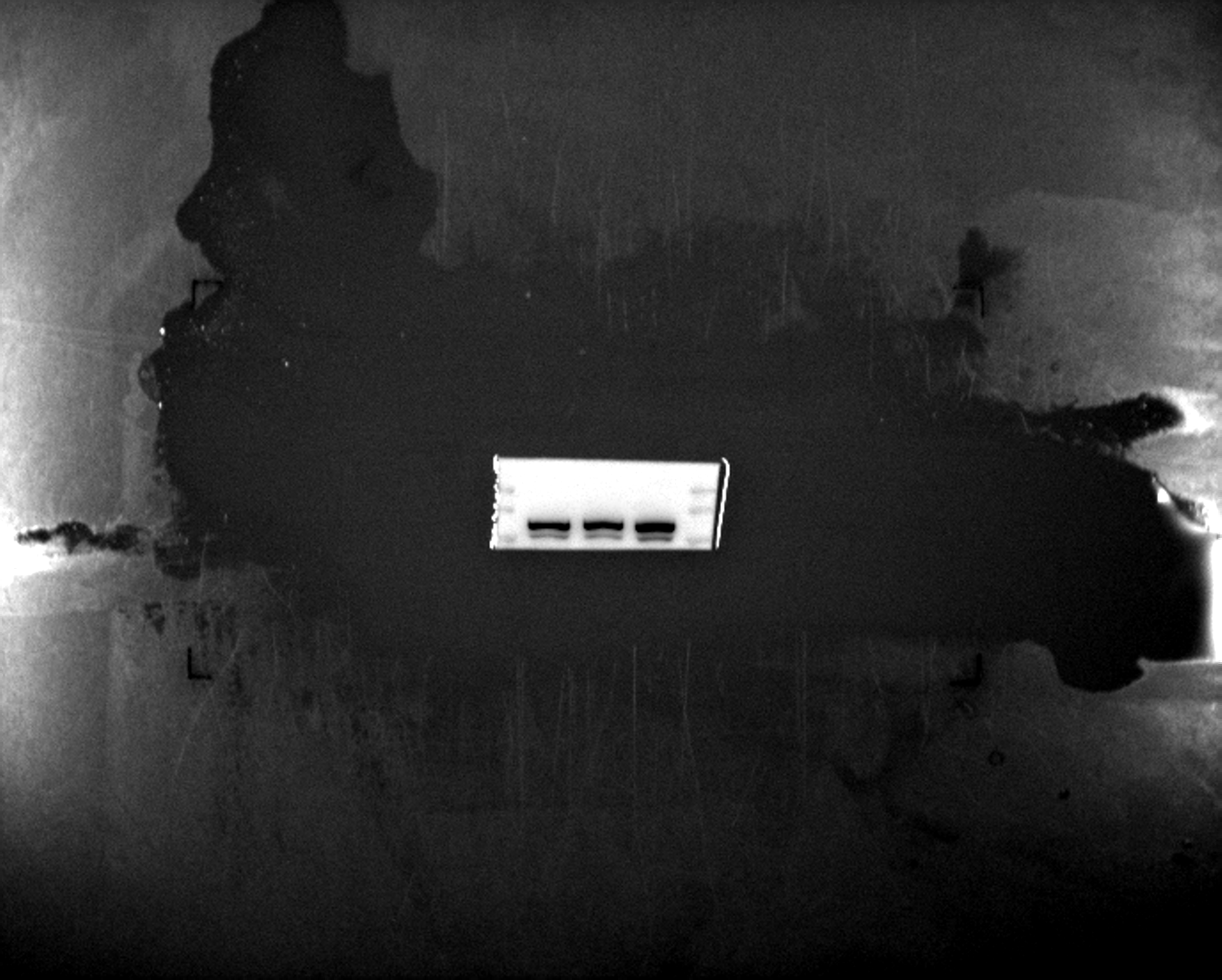

Supplement: Figure 7—figure supplement 1—source data 2. [file elife-96988-fig7-figsupp1-data2.zip › Figure 7-figure supplement 1-source data 2/3/SND1.Tif]

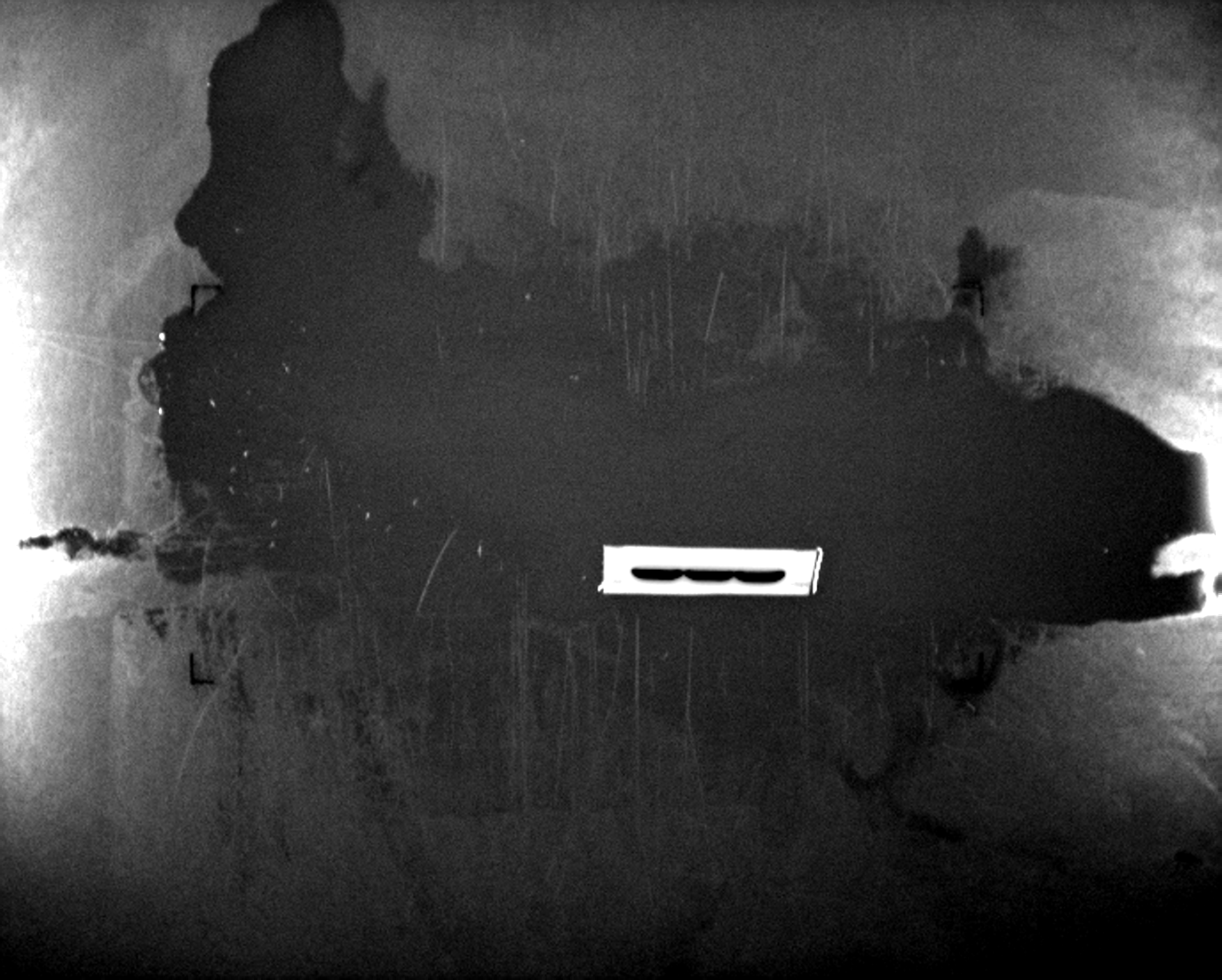

Supplement: Figure 7—figure supplement 1—source data 2. [file elife-96988-fig7-figsupp1-data2.zip › Figure 7-figure supplement 1-source data 2/3/β-ACTIN.Tif]

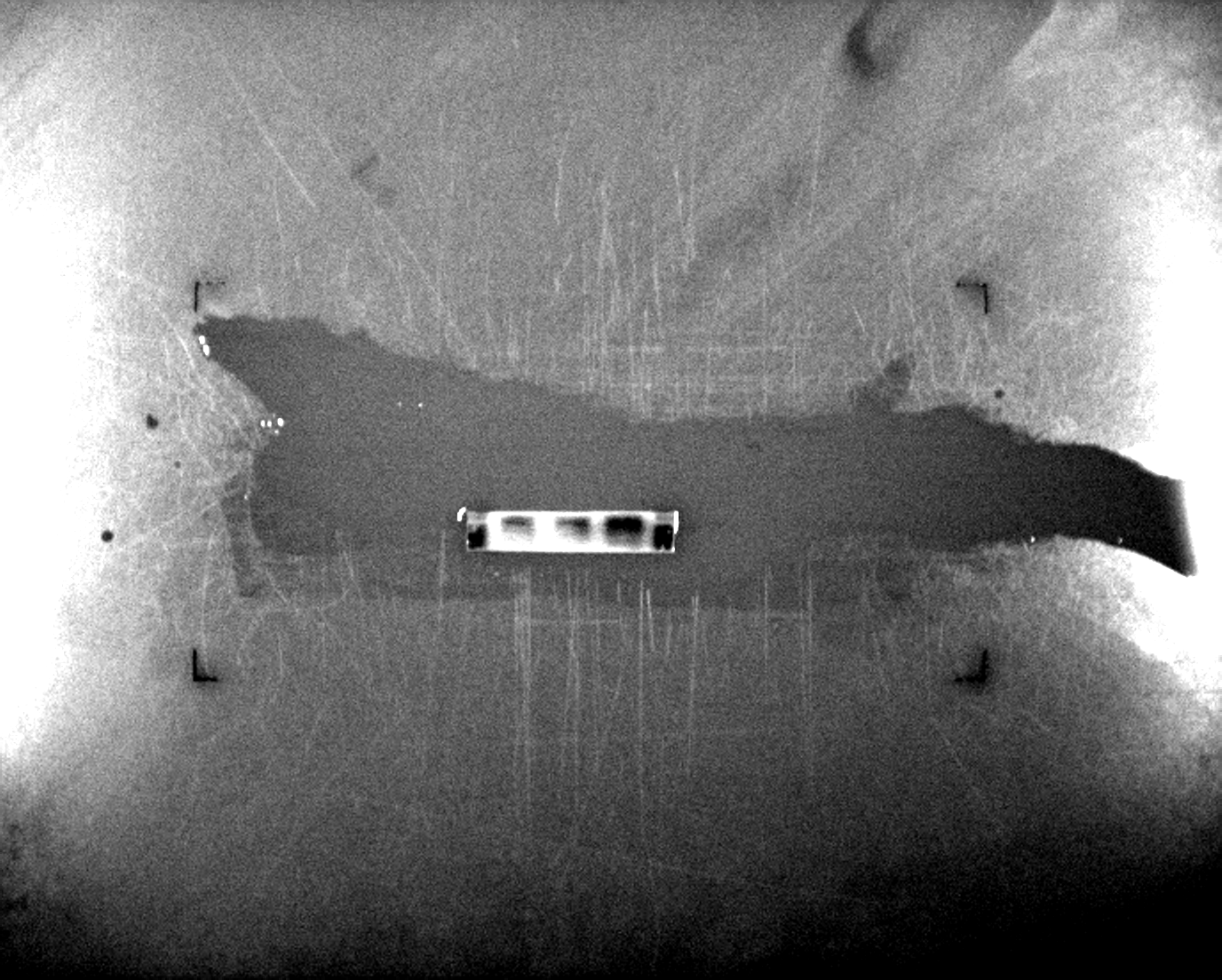

Supplement: Figure 7—figure supplement 1—source data 2. [file elife-96988-fig7-figsupp1-data2.zip › Figure 7-figure supplement 1-source data 2/4/PPARG.Tif]

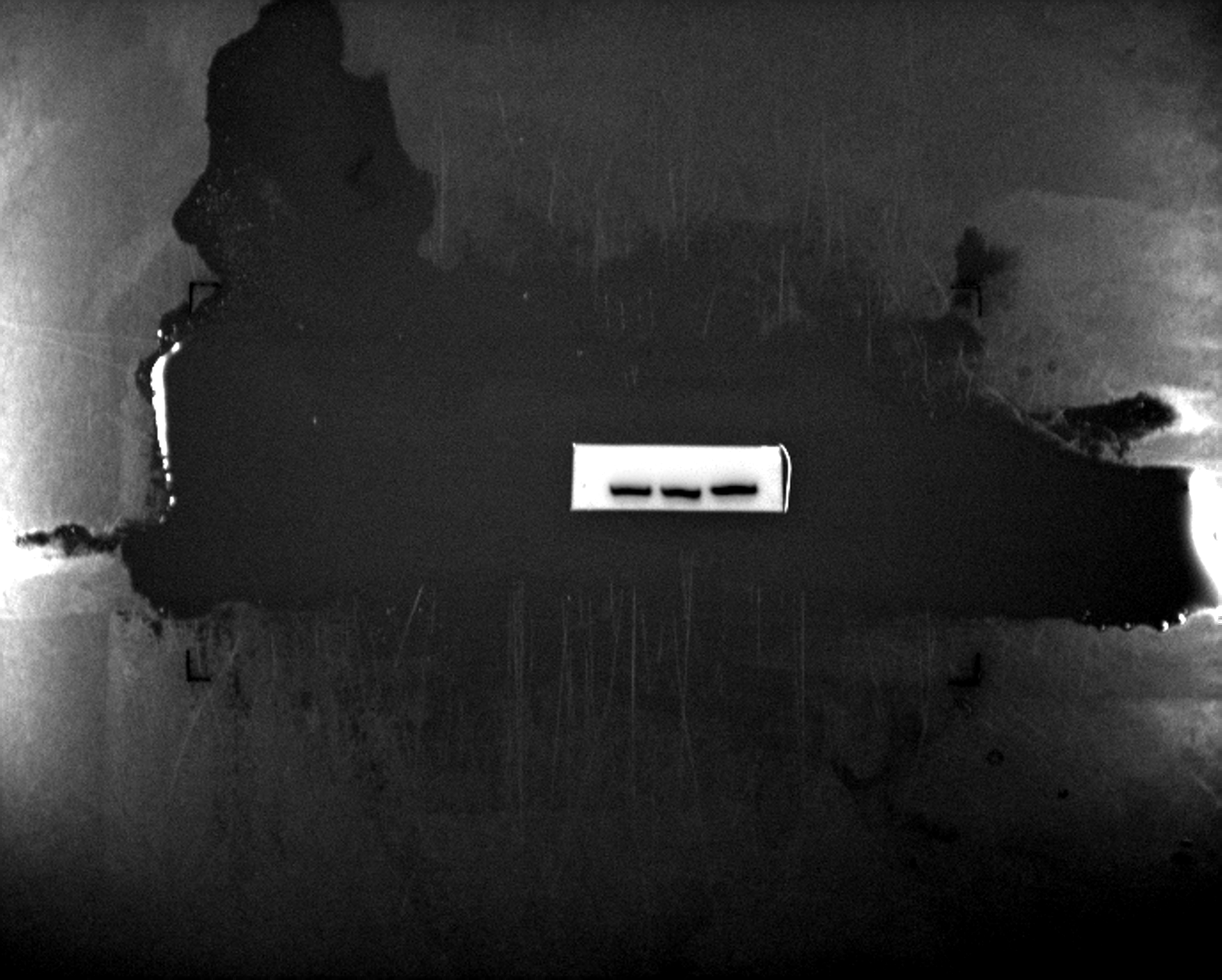

Supplement: Figure 7—figure supplement 1—source data 2. [file elife-96988-fig7-figsupp1-data2.zip › Figure 7-figure supplement 1-source data 2/4/SND1.Tif]

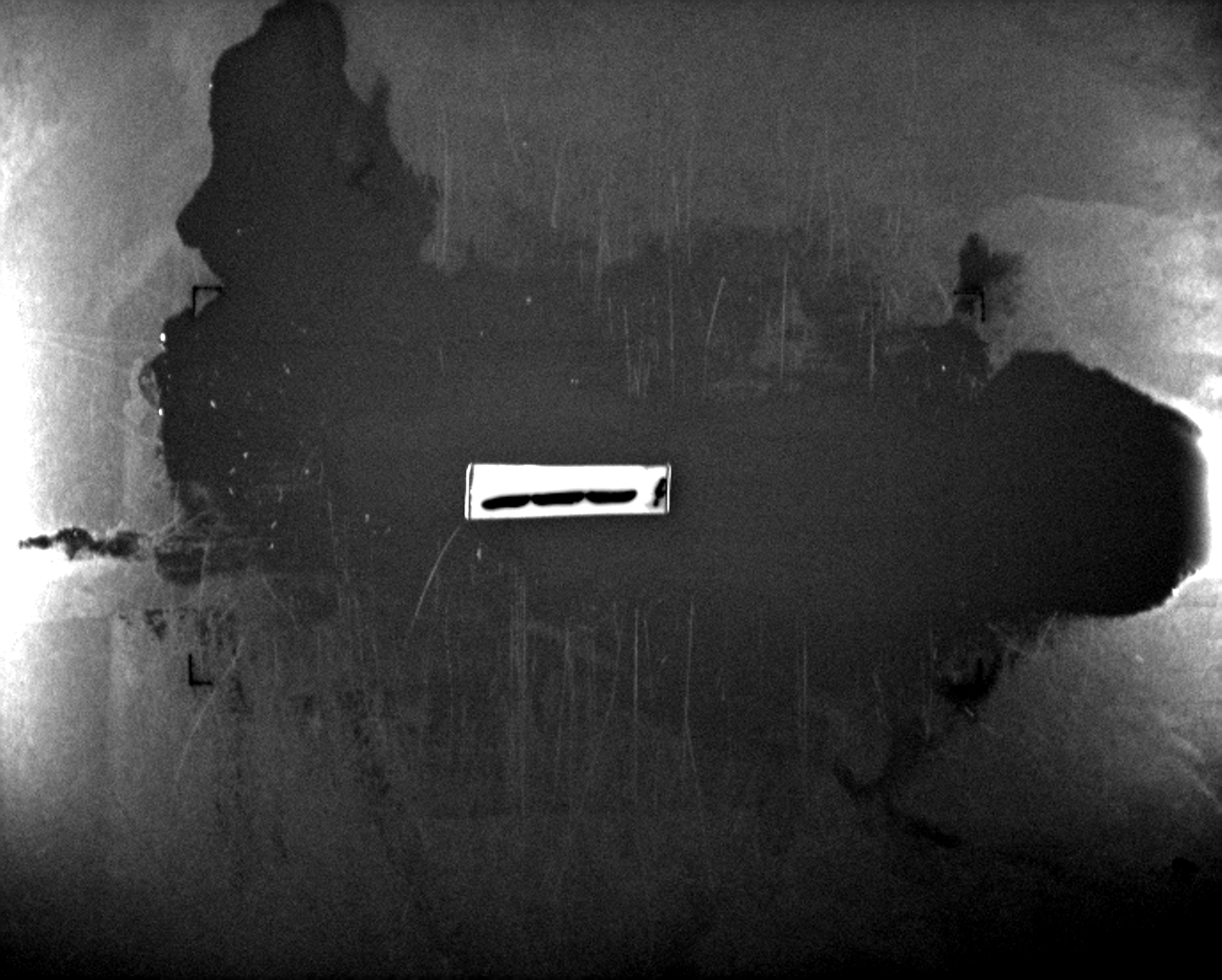

Supplement: Figure 7—figure supplement 1—source data 2. [file elife-96988-fig7-figsupp1-data2.zip › Figure 7-figure supplement 1-source data 2/4/β-ACTIN.Tif]
